# Design and Synthesis of Covalent Inhibitors of FabA

**DOI:** 10.1021/acsomega.2c08031

**Published:** 2023-03-27

**Authors:** James S. Martin, Claire J. Mackenzie, De Lin, Nadine Homeyer, David W. Gray, Fabio Zuccotto, Ian H. Gilbert

**Affiliations:** Wellcome Centre for Anti-Infectives Research, Division of Biological Chemistry and Drug Discovery, University of Dundee, Dundee DD1 5EH, United Kingdom

## Abstract

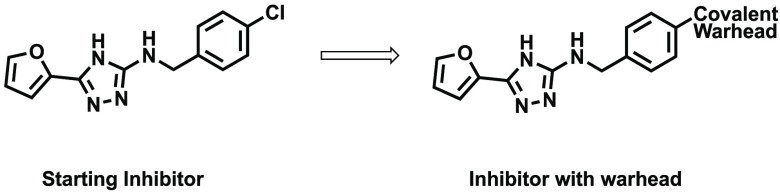

There is an urgent need for the development of new therapeutics with novel modes of action to target Gram-negative bacterial infections, due to resistance to current drugs. Previously, FabA, an enzyme in the bacterial type II fatty acid biosynthesis pathway, was identified as a potential drug target in *Pseudomonas aeruginosa*, a Gram-negative bacteria of significant clinical concern. A chemical starting point was also identified. There is a cysteine, Cys15, in the active site of FabA, adjacent to where this compound binds. This paper describes the preparation of analogues containing an electrophilic warhead with the aim of covalent inhibition of the target. A wide variety of analogues were successfully prepared. Unfortunately, these analogues did not increase inhibition, which may be due to a loop within the enzyme partially occluding access to the cysteine.

## Introduction

*Pseudomonas aeruginosa* (*P. aeruginosa*) is a Gram-negative, rod-shaped bacteria that is capable of infecting humans. *P. aeruginosa* is an opportunistic infection, which causes an array of life-threatening infections in immunocompromised patients.^1,2^*P. aeruginosa* has developed resistance to multiple classes of antibiotics and is emerging as a worldwide public health threat, so new treatments are urgently needed.^1^ To overcome issues with resistance, there is a need for novel classes of antibacterials which work by mechanisms differentiated from current antibiotics.^3^

**Figure 1:**
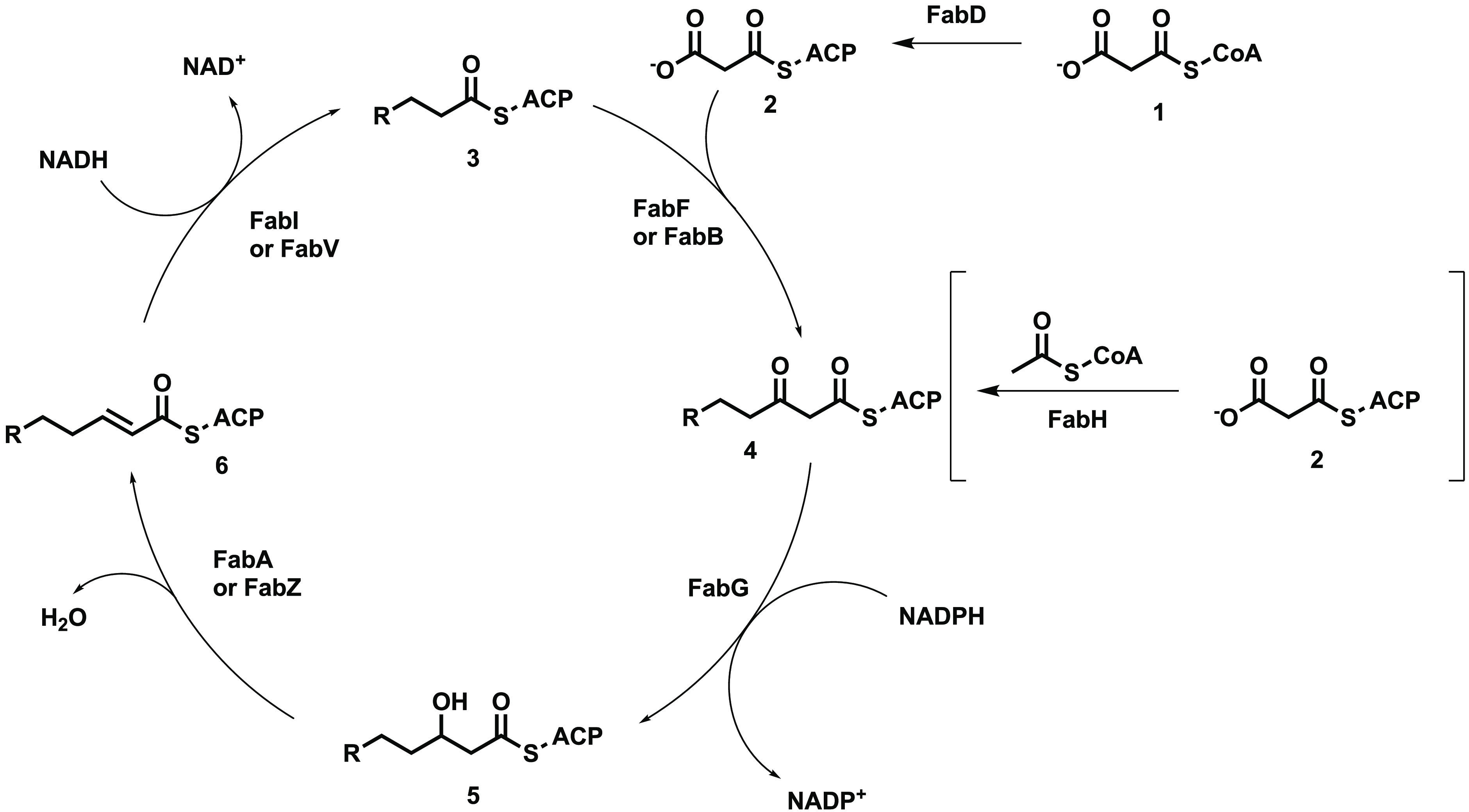
Type II fatty acid biosynthesis pathway. ACP, acyl carrier protein; CoA, coenzyme A; Fab, fatty acid biosynthesis; NAD, nicotinamide adenine dinucleotide; NADH, reduced nicotinamide adenine dinucleotide; NADP, nicotinamide adenine dinucleotide phosphate; NADPH, reduced nicotinamide adenine dinucleotide phosphate.

Fatty acids are an essential component of all cells; however, the bacterial type II fatty acid biosynthesis pathway (FASII) is sufficiently different from the type I pathway used in eukaryotic cells that FASII has become an attractive target for antibiotic research.^4,5^ In the FASII pathway, fatty acids are synthesized in a stepwise manner attached to acyl-carrier-protein (ACP) by a series of enzymes as shown in Figure 1.

**Figure 2:**
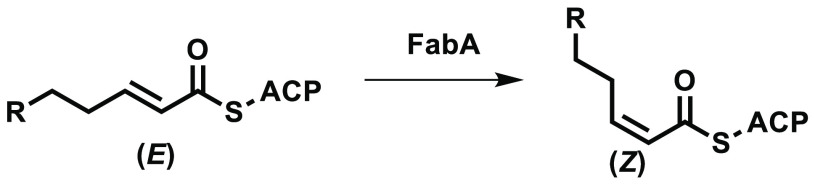
FabA is also able to isomerize *E* fatty acids to *Z* fatty acids. This is utilized to synthesize specific unsaturated fatty acids.

First, FabD converts malonyl-coenzyme A (malonyl-CoA) **1** into malonyl-acyl carrier protein (malonyl-ACP) **2**. The product from this is then used in one of two steps, either FabH catalyzes the initiation step where malonyl-ACP **2** is condensed with acetyl-CoA, or FabB or FabF condenses malonyl-ACP **2** with the growing fatty acid chain **3** to give the ketone **4**. Regardless, the next step involves the reduction of the ketone **4** to the alcohol **5** by FabG using NADPH. Either FabA or FabZ are able to dehydrate this alcohol **5** to the alkene **6**. Finally, FabI or FabV reduces the alkene **6** to the alkane **4** using NADH, and the cycle repeats to further lengthen the fatty acid chain.^6^ Many of the enzymes involved in this pathway have previously been investigated as potential antibacterial targets including FabH,^7^ FabG,^8,9^ FabZ,^9^ FabI,^9−11^ and FabF.^12^ FabA is also able to isomerize *E* fatty acids to the corresponding *Z* isomer^6^ as shown in Figure 2.

Although both FabZ and FabA are able to catalyze the dehydration shown in Figure 1, only FabA is able to catalyze this isomerization due to subtle changes in the structure of the binding site.^6,13^ FabA is therefore an essential enzyme in the synthesis of unsaturated fatty acids.^14^

**Figure 3:**
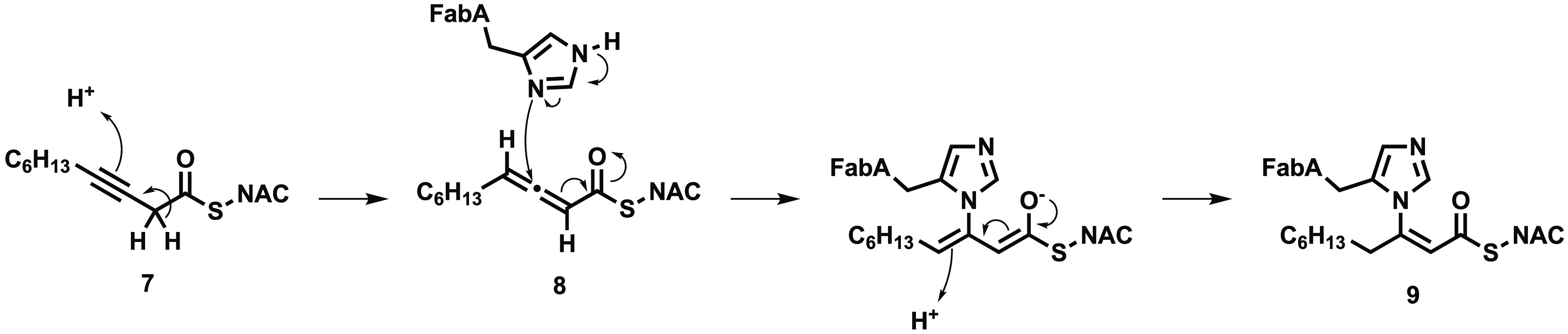
A covalent inhibitor **7** of FabA was developed and used as a tool to investigate its activity. NAC, *N*-acetylcysteine.

A covalent inhibitor **7** of FabA has been reported, which is able to react with a histidine residue, His70, in the active site of the enzyme as shown in Figure 3.^14^

First the alkyne **7** is converted to the allene **8** by FabA; this intermediate is then able to alkylate His70 via a Michael addition to give **9** and inactivate the enzyme.^14^ Although this compound covalently inhibits FabA, it was designed as a research tool, not a drug, and likely lacks the properties required to reach the site of action *in vivo* due to its high molecular weight and lipophilicity. The goal of this work was to investigate the development of a covalent inhibitor of *P. aeruginosa* FabA that can be used as a tool but also has the physiochemical properties that would make it more suited as a precursor to a novel antibacterial drug.

## Constrained Linkers to Covalent Warhead

**Figure 4:**
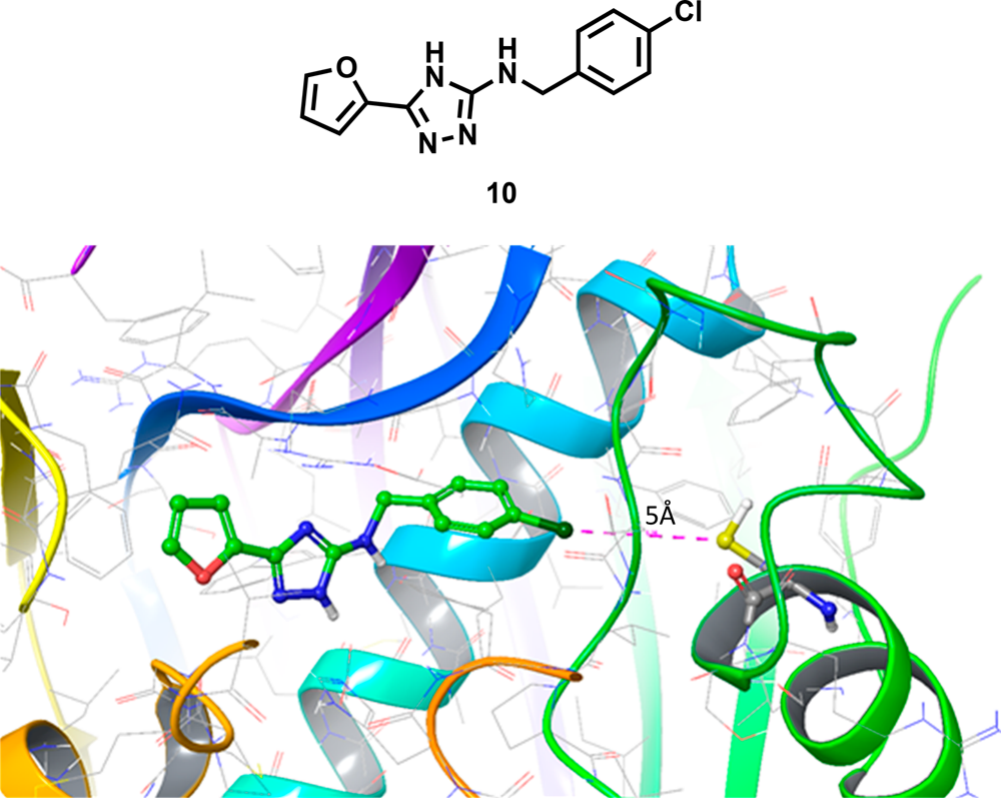
X-ray crystal structure of known inhibitor **10** bound to FabA. Cys15, the highlighted cysteine residue, is 5 Å from the chlorine atom. PDB 4CL6.^13^

Previously, an in-house, high-throughput screen had identified compound **10** (Figure 4) as a potent inhibitor of FabA, IC_50_ = 2.3 μM.^13^ The crystal structure of compound **10** bound to FabA revealed that the chlorine atom sat approximately 5 Å from Cys15, which provided a potential target for a covalent warhead (Figure 4).

**Scheme 1:**
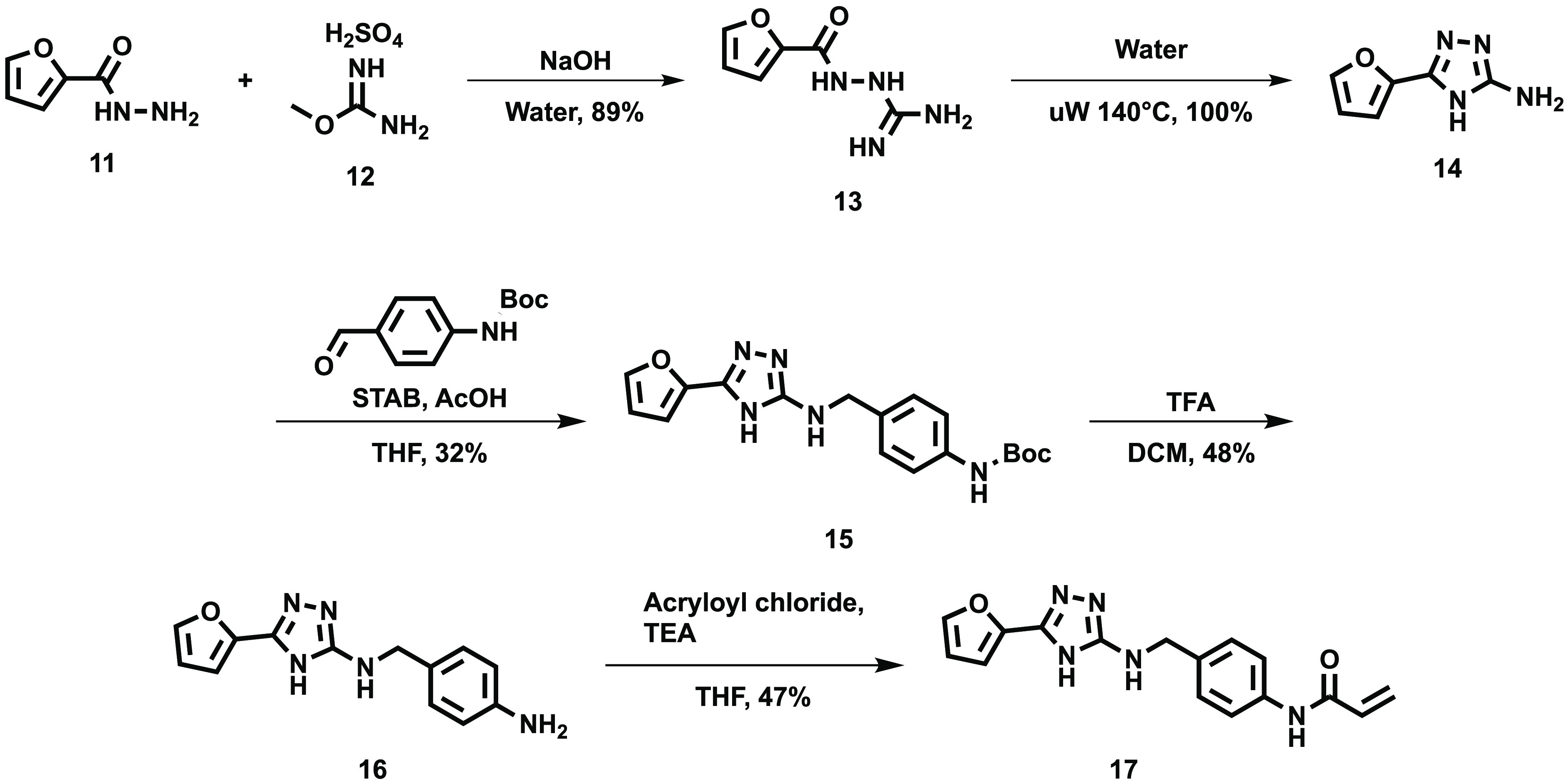
General Synthesis of Acrylamide Compounds

To take advantage of this, several compounds were designed where the left-hand side of the compound, featuring the furan ring and the triazole ring of **10**, was retained and the right-hand side altered with the intention of positioning a covalently reactive group in the correct orientation to react with Cys15. This synthesis is shown in Scheme 1.

Furan carbohydrazide **11** was reacted with methylisourea sulfate **12** in aqueous sodium hydroxide to give **13**. The intermediate was suspended in water and heated to 140 °C under microwave conditions to cyclize the intermediate and give the triazole scaffold **14** in quantitative yield. Several compounds were synthesized as shown above including the example benzylamide (Scheme 1) and saturated analogues shown in Table 1 via a reductive amination to give **15** followed by deprotection (**16**) and addition of an acrylamide (**17**) as a covalent warhead, or an unreactive isostere of this.

**Table 1:**
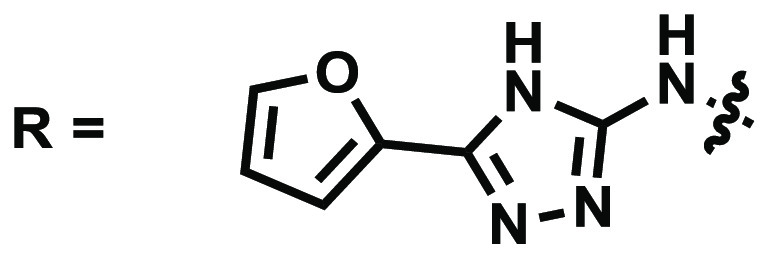

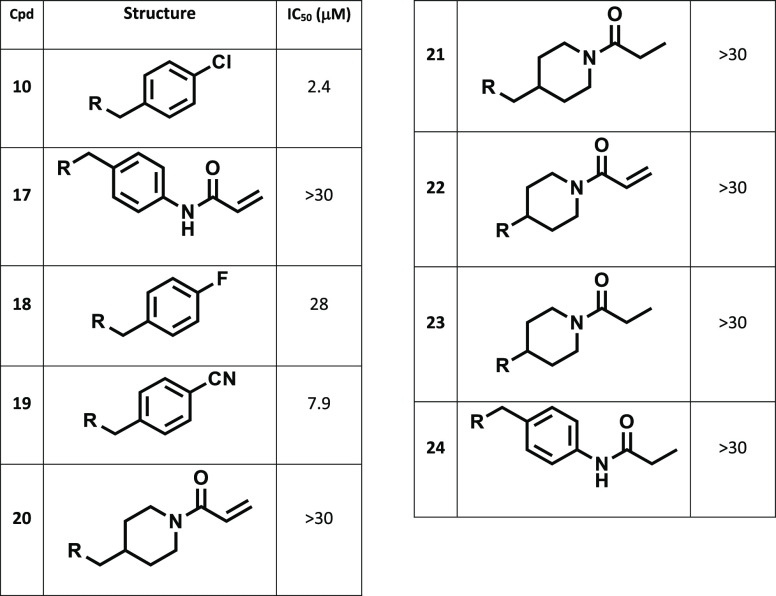
Activity of Compounds against FabA in the Biochemical Assay

The final compounds were designed to hold the covalent warhead in a slightly different orientation relative to the triazole core and to position it in the right area to form a covalent bond to the target cysteine residue - Cys15. These compounds were screened against FabA and the results are shown in Table 1.

The only compounds which showed significant inhibition of FabA were the known FabA inhibitor **10** and the closely related compound **19** where the chlorine atom had been replaced with a nitrile group. To investigate if compound **19** was able to covalently bind to the target, mass spectrometry was used. Formation of a covalent bond would result in an increase in the mass measured for the appropriate peptide, but no MS-fragment corresponding to this compound bound to a peptide was detected, suggesting there was no covalent binding.

The three most potent compounds, **10**, **18**, and **19**, all contained a benzene ring, and it is known from the crystal structure that this benzene occupies space close to the target cysteine residue - Cys15. Attaching a more reactive warhead to a benzene core may allow covalent bond formation. The vinyl sulfonamide warhead was selected as it is more reactive than the acrylamides^15^ previously investigated.

**Scheme 2:**
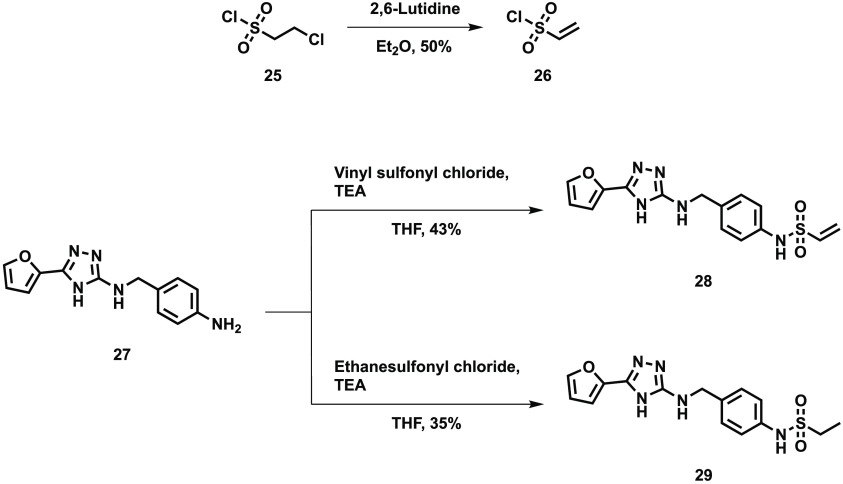
Synthesis of 2-Amino-2-*N*-(4-vinylsulfonylaminobenzyl)-5-furyl-1,3,4-triazole **28** and 2-Amino-2-*N*-(4-ethylsulfonylaminobenzyl)-5-furyl-1,3,4-triazole **29**

Treating 2-chloroethane sulfonyl chloride with a hindered pyridine derivative gave the required vinyl sulfonyl chloride, which was used to synthesize the vinyl sulfonamides **28** and **29** (Scheme 2). Compound **29** was an analogue of the vinyl sulfonamide which could not form a covalent link to the FabA protein, acting as a control.

Compounds **28** and **29** were screened against FabA but were found to be inactive (IC_50_ > 30 μM). This is unlikely to be due to the compound not being reactive enough to form a covalent bond, suggesting that either the target cysteine residue, Cys15, is not reactive or that these types of compounds are not placing the covalent warhead in the correct orientation and/or space to form a covalent bond.

**Figure 5:**
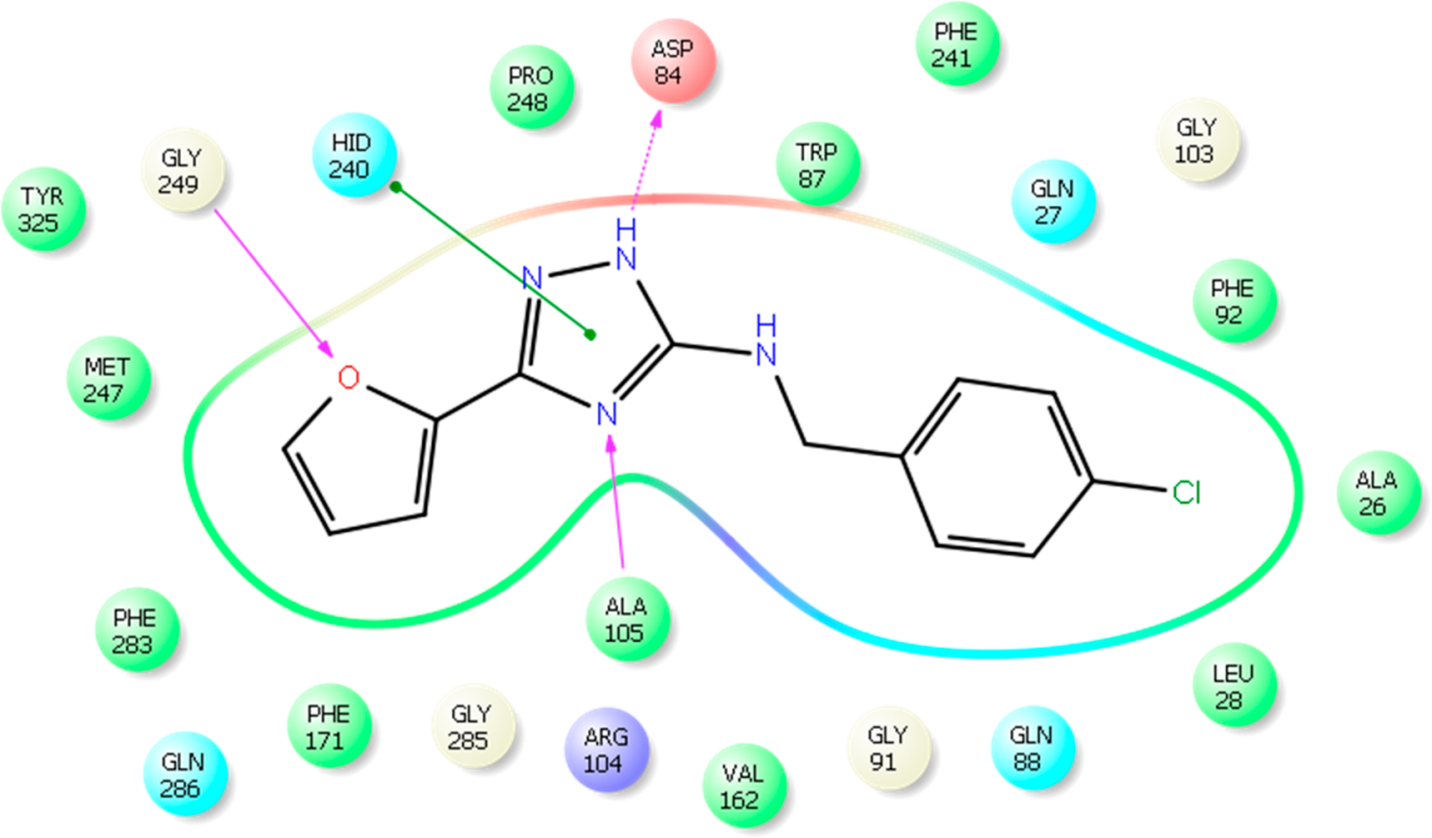
Interactions between known inhibitor and FabA based on the obtained crystal structure (PDB 4CL6).

In *in silico* modeling and docking experiments, it was observed that all predicted interactions between the known inhibitor and the protein, based on the crystal structure, occurred with the triazole ring and the furan ring as shown in Figure 5.

To take advantage of this, it was proposed that developing a set of compounds with flexible linkers of a variety of lengths should allow the covalent warhead to adopt a wide variety of conformations in the binding site. This should better facilitate the formation of a covalent bond. However, these compounds would have a lot of rotational freedom on binding and, therefore, a larger entropic penalty to binding than compounds synthesized thus far. Any of these compounds that seemed to be binding covalently would need to be studied using X-ray crystallography to understand how they bind. Using this information, the linker would be constrained to reduce the entropy while maintaining the ability to form a covalent bond.

## Flexible Linkers to Covalent Warhead

**Scheme 3:**
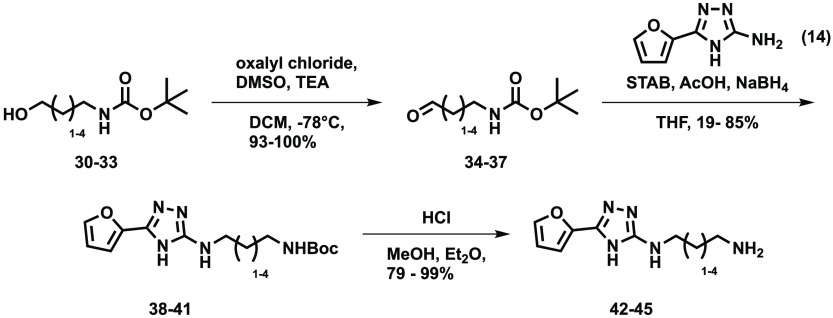
Preparation of a Range of Cores (**42**–**45**)

Previously, directly forming amines on this core via a reductive amination was successful so this method was employed to access these intermediates. The required aldehydes (**34**–**37**) were synthesized (Scheme 3) and used to add appropriate alkyl chains to the triazole core to give intermediates (**38**–**41**) which were subsequently deprotected (**42**–**45**).

**Scheme 4:**
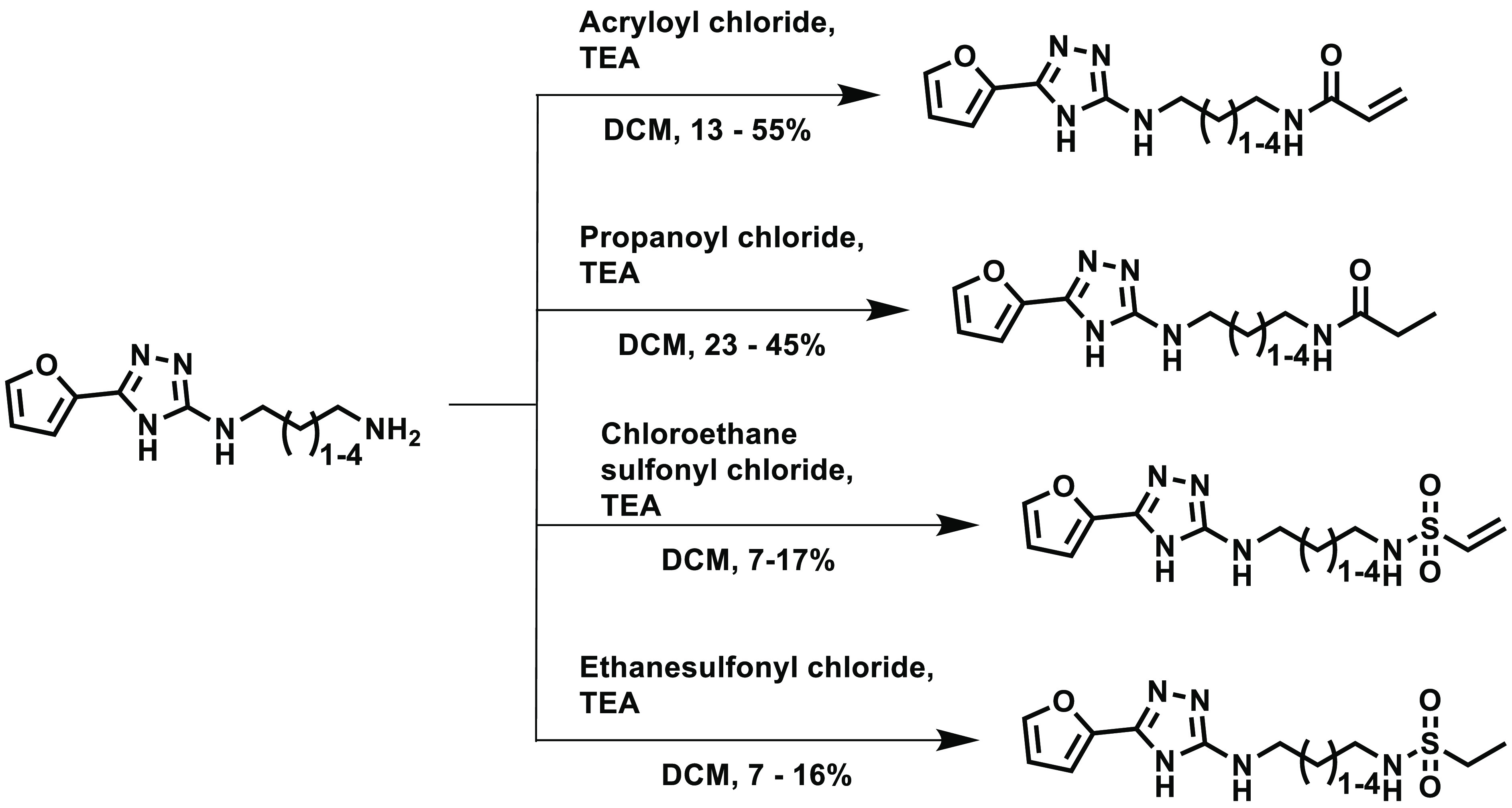
Covalent Warheads and Their Unreactive Isosteres Were Attached to the Free Amines of **42**–**45**

Finally, the covalent warheads (**46**–**49**, **54**–**57**) and an unreactive isostere (**50**–**53**, **58**–**61**) of each were installed on the amine as shown in Scheme 4.

The resulting compound library was screened against FabA; however, in all cases, no significant inhibition of the enzyme was found (IC_50_ > 30 μM). This suggested that it was extremely challenging to covalently inhibit FabA with this scaffold.

## Probing the Binding Site

**Figure 6:**
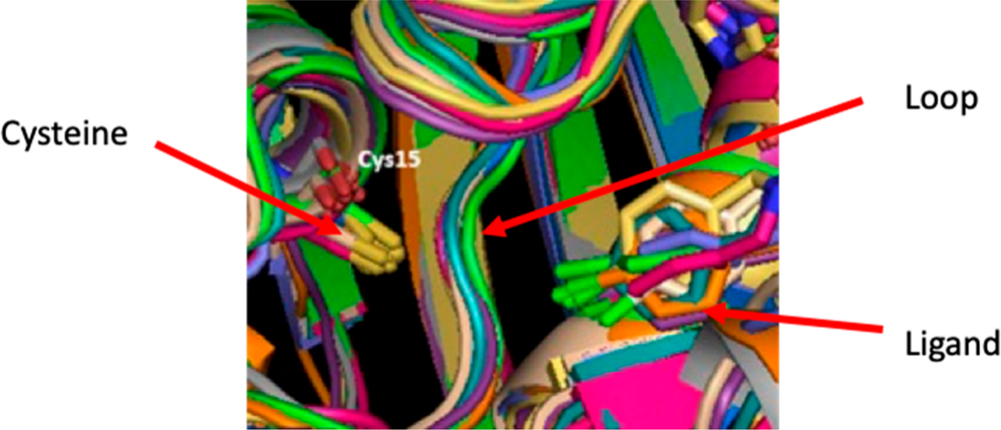
Stacked view of snapshots every 10 ns for 100 ns show this loop remained stationary throughout the simulation.

To investigate why FabA could not be covalently inhibited by compounds based on the triazole scaffold, a molecular dynamics simulation of the protein was performed using the crystal structure of the known inhibitor bound to FabA. This revealed that there is a loop in the active site which sits between the cysteine residue being targeted, Cys15, and the binding site. This loop was observed to not significantly move at any point during the simulation, as shown in Figure 6.

To investigate if this loop was blocking the cysteine residue, a small set of compounds was designed in order to probe the binding site. These were designed to put groups of various sizes and polarity in the active site in approximately the position of the loop and observe any flexibility of the protein.

**Scheme 5:**
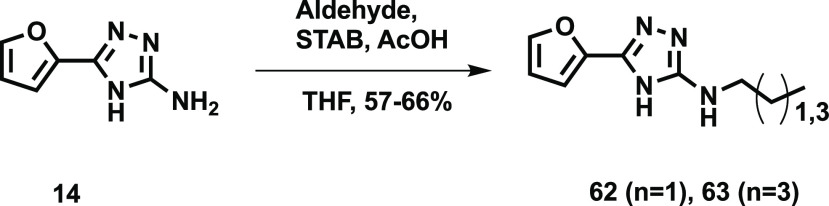
Synthesis of 2-Propylamino-5-furyl-1,3,4-triazole **62** and 2-Pentylamino-5-furyl-1,3,4-triazole **63**

**Scheme 6:**
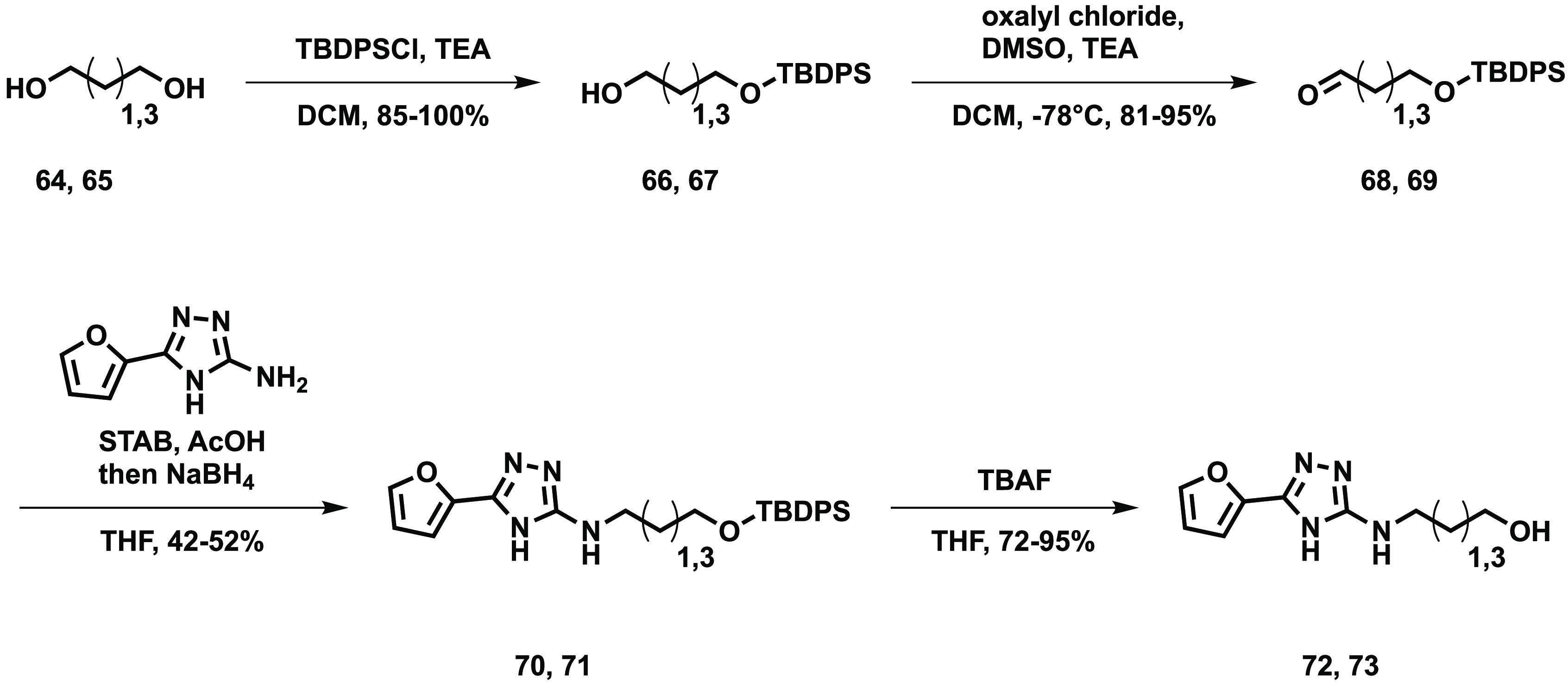
Synthesis of 2-*N*-(*O*-Methyl 4-carboxybutyl)amino-5-furyl-1,3,4-triazole **77** and 2-*N*-(4-carboxybutyl)amino-5-furyl-1,3,4-triazole **79**

Two simple alkane compounds **62** and **63** were synthesized from the corresponding aldehydes (Scheme 5). Two alcohols were also synthesized (Scheme 6).

**Scheme 7:**
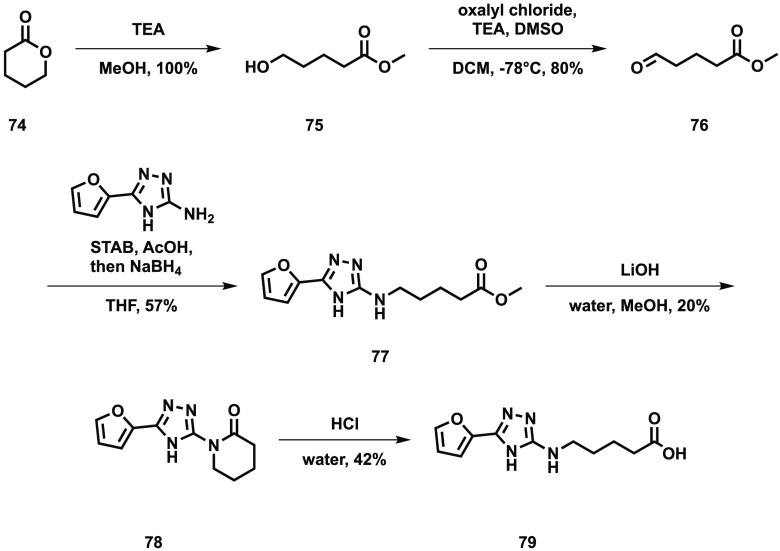
Synthesis of 2-*N*-(*O*-Methyl 4-carboxybutyl)amino-5-furyl-1,3,4-triazole **77** and 2-*N*-(4-carboxybutyl)amino-5-furyl-1,3,4-triazole **79**

The diols were reacted with *tert*-butyl(chloro)diphenylsilane to give the protected alcohols **66** and **67** in high yield. A Swern oxidation was used to convert these to the aldehydes **68** and **69** which were used in reductive aminations with the amino triazole core to give the amines **70** and **71**. The silyloxy protecting group was removed in the final step to obtain the desired alcohols **72** and **73**. Another route was required to synthesize the desired carboxylic acids and esters (Scheme 7).

The lactone **74** was treated with methanol to give the alcohol **75**, and a Swern oxidation was used to convert this to the aldehyde **76**. Performing a reductive amination with this aldehyde and the amino triazole core gave the required ester **77**. However, attempting to hydrolyze this ester to the carboxylic acid **79** using lithium hydroxide instead gave the lactam **78**. Subsequently this was converted to the desired straight chain carboxylic acid **79** by treating with aqueous acid.

**Scheme 8:**
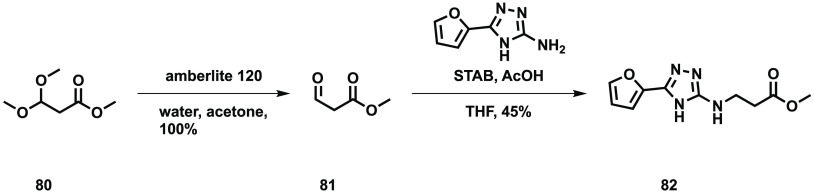
Synthesis of 2-*N*-(*O*-Methyl 2-carboxyethyl)amino-5-furyl-1,3,4-triazole **82**

For the shorter chain ester, the acetal was converted to the aldehyde using Amberlite acidic resin, and the reductive amination of the amino triazole core with the aldehyde gave the desired ester (Scheme 8).

The resulting library of potential inhibitors was screened against FabA (Table 2).

**Table 2:**
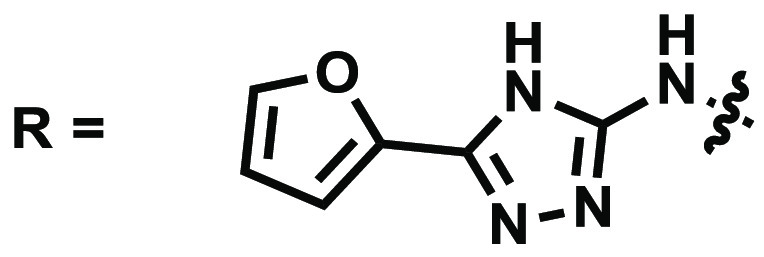

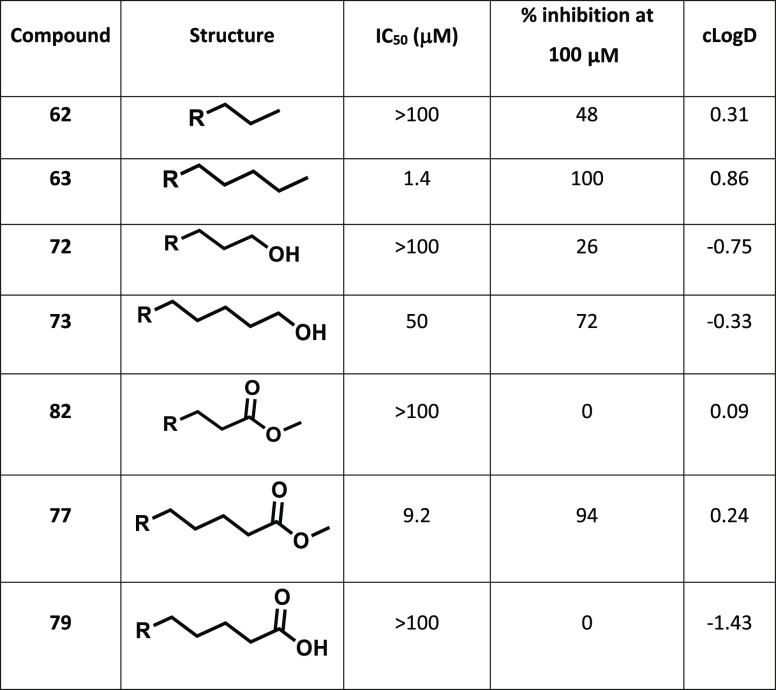
Final Library of Compounds Was Screened against FabA^a^

a Where a compound was not potent enough to measure a pIC_50_, the percent inhibition at 100 μM is reported.

A longer alkyl chain in the molecule gave better potency, e.g., **63** compared to **62**. Adding a polar group to this resulted in a decrease in the potency, e.g., **73** vs **63**. The log *D* was calculated in Stardrop and found not to be correlated well with the potency meaning, this is not just related to the lipophilicity. Ultimately, these data suggest that these compounds are binding in a hydrophobic pocket. A covalent warhead has heteroatoms which induce polarity, and even if the loop sitting in the binding site in front of the target cysteine residue, Cys15, can move it seems unlikely that a polar covalent warhead will be tolerated in this pocket. These data, along with the previous sets of data, suggest that identifying a covalent inhibitor of FabA is extremely challenging. Therefore, attempts to develop a covalent inhibitor of FabA by attaching a covalent warhead to the known inhibitor were abandoned.

## Experimental Section

### FabA RapidFire High-Throughput Mass Spectrometry Assay

**Figure 7:**
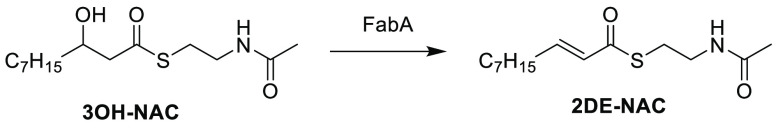
FabA RapidFire assay uses HTMS to measure the conversion of 3-OH decanoyl-*N*-acetylcysteamine **3OH-NAC** to 2-decenoyl-*N*-acetylcysteamine **2DE-NAC**. PaFabA was produced as previously described.^13^

Details of FabA RapidFire high-throughput mass spectrometry (HTMS) assay development will be published separately. An overview is shown in Figure 7. Briefly, compounds in DMSO stock were dispensed into 384-well assay plate (Greiner 781101) through an ECHO 550 acoustic liquid-handling system (LabCyte). Assays were performed by adding 5 μL 40 nM FabA protein solution in reaction buffer (50 mM Tris, pH 7.5, 1 mM DTT, 0.1% BSA, 0.005% NP40), and the reaction was initiated with the addition of a 5 μL 720 μM substrate 3-OH decanoyl-*N*-acetylcysteamine (3OH-NAC) in assay buffer. The plates were incubated in the plate shaker at 300 rpm at room temperature for 30 min, followed by addition of 90 μL of 1% formic acid to quench enzyme reaction. The reaction mixture was subjected to RapidFire HTMS analysis.

RapidFire HTMS was performed using a RapidFire 365 system (Agilent) coupled to a triple quadrupole mass spectrometer 6470 (Agilent). The samples were loaded onto a C4 cartridge (Agilent) using deionized water containing 5 mM ammonium formate at flow rate of 1.5 mL/min and eluted to the mass spectrometer using acetonitrile/deionized water (90/10, v/v) containing 5 mM ammonium formate in at a flow rate of 1.25 mL/min. The sipper was washed to minimize carryover with deionized water followed by acetonitrile. Aspiration time, load/wash time, elution time and re-equilibration time were set to 600, 3000, 5000, and 500 ms, respectively, with a cycle time of approximately 10 s. The triple-quadrupole mass spectrometer with electro-spray ion source was operated in positive multiple reaction monitoring (MRM) mode. The detailed setting for the mass spectrometer parameters was as follows: capillary voltage: 3000 V; gas temperature: 350 °C; gas flow: 7 l/min; nebulizer: 40 psi; sheath gas temperature 300 °C; sheath gas flow: 11 l/min; and nozzle voltage 1500 V. The MRM transitions (Q1 and Q3) for 2-decenoyl-N-acetylcysteamine (**2DE-NAC**), as a reaction product, were set as 272.1/153.1. The mass resolution window for both parental and daughter ions was set at as unit (0.7 Da). The dwell time, fragmentor, and collision energy for each transition were 50 ms, 100 V and 8 eV, respectively.

The inhibitory activity was calculated using the reaction product peak area. The peak area of the reaction product without enzyme was defined as 100% inhibitory activity, whereas that of the complete reaction mixture as 0% inhibitory activity. Curve fittings and calculations of IC_50_ values were performed using ActivityBase XE version 9.2.0.106 from IDBS with a four-parameter logistics model.

### General Methods

Chemicals and solvents were purchased from commercial sources and were used without any further purification unless noted otherwise. Air and water sensitive reactions were carried out under an inert nitrogen atmosphere in oven-dried glassware. Analytical thin-layer chromatography (TLC) was performed on precoated TLC plates (layer 0.20 mm silica gel 60 with fluorescent indicator UV254, from Merck). Developed plates were air-dried and analyzed under a UV lamp (UV254/365 nm) and by staining with permanganate or ninhydrin. Flash column chromatography was performed on prepacked silica gel cartridges (230–400 mesh, 40–63 μm, from SiliCycle) using a Teledyne ISCO Combiflash Rf or Combiflash Rf 200i. ^1^H (500 MHz), ^13^C (125 MHz), ^1^H (400 MHz), ^13^C (100 MHz), and 2D NMR spectra were recorded in DMSO-*d*_6_, MeOD-*d*_4_, or CDCl_3_ using a Bruker Avance spectrometer. Proton chemical shifts are reported in ppm relative to the residual DMSO peak (δ = 2.50 ppm), methanol peak (δ = 3.31 ppm), or chloroform peak (δ = 7.26 ppm). Multiplicities are given as s (singlet), d (doublet), t (triplet), q (quartet), sept (septet), m (multiplet), brs (broad singlet), dd (doublet of doublets), or as a combination of these. Coupling constants (*J*) are quoted to the nearest 0.1 Hz. ^13^C chemical shifts are reported in ppm relative to the residual DMSO peak (δ = 39.51 ppm), methanol peak (δ = 49.00 ppm), or chloroform peak (δ = 77.16 ppm). High-resolution electrospray measurements were performed on a Bruker Daltonics MicrOTOF mass spectrometer.

#### 1-(Furan-2-carbonylamino)guanidine **13**

Furan-2-carbohydrazide (5.0 g, 39.64 mmol, 1 equiv) and *o*-methylisourea bisulfate (20.5 g, 119 mmol, 5 equiv) were added to a solution of sodium hydroxide (7.93 g, 198 mmol, 8 equiv) in water (100 mL) and the reaction was stirred at room temperature. After 6 h the resultant precipitate was filtered, washed with water, diethyl ether and dried to give 1-(furan-2-carbonylamino)guanidine (5.99 g, 90%) as a gray powder. ^1^H (500 MHz, DMSO-*d*_6_): δ 10.81 (1H, brs), 7.54 (1H, q, *J* = 0.8 Hz), 6.91 (2H, brs), 6.76 (2H, brs), 6.63 (1H, d, *J* = 2.9 Hz), 6.44 (1H, dd, *J* = 3.2, 1.8 Hz); ^13^C (125 MHz, DMSO-*d*_6_): δ 155.19, 152.78, 141.87, 110.80, 108.28; MS (ESI): *m*/*z* = 169.1 [M + H]^+^.

#### 2-Amino-5-furyl-1,3,4-triazole **14**

1-(Furan-2-carbonylamino)guanidine **13** (5.78 g, 34.39 mmol, 1 equiv) was suspended in water (15 mL) and heated in the microwave to 140 °C, 6 bar, for 1 h. The reaction was evaporated to dryness to give 2-amino-5-furyl-1,3,4-triazole (5.1 g, 99%) as an off-white powder. ^1^H (500 MHz, DMSO-*d*_6_): δ 12.11 (1H, brs), 7.68 (1H, s), 6.69 (1H, s), 6.54 (1H, s), 6.05 (2H, brs); ^13^C (125 MHz, DMSO-*d*_6_): δ 142.74, 111.32, 107.63; MS (ESI): *m*/*z* = 151.1 [M + H]^+^. Analysis is in agreement with the literature.^16^

#### 2-Amino-2-*N*-(4-(Boc)aminobenzyl)-5-furyl-1,3,4-triazole **15**

2-Amino-5-furyl-1,3,4-triazole **14** (400 mg, 2.66 mmol, 1 equiv), *tert*-butyl *N*-(4-formylphenyl)carbamate (884 mg, 4.00 mmol, 1.5 equiv) and sodium triacetoxyborohydride (1130 mg, 5.33 mmol, 2 equiv) were dissolved in THF (25 mL) under nitrogen and acetic acid (305 μL, 5.33 mmol, 2 equiv) was added. The reaction was stirred overnight at room temperature. LCMS indicated the reaction stalled at 50% conversion, so a second portion of sodium triacetoxyborohydride (1130 mg, 5.33 mmol, 2 equiv) and acetic acid (305 μL, 5.33 mmol, 2 equiv) were added, and the reaction stirred overnight at room temperature. LCMS indicated no further conversion occurred, so 10 mL saturated sodium hydrogen carbonate solution was added, and the mixture stirred vigorously, the layers were separated, and the aqueous layer extracted 2x with 20 mL ethyl acetate. The combined organic layers were dried over MgSO_4_, passed through a phase separator and evaporated to dryness. The residue was purified by flash chromatography (0–10% MeOH in DCM) to give 2-amino-2-*N*-(4-(Boc)aminobenzyl)-5-furyl-1,3,4-triazole (307 mg, 32%) as a white powder. ^1^H (500 MHz, MeOD-*d*_4_): δ 7.59 (1H, d, *J* = 1.1 Hz), 7.36 (2H, d, *J* = 8.5 Hz), 7.28 (2H, d, *J* = 8.7 Hz), 6.90 (1H, d, *J* = 3.4 Hz), 6.54 (1H, dd, *J* = 3.4, 1.8 Hz), 4.41 (2H, s), 1.51 (9H, s); MS (ESI): *m*/*z* = 356.2 [M + H]^+^.

#### 2-Amino-2-*N*-(4-aminobenzyl)-5-furyl-1,3,4-triazole **16**

2-Amino-2-*N*-(4-(Boc)aminobenzyl)-5-furyl-1,3,4-triazole **15** (307 mg, 0.86 mmol, 1 equiv) was dissolved in DCM (20 mL), trifluoroacetic acid (662 μL, 8.64 mmol, 10 equiv) was added, and the reaction was stirred at room temperature. After 2 h, LCMS indicated no starting material remained, so the reaction was evaporated to dryness. The residue was dissolved in 10 mL saturated sodium hydrogen carbonate solution and extracted 3× with 20 mL ethyl acetate. The combined organics were dried over MgSO_4_, passed through a phase separator and evaporated to dryness. The residue was purified by flash chromatography (0–10% MeOH in DCM) to give 2-amino-2-*N*-(4-aminobenzyl)-5-furyl-1,3,4-triazole (105 mg, 48%) as a white solid. ^1^H (500 MHz, MeOD-*d*_4_): δ 7.59 (1H, s), 7.12 (2H, d, *J* = 8.5 Hz), 6.89 (1H, s), 6.71 (2H, dt, *J* = 8.7, 2.3 Hz), 6.54 (1H, s), 6.54 (1H, s), 4.32 (2H, s). HRMS *m*/*z* (ESI^+^) calcd for C_13_H_14_N_5_O [M + H]^+^: 256.1193, found 256.1205 (4.6 ppm).

#### 2-Amino-2-*N*-(4-acrylamidobenzyl)-5-furyl-1,3,4-triazole **17**

2-Amino-2-*N*-(4-aminobenzyl)-5-furyl-1,3,4-triazole **16** (10 mg, 0.039 mmol, 1 equiv) was dissolved in THF (5 mL), and triethylamine (11 μL, 0.078 mmol, 2 equiv) was added. The reaction was cooled to 0 °C and prop-2-enoyl chloride (3.2 μL, 0.039 mmol, 1 equiv) was added. After 5 min, the reaction was quenched by the addition of 5 mL methanol and the solvent removed *in vacuo*. The residue was purified by HPLC (5–95% acetonitrile in water (0.1% formic acid)) to give 2-amino-2-*N*-(4-acrylamidobenzyl)-5-furyl-1,3,4-triazole (6 mg, 50%) as a white powder. ^1^H (500 MHz, DMSO-*d*_6_): δ 12.37 (1H, brs), 10.11 (1H, s), 7.71 (1H, s), 7.61 (2H, d, *J* = 7.5 Hz), 7.30 (2H, d, *J* = 8.3 Hz), 6.74 (1H,s), 6.56 (1H, s), 6.42 (1H, dd, *J* = 16.9, 10.1 Hz), 6.24 (1H, d, *J* = 17.0 Hz), 5.73 (1H, d, *J* = 10.1 Hz), 4.33 (2H, s); ^13^C (125 MHz, DMSO-*d*_6_): δ 162.99, 143.0, 137.74, 131.88, 127.63, 126.70, 119.20, 111.39, 45.84; HRMS *m*/*z* (ESI^+^) calcd for C_16_H_16_N_5_O_2_ [M + H]^+^: 310.1299, found 310.1308 (2.9 ppm).

#### 2-(4-Chlorobenzyl)amino-5-furyl-1,3,4-triazole **10**

2-Amino-5-furyl-1,3,4-triazole **14** (200 mg, 1.33 mmol, 1 equiv) and 4-chlorobenzaldehyde (281 mg, 2.00 mmol, 1.5 equiv) were dissolved in dry methanol (15 mL) under nitrogen. Acetic acid (0.15 mL, 2.66 mmol, 2 equiv) was added, and the reaction stirred at room temperature. After 20 min, sodium cyanoborohydride (167 mg, 2.66 mmol, 2 equiv) was added and the reaction stirred overnight.

The reaction was evaporated to dryness and the residue dissolved in 20 mL ethyl acetate, filtered through Celite, and washed with 10 mL water. The aqueous layer was extracted 2× with 10 mL ethyl acetate and the combined organic layers washed with 15 mL saturated sodium hydrogen carbonate solution, dried over MgSO_4_, passed through a phase separator, and evaporated to dryness. The residue was purified by flash chromatography (0–8% MeOH in DCM) to give 2-(4-chlorobenzyl)amino-5-furyl-1,3,4-triazole (72.6 mg, 19%) as a white powder. ^1^H (400 MHz, MeOD-*d*_4_): δ 7.61 (1H, s), 7.40–7.32 (4H, m), 6.92 (1H, d, *J* = 3.2 Hz), 6.56 (1H, dd, *J* = 3.3, 1.8 Hz), 4.49 (2H, s); ^13^C (100 MHz, MeOD-*d*_4_): δ 145.83, 131.96, 129.87, 129.56, 112.46, 54.79, 49.85, 47.16; HRMS *m*/*z* (ESI^+^) calcd for C_13_H_12_N_4_OCl [M + H]^+^: 275.0694, found 275.0711 (6.2 ppm).

#### 2-(4-Fluorobenzyl)amino-5-furyl-1,3,4-triazole **18**

2-Amino-5-furyl-1,3,4-triazole **14** (100 mg, 0.67 mmol, 1 equiv) was dissolved in methanol (15 mL) at room temperature, and 4-fluorobenzaldehyde (165 mg, 1.33 mmol, 2 equiv) and 3 Å molecular sieves were added. The mixture was heated to reflux and stirred gently overnight. TLC confirmed complete conversion of the starting material, so the reaction was cooled to 0 °C, and sodium cyanoborohydride (84 mg, 1.33 mmol, 2 equiv) was added and allowed to slowly warm to room temperature overnight. The mixture was evaporated to dryness, dissolved in ethyl acetate and filtered through Celite, washed with saturated sodium hydrogen carbonate solution, brine, dried over MgSO_4_, and evaporated to dryness. The residue was purified by flash chromatography (2–10% MeOH in DCM) to yield 2-(4-fluorobenzyl)amino-5-furyl-1,3,4-triazole (25.2 mg, 14%) as a white powder. ^1^H (400 MHz, MeOD-*d*_4_): δ 7.59 (1H, s), 7.38 (2H, dd, *J* = 8.6, 5.4 Hz), 7.05 (2H, t, *J* = 8.8 Hz), 6.90 (1H, s), 6.54 (1H, s), 4.46 (2H, s); HRMS *m*/*z* (ESI^+^) calcd for C_13_H_12_N_4_OF [M + H]^+^: 259.0990, found 259.0984 (2.4 ppm).

#### 2-(4-Cyanobenzyl)amino-5-furyl-1,3,4-triazole **19**

2-Amino-5-furyl-1,3,4-triazole **14** (100 mg, 0.67 mmol, 1 equiv) and 4-formylbenzonitrile (131 mg, 1.00 mmol, 1.5 equiv) were dissolved in dry methanol (10 mL) under nitrogen. Acetic acid (76 μL, 1.33 mmol, 2 equiv) was added and the reaction stirred at room temperature. After 2 h, sodium cyanoborohydride (84 mg, 1.33 mmol, 2 equiv) was added, the reaction stirred overnight. LCMS indicated incomplete conversion, so a second portion of sodium cyanoborohydride (84 mg, 1.33 mmol, 2 equiv) was added, and the reaction stirred for 3 h then evaporated to dryness. The residue dissolved in 20 mL ethyl acetate, filtered through Celite and washed with 10 mL water. The aqueous layer was extracted 2× with 10 mL ethyl acetate, and the combined organic layers washed with 15 mL saturated sodium hydrogen carbonate solution, dried over MgSO_4_, passed through a phase separator, and evaporated to dryness. The residue was purified by flash chromatography (3–8% MeOH in DCM) to yield 2-(4-cyanobenzyl)amino-5-furyl-1,3,4-triazole (32 mg, 16%) as a white powder. ^1^H (400 MHz, MeOD-*d*_4_): δ 7.69 (4H, m), 7.60 (1H, d, *J* = 1.0 Hz), 7.53 (4H, m), 6.90 (1H, d, *J* = 3.3 Hz), 6.54 (1H, dd, *J* = 3.4, 1.8 Hz), 4.58 (2H, s); ^13^C (100 MHz, MeOD-*d*_4_): δ 148.93, 144.69, 133.39, 133.25, 129.00, 128.28, 112.51, 110.43, 47.37; HRMS *m*/*z* (ESI^+^) calcd for C_14_H_11_N_5_O [M + H]^+^: 266.1036, found 266.1034 (1.1 ppm). Analysis is in agreement with the literature.^17^

#### 2-Amino-2-*N*-(1-(Boc)piperidin-4-ylmethyl)-5-furyl-1,3,4-triazole **83**

2-Amino-5-furyl-1,3,4-triazole **14** (100 mg, 0.67 mmol, 1 equiv) and *tert*-butyl 4-formylpiperidine-1-carboxylate (213 mg, 0.1 mmol, 1.5 equiv) were dissolved in dry methanol (10 mL) under nitrogen. Acetic acid (76 μL, 1.3 mmol, 2 equiv) was added and the reaction stirred at room temperature. After 2 h, sodium cyanoborohydride (83.7 mg, 1.3 mmol, 2 equiv) was added, and the reaction stirred overnight. LCMS indicated incomplete conversion so a second portion of sodium cyanoborohydride (83.7 mg, 1.3 mmol, 2 equiv) and the reaction stirred for 3 h then evaporated to dryness. The residue dissolved in 20 mL ethyl acetate, filtered through Celite and washed with 10 mL water. The aqueous layer was extracted 2× with 10 mL ethyl acetate, and the combined organic layers were washed with 15 mL saturated sodium hydrogen carbonate solution, dried over MgSO_4_, passed through a phase separator, and evaporated to dryness. The residue was purified by flash chromatography (0–8% MeOH in DCM) to give 2-amino-2-*N*-(1-(Boc)piperidin-4-ylmethyl)-5-furyl-1,3,4-triazole (70.4 mg, 30%) as a white powder. ^1^H (400 MHz, MeOD-*d*_4_): δ 7.59 (1H, d, *J* = 1.0 Hz), 6.89 (1H, d, *J* = 3.2 Hz), 6.84 (1H, dd, *J* = 3.3, 1.8 Hz), 4.08 (2H, d, *J* = 13.2 Hz), 3.16 (2H, d, *J* = 6.5 Hz), 2.74 (2H, s), 1.86–1.73 (3H, m), 1.45 (9H, s), 1.12 (2H, qd, *J* = 12.7, 3.7 Hz); ^13^C (100 MHz, MeOD-*d*_4_): δ 156.48, 144.45, 112.42, 110.11, 80.91, 49.67, 45.13, 37.49, 30.78, 28.70; MS (ESI): *m*/*z* = 348.2 [M + H]^+^.

#### 2-Amino-2-*N*-(1-propanoylpiperidin-4-ylmethyl)-5-furyl-1,3,4-triazole **21**

2-Amino-2-*N*-(1-(Boc)piperidin-4-ylmethyl)-5-furyl-1,3,4-triazole **83** (61 mg, 0.18 mmol, 1 equiv) was dissolved in DCM (5 mL), TFA (202 μL, 2.63 mmol, 15 equiv) was added, and the reaction was stirred for 5 h. The residue was dissolved in DCM (5 mL), and triethylamine (73 μL, 0.53 mmol, 3 equiv) and propanoyl chloride (17 μL, 0.19 mmol, 1.1 equiv) were added. After 30 min, water (5 mL) was added, the layers separated, and the aqueous layer extracted 2× with 5 mL DCM, the combined organics were dried over MgSO_4_ and evaporated to dryness. The residue was purified by flash chromatography (0–10% MeOH in DCM) to give 2-amino-2-*N*-(1-propanoylpiperidin-4-ylmethyl)-5-furyl-1,3,4-triazole (17 mg, 26%) as a white powder. ^1^H (500 MHz, DMSO-*d*_6_): δ 7.80 (1H, d, *J* = 1.0 Hz), 6.91 (1H, d, *J* = 3.3 Hz), 6.62 (1H, dd, *J* = 3.4, 1.8 Hz), 4.38 (1H, d, *J* = 12.8 Hz), 3.85 (1H, d, *J* = 13.3 Hz), 3.11 (2H, d, *J* = 6.8 Hz), 2.95 (1H, t, *J* = 12.3 Hz), 2.50 (1H, m), 2.29 (2H, q, *J* = 7.4 Hz), 1.77 (3H, m), 1.09 (1H, m), 0.98 (4H, t, *J* = 7.4 Hz); ^13^C (125 MHz, DMSO-*d*_6_): δ 170.97, 143.98, 111.66, 109.66, 48.11, 44.56, 40.82, 36.63, 29.81, 29.03, 25.56, 9.46; MS (ESI): *m*/*z* = 304.2 [M + H]^+^.

#### t*ert*-Butyl 4-Oxopiperidine-1-carboxylate **84**

Piperidin-4-one (500 mg, 5.04 mmol, 1 equiv) and di-*tert*-butyl dicarbonate (2202 mg, 10.09 mmol, 2 equiv) were dissolved in methanol (20 mL). Triethylamine (1.05 mL, 7.57 mmol, 1.5 equiv) was added and the reaction stirred overnight at room temperature. The reaction was evaporated to dryness, the residue partitioned between ethyl acetate and saturated sodium hydrogen carbonate solution, the layers separated, and the aqueous layer extracted 2× with ethyl acetate. The combined organic layers were dried over MgSO_4_ and evaporated to dryness. The residue was purified by flash chromatography (0–5% MeOH in DCM) to give *tert*-butyl 4-oxopiperidine-1-carboxylate (318 mg, 32%) as a white solid. ^1^H (400 MHz, CDCl_3_): δ 3.64 (4H, t, *J* = 6.2 Hz), 2.36 (4H, t, *J* = 6.2 Hz), 1.41 (9H, s); ^13^C (100 MHz, CDCl_3_): δ 207.64, 154.43, 80.34, 41.12, 28.42. Analysis is in agreement with the literature.^18^

#### 2-Amino-2-*N*-(1-(Boc)piperidin-4-yl)-5-furyl-1,3,4-triazole **85**

2-Amino-5-furyl-1,3,4-triazole **14** (350 mg, 2.33 mmol, 1 equiv), *tert*-butyl 4-oxopiperidine-1-carboxylate **84** (697 mg, 3.50 mmol, 1.5 equiv) and sodium triacetoxyborohydride (988 mg, 4.66 mmol, 2 equiv) were dissolved in THF (25 mL) under nitrogen, and acetic acid (267 μL, 4.66 mmol, 2 equiv) was added. The reaction was stirred at room temperature overnight.

LCMS indicated the reaction was incomplete, so a second portion of sodium triacetoxyborohydride (988 mg, 4.66 mmol, 2 equiv) and acetic acid (267 μL, 4.66 mmol, 2 equiv) were added, and the reaction stirred overnight at room temperature. LCMS indicated no further conversion occurred, so 10 mL saturated sodium hydrogen carbonate solution was added, and the mixture stirred vigorously, the layers were separated, and the aqueous layer extracted 2× with 20 mL ethyl acetate. The combined organic layers were dried over MgSO_4_, passed through a phase separator, and evaporated to dryness. The residue was purified by flash chromatography (0–10% MeOH in DCM) to give 2-amino-2-*N*-(1-(Boc)piperidin-4-yl)-5-furyl-1,3,4-triazole (611 mg, 79%) as a white powder. ^1^H (500 MHz, MeOD-*d*_4_): δ 7.60 (1H, s), 6.89 (1H, s), 6.54 (1H, s), 4.05 (2H, d, *J* = 13.5 Hz), 3.76 (2H, m, H11b), 3.12–2.85 (2H, m), 2.04–1.96 (2H, m), 1.45 (9H, s), 1.44–1.35 (2H, m). MS (ESI): *m*/*z* = 334.2 [M + H]^+^.

#### 2-Amino-2-*N*-(1-acrylpiperidin-4-yl)-5-furyl-1,3,4-triazole **22**

2-Amino-2-*N*-(1-(Boc)piperidin-4-yl)-5-furyl-1,3,4-triazole **85** (17 mg, 0.052 mmol, 1 equiv) was dissolved in DCM (5 mL), TFA (60 μL, 0.78 mmol, 15 equiv) was added, and the reaction stirred at room temperature for 3 h when TLC showed no starting material remained. The reaction was evaporated to dryness, the residue dissolved in DCM (5 mL), and triethylamine (22 μL, 0.16 mmol, 3 equiv) and prop-2-enoyl chloride (4.6 μL, 0.057 mmol, 1.1 equiv) were added. The reaction was stirred at room temperature for 3 h when TLC indicated no intermediate remained. Water (3 mL) was added, the layers separated, and the aqueous layer extracted 2× with DCM, the combined organics were dried over MgSO_4_, and evaporated *in vacuo*. The residue was purified by flash chromatography (0–5% MeOH in DCM) to give 2-amino-2-*N*-(1-acrylpiperidin-4-yl)-5-furyl-1,3,4-triazole (13 mg, 83%) yield as a white powder. ^1^H (400 MHz, CDCl_3_): δ 7.50 (1H, dd, *J* = 1.8, 0.8 Hz), 6.93 (1H, dd, *J* = 3.4, 0.6 Hz), 6.59 (1H, dd, *J* = 16.8, 10.6 Hz), 6.52 (1H, dd, *J* = 3.4, 1.8 Hz), 6.27 (1H, dd, *J* = 16.8, 1.9 Hz), 5.69 (1H, dd, *J* = 10.6, 1.9 Hz), 4.47 (2H, m), 3.91 (2H, m), 2.96 (1H, m), 2.16 (2H, m), 1.44 (2H, m); MS (ESI): *m*/*z* = 288.2 [M + H]^+^.

#### 2-Amino-2-*N*-(1-propanoylpiperidin-4-yl)-5-furyl-1,3,4-triazole **23**

2-Amino-2-*N*-(1-(Boc)piperidin-4-yl)-5-furyl-1,3,4-triazole **85** (70 mg, 0.21 mmol, 1 equiv) was dissolved in DCM (5 mL) and TFA (241 μL, 3.15 mmol, 15 equiv) added. The reaction was stirred for 3 h and evaporated to dryness. The residue was dissolved in DCM (5 mL), and triethylamine (88 μL, 0.63 mmol, 3 equiv) and propanoyl chloride (18 μL, 0.21 mmol, 1 equiv) were added. The reaction was stirred for 3 h and evaporated to dryness. The residue was dissolved in 5 mL DCM and 3 mL water, the layers separated, and the aqueous layer extracted 2× with 5 mL DCM, the combined organics were dried over MgSO_4_ and evaporated to dryness. The residue was purified by flash chromatography eluting. The residue was purified by flash chromatography (0–8% MeOH in DCM) to give 2-amino-2-*N*-(1-propanoylpiperidin-4-yl)-5-furyl-1,3,4-triazole (59 mg, 73%) yield as a yellow solid. ^1^H (500 MHz, DMSO-*d*_6_): δ 7.76 (1H, d, *J* = 1.0 Hz), 6.83 (1H, d, *J* = 3.3 Hz), 6.59 (1H, dd, *J* = 3.3, 1.8 Hz), 4.27 (1H, d, *J* = 13.0 Hz), 3.83 (1H, d, *J* = 13.5 Hz), 3.61 (1H, m), 3.10 (1H, m), 2.74 (1H, t, *J* = 11.5 Hz), 2.32 (2H, q, *J* = 7.4 Hz), 1.92 (2H, dd, *J* = 23.0, 12.9 Hz), 1.38 (1H, q, *J* = 10.1 Hz), 1.29 (1H, q, *J* = 10.1 Hz), 0.99 (3H, t, *J* = 7.4 Hz); ^13^C (125 MHz, DMSO-*d*_6_): δ 171.06, 143.46, 111.50, 108.84, 49.90, 43.44, 40.43, 32.21, 31.43, 25.48, 9.44; MS (ESI): *m*/*z* = 290.1 [M + H]^+^.

#### 2-Amino-2-*N*-(4-propanoylamidobenzyl)-5-furyl-1,3,4-triazole **24**

2-Amino-2-*N*-(4-aminobenzyl)-5-furyl-1,3,4-triazole **27** (10 mg, 0.039 mmol, 1 equiv) was dissolved in THF (5 mL), and triethylamine (11 μL, 0.078 mmol, 2 equiv) was added. The reaction was cooled to 0 °C, and propanoyl chloride (3.4 μL, 0.039 mmol, 1 equiv) was added. After 5 min, the reaction was quenched by the addition of 5 mL methanol and the solvent removed *in vacuo*. The residue was purified by HPLC (5–95% acetonitrile in water (0.1% formic acid)) to give 2-amino-2-*N*-(4-propanoylamidobenzyl)-5-furyl-1,3,4-triazole (12 mg, 93%) as a white powder. ^1^H (500 MHz, DMSO-*d*_6_): δ 12.28 (1H, brs), 9.80 (1H, brs), 7.68 (1H, s), 7.52 (2H, d, *J* = 8.1 Hz), 7.25 (2H, d, *J* = 8.4 Hz), 7.11 (1H, brs), 6.70 (1H, brs), 6.54 (1H, brs), 4.31 (2H, d, *J* = 5.7 Hz), 2.29 (2H, q, *J* = 7.6 Hz), 1.06 (3H, t, *J* = 7.6 Hz); ^13^C (125 MHz, DMSO-*d*_6_): δ 171.91, 142.81, 127.63, 118.97, 111.38, 107.59, 45.92, 29.54, 9.76. HRMS *m*/*z* (ESI^+^) calcd for C_16_H_18_N_5_O_2_ [M + H]^+^: 312.1455, found 312.1459 (1.3 ppm).

#### Vinyl Sulfonyl Chloride **26**

2-Chloroethanesulfonyl chloride (**25**) (500 mg, 3.07 mmol, 1 equiv) was dissolved in ether (5 mL) and cooled to −78 °C. 2,6-Dimethylpyridine (0.43 mL, 3.68 mmol, 1.2 equiv) in ether (2 mL) was added dropwise over 10 min, and the reaction was stirred for 45 min then allowed to warm to room temperature and stirred overnight. The reaction was cooled to 0 °C, and 5 mL 1% H_2_SO_4_ solution was added. The organic layer was separated, and the aqueous layer extracted 2× with 5 mL ether, dried over MgSO_4_, passed through a phase separator, and evaporated to dryness. The residue was purified by distillation using a kugelrohr apparatus (65 °C, 25 mbar) to give vinyl sulfonyl chloride (196.2 mg, 50%) as a colorless oil. ^1^H (400 MHz, DMSO-*d*_6_): δ 6.47–6.37 (1H, m), 5.63–5.55 (1H, m), 5.31–5.23 (1H, m).

#### 2-Amino-2-*N*-(4-vinylsulfonylaminobenzyl)-5-furyl-1,3,4-triazole **28**

2-Amino-2-*N*-(4-aminobenzyl)-5-furyl-1,3,4-triazole **27** (10 mg, 0.039 mmol, 1 equiv) was dissolved in THF (5 mL), and triethylamine (7.9 mg, 0.078 mmol, 2 equiv) was added. The reaction was cooled to 0 °C, and vinyl sulfonyl chloride **26** (6.2 mg, 0.039 mmol, 1 equiv) in THF (2 mL) was added. After 5 min the reaction was warmed to room temperature and stirred for 30 min. Methanol (5 mL) was added and the reaction evaporated to dryness. The residue was purified by HPLC (5–95% acetonitrile in water (0.1% formic acid)) to give 2-amino-2-*N*-(4-vinylsulfonylaminobenzyl)-5-furyl-1,3,4-triazole (6 mg, 44%) as a white powder. ^1^H (500 MHz, DMSO-*d*_6_): δ 12.35 (1H, brs), 9.74 (1H, brs), 7.70 (1H, s), 7.26 (2H, d, *J* = 8.4 Hz), 7.10 (2H, d, *J* = 8.4 Hz), 6.73 (2H, dd, *J* = 16.4, 10.0 Hz), 6.55 (1H, brs), 6.07 (1H, d, *J* = 16.5 Hz), 5.99 (1H, d, *J* = 10.0 Hz), 4.31 (1H, d, *J* = 6.4 Hz); ^13^C (125 MHz, DMSO-*d*_6_): δ 136.30, 127.99, 127.43, 119.88, 111.37, 45.68. HRMS *m*/*z* (ESI^+^) calcd for C_15_H_16_N_5_O_3_S [M + H]^+^: 346.0968, found 346.0969 (0.2 ppm).

#### 2-Amino-2-*N*-(4-ethylsulfonylaminobenzyl)-5-furyl-1,3,4-triazole **29**

2-Amino-2-*N*-(4-aminobenzyl)-5-furyl-1,3,4-triazole **27** (10 mg, 0.039 mmol, 1 equiv) was dissolved in THF (5 mL), and triethylamine (11 μL, 0.078 mmol, 2 equiv) was added. The reaction was cooled to 0 °C, and ethane sulfonyl chloride **26** (3.7 μL, 0.039 mmol, 1 equiv) added. The reaction was stirred for 20 min then warmed to room temperature and stirred overnight. LCMS indicated 50% of the starting material remained, so a second portion of ethane sulfonyl chloride (3.7 μL, 0.039 mmol, 1 equiv) was added and the reaction stirred. After a further 4 h, starting material still remained so a further portion of ethane sulfonyl chloride (3.7 μL, 0.039 mmol, 1 equiv) was added. After a further 2 h, starting material still remained so a fourth portion of ethane sulfonyl chloride (3.7 μL, 0.039 mmol, 1 equiv) was added. After 1 h, starting material still remained so a final portion of ethenesulfonyl chloride (3.7 μL, 0.039 mmol, 1 equiv) was added, and another portion of triethylamine (11 μL, 0.078 mmol, 2 equiv) was added. The reaction was stirred overnight at room temperature. The reaction was quenched by the addition of 5 mL methanol and evaporated to dryness. The residue was purified by HPLC (5–95% acetonitrile in water (0.1% formic acid)) to give 2-amino-2-*N*-(4-ethylsulfonylaminobenzyl)-5-furyl-1,3,4-triazole (5 mg, 37%) as a white powder. ^1^H (400 MHz, DMSO-*d*_6_): δ 12.38 (1H, brs), 9.68 (1H, brs), 7.70 (1H, s), 7.29 (2H, d, *J* = 8.4 Hz), 7.15 (2H, d, *J* = 8.4 Hz), 6.73 (1H, brs), 6.55 (1H, brs), 4.32 (2H, d, *J* = 6.4 Hz), 3.04 (2H, q, *J* = 7.3 Hz), 1.17 (3H, t, *J* = 7.3 Hz); ^13^C (100 MHz, DMSO-*d*_6_): δ 143.38, 137.53, 128.65, 120.04, 111.85, 108.12, 46.16, 45.39, 8.46. HRMS *m*/*z* (ESI^+^) calcd for C_15_H_18_N_5_O_3_S [M + H]^+^: 348.1125, found 348.1129 (1.1 ppm).

#### 3-(Boc)aminopropanal **34**

Dry DCM (40 mL) was cooled to −78 °C under nitrogen, and oxalyl chloride (0.72 mL, 8.56 mmol, 1.5 equiv) was added. DMSO (1.21 mL, 17.12 mmol, 3 equiv) was added and the reaction stirred at −78 °C for 30 min. 3-(Boc)aminopropanol (**31**) (1.00 g, 5.71 mmol, 1 equiv) was dissolved in dry DCM (10 mL) and added. The reaction was stirred for 30 min then triethylamine (3.98 mL, 28.53 mmol, 5 equiv) was added and the reaction allowed to slowly warm to room temperature overnight. 40 mL saturated sodium hydrogen carbonate solution was added and stirred vigorously, the layers were separated, and the aqueous layer extracted 2× with 25 mL DCM. The combined organic layers were washed with brine, dried over MgSO_4_, passed through a phase separator, and evaporated to dryness. The residue was purified by flash chromatography (0–80% ethyl acetate in heptane) to give 3-(Boc)aminopropanal (0.956 g, 97%) as a colorless oil. ^1^H (400 MHz, CDCl_3_): δ 9.56 (1H, t, *J* = 1.0 Hz), 3.19 (2H, q, *J* = 6.1 Hz), 2.47 (2H, t, *J* = 5.9 Hz), 1.19 (9H, s). Analysis is in agreement with the literature.^19^

#### 1-Boc-2-hydroxypyrrolidine **35**

Dry DCM (40 mL) was cooled to −78 °C under nitrogen, and oxalyl chloride (0.67 mL, 7.93 mmol, 1.5 equiv) was added. DMSO (1.12 mL, 15.9 mmol, 3 equiv) was added and the reaction stirred at −78 °C for 30 min. 4-(Boc)aminobutanol (**31**) (1.00 g, 5.28 mmol, 1 equiv) was dissolved in dry DCM (10 mL) and added. The reaction was stirred for 30 min then triethylamine (3.68 mL, 26.42 mmol, 3 equiv) was added, and the reaction was allowed to slowly warm to room temperature overnight. Twenty mL saturated sodium hydrogen carbonate solution was added and stirred vigorously, the layers were separated, and the aqueous layer extracted 2× with 25 mL DCM. The combined organic layers were dried over MgSO_4_, passed through a phase separator, and evaporated to dryness. The residue was purified by flash chromatography (0–50% ethyl acetate in heptane) to give 1-Boc-2-hydroxypyrrolidine (965 mg, 98%) as a pale yellow oil. ^1^H (500 MHz, CDCl_3_): δ 5.26 (1H, m), 3.30 (1H, m) 3.07 (1H, m), 1.89 (1H, m), 1.72 (2H, m) 1.64 (1H, m), 1.28 (9H, m); ^13^C (125 MHz, CDCl_3_): δ154.66, 81.00, 79.48, 45.60, 32.74, 28.16, 22.43. Analysis is in agreement with the literature.^20^

#### 1-Boc-2-hydroxypiperidine **36**

Dry DCM (40 mL) was cooled to −78 °C under nitrogen, and oxalyl chloride (0.53 mL, 6.27 mmol, 1.5 equiv) was added. DMSO (0.89 mL, 12.54 mmol, 3 equiv) was added and the reaction stirred at −78 °C for 30 min. 5-(Boc)aminopentanol (**32**) (850 mg, 4.18 mmol, 1 equiv) was dissolved in dry DCM (10 mL) and added. The reaction was stirred for 30 min then triethylamine (2.91 mL, 20.91 mmol, 5 equiv) was added, and the reaction was allowed to slowly warm to room temperature overnight. 40 mL saturated sodium hydrogen carbonate solution was added and stirred vigorously, the layers were separated, and the aqueous layer extracted 2× with 25 mL DCM. The combined organic layers were washed with brine, dried over MgSO_4_, passed through a phase separator, and evaporated to dryness. The residue was purified by flash chromatography (0–80% ethyl acetate in heptane) to give 1-Boc-2-hydroxypiperidine (868 mg, 100%) as a colorless oil. ^1^H (500 MHz, CDCl_3_): δ 6.59 (1H, d, *J* = 2.0 Hz), 3.65 (1H, d, *J* = 9.3 Hz), 2.98 (1H, t, *J* = 11.9 Hz), 1.69 (2H, m), 1.53 (1H, d, *J* = 12.7 Hz), 1.42 (2H, m), 1.33 (9H, s), 1.29 (1H, m). ^13^C (125 MHz, CDCl_3_): δ 155.06, 79.74, 74.07, 38.85, 30.67, 28.22, 24.82, 17.67. Analysis is in agreement with the literature.^20^

#### 6-(Boc)aminohexanal **37**

Dry DCM (40 mL) was cooled to −78 °C under nitrogen, and oxalyl chloride (0.58 mL, 6.90 mmol, 1.5 equiv) was added. DMSO (0.98 mL, 13.81 mmol, 3 equiv) was added and the reaction stirred at −78 °C for 30 min. 6-(Boc)aminohexanol (**33**) (1.00 g, 4.60 mmol, 1 equiv) was dissolved in dry DCM (10 mL) and added. The reaction was stirred for 30 min then triethylamine (3.21 mL, 23.01 mmol, 5 equiv) was added and the reaction allowed to slowly warm to room temperature overnight. 40 mL saturated sodium hydrogen carbonate solution was added and stirred vigorously, the layers were separated, and the aqueous layer extracted 2× with 25 mL DCM. The combined organic layers were washed with brine, dried over MgSO_4_, passed through a phase separator, and evaporated to dryness. The residue was purified by flash chromatography (0–60% ethyl acetate in heptane) to give 6-(Boc)aminohexanal (0.9210g 93%) as a pale yellow oil. ^1^H (500 MHz, CDCl_3_): δ 9.55 (1H, t, *J* = 1.7 Hz), 4.87 (1H, brs), 2.91 (2H, q, *J* = 6.4 Hz), 2.24 (2H, td, *J* = 7.3, 1.7 Hz), 1.45 (2H, quint, *J* = 7.5 Hz), 1.31 (2H, quint, *J* = 7.3 Hz), 1.23 (9H, s), 1.16 (2H, m). Analysis is in agreement with the literature.^21^

#### 2-(3-(Boc)aminopropylamino)-5-furyl-1,3,4-triazole **38**

3-(Boc)aminopropanal **34** (1.30 g, 7.50 mmol, 1.5 equiv), 2-amino-5-furyl-1,3,4-triazole **14** (750 mg, 5.00 mmol, 1 equiv) and sodium triacetoxyborohydride (5.29 g, 25.0 mmol, 5 equiv) were dissolved in THF (50 mL) with 3 Å molecular sieves. Acetic acid (0.57 mL, 10.0 mmol, 2 equiv) was added, and the reaction was stirred for 3 days at room temperature. LCMS indicated the imine had been completely reduced so the reaction was quenched with 20 mL 2 N sodium hydroxide solution, filtered, and the aqueous layer extracted 2× with 25 mL ethyl acetate. The combined organic layers were washed with brine, dried over MgSO_4_ and evaporated to dryness. The residue was purified by flash chromatography (0–5% MeOH in DCM) to give 2-(3-(Boc)aminopropylamino)-5-furyl-1,3,4-triazole (695 mg, 45%) as a white foam. ^1^H (500 MHz, MeOD-*d*_4_): δ 7.59 (1H, s), 6.94 (1H, d, *J* = 3.2 Hz), 6.54 (1H, dd, *J* = 3.0, 1.6 Hz), 3.34 (2H, t, *J* = 6.6 Hz), 3.17 (2H, t, *J* = 6.3 Hz), 1.78 (2H, quint, *J* = 6.6 Hz), 1.43 (9H, s); ^13^C (125 MHz, MeOD-*d*_4_): δ 158.42, 144.30, 112.35, 110.07, 79.84, 41.53, 38.48, 30.88, 28.76; MS (ESI): *m*/*z* = 308.2 [M + H]^+^.

#### 2-(3-Aminopropylamino)-5-furyl-1,3,4-triazole **42**

2-(3-(Boc)aminopropylamino)-5-furyl-1,3,4-triazole **38** (642 mg, 2.09 mmol, 1 equiv) was dissolved in methanol (20 mL), and hydrogen chloride (2 M in ether) (15.7 mL, 31.33 mmol, 15 equiv) was added. The reaction was allowed to stir overnight. LCMS indicated complete conversion, so the mixture was evaporated to dryness. The residue purified by SCX eluting with 3.5 N NH_3_ in methanol to give 2-(3-aminopropylamino)-5-furyl-1,3,4-triazole (430 mg, 99%) as a pale brown oil. ^1^H (500 MHz, MeOD-*d*_4_): δ 7.53 (1H, s), 6.83 (1H, m), 6.48 (1H, m), 3.27 (2H, m), 2.73 (2H, m), 1.78 (2H, m).

#### 2-(4-(Boc)aminobutylamino)-5-furyl-1,3,4-triazole **39**

1-Boc-2-hydroxypyrrolidine **35** (281 mg, 1.50 mmol, 1.5 equiv), 2-amino-5-furyl-1,3,4-triazole **14** (150 mg, 1.00 mmol, 1 equiv), and sodium triacetoxyborohydride (1059 mg, 5.00 mmol, 5 equiv) were dissolved in THF (10 mL) with 3 Å molecular sieves. Acetic acid (0.11 mL, 2.00 mmol, 2 equiv) was added, and the reaction was stirred for 3 days at room temperature. LCMS indicated that unreduced imine still remained, so sodium borohydride (76 mg, 2 mmol, 2 equiv) was added and the reaction stirred overnight. Four mL 2 N NaOH solution was added and the mixture stirred. The layers were separated, and the aqueous layer extracted 2× with 15 mL ethyl acetate. The combined organic layers were dried over MgSO_4_, passed through a phase separator, and evaporated to dryness. The residue was purified by flash chromatography (0–5% MeOH in DCM) to give 2-(4-(Boc)aminobutylamino)-5-furyl-1,3,4-triazole (111 mg, 35%). ^1^H (400 MHz, MeOD-*d*_4_): δ 7.59 (1H, dd, *J* = 1.7, 0.7 Hz), 6.89 (1H, d, *J* = 3.4 Hz), 6.53 (1H, dd, *J* = 3.4, 1.8 Hz), 3.27 (2H, t, *I* = 6.9 Hz), 3.07 (2H, t, *J* = 6.7 Hz), 1.68–1.51 (4H, m), 1.42 (9H, s); MS (ESI): *m*/*z* = 322.2 [M + H]^+^.

#### 2-(4-Aminobutylamino)-5-furyl-1,3,4-triazole **43**

2-(4-(Boc)aminobutylamino)-5-furyl-1,3,4-triazole **39** (238 mg, 0.74 mmol, 1 equiv) was dissolved in methanol (2 mL), and hydrogen chloride (2 M in ether)(1.85 mL, 3.70 mmol, 5 equiv) was added and the reaction stirred at room temperature overnight. The reaction was evaporated to dryness, dissolved in methanol, and purified by SCX eluting with 3.5 N NH_3_ in methanol to give 2-(4-aminobutylamino)-5-furyl-1,3,4-triazole (144 mg, 88%) as a brown powder. ^1^H (500 MHz, MeOD-*d*_4_): δ 7.53 (1H, s), 6.84 (1H, d, *J* = 2.7 Hz), 6.47 (1H, m), 3.22 (2H, t, *J* = 6.9 Hz), 2.65 (2H, t, *J* = 6.1 Hz), 1.58 (2H, quint, *J* = 6.9 Hz), 1.50 (2H, quint, *J* = 6.9 Hz).

#### 2-(5-(Boc)aminopentylamino)-5-furyl-1,3,4-triazole **40**

2-Amino-5-furyl-1,3,4-triazole **14** (500 mg, 3.33 mmol, 1 equiv), sodium triacetoxyborohydride (1412 mg, 6.66 mmol, 2 equiv), and 1-Boc-2-hydroxypiperidine **36** (871 mg, 4.33 mmol, 1.3 equiv) were dissolved in THF (30 mL) over MgSO_4_. Acetic acid (0.38 mL, 6.66 mmol, 2 equiv) was added and the reaction stirred at 60 °C overnight. Ten mL 2 N NaOH solution was added and the layers separated. The aqueous layer was extracted 2× with 25 mL ethyl acetate, the combined organic layers were dried over MgSO_4_, and evaporated to dryness. The residue was purified by flash chromatography (0–10% MeOH in DCM) to give 2-(5-aminopentylamino)-5-furyl-1,3,4-triazole (207 mg, 19%) as a colorless oil. ^1^H (500 MHz, MeOD-*d*_4_): δ 7.51 (1H, s), 6.94 (1H, s), 6.52 (1H, s), 3.59 (2H, m), 3.14 (2H, s), 1.61 (2H, s), 1.52 (2H, s), 1.48 (9H, s), 1.32 (4H, m).

#### 2-(5-Aminopentylamino)-5-furyl-1,3,4-triazole **44**

2-(5-(Boc)aminopentylamino)-5-furyl-1,3,4-triazole **40** (280 mg, 0.83 mmol, 1 equiv) was dissolved in methanol (2 mL), and hydrogen chloride (2 M in ether) (2.09 mL, 4.17 mmol, 5 equiv) was added and the reaction stirred at room temperature overnight. The reaction was evaporated to dryness, dissolved in methanol, and purified by SCX eluting with 3.5 N NH_3_ in methanol to give 2-(5-aminopentylamino)-5-furyl-1,3,4-triazole (155 mg, 79%) as a brown oil. ^1^H (500 MHz, MeOD-*d*_4_): δ 7.57 (1H, dd, *J* = 5.7, 1.0 Hz), 6.86 (1H, dd, *J* = 18.4, 3.3 Hz), 6.52 (1H, m), 3.55 (2H, t, *J* = 6.5 Hz), 3.25 (1H, t, *J* = 7.0 Hz), 2.65 (2H, t, *J* = 6.6 Hz), 1.65–1.43 (4H, m), 1.38 (2H, m); HRMS *m*/*z* (ESI^+^) calcd for C_11_H_18_N_5_O [M + H]^+^: 236.1517, found 236.1571 (22.8 ppm).

#### 2-(6-(Boc)aminohexylamino)-5-furyl-1,3,4-triazole **41**

2-Amino-5-furyl-1,3,4-triazole **14** (500 mg, 3.33 mmol, 1 equiv), 6-(Boc)aminohexanal **37** (717 mg, 3.33 mmol, 1 equiv), and acetic acid (0.38 mL, 6.66 mmol, 2 equiv) were dissolved in dry THF (30 mL) under nitrogen, and sodium triacetoxyborohydride (1412 mg, 6.66 mmol, 2 equiv) was added. The reaction was stirred at room temperature overnight. LCMS indicated some imine remained, so a second portion of sodium triacetoxyborohydride (1412 mg, 6.66 mmol, 2 equiv) was added, and the reaction stirred for a further 3 h. Twenty mL 2 N NaOH was added, the layers separated, and the aqueous layer extracted 2× with ethyl acetate. The combined organic layers were dried over MgSO_4_ and evaporated to dryness. The residue was purified by flash chromatography (0–6% MeOH in DCM) to give 2-(6-(Boc)aminohexylamino)-5-furyl-1,3,4-triazole (554 mg, 48%) as a white solid. ^1^H (500 MHz, MeOD-*d*_4_): δ 7.57 (1H, d, *J* = 1.3 Hz), 6.89 (1H, d, *J* = 2.9 Hz), 6.52 (1H, dd, *J* = 3.4, 1.8 Hz), 3.24 (2H, t, *J* = 7.1 Hz), 3.02 (2H, t, *J* = 7.0 Hz), 1.60 (2H, quint, *J* = 7.3 Hz), 1.47 (2H, m), 1.41 (9H, s), 1.35 (4H, m); ^13^C (125 MHz, MeOD-*d*_4_): δ 158.48, 147.86, 144.36, 112.38, 110.00, 79.74, 44.24, 41.23, 30.87, 30.63, 28.79, 27.52, 27.50. MS (ESI): *m*/*z* = 350.2 [M + H]^+^.

#### 2-(6-Aminohexylamino)-5-furyl-1,3,4-triazole **45**

2-(6-(Boc)aminohexylamino)-5-furyl-1,3,4-triazole **41** (794 mg, 2.27 mmol, 1 equiv) was dissolved in methanol (20 mL), and hydrogen chloride (2 M in Ether) (17.0 mL, 34.08 mmol, 15 equiv) was added. The reaction was allowed to stir overnight. LCMS indicated complete conversion, so the mixture was evaporated to dryness. The residue purified by SCX eluting with 3.5 N NH_3_ in methanol to give 2-(6-aminohexylamino)-5-furyl-1,3,4-triazole (546 mg, 96%). ^1^H (500 MHz, MeOD-*d*_4_): δ 7.53 (1H, d, *J* = 1.1 Hz), 6.84 (1H, d, *J* = 3.3 Hz), 6.48 (1H, dd, *J* = 3.4, 1.8 Hz), 3.20 (2H, t, *J* = 7.1 Hz), 2.59 (2H, t, *J* = 7.1 Hz), 1.55 (2H, quint, *J* = 7.1 Hz), 1.42 (2H, quint, *J* = 7.0 Hz), 1.36–1.24 (4H, m); MS (ESI): *m*/*z* = 250.2 [M + H]^+^.

#### 2-(3-Acrylamidopropylamino)-5-furyl-1,3,4-triazole **46**

2-(3-(Boc)aminopropylamino)-5-furyl-1,3,4-triazole **42** (20 mg, 0.097 mmol, 1 equiv) was dissolved in DCM (2 mL) with triethylamine (40.4 μL, 0.29 mmol, 3 equiv) and cooled to 0 °C. Prop-2-enoyl chloride (7.8 μL, 0.097 mmol, 1 equiv) was added and the reaction allowed to warm to room temperature overnight. Methanol (2 mL) was added, stirred for 30 min. and the reaction evaporated to dryness. The residue was purified by HPLC (5–95% acetonitrile in water (0.1% formic acid)) to give 2-(3-acrylamidopropylamino)-5-furyl-1,3,4-triazole (8 mg, 32%) as a white powder. ^1^H (500 MHz, MeOD-*d*_4_): δ 7.60 (1H, d, *J* 1.2 Hz), 6.89 (1H, d, *J* = 3.4 Hz), 6.54 (1H, dd, *J* = 3.4, 1.8 Hz), 6.24 (2H, m), 5.66 (1H, dd, *J* = 8.6, 3.4 Hz), 3.36 (2H, t, *J* = 6.9 Hz), 3.32 (2H, m), 1.84 (2H, quint, *J* = 6.8 Hz); ^13^C (125 MHz, MeOD-*d*_4_): δ 144.37, 132.04, 126.63, 112.43, 110.14, 41.74, 37.79, 30.48. HRMS *m*/*z* (ESI^+^) calcd for C_12_H_16_N_5_O_2_ [M + H]^+^; 262.1299, found 262.1306 (2.8 ppm).

#### 2-(3-Propionamidopropylamino)-5-furyl-1,3,4-triazole **50**

2-(3-(Boc)aminopropylamino)-5-furyl-1,3,4-triazole **42** (20 mg, 0.097 mmol, 1 equiv) was dissolved in DCM (3 mL) with triethylamine (40.4 μL, 0.29 mmol, 3 equiv) and cooled to 0 °C. Propanoyl chloride (8.4 μL, 0.097 mmol, 1 equiv) was added and the reaction allowed to warm to room temperature overnight. Methanol (3 mL) was added, stirred for 30 min, and the reaction evaporated to dryness. The residue was purified by HPLC (5–95% acetonitrile in water (0.1% formic acid)) to give 2-(3-propionamidopropylamino)-5-furyl-1,3,4-triazole (11 mg, 41%) as a white powder. ^1^H (500 MHz, MeOD-*d*_4_): δ 7.60 (1H, d, *J* = 1.1 Hz), 6.89 (1H, d, *J* = 3.3 Hz), 6.54 (1H, dd, *J* = 3.3, 1.8 Hz), 3.33–3.24 (4H, m), 2.21 (2H, q, *J* = 7.7 Hz), 1.80 (2H, quint, *J* = 6.8 Hz), 1.13 (3H, t, *J* = 7.7 Hz); ^13^C (125 MHz, MeOD-*d*_4_): δ 177.34, 144.58, 112.51, 110.16, 41.77, 37.78, 30.60, 30.32, 10.60; HRMS *m*/*z* (ESI^+^) calcd for C_12_H_18_N_5_O_2_ [M + H]^+^; 264.1455, found 264.1452 (1.0 ppm).

#### 2-(3-(Vinyl)sulfonamidopropylamino)-5-furyl-1,3,4-triazole **54**

2-(3-(Boc)aminopropylamino)-5-furyl-1,3,4-triazole **42** (20 mg, 0.097 mmol, 1 equiv) was dissolved in DCM (2 mL) with triethylamine (40.4 μL, 0.29 mmol, 3 equiv) and cooled to 0 °C. 2-Chloroethanesulfonyl chloride (10.1 μL, 0.097 mmol, 1 equiv) was added and the reaction allowed to slowly warm to room temperature overnight. The residue was purified by HPLC (5–95% acetonitrile in water (0.1% formic acid)) to give 2-(3-(vinyl)sulfonamidopropylamino)-5-furyl-1,3,4-triazole (5 mg, 17%) yield. ^1^H (500 MHz, MeOD-*d*_4_): δ 7.60 (1H, dd, *J* = 1.7, 0.6 Hz), 6.90 (1H, d, *J* = 3.4 Hz), 6.62 (1H, dd, *J* – 16.6, 10.0 Hz), 6.54 (1H, dd, *J* = 3.4, 1.8 Hz), 6.13 (1H, d, *J* = 16.6 Hz), 5.94 (1H, d, *J* = 10.0 Hz), 3.37 (2H, t, *J* = 6.7 Hz), 3.06 (2H, t, *J* = 6.7 Hz), 1.83 (2H, quint, *J* = 6.7 Hz); ^13^C (125 MHz, MeOD-*d*_4_): δ 144.212, 137.74, 126.54, 112.17, 109.81, 41.45, 41.22, 31.11; HRMS *m*/*z* (ESI^+^) calcd for C_11_H_16_N_5_O_3_S [M + H]^+^; 298.0968, found 298.0965 (1.3 ppm).

#### 2-(3-(Ethyl)sulfonamidopropylamino)-5-furyl-1,3,4-triazole **56**

2-(3-(Boc)aminopropylamino)-5-furyl-1,3,4-triazole **43** (20 mg, 0.097 mmol, 1 equiv) was dissolved in DCM (3 mL) with triethylamine (40.4 μL, 0.29 mmol, 3 equiv) and cooled to 0 °C. Ethanesulfonyl chloride (9.1 μL, 0.097 mmol, 1 equiv) was added and the reaction allowed to warm to room temperature overnight. Methanol (3 mL) was added, stirred for 30 min, and the reaction evaporated to dryness. The residue was purified by HPLC (5–95% acetonitrile in water (0.1% formic acid)) to give 2-(3-(ethyl)sulfonamidopropylamino)-5-furyl-1,3,4-triazole (5 mg, 17%) as a white powder. ^1^H (500 MHz, MeOD-*d*_4_): δ 7.59 (1H, s), 6.90 (1H, s), 6.54 (1H, s), 3.15 (2H, t, *J* = 6.7 Hz), 3.11–3.01 (4H, m), 1.84 (2H, quint, *J* = 6.8 Hz), 1.30 (3H, t, *J* = 7.4 Hz); HRMS *m*/*z* (ESI^+^) calcd for C_11_H_18_N_5_O_3_S [M + H]^+^; 300.1129, found 300.1125 (0.1 ppm).

#### 2-(4-Acrylaminobutylamino)-5-furyl-1,3,4-triazole **47**

2-(4-Aminobutylamino)-5-furyl-1,3,4-triazole **43** (20 mg, 0.09 mmol, 1 equiv) and triethylamine (37.8 μL, 0.27 mmol, 3 equiv) were dissolved in DCM (2 mL) and cooled to 0 °C. Prop-2-enoyl chloride (7.3 μL, 0.09 mmol, 1 equiv) was added and the reaction allowed to warm to room temperature overnight. Methanol (2 mL) was added and stirred for 30 min, and the reaction was evaporated to dryness. The residue was purified by HPLC (5–95% acetonitrile in water (0.1% formic acid)) to give 2-(4-acrylaminobutylamino)-5-furyl-1,3,4-triazole (6 mg, 23%) as a white solid. ^1^H (500 MHz, MeOD-*d*_4_): δ 7.59 (1H, s), 6.89 (1H, d, *J* = 3.1 Hz), 6.54 (1H, dd, *J* = 3.3, 1.8 Hz), 6.22 (2H, m, H15), 5.64 (1H, dd, *J* = 8.0, 4.1 Hz), 3.29 (4H, m), 1.65 (4H, m); ^13^C (125 MHz, MeOD-*d*_4_): δ 144.32, 132.07, 126.56, 112.42, 110.00, 43.94, 40.05, 28.11, 27.72. HRMS *m*/*z* (ESI^+^) calcd for C_13_H_18_N_5_O_2_ [M + H]^+^: 276.1455, found 276.1467 (4.4 ppm).

#### 2-(4-Propionaminobutylamino)-5-furyl-1,3,4-triazole **51**

2-(4-Aminobutylamino)-5-furyl-1,3,4-triazole **43** (20 mg, 0.09 mmol, 1 equiv) and triethylamine (37.8 μL, 0.27 mmol, 3 equiv) were dissolved in DCM (2 mL) and cooled to 0 °C. Propanoyl chloride (7.9 μL, 0.09 mmol, 1 equiv) was added and the reaction allowed to warm to room temperature overnight. Methanol (2 mL) was added and stirred for 30 min, and the reaction was evaporated to dryness. The residue was purified by HPLC (5–95% acetonitrile in water (0.1% formic acid)) to give 2-(4-propionaminobutylamino)-5-furyl-1,3,4-triazole (12 mg, 45%) as a white powder. ^1^H (500 MHz, MeOD-*d*_4_): δ 7.59 (1H, s), 6.88 (1H, s), 6.54 (1H, s), 3.28 (2H, t, *J* = 6.8 Hz), 3.21 (2H, t, *J* = 6.7 Hz), 2.18 (2H, q, *J* = 7.6 Hz), 1.62 (4H, m), 1.11 (3H, t, *J* = 7.7 Hz); ^13^C (125 MHz, MeOD-*d*_4_): δ 177.10, 143.89, 112.40, 109.57, 43.95, 39.98, 30.23, 28.05, 27.77, 10.56; HRMS *m*/*z* (ESI^+^) calcd for C_13_H_20_N_5_O_2_ [M + H]^+^; 278.1612, found 278.1611 (0.1 ppm).

#### 2-(4-(Vinyl)sulfonaminobutylamino)-5-furyl-1,3,4-triazole **55**

2-(4-Aminobutylamino)-5-furyl-1,3,4-triazole **43** (20 mg, 0.090 mmol, 1 equiv) and triethylamine (37.8 μL, 0.27 mmol, 3 equiv) were dissolved in DCM (2 mL) and cooled to 0 °C. 2-Chloroethanesulfonyl chloride (9.4 μL, 0.090 mmol, 1 equiv) was added and the reaction allowed to warm to room temperature overnight. Methanol (2 mL) was added and stirred for 30 min, and the reaction was evaporated to dryness. The residue was purified by HPLC (5–95% acetonitrile in water (0.1% formic acid)) to give 2-(4-(vinyl)sulfonaminobutylamino)-5-furyl-1,3,4-triazole (2 mg, 7%) as a colorless oil. ^1^H (400 MHz, MeOD-*d*_4_): δ 7.59 (1H, s), 6.89 (1H, s), 6.62 (1H, dd, *J* = 16.6, 10.0 Hz), 6.54 (1H, s), 6.12 (1H, d, *J* = 16.6 Hz), 5.94 (1H, d, *J* = 10.0 Hz), 3.29 (2H, m), 2.99 (2H, t, *J* = 6.7 Hz), 1.72–1.60 (4H, m); HRMS *m*/*z* (ESI^+^) calcd for C_12_H_18_N_5_O_3_S [M + H]^+^; 312.1125, found 312.1127 (0.8 ppm).

#### 2-(4-(Ethyl)sulfonaminobutylamino)-5-furyl-1,3,4-triazole **59**

2-(4-Aminobutylamino)-5-furyl-1,3,4-triazole **43** (20 mg, 0.09 mmol, 1 equiv) and triethylamine (37.8 μL, 0.27 mmol, 3 equiv) were dissolved in DCM (2 mL) and cooled to 0 °C. Ethanesulfonyl chloride (8.6 μL, 0.09 mmol, 1 equiv) was added and the reaction allowed to warm to room temperature overnight. Methanol (2 mL) was added and stirred for 30 min, and the reaction was evaporated to dryness. The residue was purified by HPLC (5–95% acetonitrile in water (0.1% formic acid)) to give 2-(4-(ethyl)sulfonaminobutylamino)-5-furyl-1,3,4-triazole (2 mg, 7%) as a white powder. ^1^H (500 MHz, MeOD-*d*_4_): δ 7.59 (1H, s), 6.89 (1H, d, *J* = 2.8 Hz), 6.54 (1H, dd, *J* = 3.0, 1.7 Hz), 3.09 (2H, t, *J* = 6.7 Hz), 3.29 (2H, m), 3.03 (2H, q, *J* = 7.4 Hz), 1.67 (4H, m), 1.30 (3H, t, *J* = 7.4 Hz); ^13^C (125 MHz, MeOD-*d*_4_): δ 112.42, 47.08, 43.87, 43.59, 28.70, 27.86, 8.47. HRMS *m*/*z* (ESI^+^) calcd for C_12_H_20_N_5_O_3_S [M + H]^+^; 314.1281, found 314.1275 (2.2 ppm).

#### 2-(5-Acrylaminopentylamino)-5-furyl-1,3,4-triazole **48**

2-(5-Aminopentylamino)-5-furyl-1,3,4-triazole **44** (20 mg, 0.085 mmol, 1 equiv) and triethylamine (35.5 μL, 0.255 mmol, 3 equiv) were dissolved in DCM (2 mL) and cooled to 0 °C. Prop-2-enoyl chloride (6.7 μL, 0.085 mmol, 1 equiv) was added and the reaction allowed to warm to room temperature overnight. Methanol (2 mL) was added and stirred for 30 min, and the reaction was evaporated to dryness. The residue was purified by HPLC (5–95% acetonitrile in water (0.1% formic acid)) to give 2-(5-acrylaminopentylamino)-5-furyl-1,3,4-triazole (4 mg, 15%) as a white powder. ^1^H (500 MHz, MeOD-*d*_4_): δ 8.48 (1H, s), 7.59 (1H, brs), 6.89 (1H, brs), 6.54 (1H, brs), 6.21 (1H, d, *J* = 8.4 Hz), 6.20 (1H, d, *J* = 3.7 Hz), 5.63 (1H, dd, *J* = 8.4, 3.7 Hz), 3.27 (2H, td, *J* = 7.1, 1.6 Hz), 1.54 (6H, m); HRMS *m*/*z* (ESI^+^) calcd for C_14_H_20_N_5_O_2_ [M + H]^+^; 290.1612, found 290.1616 (1.5 ppm).

#### Synthesis of 2-(5-Propionaminopentylamino)-5-furyl-1,3,4-triazole **52**

2-(5-Aminopentylamino)-5-furyl-1,3,4-triazole **44** (20 mg, 0.085 mmol, 1 equiv) and triethylamine (35.5 μL, 0.255 mmol, 3 equiv) were dissolved in DCM (2 mL) and cooled to 0 °C. Propanoyl chloride (7.4 μL, 0.085 mmol, 1 equiv) was added and the reaction allowed to warm to room temperature overnight. Methanol (2 mL) was added and stirred for 30 min, and the reaction was evaporated to dryness. The residue was purified by HPLC (5–95% acetonitrile in water (0.1% formic acid)) to give 2-(5-propionaminopentylamino)-5-furyl-1,3,4-triazole (6 mg, 23%) as a white solid. ^1^H (500 MHz, MeOD-*d*_4_): δ 7.58 (1H, s), 6.87 (1H, s), 6.53 (1H, s), 3.26 (2H, t, *J* = 7.1 Hz), 3.18 (2H, t, *J* = 7.1 Hz), 2.18 (2H, q, *J* = 7.7 Hz), 1.65 (2H, quint, *J* = 7.4 Hz), 1.55 (2H, quint, *J* = 7.3 Hz), 1.44 (2H, m), 1.11 (3H, t, *J* 7.7 Hz); ^13^C (125 MHz, MeOD-*d*_4_): δ 112.53, 109.82, 44.22, 40.23, 30.39, 30.23, 30.16, 25.18, 10.57; HRMS *m*/*z* (ESI^+^) calcd for C_14_H_22_N_5_O_2_ [M + H]^+^; 292.1768, found 292.1782 (4.8 ppm).

#### Synthesis of 2-(5-(Ethyl)sulfonaminopentylamino)-5-furyl-1,3,4-triazole **60**

2-(5-Aminopentylamino)-5-furyl-1,3,4-triazole **44** (20 mg, 0.085 mmol, 1 equiv) and triethylamine (35.5 μL, 0.26 mmol, 3 equiv) were dissolved in DCM (2 mL) and cooled to 0 °C. Ethanesulfonyl chloride (8.1 μL, 0.085 mmol, 1 equiv) was added and the reaction allowed to warm to room temperature overnight. Methanol (2 mL) was added and stirred for 30 min, and the reaction was evaporated to dryness. The residue was purified by HPLC (5–95% acetonitrile in water (0.1% formic acid)) to give 2-(5-(ethyl)sulfonaminopentylamino)-5-furyl-1,3,4-triazole (4 mg, 14%) as a white powder. ^1^H (500 MHz, MeOD-*d*_4_): δ 7.59 (1H, dd, *J* = 1.7, 0.6 Hz), 6.89 (1H, d, *J* = 3.2 Hz), 6.54 (1H, dd, *J* = 3.4, 1.8 Hz), 3.29 (2H, d, *J* = 7.2 Hz), 3.04 (2H, m), 2.97 (2H, m), 1.70 (4H, m), 1.49 (2H, m), 1.24 (3H, t, *J* = 7.2 Hz).

#### Synthesis of 2-(6-Acrylaminohexylamino)-5-furyl-1,3,4-triazole **49**

2-(6-Aminohexylamino)-5-furyl-1,3,4-triazole **45** (20 mg, 0.080 mmol, 1 equiv) was dissolved in DCM (3 mL) with triethylamine (33.5 μL, 0.24 mmol, 3 equiv) and cooled to 0 °C. Prop-2-enoyl chloride (6.5 μL, 0.080 mmol, 1 equiv) was added and the reaction allowed to warm to room temperature overnight. Methanol (3 mL) was added, stirred for 30 min, and the reaction evaporated to dryness. The residue was purified by HPLC (5–95% acetonitrile in water (0.1% formic acid)) to give 2-(6-acrylaminohexylamino)-5-furyl-1,3,4-triazole (14 mg, 55%) as a white powder. ^1^H (500 MHz, MeOD-*d*_4_): δ 7.58 (1H, s), 6.89 (1H, d, *J* = 2.9 Hz), 6.54 (1H, dd, *J* = 3.2, 1.8 Hz), 6.20 (2H, m), 5.63 (1H, dd, *J* = 8.6, 3.5 Hz), 3.26 (4H, td, *J* = 7.1, 2.2 Hz), 1.63 (2H, quint, *J* = 7.2 Hz), 1.56 (2H, quint, *J* = 7.2 Hz), 1.42 (4H, m); ^13^C (125 MHz, MeOD-*d*_4_): δ 168.12, 144.32, 132.11, 126.40, 112.40, 109.99, 44.25, 40.34, 30.65, 30.30, 27.71, 27.51; HRMS *m*/*z* (ESI^+^) calcd for C_15_H_22_N_5_O_2_ [M + H]^+^; 304.1768, found 304.1778 (3.4 ppm).

#### 2-(6-Propionaminohexylamino)-5-furyl-1,3,4-triazole **53**

2-(6-Aminohexylamino)-5-furyl-1,3,4-triazole **45** (20 mg, 0.08 mmol, 1 equiv) was dissolved in DCM (3 mL) with triethylamine (33.5 μL, 0.24 mmol, 3 equiv) and cooled to 0 °C. Propanoyl chloride (7.0 μL, 0.08 mmol, 1 equiv) was added and the reaction allowed to warm to room temperature overnight. Methanol (3 mL) was added, stirred for 30 min, and the reaction evaporated to dryness. The residue was purified by HPLC (5–95% acetonitrile in water (0.1% formic acid)) to give 2-(6-propionaminohexylamino)-5-furyl-1,3,4-triazole (10 mg, 39%) as a white powder. ^1^H (500 MHz, MeOD-*d*_4_): δ 7.59 (1H, s), 6.88 (1H, s), 6.54 (1H, s), 3.26 (2H, t, *J* = 7.1 Hz), 3.16 (2H, t, *J* = 7.1 Hz), 2.17 (2H, q, *J* = 7.1 Hz), 1.63 (2H, quint, *J* = 7.2 Hz), 1.52 (2H, quint, *J* = 7.2 Hz), 1.47–1.34 (4H, m), 1.11 (3H, t, *J* = 7.7 Hz); ^13^C (125 MHz, MeOD-*d*_4_): δ 175.57, 142.85, 110.97, 108.44, 42.82, 38.84, 29.23, 28.94, 28.79, 26.24, 26.09, 9.15; HRMS *m*/*z* (ESI^+^) calcd for C_15_H_24_N_5_O_2_ [M + H]^+^; 306.1925, found 306.1933 (1.9 ppm).

#### 2-(6-(Vinyl)sulfonaminohexylamino)-5-furyl-1,3,4-triazole **57**

2-(6-Aminohexylamino)-5-furyl-1,3,4-triazole **45** (20 mg, 0.080 mmol, 1 equiv) was dissolved in DCM (2 mL) with triethylamine (33.5 μL, 0.24 mmol, 3 equiv) and cooled to 0 °C. 2-Chloroethanesulfonyl chloride (8.4 μL, 0.080 mmol, 1 equiv) was added and the reaction allowed to slowly warm to room temperature overnight. Five mL methanol was added and the reaction evaporated to dryness. The residue was purified by HPLC (5–95% acetonitrile in water (0.1% formic acid)) to give 2-(6-(vinyl)sulfonaminohexylamino)-5-furyl-1,3,4-triazole (3 mg, 10%) as a colorless oil. ^1^H (500 MHz, MeOD-*d*_4_): δ 7.61 (1H, dd, *J* = 1.8, 0.8 Hz), 6.92 (1H, dd, *J* = 3.4, 0.8 Hz), 6.61 (1H, dd, *J* = 16.6, 10.0 Hz), 6.56 (1H, dd, *J* = 3.5, 1.8 Hz), 6.11 (1H, d, *J* = 16.6 Hz), 5.94 (1H, d, *J* = 10.0 Hz), 3.27 (2H, t, *J* = 7.1 Hz), 2.94 (2H, t, *J* = 7.0 Hz), 1.64 (2H, quint, *J* = 7.1 Hz), 1.56 (2H, quint, *J* = 7.0 Hz), 1.42 (4H, quint, *J* = 3.7 Hz); ^13^C (125 MHz, MeOD-*d*_4_): δ 144.71, 137.81, 126.16, 112.50, 110.45, 44.30, 43.76, 30.92, 30.56, 27.39; HRMS *m*/*z* (ESI^+^) calcd for C_14_H_22_N_5_O_3_S [M + H]^+^; 340.1448, found 340.1438 (3.0 ppm).

#### Synthesis of 2-(6-(Ethyl)sulfonaminohexylamino)-5-furyl-1,3,4-triazole **61**

2-(6-Aminohexylamino)-5-furyl-1,3,4-triazole **45** (20 mg, 0.08 mmol, 1 equiv) was dissolved in DCM (3 mL) with triethylamine (33.5 μL, 0.24 mmol, 3 equiv) and cooled to 0 °C. Ethanesulfonyl chloride (7.6 μL, 0.08 mmol, 1 equiv) was added and the reaction allowed to warm to room temperature overnight. Methanol (3 mL) was added, stirred for 30 min, and the reaction evaporated to dryness. The residue was purified by HPLC (5–95% acetonitrile in water (0.1% formic acid)) to give 2-(6-(ethyl)sulfonaminohexylamino)-5-furyl-1,3,4-triazole (9 mg, 31%) as a white powder. ^1^H (500 MHz, MeOD-*d*_4_): δ 7.59 (1H, s), 6.89 (1H, s), 6.54 (1H, s), 3.27 (2H, t, *J* = 7.1 Hz), 3.03 (4H, m), 1.64 (2H, quint, *J* = 7.1 Hz), 1.57 (2H, quint, *J* = 7.0 Hz), 1.44 (4H, quint, *J* = 3.6 Hz), 1.30 (3H, t, *J* = 7.4 Hz); ^13^C (125 MHz, MeOD-*d*_4_): δ 144.05, 112.10, 109.72, 47.01, 44.24, 43.83, 31.35, 30.65, 27.42, 27.39, 8.47; HRMS *m*/*z* (ESI^+^) calcd for C_14_H_24_N_5_O_3_S [M + H]^+^; 342.1594, found 342.1560 (0.9 ppm).

#### Synthesis of 2-Propylamino-5-furyl-1,3,4-triazole **62**

2-Amino-5-furyl-1,3,4-triazole **14** (100 mg, 0.67 mmol, 1 equiv) was dissolved in THF (7 mL). Propanal (96 μL, 1.33 mmol, 2 equiv) and sodium triacetoxyborohydride (282 mg, 1.33 mmol, 2 equiv) were added and the reaction stirred at room temperature overnight. Five mL water and 10 mL DCM were added and the layers separated. The aqueous layer was extracted 2× with 10 mL DCM, and the combined organic layers were dried over MgSO_4_, passed through a phase separator, and evaporated to dryness. The residue was purified by flash chromatography (0–100% ethyl acetate in heptane) to give 2-propylamino-5-furyl-1,3,4-triazole (84 mg, 66%) as a white powder. ^1^H (500 MHz, DMSO-*d*_6_): δ 12.14 (1H, s), 7.67 (1H, s), 6.69 (1H, d, *J* = 3.1 Hz), 6.58 (1H, t, *J* = 5.7 Hz), 6.53 (1H, m), 3.10 (2H, q, *J* = 6.7 Hz), 1.53 (2H, sext, *J* = 7.4 Hz), 0.89 (3H, t, *J* = 7.4 Hz); MS (ESI): *m*/*z* = 193.2 [M + H]^+^.

#### Synthesis of 2-Pentylamino-5-furyl-1,3,4-triazole **63**

2-Amino-5-furyl-1,3,4-triazole **14** (100 mg, 0.67 mmol, 1 equiv) was dissolved in THF (7 mL). Pentanal (142 μL,1.33 mmol, 2 equiv) and sodium triacetoxyborohydride (282 mg,1.33 mmol, 2 equiv) were added and the reaction stirred at room temperature overnight. Five mL water and 10 mL DCM were added and the layers separated. The aqueous layer was extracted 2× with 10 mL DCM, and the combined organic layers were dried over MgSO_4_, passed through a phase separator, and evaporated to dryness. The residue was purified by flash chromatography (0–100% ethyl acetate in heptane) to give 2-pentylamino-5-furyl-1,3,4-triazole (84 mg, 57%) as a white powder. ^1^H (400 MHz, MeOD-*d*_4_): δ 7.57 (1H, dd, *J* = 1.7, 0.7 Hz), 6.89, (1H, dd, *J* = 3.4, 0.6 Hz), 6.52 (1H, dd, *J* = 3.4, 1.8 Hz), 3.23 (2H, t, *J* = 7.2 Hz), 1.60 (2H, quint, *J* = 7.2 Hz), 1.35 (4H, m), 1.22 (3H, t, *J* = 7.1 Hz); ^13^C (100 MHz, MeOD-*d*_4_): δ 171.55, 143.02, 111.03, 108.70, 43.00, 29.09, 28.73, 22.11, 13.02; MS (ESI): *m*/*z* = 221.2 [M + H]^+^.

#### 3-TBDPS-oxypropan-1-ol **66**

TBDPSCl (1.00 mL, 3.86 mmol, 1 equiv) was dissolved in DCM (10 mL), and triethylamine (0.81 mL, 5.80 mmol, 3 equiv) and propan-1,3-diol (**64**) (0.84 mL, 11.59 mmol, 3 equiv) were added. The mixture was stirred overnight at room temperature. The reaction was diluted with 30 mL DCM and washed with 15 mL water, 15 mL saturated sodium hydrogen carbonate solution, 15 mL brine, dried over MgSO_4_, passed through a phase separator, and evaporated to dryness. The residue was purified by flash chromatography (0–30% ethyl acetate in heptane) to give 3-TBDPS-oxypropan-1-ol (1.22 g, 100%) as a colorless oil. ^1^H (500 MHz, CDCl_3_): δ 7.69 (4H, d, *J* = 7.6 Hz), 7.42 (6H, m), 3.86 (4H, m), 2.33 (1H, brs), 1.82 (2H, quint, *J* = 5.7 Hz), 1.07 (9H, s); ^13^C (125 MHz, CDCl_3_): δ 135.70, 133.47, 129.91, 127.90, 63.32, 62.00, 35.50, 26.99, 19.24. Analysis is in agreement with the literature.^22^

#### 3-TBDPS-oxypropanal **68**

DCM (15 mL) was cooled to −78 °C, and oxalyl chloride (0.48 mL, 5.64 mmol, 1.5 equiv) was added. DMSO (0.80 mL, 11.28 mmol, 3 equiv) was added and the mixture stirred at −78 °C for 30 min. 3-TBDPS-oxypropan-1-ol **66** (1.18 g, 3.76 mmol, 1 equiv) in DCM (10 mL) was added and the mixture stirred at −78 °C for 30 min. Triethylamine (2.62 mL, 18.79 mmol, 5 equiv) was added and the reaction allowed to slowly warm to room temperature overnight. Ten mL saturated sodium hydrogen carbonate solution and 10 mL water were added, stirred vigorously, and the layers separated. The aqueous layer was extracted 2× with 25 mL DCM. The combined organic layers were dried over MgSO_4_, passed through a phase separator, and evaporated to dryness. The residue was purified by flash chromatography (0–30% ethyl acetate in heptane) to give 3-TBDPS-oxypropanal (951 mg, 81%) as a colorless oil. ^1^H (500 MHz, CDCl_3_): δ 9.83 (1H, t, *J* = 2.2 Hz), 7.66 (4H, dd, *J* = 7.9, 1.4 Hz), 7.42 (6H, m), 4.03 (2H, t, *J* = 6.0 Hz), 2.61 (2H, td, *J* = 6.0, 2.2 Hz), 1.05 (9H, s); ^13^C (125 MHz, CDCl_3_): δ 201.92, 135.70, 133.44, 129.96, 127.92, 58.47, 46.45, 26.91, 19.29. Analysis is in agreement with the literature.^22^

#### Synthesis of 2-*N*-(3-TBDPS-oxypropyl)amino-5-furyl-1,3,4-triazole **70**

2-Amino-5-furyl-1,3,4-triazole **14** (867 mg, 5.77 mmol, 2 equiv), 3-TBDPS-oxypropanal **68** (902 mg, 2.89 mmol, 1 equiv), and sodium triacetoxyborohydride (3.67 g, 17.32 mmol, 6 equiv) were dissolved in THF (25 mL) under nitrogen with 3 Å molecular sieves. Acetic acid (0.33 mL, 5.77 mmol, 2 equiv) was added and the reaction stirred for 24 h. TLC indicated no starting material remained, but LCMS seemed to suggest some unreduced imine remained, so sodium borohydride (218 mg, 5.77 mmol, 2 equiv) was added and the mixture stirred for 1 h. Thirty mL saturated sodium hydrogen carbonate solution was added and mixed until the bubbling was stopped. Ten mL water was added, and the layers separated, and the aqueous layer extracted 3× with 30 mL DCM. The combined organic layers were washed with brine, dried over MgSO_4_, passed through a phase separator, and evaporated to dryness. The residue was purified by flash chromatography (0–6% MeOH in DCM) to give 2-*N*-(3-TBDPS-oxypropyl)amino-5-furyl-1,3,4-triazole (544 mg, 42%) as a white solid. ^1^H (500 MHz, CDCl_3_): δ 7.67 (4H, dt, *J* = 6.4, 1.5 Hz), 7.48 (1H, dd, *J* = 1.7, 0.7 Hz), 7.42 (6H, m), 6.88 (1H, dd, *J* = 3.4, 0.7 Hz), 6.48 (1H, dd, *J* = 3.4, 1.8 Hz), 3.83 (2H, t, *J* = 5.7 Hz), 3.45 (2H, t, *J* = 6.2 Hz), 1.76 (2H, quint, *J* = 5.9 Hz), 1.11 (9H, s); ^13^C (125 MHz, CDCl_3_): δ 143.27, 135.74, 133.33, 130.10, 128.02, 111.57, 109.43, 61.60, 40.84, 32.47, 27.17, 19.38.

#### 2-*N*-(3-Hydroxypropyl)amino-5-furyl-1,3,4-triazole **72**

2-*N*-(3-TBDPSoxypropyl)amino-5-furyl-1,3,4-triazole **70** (366 mg, 0.82 mmol, 1 equiv) was dissolved in THF (10 mL) under nitrogen. TBAF (1 M in THF) (1.64 mL, 1.64 mmol, 2 equiv) was added and the reaction stirred for 3 h when TLC showed no starting material remained. The reaction was evaporated to dryness then dissolved in 10 mL DCM and washed with 10 mL water. The aqueous layer was extracted 2× with 10 mL DCM, and the combined organics were dried over MgSO_4_, passed through a phase separator, and evaporated to dryness. LCMS revealed product remains in the aqueous layer, so the aqueous layer was evaporated to dryness. The combined residues were purified by flash chromatography (0–10% MeOH in DCM) to give 2-*N*-(3-hydroxypropyl)amino-5-furyl-1,3,4-triazole (129 mg, 72%) as a white powder. ^1^H (500 MHz, DMSO-*d*_6_): δ 12.17 (1H, brs), 7.68 (1H, brs), 6.69 (1H, brs), 6.54 (2H, brs), 4.51 (1H, brs), 3.47 (2H, q, *J* = 5.8 Hz), 3.20 (2H, q, *J* = 6.5 Hz), 1.67 (2H, quint, *J* = 6.6 Hz); ^13^C (125 MHz, DMSO-*d*_6_): δ 142.61, 111.22, 107.50, 58.36, 32.42, 23.02.

#### 5-TBDPS-oxypentan-1-ol **67**

Pentan-1,5-diol (**65**) (1.14 mL, 10.92 mmol, 3 equiv) was dissolved in DCM (10 mL) under nitrogen, and triethylamine (0.76 mL, 5.46 mmol, 1.5 equiv) and TBDPSCl (0.95 mL, 3.64 mmol, 1 equiv) were added. The reaction was stirred overnight at room temperature. The reaction was diluted with 30 mL DCM and washed with 15 mL water, 15 mL saturated sodium hydrogen carbonate solution, 15 mL brine, dried over MgSO_4_, passed through a phase separator, and evaporated to dryness. The residue was purified by flash chromatography (0–30% ethyl acetate in heptane) to give 5-TBDPS-oxypentan-1-ol (1.06 g, 85%) as a colorless oil. ^1^H (500 MHz, CDCl_3_): δ 7.67 (4H, dt, *J* = 8.0, 1.1 Hz), 7.40 (6H, m), 3.68 (2H, t, *J* = 6.4 Hz), 3.62 (2H, q, *J* = 6.2 Hz), 1.58 (4H, m), 1.44 (2H, m), 1.06 (9H, d, *J* = 0.8 Hz); ^13^C (125 MHz, CDCl_3_): δ 135.73, 134.26, 129.67, 127.74, 63.95, 63.12, 32.64, 32.43, 27.04, 22.14, 19.37. Analysis is in agreement with the literature.^23^

#### 5-TBDPS-oxypentanal **69**

DCM (15 mL) was cooled to −78 °C, and oxalyl chloride (0.22 mL, 2.59 mmol, 1.5 equiv) was added. DMSO (0.37 mL, 5.18 mmol, 3 equiv) was added and the mixture stirred at −78 °C for 15 min. 5-TBDPS-oxypentan-1-ol **67** (591 mg, 1.73 mmol, 1 equiv) in DCM (10 mL) was added and the mixture stirred for 30 min at −78 °C. Triethylamine (1.20 mL, 8.63 mmol, 5 equiv) was added and the reaction allowed to slowly warm to room temperature overnight. Ten mL saturated sodium hydrogen carbonate solution and 10 mL water were added and stirred vigorously and the layers separated. The aqueous layer was extracted 2× with 25 mL DCM. The combined organic layers were washed with brine, dried over MgSO_4_, passed through a phase separatorm and evaporated to dryness. The residue was purified by flash chromatography (0–30% ethyl acetate in heptane) to give 5-TBDPS-oxypentanal (560 mg, 95%) as a colorless oil. ^1^H (500 MHz, CDCl_3_): δ 9.75 (1H, t, *J* = 1.7 Hz), 7.66 (4H, m), 7.41 (6H, m), 3.68 (2H, t, *J* = 6.2 Hz), 2.41 (2H, td, *J* = 7.3, 1.7 Hz), 1.74 (2H, m), 1.60 (2H, m), 1.06 (9H, s); ^13^C (125 MHz, CDCl_3_): δ 202.68, 135.71, 134.06, 129.75, 127.79, 63.45, 43.57, 32.02, 27.02, 19.36, 18.75. Analysis is in agreement with the literature.^24^

#### 2-*N*-(5-TBDPS-oxypentyl)amino-5-furyl-1,3,4-triazole **71**

2-Amino-5-furyl-1,3,4-triazole **14** (523 mg, 3.48 mmol, 2 equiv), 5-TBDPS-oxypentanal **69** (593 mg, 1.74 mmol, 1 equiv), and sodium triacetoxyborohydride (2.21 g, 10.45 mmol, 6 equiv) were dissolved in THF (25 mL) under nitrogen with 3 Å molecular sieves. Acetic acid (0.20 mL, 3.48 mmol, 2 equiv) was added and the reaction stirred for 24 h. TLC indicated no starting material remained, but LCMS suggested some unreduced imine remained so sodium borohydride (132 mg, 3.48 mmol, 2 equiv) was added and the mixture stirred for 1 h. Thirty mL saturated sodium hydrogen carbonate solution was added and mixed until the bubbling was stopped. Ten mL water were added, and this was extracted 3× with 50 mL DCM. The combined organic layers were washed with brine, dried over MgSO_4_, passed through a phase separator, and evaporated to dryness. The residue was purified by flash chromatography (0–6% MeOH in DCM) to give 2-*N*-(5-TBDPS-oxypentyl)amino-5-furyl-1,3,4-triazole (433 mg, 52%) as a white solid. ^1^H (500 MHz, CDCl_3_): δ 7.66 (4H, m), 7.49 (1H, dd, *J* = 1.7, 0.7 Hz), 7.39 (6H, m), 6.90 (1H, dd, *J* = 3.4, 0.7 Hz), 6.49 (1H, dd, *J* = 3.4, 1.8 Hz), 3.67 (2H, t, *J* = 6.3 Hz), 3.25 (2H, t, *J* = 7.1 Hz), 1.61 (4H, m), 2.78 (2H, m), 1.05 (9H, s); ^13^C (125 MHz, CDCl_3_): δ 135.74, 134.21, 129.72, 127.78, 111.74, 109.78, 63.80, 43.88, 32.27, 29.59, 27.05, 23.26, 19.38.

#### 2-*N*-(5-Hydroxypentyl)amino-5-furyl-1,3,4-triazole **73**

2-*N*-(5-TBDPS-oxypentyl)amino-5-furyl-1,3,4-triazole **71** (214 mg, 0.45 mmol, 1 equiv) was dissolved in THF (10 mL) under nitrogen. TBAF (1 M in THF) (0.90 mL, 0.90 mmol, 2 equiv) was added and the reaction stirred for 3 h when TLC showed no starting material remained. The reaction was evaporated to dryness then dissolved in 10 mL DCM and washed with 10 mL water. The aqueous layer was extracted 2× with 10 mL DCM, and the combined organics were dried over MgSO_4_, passed through a phase separator and evaporated to dryness. LCMS revealed product remains in the aqueous layer, so the aqueous layer was evaporated to dryness. The residues were combined and purified by flash chromatography (0–10% MeOH in DCM) to give 2-*N*-(5-hydroxypentyl)amino-5-furyl-1,3,4-triazole (107 mg, 95%) as a white powder. ^1^H (500 MHz, DMSO-*d*_6_): δ 12.15 (1H, brs), 7.68 (1H, brs), 6.69 (1H, brs), 6.54 (2H, brs), 4.32 (1H, t, *J* = 5.1 Hz), 3.39 (2H, q, *J* = 6.6 Hz), 3.12 (2H, q, *J* = 6.7 Hz), 1.51 (2H, m), 1.42 (2H, m), 1.33 (2H, m); ^13^C (125 MHz, DMSO-*d*_6_): δ 60.61, 42.83, 32.21, 29.10, 22.83.

#### Methyl 5-Hydroxypentanoate **75**

Tetrahydropyran-2-one (**74**) (1 mL, 10.78 mmol, 1 equiv) was dissolved in dry methanol (4.4 mL, 107.8 mmol, 10 equiv) under nitrogen, and triethylamine (0.15 mL, 1.078 mmol, 0.1 equiv) was added. The mixture was stirred at room temperature for 2 h. The mixture was evaporated to dryness to give methyl 5-hydroxypentanoate (1497 mg, 100%) as a colorless oil which was used without further purification. ^1^H (500 MHz, DMSO-*d*_6_): δ 4.35 (1H, t, *J* = 5.2 Hz), 3.58 (3H, s), 3.38 (2H, q, *J* = 6.0 Hz), 2.30 (2H, t, *J* = 7.4 Hz), 1.55 (2H, quint, *J* = 7.5 Hz), 1.41 (2H, quint, *J* = 7.0 Hz); ^13^C (125 MHz, DMSO-*d*_6_): δ 173.29, 60.18, 51.05, 33.06, 31.71, 21.09; HRMS (ESI-TOF) calcd for C_6_H_13_O_3_ [M + H]^+^ = 133.0859, found 133.0857 (1.3 ppm).

#### Methyl 5-Oxopentanoate **76**

DCM (25 mL) was cooled to −78 °C, and oxalyl chloride (0.99 mL, 11.66 mmol, 1.5 equiv) was added. DMSO (1.66 mL, 23.31 mmol, 3 equiv) was added and the mixture stirred for 30 min at −78 °C. Methyl 5-hydroxypentanoate **169** (1 mL, 7.77 mmol, 1 equiv) was added and the mixture stirred at −78 °C for 30 min. Triethylamine (5.42 mL, 38.85 mmol, 5 equiv) was added and the reaction allowed to slowly warm to room temperature overnight. Ten mL saturated sodium hydrogen carbonate solution and 10 mL water were added and stirred vigorously, and the layers were separated. The aqueous layer was extracted 2× with 25 mL DCM. The combined organic layers were dried over MgSO_4_, passed through a phase separator and evaporated to dryness. The residue was purified by flash chromatography (0–50% ethyl acetate in heptane) to give methyl 5-oxopentanoate (812 mg, 80%) as a colorless oil. ^1^H (500 MHz, DMSO-*d*_6_): δ 9.66 (1H, t, *J* = 1.1 Hz), 3.59 (3H, s), 2.48 (2H, td, *J* = 7.3, 1.0 Hz), 2.33 (2H, t, *J* = 7.4 Hz), 1.77 (2H, quint, *J* = 7.3 Hz); ^13^C (125 MHz, DMSO-*d*_6_): δ 200.79, 172.89, 51.16, 42.04, 32.33, 17.03; HRMS (ESI-TOF) calcd for C_6_H_11_O_3_ [M + H]^+^ = 131.0703, found 131.0693 (7.8 ppm).

#### 2-*N*-(*O*-Methyl 4-carboxybutyl)amino-5-furyl-1,3,4-triazole **77**

2-Amino-5-furyl-1,3,4-triazole **14** (500 mg, 3.33 mmol, 1 equiv), methyl 5-oxopentanoate **76** (867 mg, 6.66 mmol, 2 equiv) and sodium triacetoxyborohydride (3.53 g, 16.65 mmol, 5 equiv) were dissolved in THF (25 mL) under nitrogen with 3 Å molecular sieves. Acetic acid (0.38 mL, 6.66 mmol, 2 equiv) was added and the reaction stirred overnight. Thirty mL saturated sodium hydrogen carbonate solution was added and stirred until the fizzing stopped. The layers were separated, and the aqueous layer extracted 3× with 25 mL DCM. The combined organic layers were washed with brine, dried over MgSO_4_, passed through a phase separator, and evaporated to dryness. The residue was purified by flash chromatography (0–10% MeOH in DCM) to give 2-*N*-(*O*-methyl 4-carboxybutyl)amino-5-furyl-1,3,4-triazole (531 mg, 57%) yield as a white powder. ^1^H (500 MHz, DMSO-*d*_6_): δ 12.17 (1H, brs), 7.67 (1H, brs), 6.69 (1H, brs), 6.60 (1H, t, *J* = 5.3 Hz), 6.53 (1H, brs), 3.58 (3H, s), 3.14 (2H, q, *J* = 6.1 Hz), 2.33 (2H, t, *J* = 7.2 Hz), 1.55 (4H, m); ^13^C (125 MHz, DMSO-*d*_6_): δ 51.13, 32.93, 28.59, 21.76.

#### Synthesis of 2-*N*-Piperidin-2-onyl-5-furyl-1,3,4-triazole **78**

2-*N*-(O-Methyl 4-carboxybutyl)amino-5-furyl-1,3,4-triazole **77** (100 mg, 0.38 mmol, 1 equiv) was dissolved in methanol (5 mL), and lithium hydroxide hydrate (0.5 M in water) (0.91 mL, 0.45 mmol, 1.2 equiv) was added. The mixture was stirred overnight. LCMS indicated 100% conversion of starting material, so the mixture was evaporated to dryness. The residue was purified by HPLC (5–95% acetonitrile in water (0.1% formic acid)) to give a white powder which NMR revealed to be the cyclized lactam, 2-*N*-piperidin-2-onyl-5-furyl-1,3,4-triazole (20 mg, 23%). ^1^H (500 MHz, DMSO-*d*_6_): δ 13.61 (1H, brs), 7.77 (1H, dd, *J* = 1.6, 0.6 Hz), 6.86 (1H, dd, *J* = 3.3, 0.6 Hz), 6.60 (1H, dd, *J* = 3.4, 1.8 Hz), 3.92 (2H, t, *J* = 6.0 Hz), 2.54 (1H, t, *J* = 6.6 Hz), 1.89 (2H, m), 1.81 (2H, m); ^13^C (125 MHz, DMSO-*d*_6_): δ 170.01, 143.41, 111.51, 108.56, 47.80, 32.65, 21.86, 19.80.

#### Synthesis of 2-*N*-(4-Carboxybutyl)amino-5-furyl-1,3,4-triazole **79**

2-*N*-Piperidin-2-onyl-5-furyl-1,3,4-triazole **78** (20 mg, 0.086 mmol, 1 equiv) was dissolved in 2 N hydrochloric acid (1.29 mL, 2.58 mmol, 30 equiv) and stirred overnight at room temperature. LCMS showed no conversion so the reaction was heated to 130 °C overnight in a sealed tube. Starting material remained so the reaction was stirred over the weekend at 130 °C. LCMS showed complete conversion so the reaction was evaporated to dryness and the residue purified by HPLC (5–95% acetonitrile in water (0.1% formic acid)) to give 2-*N*-(4-carboxybutyl)amino-5-furyl-1,3,4-triazole (9 mg, 42%) as a white powder. ^1^H (500 MHz, DMSO-*d*_6_): δ 7.86 (1H, s), 7.17 (1H, d, *J* = 2.8 Hz), 6.66 (1H, dd, *J* = 3.3, 1.7 Hz), 3.28 (2H, m), 2.25 (2H, t, *J* = 6.6 Hz), 1.57 (4H, m); ^13^C (125 MHz, DMSO-*d*_6_): δ 174.24, 144.65, 111.81, 111.19, 42.77, 33.23, 28.25, 21.62; MS (ESI): *m*/*z* = 251.1 [M + H]^+^.

#### Methyl 3-Oxopropanoate **81**

Amberlite IR120 resin hydrogen form (10 g) was suspended in acetone (150 mL) and water (6.1 mL, 337 mmol, 10 equiv) added. Methyl 3,3-dimethoxypropanoate (4.78 mL, 33.75 mmol, 1 equiv) was added and the reaction allowed to stir for 3 days. 4 Å molecular sieves were added and the reaction stirred for an hour to remove the water and any methanol. The mixture was passed through a phase separator to further dry it and filter out the resin. The solvent was then removed *in vacuo* to give methyl 3-oxopropanoate (4.07 g) as a yellow oil. NMR showed the residue contained a mixture of starting material and product. This material was used directly in the next step. ^1^H (500 MHz, CDCl_3_): δ 9.80 (1H, t, *J* = 2.4 Hz), 3.78 (3H, s), 3.40 (2H, d, *J* = 2.4 Hz).

#### 2-*N*-(*O*-Methyl 2-carboxyethyl)amino-5-furyl-1,3,4-triazole **82**

2-Amino-5-furyl-1,3,4-triazole **14** (1.00 g, 6.66 mmol, 1 equiv) and methyl 3-oxopropanoate **81** (680 mg, 6.66 mmol, 1 equiv) were dissolved in THF (30 mL) over 3 Å molecular sieves. Acetic acid (0.76 mL, 13.32 mmol, 2 equiv) was added followed by sodium cyanoborohydride (13.3 mL, 13.32 mmol, 2 equiv) (1 M in THF) and the reaction stirred over the weekend. The reaction was filtered through Celite and the residue washed with DCM, water, and again with DCM. The filtrate was neutralized with saturated sodium hydrogen carbonate solution and extracted 3× with 50 mL DCM. The combined organic layers were washed with brine, dried over MgSO_4_, passed through a phase separator, and evaporated to dryness. The residue was purified by flash chromatography (0–5% MeOH in DCM) to give methyl 3-[[3-(2-furyl)-1H-1,2,4-triazol-5-yl]amino]propanoate (839 mg, 53%) as a white powder. ^1^H (500 MHz, DMSO-*d*_6_): δ 12.23 (1H, brs), 7.68 (1H, brs), 6.71 (1H, brs), 6.60 (1H, brs), 6.54 (1H, brs), 3.61 (3H, s), 3.41 (2H, q, *J* = 6.0 Hz), 2.60 (2H, t, *J* = 6.9 Hz); ^13^C (125 MHz, DMSO-*d*_6_): δ 172.34, 157.29, 152.22, 147.47, 142.62, 111.23, 107.59, 51.25, 38.80, 33.87; MS (ESI): *m*/*z* = 237.1 [M + H]^+^.

## Supporting Information Available

Spectral data for the compounds (PDF)
