# Supplementary material for: Design and Synthesis of Covalent Inhibitors of FabA
Source: ACS Omega. 2023 Mar 27;8(14):12787–804. doi: 10.1021/acsomega.2c08031 (PMC10099128; doi:10.1021/acsomega.2c08031)
Supplement: Supplementary file 1 — ao2c08031_si_001.pdf [file ao2c08031_si_001.pdf]

SUPPORTING INFORMATION

Design and Synthesis of Covalent Inhibitors of FabA

James Martin, Claire J. Mackenzie, De Lin, Nadine Homeyer, David W. Gray, Fabio Zuccotto, Ian H. Gilbert*

Wellcome Centre for Anti-Infectives Research, Division of Biological Chemistry and Drug Discovery, University of Dundee, Dundee, DD1 5EH, United Kingdon

Author for correspondence: Ian Gilbert.
i.h.gilbert@dundee.ac.uk

Compound 10

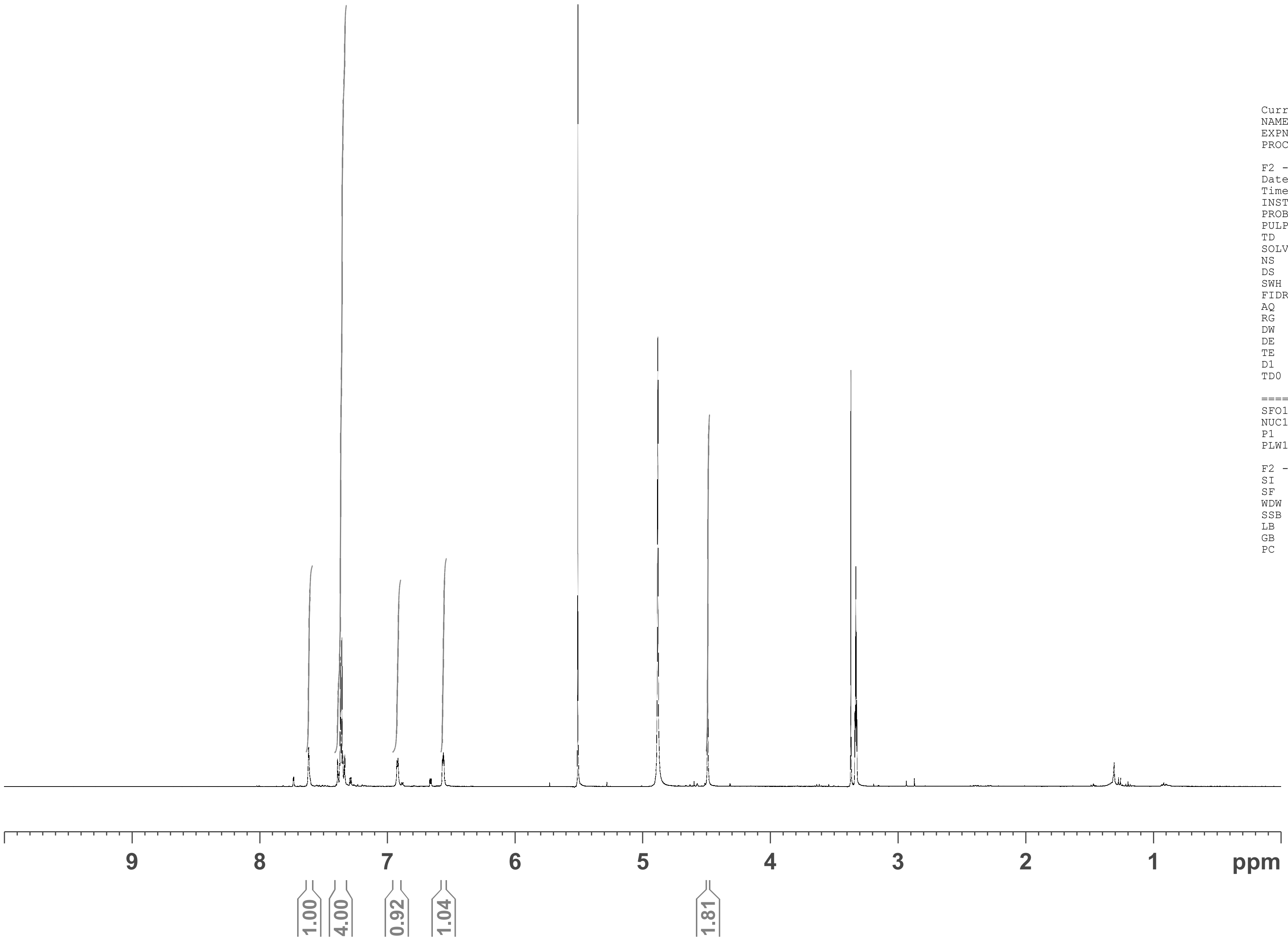


```
Current Data Parameters
NAME      IG-JM-200398-024-001
EXPNO                    1
PROCNO                   1

F2 - Acquisition Parameters
Date_             20161114
Time                 14.40
INSTRUM              spect
PROBHD    5 mm PADUL 13C
PULPROG               zg30
TD                  131072
SOLVENT               MeOD
NS                      16
DS                       4
SWH             12019.230 Hz
FIDRES           0.091699 Hz
AQ              5.4525952 sec
RG                 196.14
DW                 41.600 usec
DE                  12.17 usec
TE                  298.1 K
D1             0.10000000 sec
TD0                      1

======== CHANNEL f1 ========
SFO1          400.1324710 MHz
NUC1                    1H
P1                   10.00 usec
PLW1          20.00000000 W

F2 - Processing parameters
SI                  131072
SF            400.1300000 MHz
WDW                     EM
SSB       0
LB                    0.10 Hz
GB        0
PC                    1.00
```

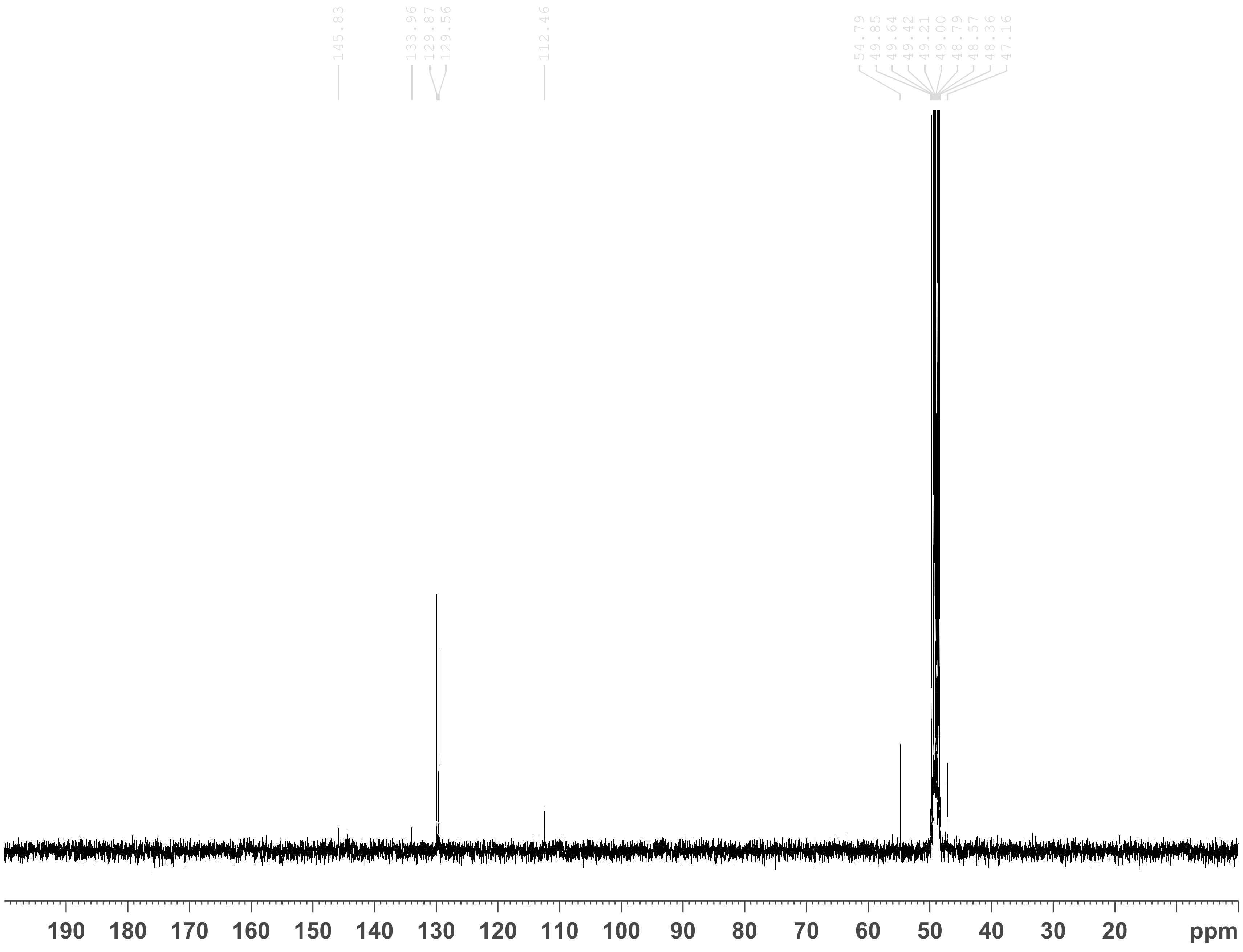


```
Current Data Parameters
NAME     IG-JM-200398-024-00
EXPNO                  2
PROCNO                 1

F2 - Acquisition Parameters
Date_          20161114
Time              15.06
INSTRUM           spect
PROBHD   5 mm PADUL 13C
PULPROG          zgpg30
TD               119044
SOLVENT            MeOD
NS                  256
DS                    4
SWH           25000.000 Hz
FIDRES         0.210006 Hz
AQ            2.3808801 sec
RG               196.14
DW               20.000 usec
DE                 7.92 usec
TE                298.2 K
D1           1.00000000 sec
D11          0.03000000 sec
TD0                   1

======== CHANNEL f1 ========
SFO1        100.6238351 MHz
NUC1                13C
P1                10.00 usec
PLW1        36.00000000 W

======== CHANNEL f2 ========
SFO2        400.1316005 MHz
NUC2                 1H
CPDPRG[2        waltz64
PCPD2             90.00 usec
PLW2        20.00000000 W
PLW12        0.24691001 W
PLW13        0.24691001 W

F2 - Processing parameters
SI               131072
SF          100.6126280 MHz
WDW                  EM
SSB      0
LB                 1.00 Hz
GB       0
PC                 1.40
```

Compound 10

Compound Verification Report (Compass OpenAccess/QC)

Sample-ID	JM-200398-024-001	Station	Microtof-2
Submitter	James Martin	Supervisor	System Administrator
Analysis Name	JM-200398-024-001_9096_RC7_01_9778.d	Acquisition Date	15/11/2016 14:48:15
Sample Description		Method	2-microtof-2 verify compounds lcms pos 5-95.m

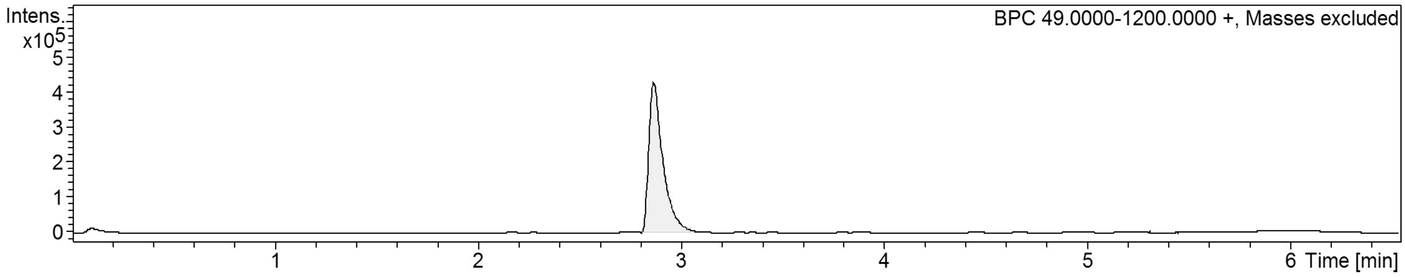

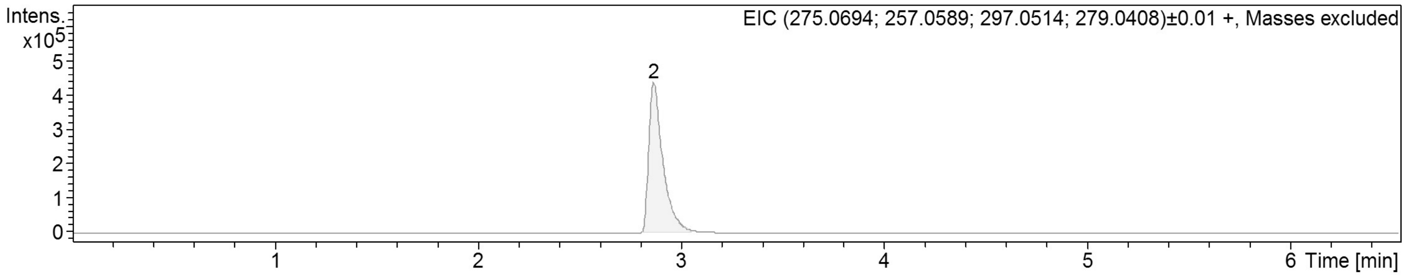

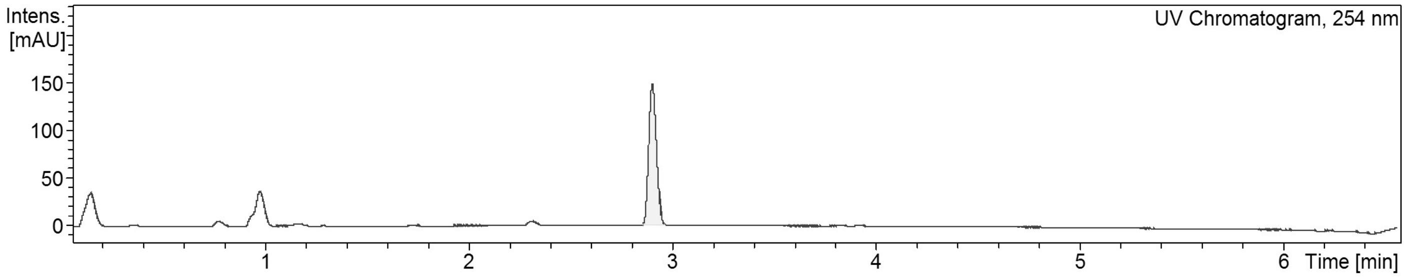

SmartFormula Settings

Tolerance	mSigma Limit	Electron Conf.
10 ppm	60	even

Adduction(s): H, Na **Neutral Loss(es):** H2O

Compound Verification Results

Expected Formula: C13H11ClN4O

#	meas. m/z	theo. m/z	|err| [ppm]	mSigma	Formula	Modification	Purity(UVC)[%]	Purity(BPC)[%]
2	275.0711	275.0694	6.2	28	C13 H12 Cl N4 O	(M+H)+	100.0	100.0

Note: mSigma values <20 indicate high probability of correct molecular formula

Compound Verification Report (Compass OpenAccess/QC)

Cmpd 2, 2.9 min

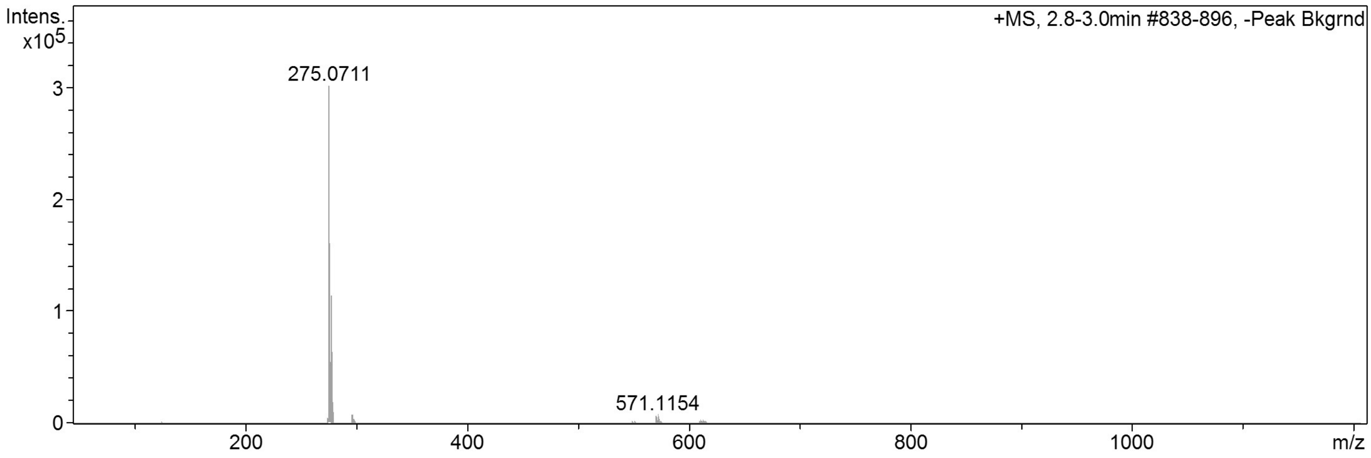

PROTON.d DMSO {C:\Bruker\TopSpin3.2} IG500 9

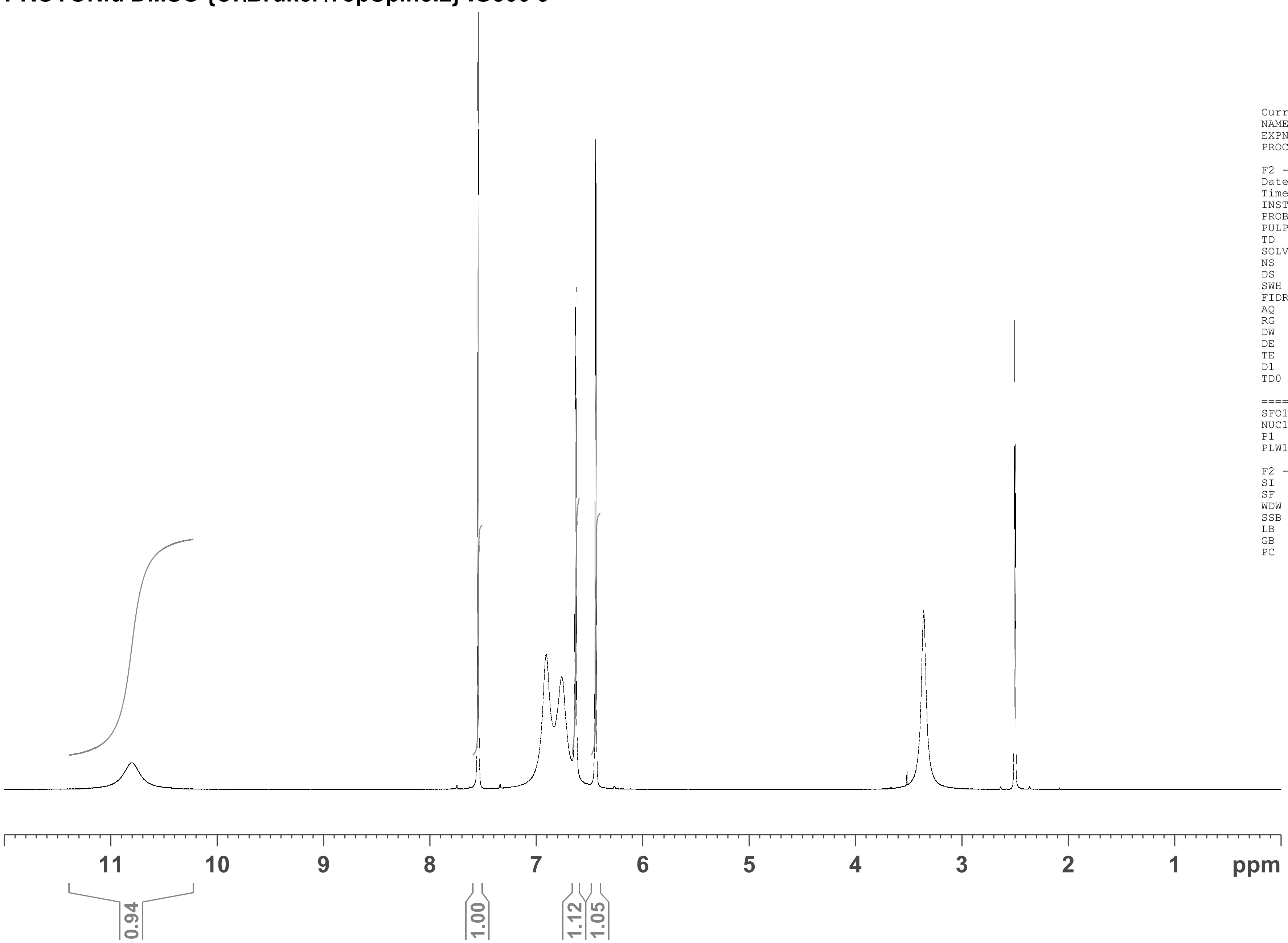


```
Current Data Parameters
NAME      IG-JM-200398-016-001
EXPNO                    1
PROCNO                   1

F2 - Acquisition Parameters
Date_             20161103
Time                 15.12
INSTRUM              spect
PROBHD    5 mm QNP 1H/13
PULPROG               zg30
TD                   65536
SOLVENT               DMSO
NS                      16
DS                       2
SWH             10000.000 Hz
FIDRES           0.152588 Hz
AQ              3.2767999 sec
RG                     287
DW                  50.000 usec
DE                    6.50 usec
TE                   298.2 K
D1             1.00000000 sec
TD0                      1

======== CHANNEL f1 ========
SFO1          500.1330885 MHz
NUC1                    1H
P1                   10.00 usec
PLW1          25.00000000 W

F2 - Processing parameters
SI                   65536
SF            500.1300048 MHz
WDW                     EM
SSB       0
LB                    0.30 Hz
GB        0
PC                    1.00
```

Compound 11

C13CPD.d DMSO {C:\Bruker\TopSpin3.2} IG500 9

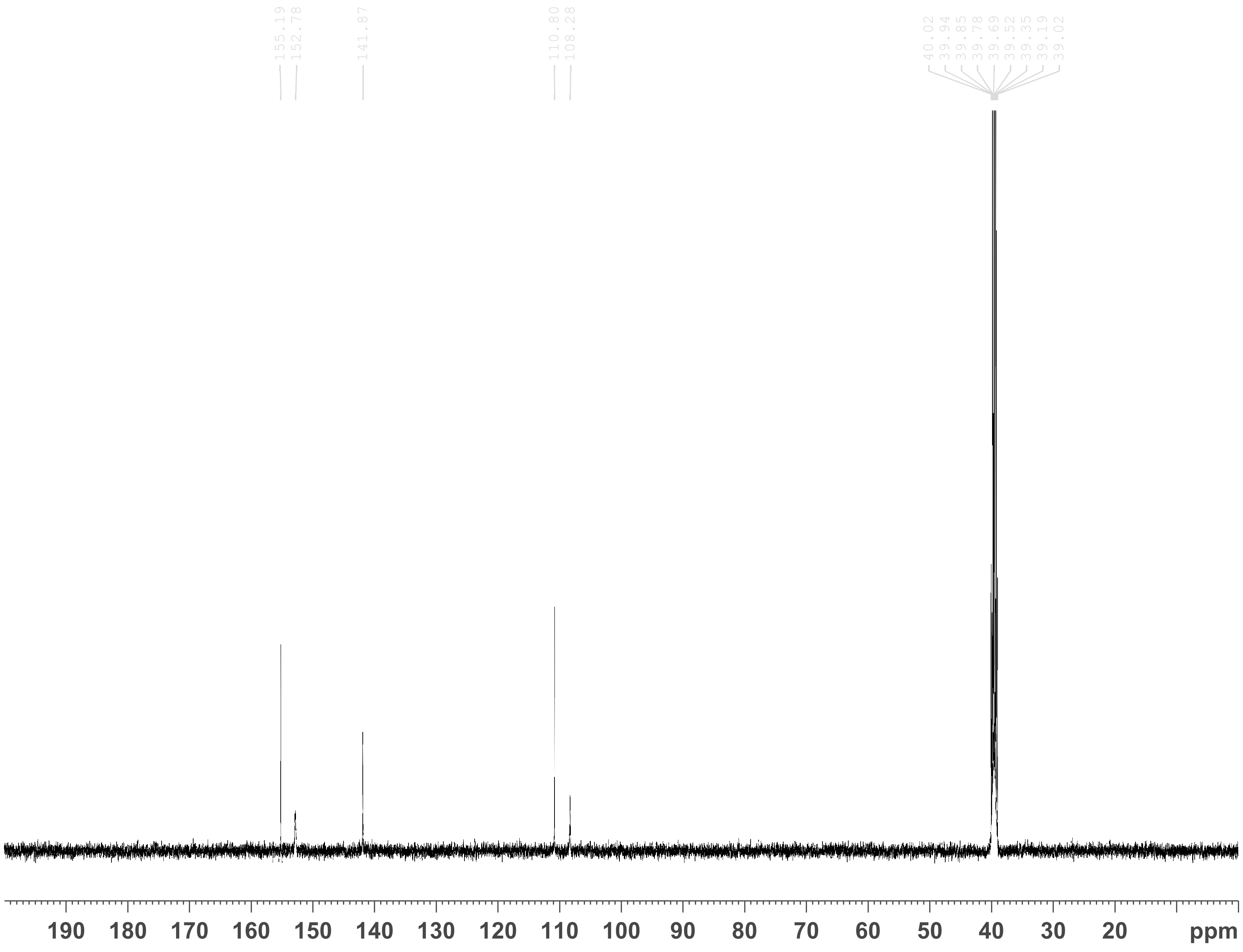


```
Current Data Parameters
NAME     IG-JM-200398-016-00
EXPNO                      2
PROCNO                     1

F2 - Acquisition Parameters
Date_               20161103
Time                   15.41
INSTRUM                spect
PROBHD   5 mm QNP 1H/13
PULPROG              zgpg30
TD                     65536
SOLVENT                 DMSO
NS                       256
DS                         4
SWH              29761.904 Hz
FIDRES            0.454131 Hz
AQ               1.1010048 sec
RG                      2050
DW                    16.800 usec
DE                      6.50 usec
TE                     298.2 K
D1                2.00000000 sec
D11               0.03000000 sec
TD0                        1

======== CHANNEL f1 ========
SFO1          125.7703637 MHz
NUC1                     13C
P1                      7.50 usec
PLW1          92.00000000 W

======== CHANNEL f2 ========
SFO2          500.1320005 MHz
NUC2                      1H
CPDPRG[2             waltz16
PCPD2                  80.00 usec
PLW2          25.00000000 W
PLW12          0.39063001 W
PLW13          0.25000000 W

F2 - Processing parameters
SI                     32768
SF            125.7578475 MHz
WDW                       EM
SSB      0
LB                      1.00 Hz
GB       0
PC                      1.40
```

Compound 11

PROTON.d DMSO {C:\Bruker\TopSpin3.2} IG500 11

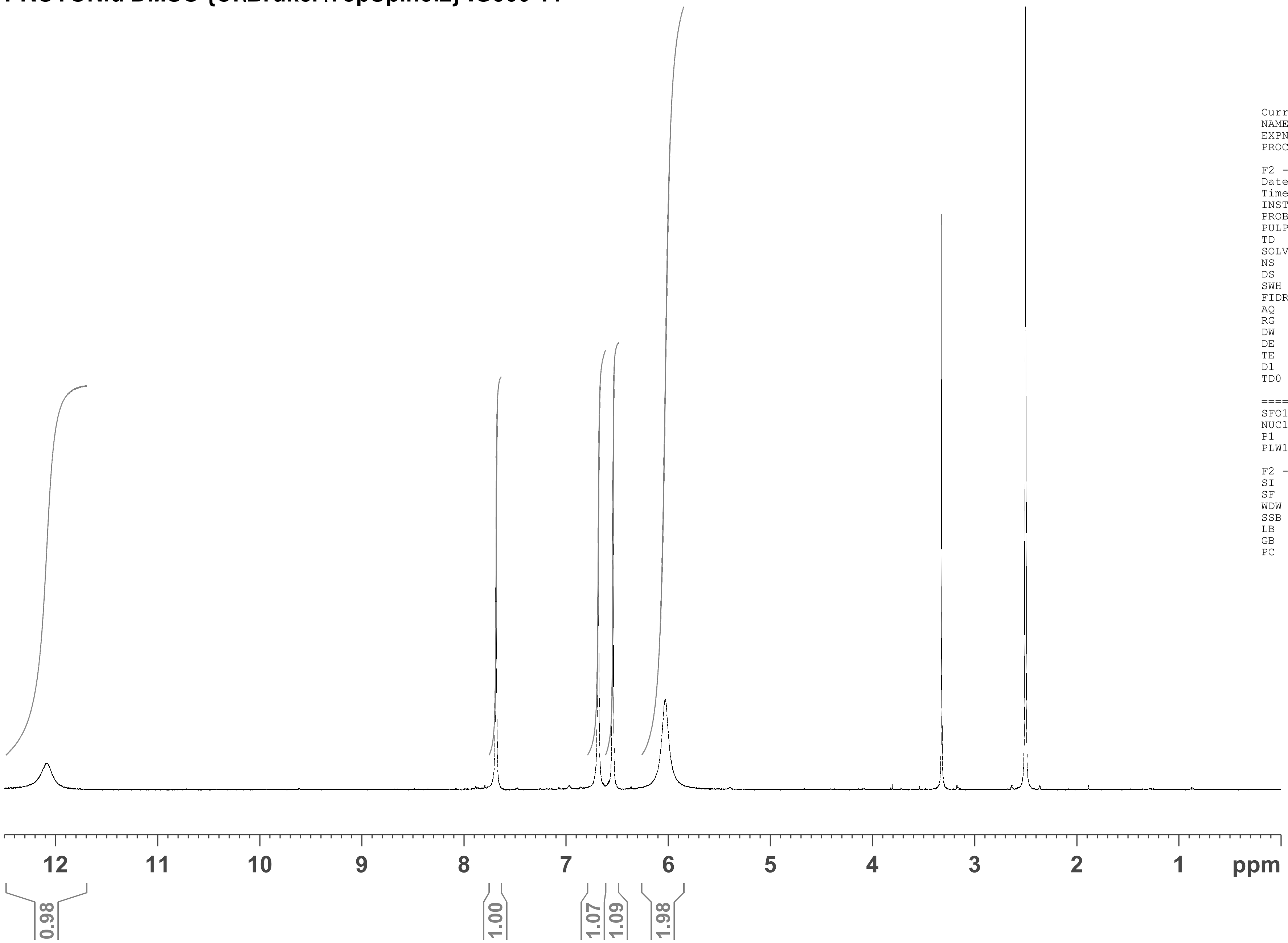


```
Current Data Parameters
NAME     IG-JM-200398-076-001
EXPNO                       1
PROCNO                      1

F2 - Acquisition Parameters
Date_            20170301
Time                12.06
INSTRUM             spect
PROBHD   5 mm QNP 1H/13
PULPROG              zg30
TD                  65536
SOLVENT              DMSO
NS                     16
DS                      2
SWH             10000.000 Hz
FIDRES           0.152588 Hz
AQ              3.2767999 sec
RG                    456
DW                 50.000 usec
DE                   6.50 usec
TE                  298.2 K
D1             1.00000000 sec
TD0                     1

======== CHANNEL f1 ========
SFO1          500.1330885 MHz
NUC1                   1H
P1                  10.00 usec
PLW1          25.00000000 W

F2 - Processing parameters
SI                  65536
SF            500.1300046 MHz
WDW                    EM
SSB      0
LB                   0.30 Hz
GB       0
PC                   1.00
```

Compound 14

PROTON.d MeOD {C:\Bruker\TopSpin3.2} IG500 17

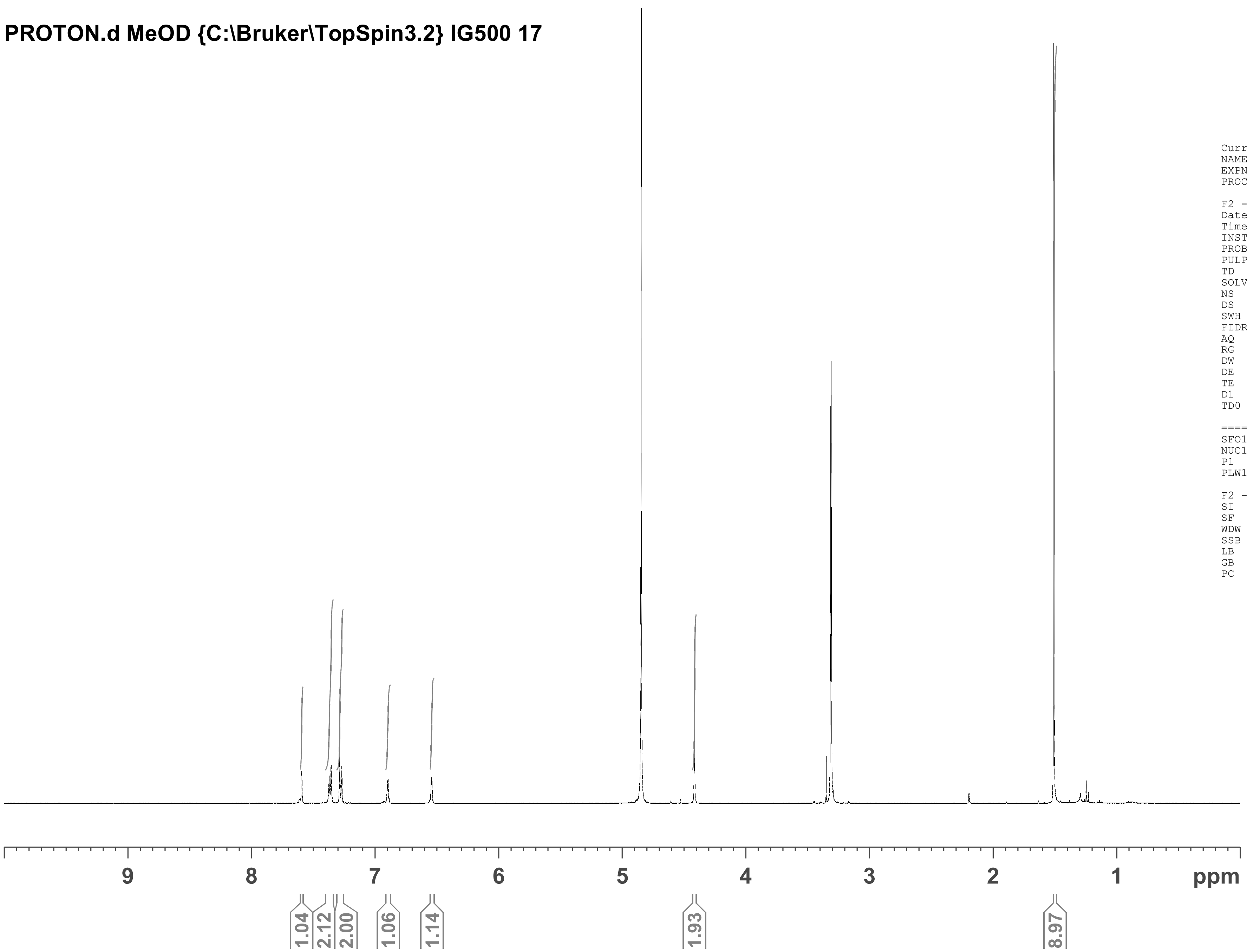


```
Current Data Parameters
NAME     IG-JM-200398-039-001
EXPNO                   1
PROCNO                  1

F2 - Acquisition Parameters
Date_            20161201
Time                16.02
INSTRUM             spect
PROBHD   5 mm QNP 1H/13
PULPROG              zg30
TD                  65536
SOLVENT              MeOD
NS                     16
DS                      2
SWH             10000.000 Hz
FIDRES           0.152588 Hz
AQ              3.2767999 sec
RG                    512
DW                 50.000 usec
DE                   6.50 usec
TE                  298.2 K
D1             1.00000000 sec
TD0                     1

======== CHANNEL f1 ========
SFO1          500.1330885 MHz
NUC1                   1H
P1                  10.00 usec
PLW1          25.00000000 W

F2 - Processing parameters
SI                  65536
SF            500.1299920 MHz
WDW                    EM
SSB      0
LB                   0.30 Hz
GB       0
PC                   1.00
```

Compound 15

Analytical Studio Reviewer Report

Sample Name: JM-200398-039-T38
Location: 1,1:D,4
Acquired: 30-Nov-16 6:12 PM
Filename: JM-200398-039-T38_005765.D
Instrument: LCMS 6130A

Compound 15

Peak #	Time	Area % TIC(+)	Area % UV254	BPM
1	1.405	87.8	100.0	356.2
2	2.226	12.2	0.0	282.3

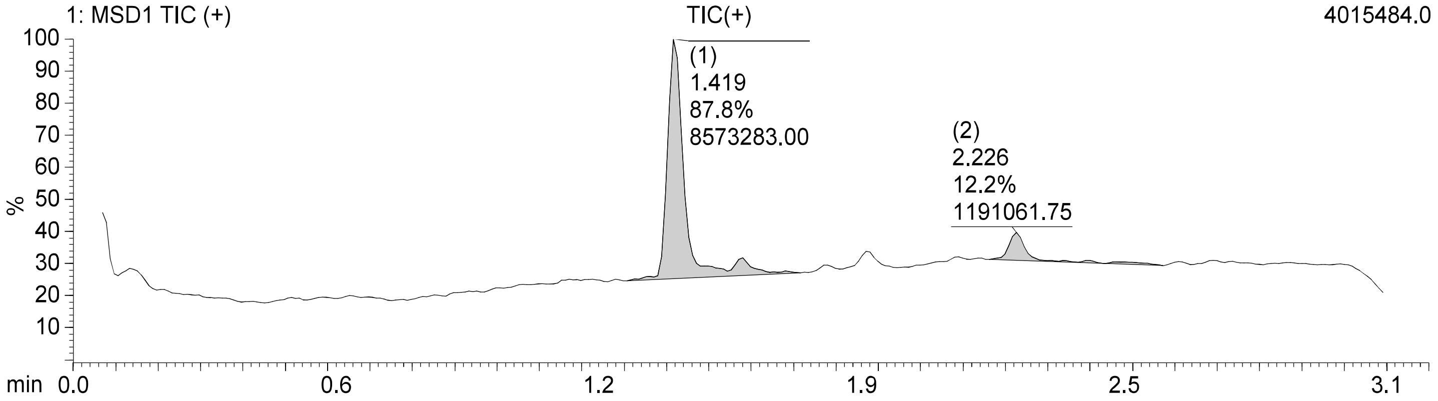

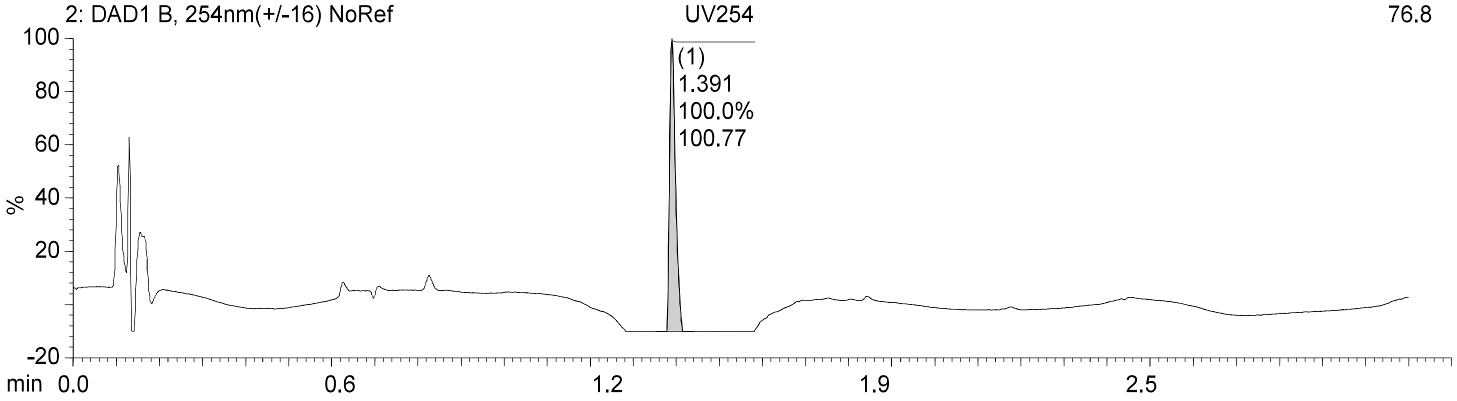

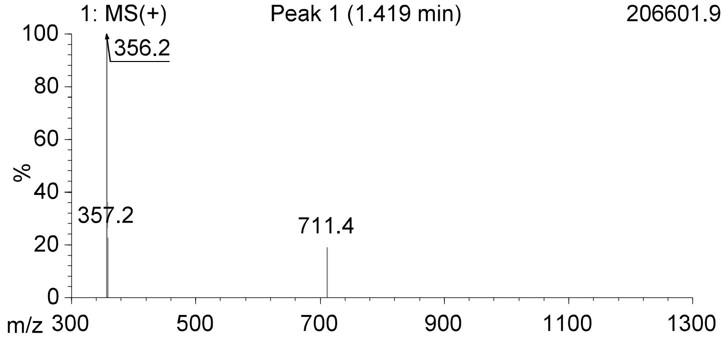

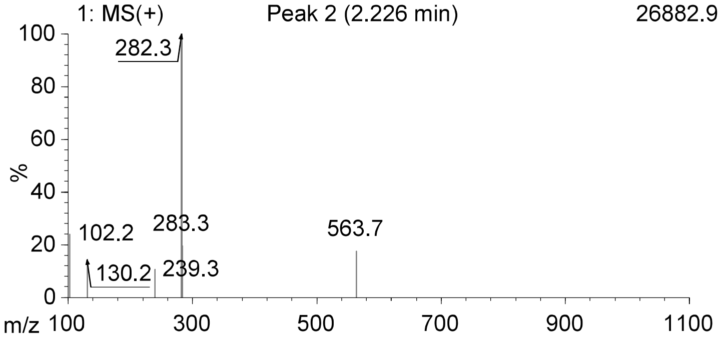

Printed: 30-Nov-16 6:16 PM

PROTON.d MeOD {C:\Bruker\TopSpin3.2} IG500 32

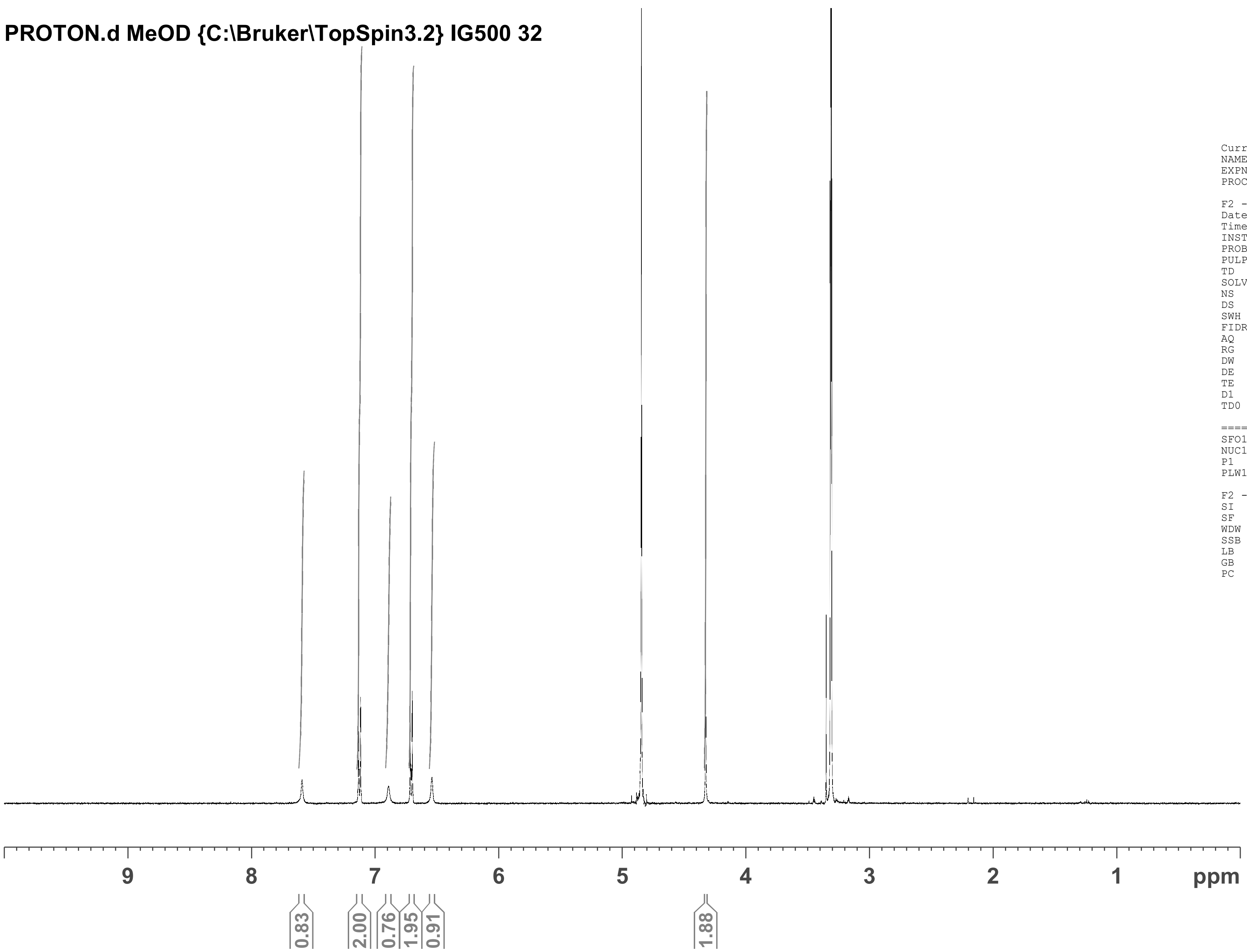


```
Current Data Parameters
NAME     IG-JM-200398-043-001
EXPNO                    1
PROCNO                   1

F2 - Acquisition Parameters
Date_          20161208
Time              14.26
INSTRUM           spect
PROBHD   5 mm QNP 1H/13
PULPROG            zg30
TD                65536
SOLVENT            MeOD
NS                   16
DS                    2
SWH           10000.000 Hz
FIDRES         0.152588 Hz
AQ            3.2767999 sec
RG                  645
DW               50.000 usec
DE                 6.50 usec
TE                298.2 K
D1           1.00000000 sec
TD0                   1

======== CHANNEL f1 ========
SFO1        500.1330885 MHz
NUC1                 1H
P1                10.00 usec
PLW1        25.00000000 W

F2 - Processing parameters
SI                65536
SF          500.1299944 MHz
WDW                  EM
SSB      0
LB                 0.30 Hz
GB       0
PC                 1.00
```

Compound 16

Compound 16

Compass OpenAccess LC-MS Identification Report

Sample-ID	200398-043-001	Station	Microtof-2
Submitter	James Martin	Supervisor	System Administrator
Analysis Name	200398-043-001_10254_RD8_01_11159.d	Acquisition Date	09/01/2017 17:14:47
Sample Description		Method	1-microtof-2 Identify Compounds LCMS Pos 5-95.m

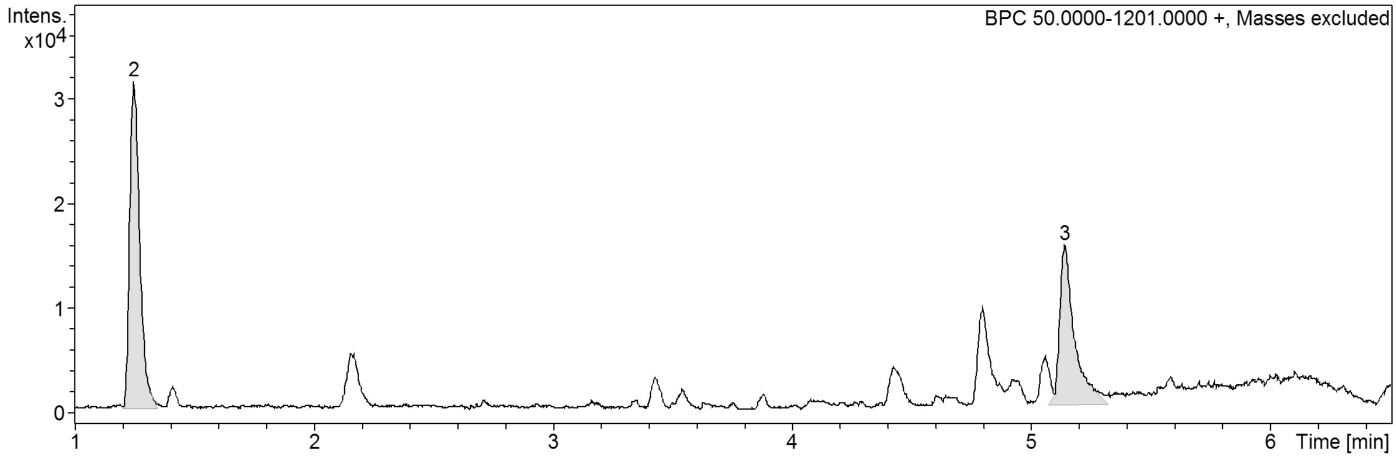

Sample Description

Method 1-microtof-2 Identify Compounds LCMS Pos 5-95.m

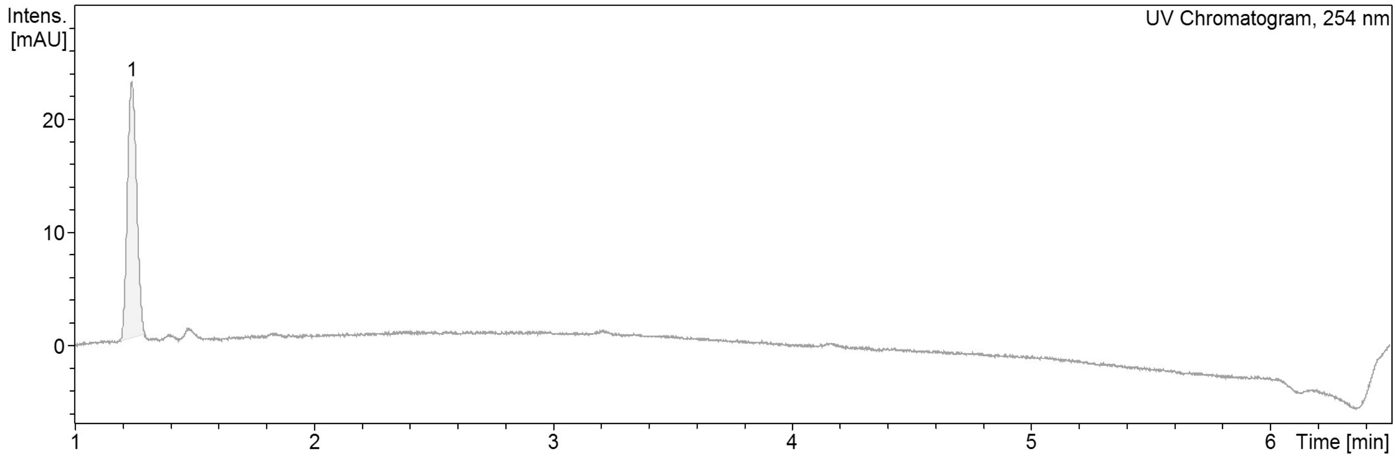

#	RT [min]	Area Frac. %	Chromatogram
1	1.2	100.00	UV Chromatogram, 254 nm
2	1.2	58.12	BPC 50.0000-1201.0000 +, Masses excluded
3	5.1	41.88	BPC 50.0000-1201.0000 +, Masses excluded

SmartFormula Settings

Tolerance	mSigma Limit	Electron Conf.
20 ppm	60	even

Adduction(s): **Neutral Loss(es):**

Compass OpenAccess LC-MS Identification Report

SmartFormula Results

FormulaMin: C1H1 **FormulaMax:** Na

#	meas. m/z	theo. m/z	|err| [ppm]	mSigma	Formula	Purity(UVC)[%]	Purity(BPC)[%]
2	256.1205	256.1193	4.6	3	C13 H14 N5 O	100.0	58.1
		256.1169	14.0	15	C11 H15 N5 Na O		
		511.2217	2.5	4	C30 H28 N6 Na O		
		511.2329	19.5	4	C29 H28 N8 Na		
3	283.2836	283.2843	2.5	35	C15 H39 O4	0.0	41.9
		283.2832	1.2	36	C14 H36 N4 Na		
		563.5299	8.2	3	C39 H67 N2		
		563.5275	12.4	10	C37 H68 N2 Na		

Note: mSigma values <30 indicate high probability of correct molecular formula

Cmpd 1, 1.2 min

Undefined context.
Component not placed in a spectrum iterator.

Cmpd 2, 1.2 min

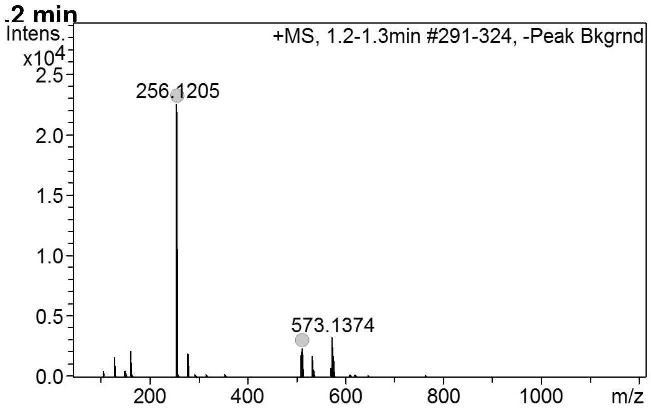

Cmpd 3, 5.1 min

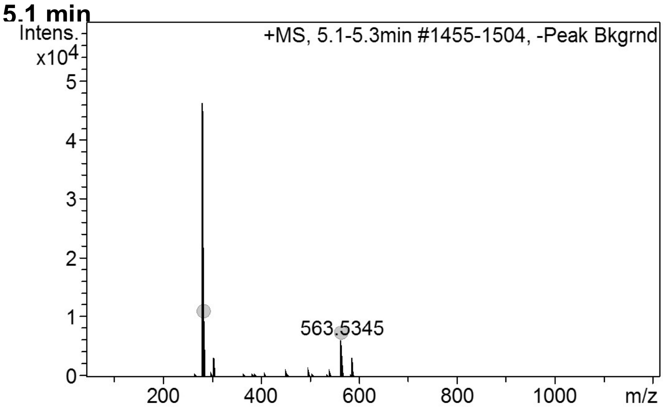

PROTON256 DMSO {C:\Bruker\TopSpin3.2} IG500 5

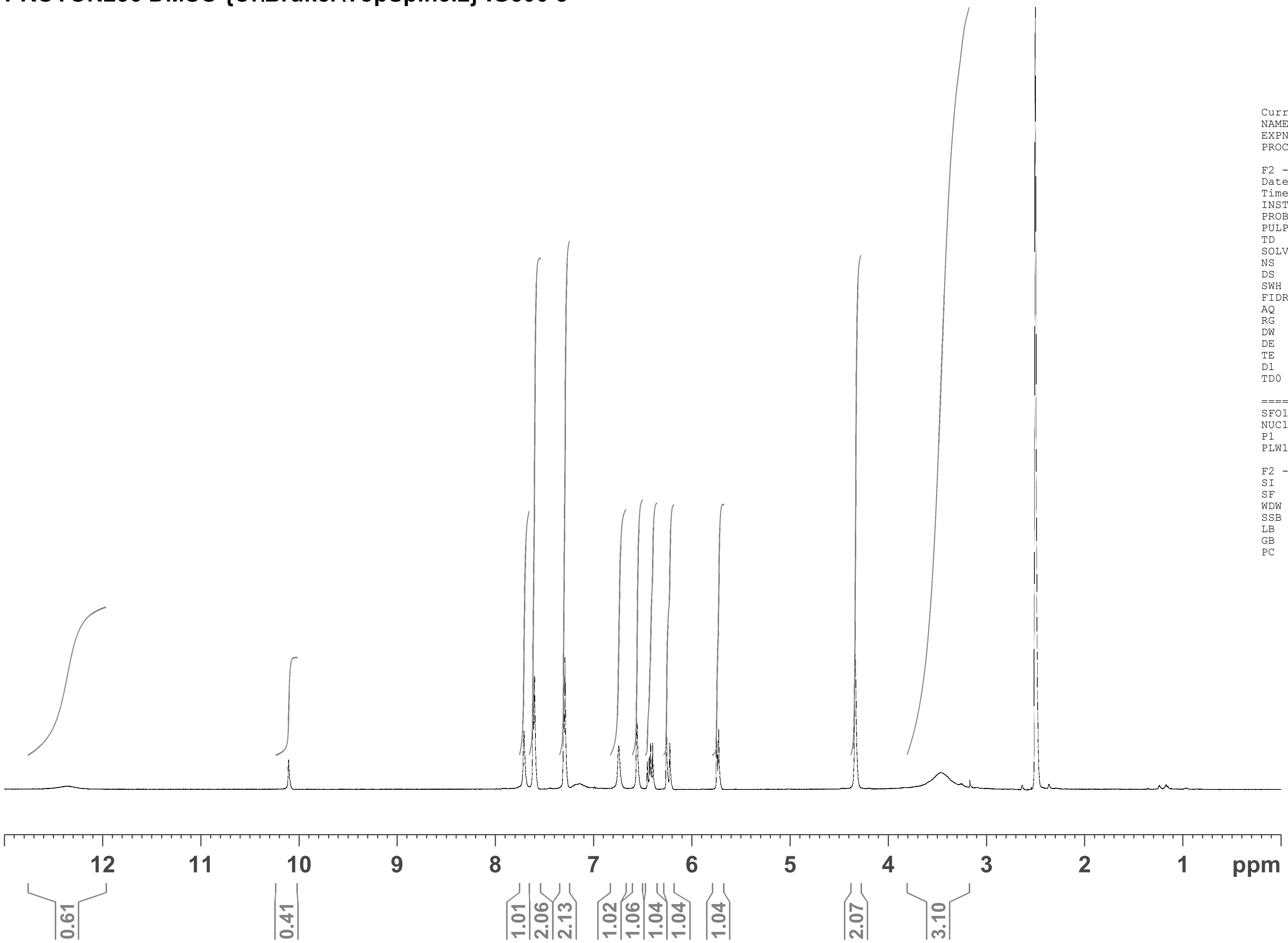


```
Current Data Parameters
NAME     IG-JM-200398-044-001
EXPNO                  1
PROCNO                 1

F2 - Acquisition Parameters
Date_          20161213
Time              13.16
INSTRUM           spect
PROBHD   5 mm QNP 1H/13
PULPROG            zg30
TD                65536
SOLVENT            DMSO
NS                  256
DS                    2
SWH           10000.000 Hz
FIDRES         0.152588 Hz
AQ            3.2767999 sec
RG                   32
DW               50.000 usec
DE                 6.50 usec
TE                298.2 K
D1           1.00000000 sec
TD0                   1

======== CHANNEL f1 ========
SFO1        500.1330885 MHz
NUC1                 1H
P1                10.00 usec
PLW1        25.00000000 W

F2 - Processing parameters
SI                65536
SF          500.1303954 MHz
WDW                  EM
SSB      0
LB                 0.30 Hz
GB       0
PC                 1.00
```

Compound 17

C13CPD6.d DMSO {C:\Bruker\TopSpin3.2} IG500 20

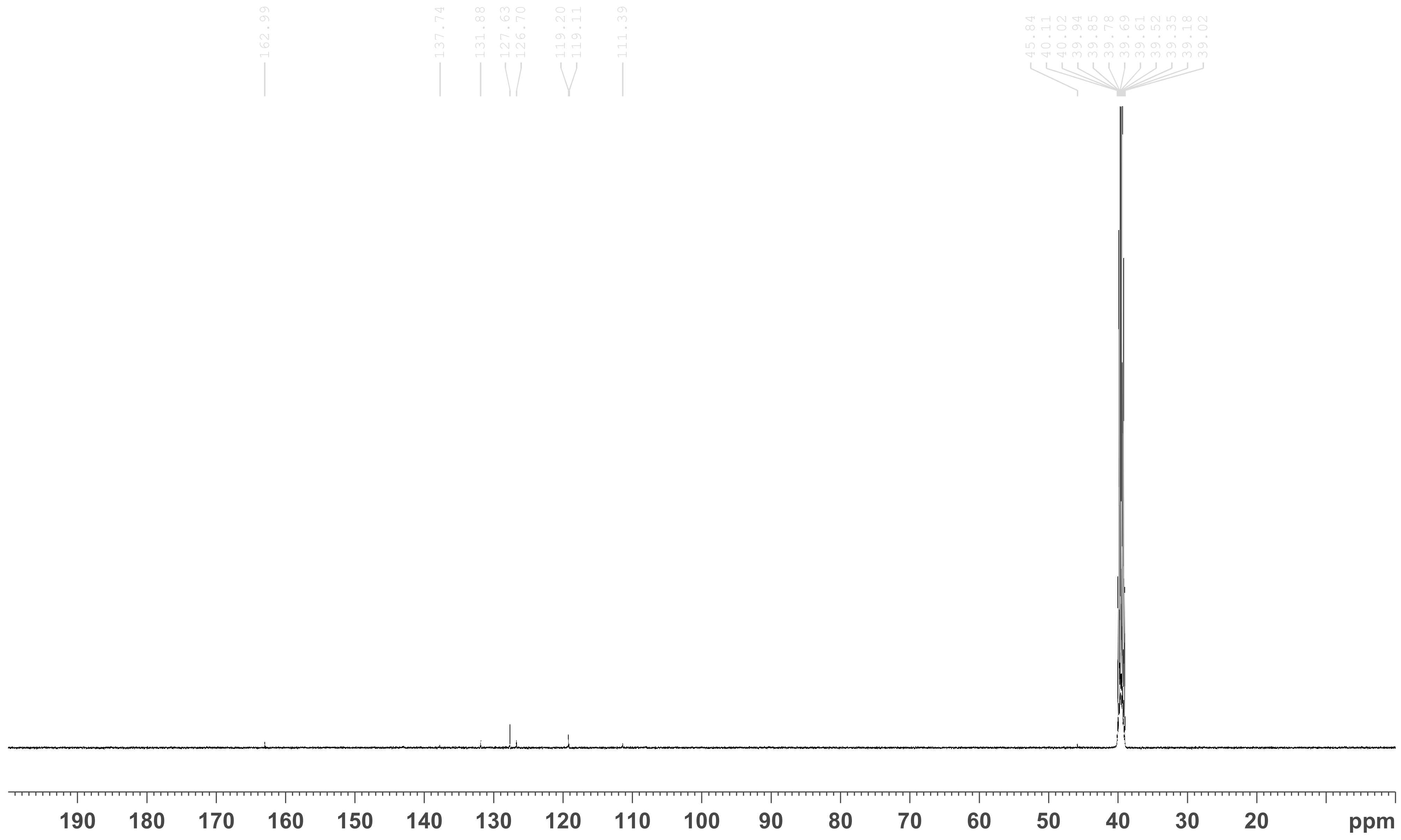

Compound 17

Compound 17

Compound Verification Report (Compass OpenAccess/QC)

Sample-ID	200398-044-001	Station	Microtof-2
Submitter	James Martin	Supervisor	System Administrator
Analysis Name	200398-044-001_10230_RA8_01_11130.d	Acquisition Date	09/01/2017 10:13:32
Sample Description		Method	2-microtof-2 verify compounds lcms pos 5-95.m

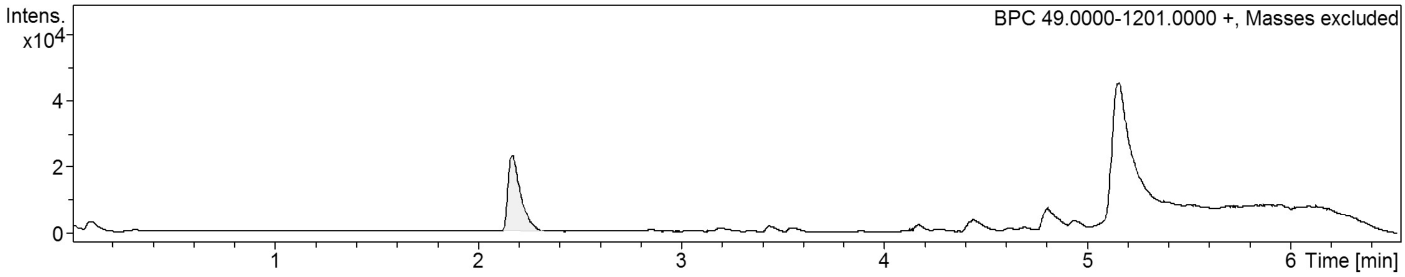

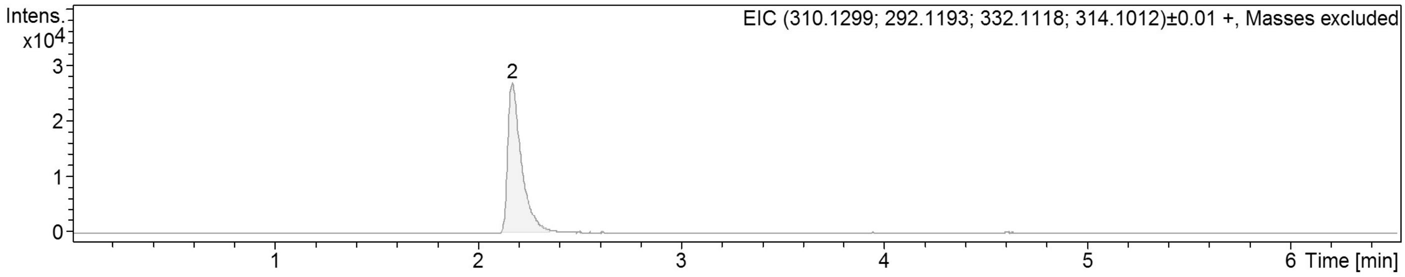

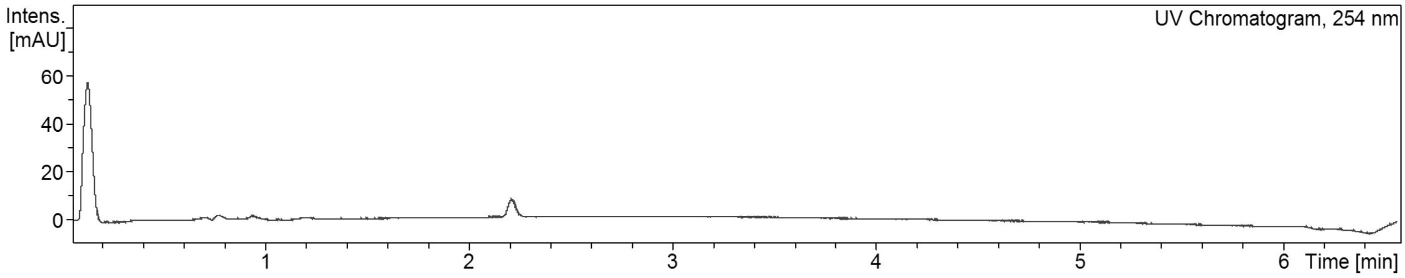

SmartFormula Settings

Tolerance	mSigma Limit	Electron Conf.
10 ppm	60	even

Adduction(s): H, Na **Neutral Loss(es):** H2O

Compound Verification Results

Expected Formula: C16H15N5O2

#	meas. m/z	theo. m/z	|err| [ppm]	mSigma	Formula	Modification	Purity(UVC)[%]	Purity(BPC)[%]
2	310.1308	310.1299	2.9	2	C16 H16 N5 O2	(M+H)+	0.0	100.0

Note: mSigma values <20 indicate high probability of correct molecular formula

Compound Verification Report (Compass OpenAccess/QC)

Cmpd 2, 2.2 min

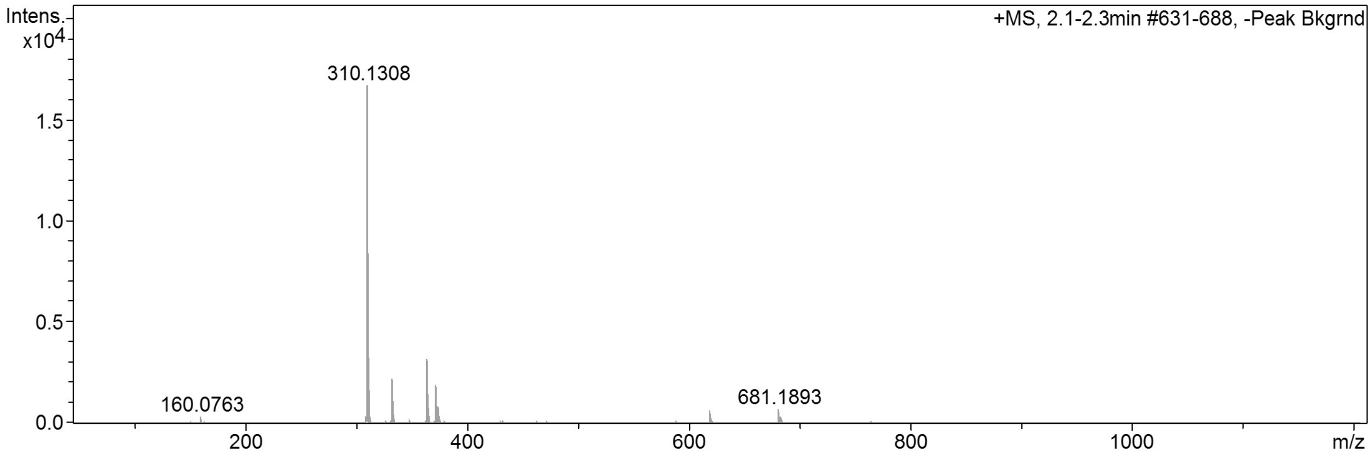

Analysis Name D:\Data\james\200398-044-001_10230_RA8_01_11130.d

Compass OpenAccess LC-MS Identification Report

Sample-ID	200398-044-001	Station	Microtof-2
Submitter	James Martin	Supervisor	System Administrator
Analysis Name	200398-044-001_10236_RB6_01_11136.d	Acquisition Date	09/01/2017 11:08:20
Sample Description		Method	3-microtof-2 Identify Compounds BASIC LCMS Pos 5-95.m

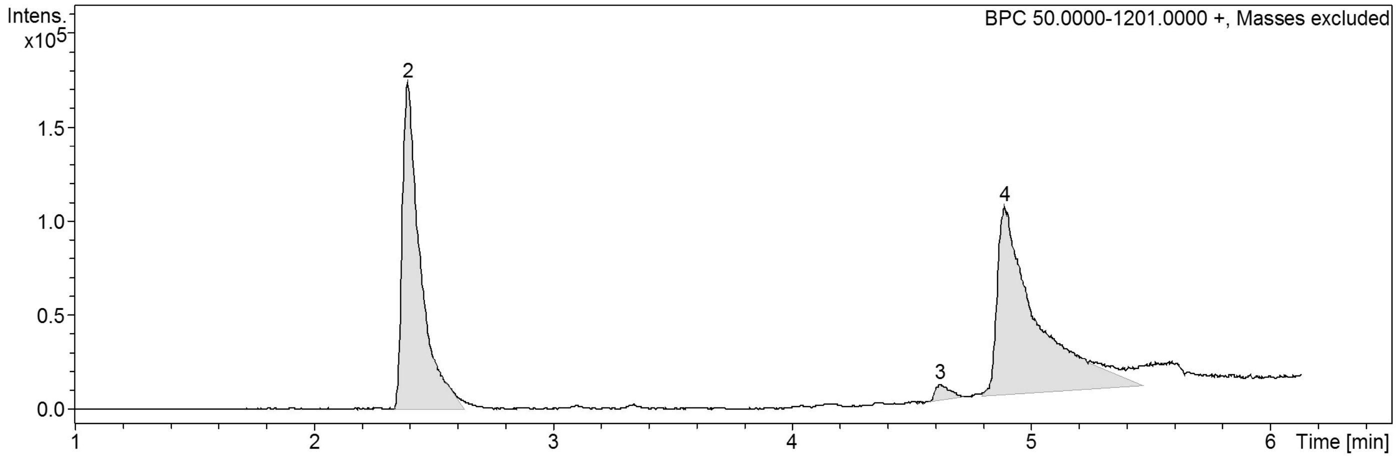

Sample Description

Method 3-microtof-2 Identify Compounds BASIC LCMS Pos 5-95.m

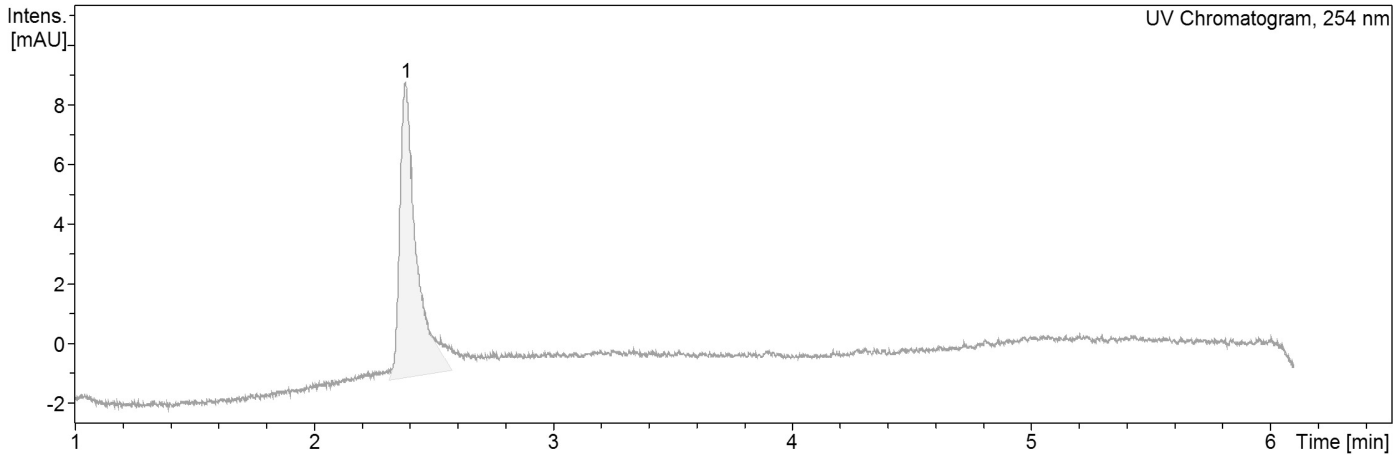

#	RT [min]	Area Frac. %	Chromatogram
1	2.4	100.000	UV Chromatogram, 254 nm
2	2.4	43.628	BPC 50.0000-1201.0000 +, Masses excluded
3	4.6	1.745	BPC 50.0000-1201.0000 +, Masses excluded
4	4.9	54.627	BPC 50.0000-1201.0000 +, Masses excluded

SmartFormula Settings

Compass OpenAccess LC-MS Identification Report

Tolerance	mSigma Limit	Electron Conf.
10 ppm	60	even

Adduction(s): **Neutral Loss(es):**

SmartFormula Results

FormulaMin: C1H1 **FormulaMax:** Na

#	meas. m/z	theo. m/z	|err| [ppm]	mSigma	Formula	Purity(UVC)[%]	Purity(BPC)[%]
2	310.1299	310.1299	0.2	6	C16 H16 N5 O2	100.0	43.6
		310.1315	5.0	8	C19 H17 N3 Na		
		619.2511	6.7	4	C31 H35 N6 O8		
		619.2500	8.5	4	C30 H32 N10 Na O4		
3	280.2634	280.2635	0.4	18	C18 H34 N O	0.0	1.7
		280.2611	8.1	27	C16 H35 N Na O		
		438.3762	8.9	22	C19 H48 N7 O4		
		438.3765	8.2	24	C21 H53 N Na O6		
		482.4105	0.9	31	C31 H52 N3 O		
		482.4081	5.8	34	C29 H53 N3 Na O		
4	282.2790	282.2767	8.2	12	C16 H37 N Na O	0.0	54.6
		282.2791	0.3	14	C18 H36 N O		
		563.5510	4.1	7	C36 H71 N2 O2		
		563.5486	8.4	11	C34 H72 N2 Na O2		

Note: mSigma values <30 indicate high probability of correct molecular formula

Cmpd 1, 2.4 min

Undefined context.
Component not placed in a spectrum iterator.

Cmpd 2, 2.4 min

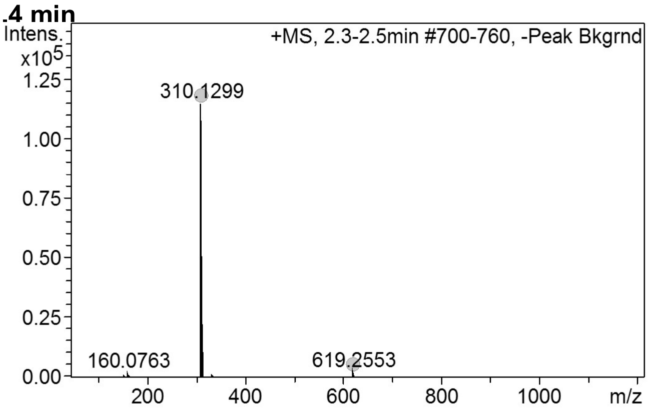

Cmpd 3, 4.6 min

Cmpd 4, 4.9 min

Compass OpenAccess LC-MS Identification Report

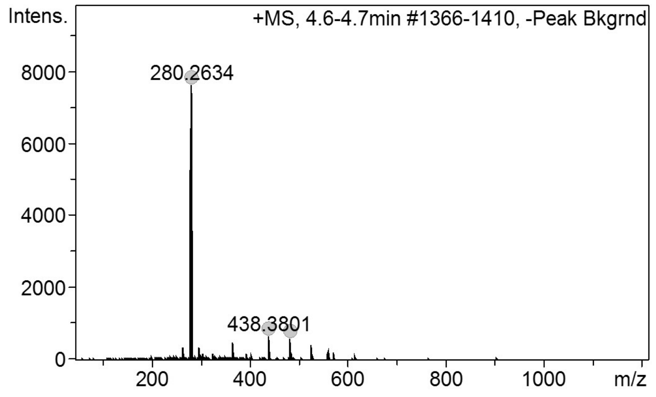

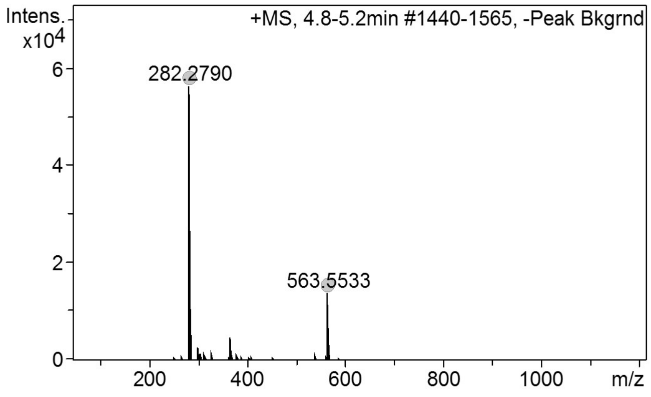

Analysis Name D:\Data\james\200398-044-001_10236_RB6_01_11136.d

PROTON.d MeOD {C:\Bruker\TopSpin3.2} IG500 23

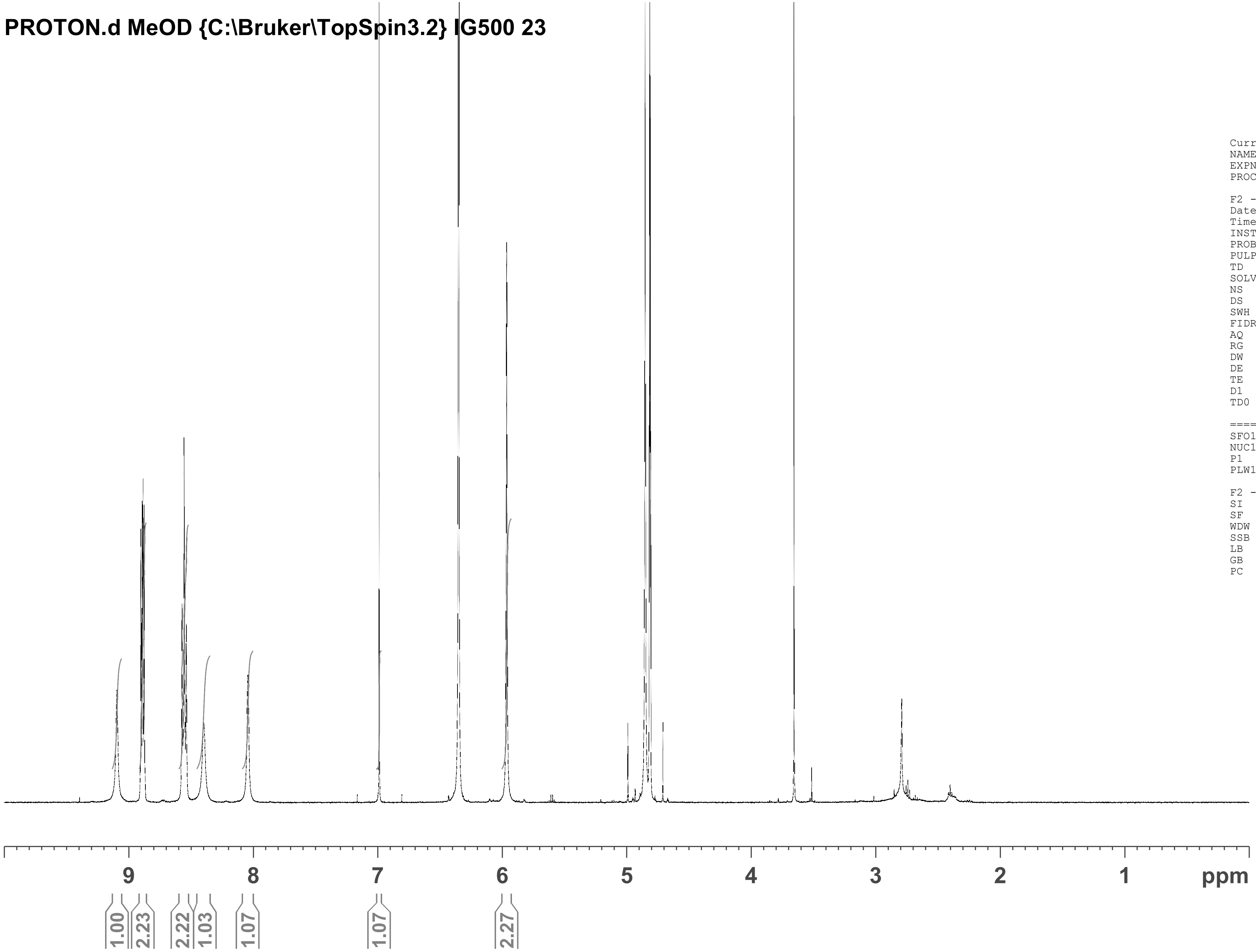

Current Data Parameters
NAME IG-JM-200398-003-001
EXPNO 1
PROCNO 1

F2 - Acquisition Parameters
Date_ 20160922
Time 17.00
INSTRUM spect
PROBHD 5 mm QNP 1H/13
PULPROG zg30
TD 65536
SOLVENT MeOD
NS 16
DS 2
SWH 10000.000 Hz
FIDRES 0.152588 Hz
AQ 3.2767999 sec
RG 362
DW 50.000 usec
DE 6.50 usec
TE 298.2 K
D1 1.00000000 sec
TD0 1

======== CHANNEL f1 ========
SFO1 500.1330885 MHz
NUC1 1H
P1 10.00 usec
PLW1 25.00000000 W

F2 - Processing parameters
SI 65536
SF 500.1300000 MHz
WDW EM
SSB 0
LB 0.30 Hz
GB 0
PC 1.00

Compound 18

Compound Verification Report (Compass OpenAccess/QC)

Sample-ID	200398-003-001	Station	Microtof-2
Submitter	James Martin	Supervisor	System Administrator
Analysis Name	200398-003-001_8752_RC5_01_9371.d	Acquisition Date	31/10/2016 17:04:12
Sample Description		Method	5-LC-HRMS-ISOCRATIC-GE8.m

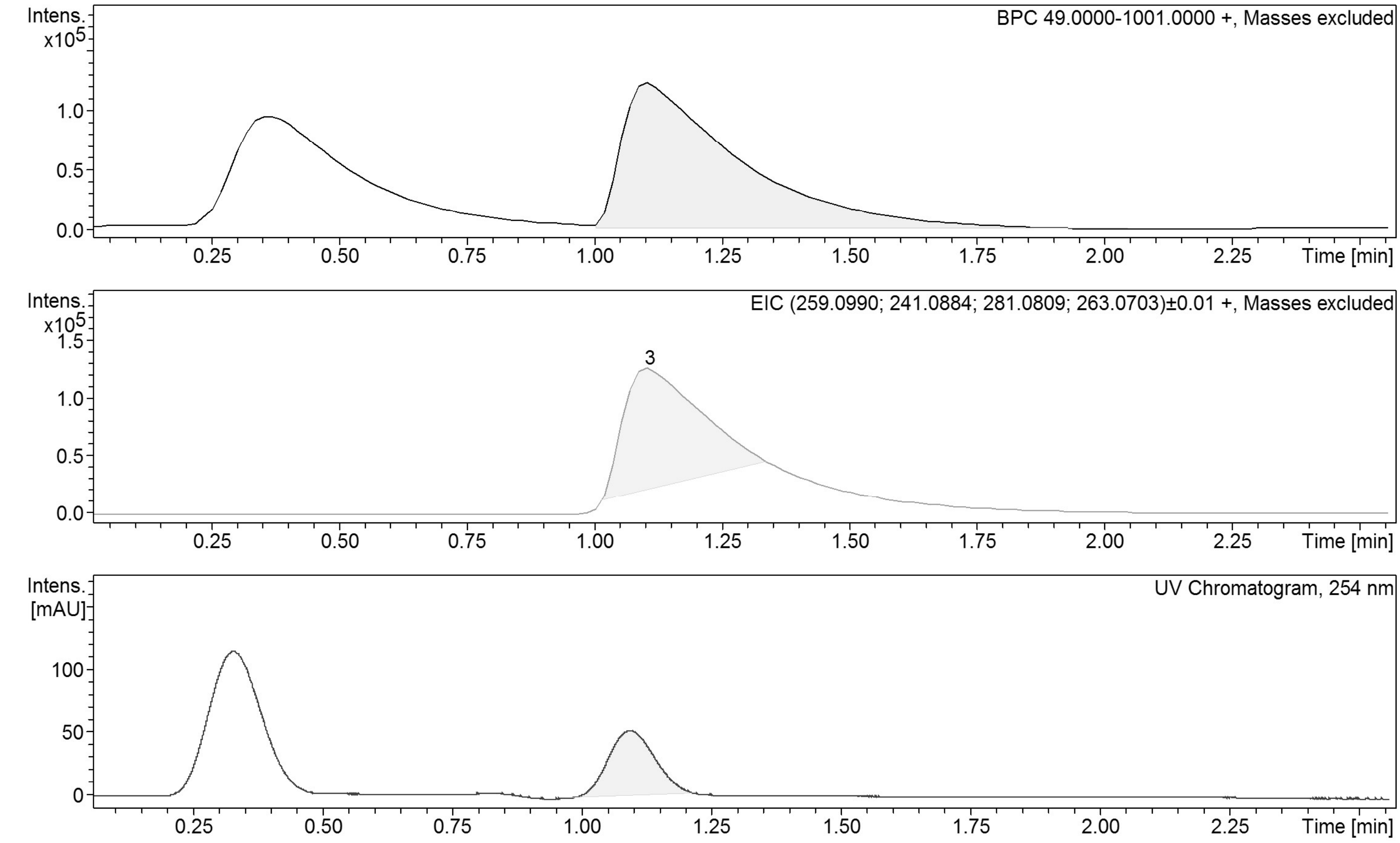

SmartFormula Settings

Tolerance	mSigma Limit	Electron Conf.
10 ppm	60	even

Adduction(s): H, Na **Neutral Loss(es):** H2O

Compound Verification Results

Expected Formula: C13H11FN4O

#	meas. m/z	theo. m/z	|err| [ppm]	mSigma	Formula	Modification	Purity(UVC)[%]	Purity(BPC)[%]
3	259.0984	259.0990	2.4	34	C13 H12 F N4 O	(M+H)+	100.0	100.0

Note: mSigma values <20 indicate high probability of correct molecular formula

Compound Verification Report (Compass OpenAccess/QC)

Cmpd 3, 1.1 min

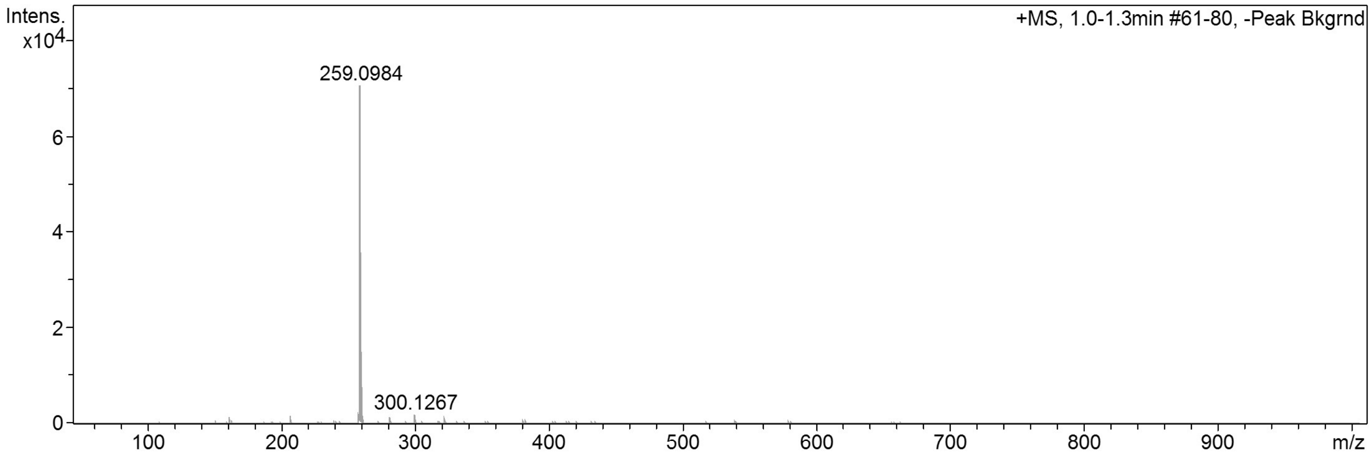

Analysis Name D:\Data\james\200398-003-001_8752_RC5_01_9371.d

Compass OpenAccess LC-MS Identification Report

Sample-ID	200398-003-001	Station	Microtof-2
Submitter	James Martin	Supervisor	System Administrator
Analysis Name	200398-003-001_8761_RD6_01_9381.d	Acquisition Date	01/11/2016 10:04:04
Sample Description		Method	1-microtof-2 Identify Compounds LCMS Pos 5-95.m

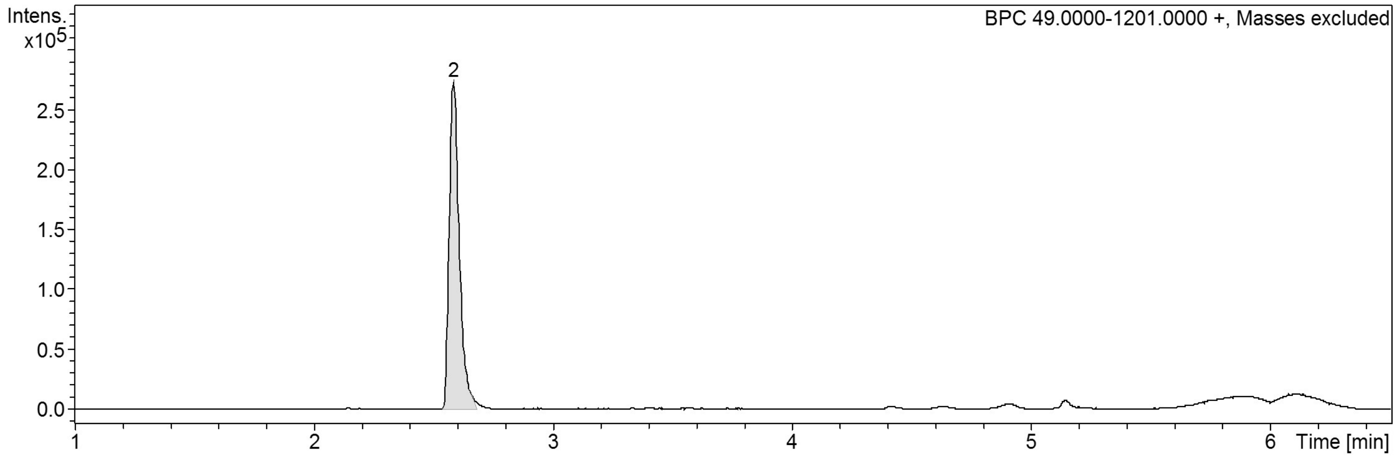

Sample Description

Method 1-microtof-2 Identify Compounds LCMS Pos 5-95.m

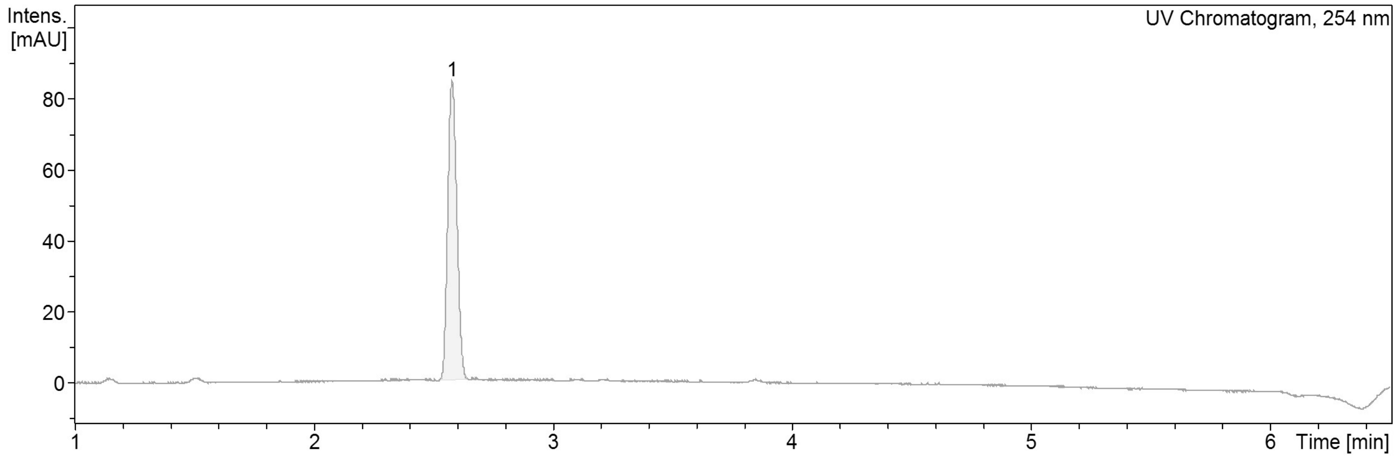

#	RT [min]	Area Frac. %	Chromatogram
1	2.6	100.00	UV Chromatogram, 254 nm
2	2.6	100.00	BPC 49.0000-1201.0000 +, Masses excluded

SmartFormula Settings

Tolerance	mSigma Limit	Electron Conf.
20 ppm	60	even

Adduction(s):

Neutral Loss(es):

Compass OpenAccess LC-MS Identification Report

SmartFormula Results

FormulaMin: C13H12FN4O **FormulaMax:** Na

#	meas. m/z	theo. m/z	|err| [ppm]	mSigma	Formula	Purity(UVC)[%]	Purity(BPC)[%]
2	539.1707	539.1611	17.8	1	C25 H17 F2 N12 O	100.0	100.0
		539.1613	17.3	1	C27 H20 F5 N6 O		

Note: mSigma values <30 indicate high probability of correct molecular formula

Cmpd 1, 2.6 min

Undefined context.
Component not placed in a spectrum iterator.

Cmpd 2, 2.6 min

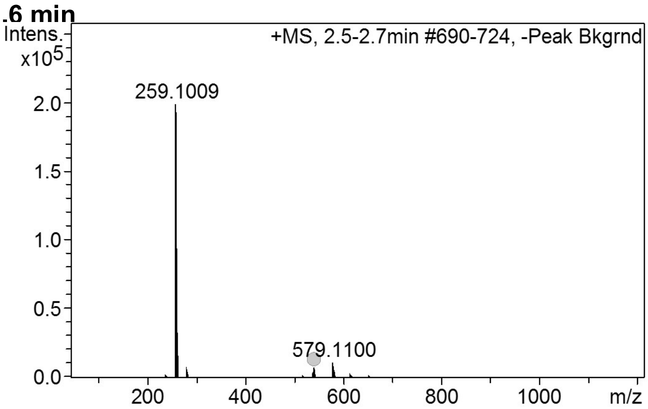

Compound 19

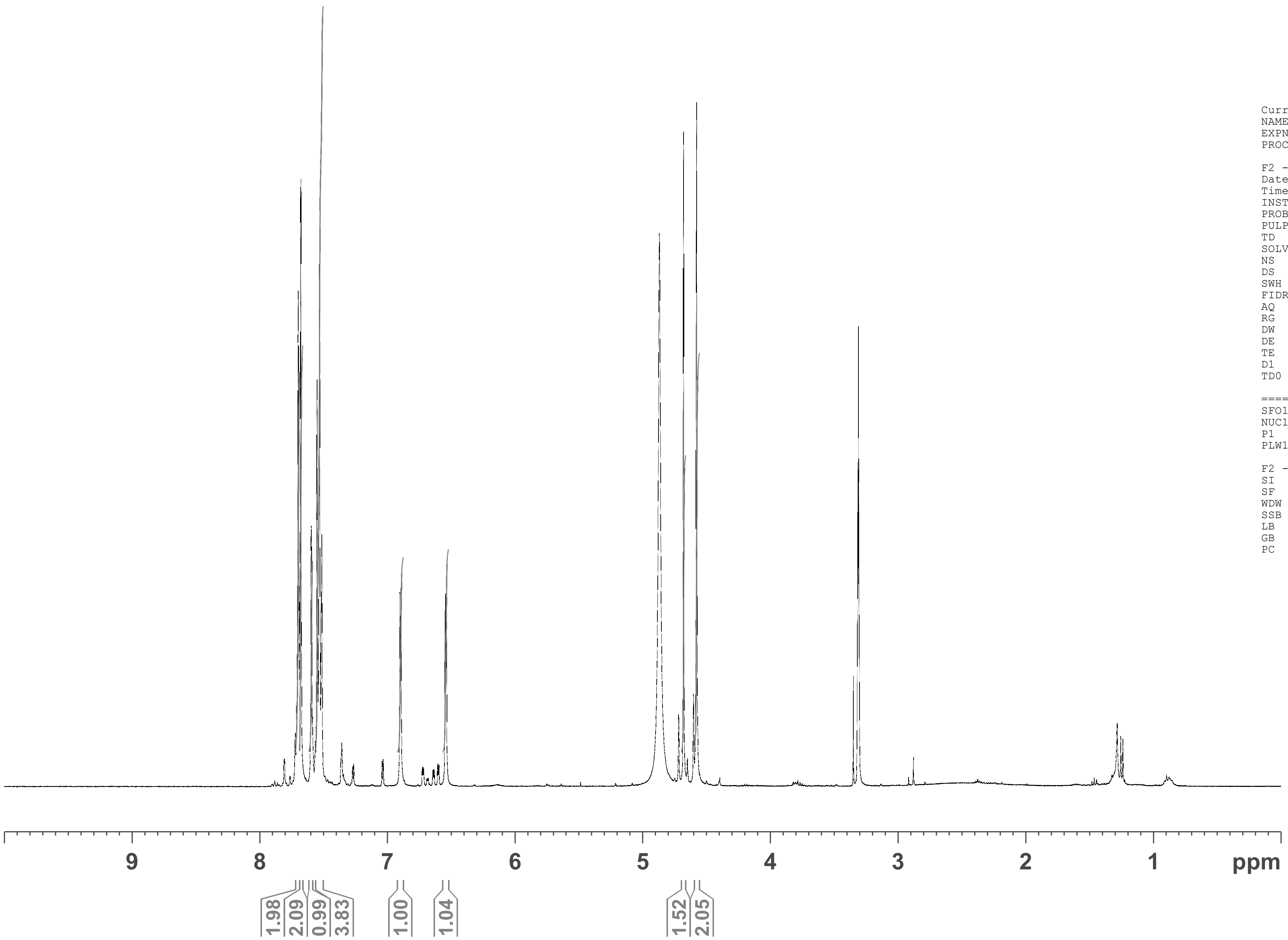


```
Current Data Parameters
NAME     IG-JM-200398-019-001
EXPNO                       1
PROCNO                      1

F2 - Acquisition Parameters
Date_            20161111
Time                10.25
INSTRUM             spect
PROBHD   5 mm PADUL 13C
PULPROG              zg30
TD                 131072
SOLVENT              MeOD
NS                     16
DS                      4
SWH           12019.230 Hz
FIDRES         0.091699 Hz
AQ            5.4525952 sec
RG               127.77
DW               41.600 usec
DE                12.17 usec
TE                298.1 K
D1           0.10000000 sec
TD0                     1

======== CHANNEL f1 ========
SFO1        400.1324710 MHz
NUC1                 1H
P1                10.00 usec
PLW1        20.00000000 W

F2 - Processing parameters
SI                 131072
SF          400.1300078 MHz
WDW                    EM
SSB      0
LB                  0.10 Hz
GB       0
PC                  1.00
```

Compound 19

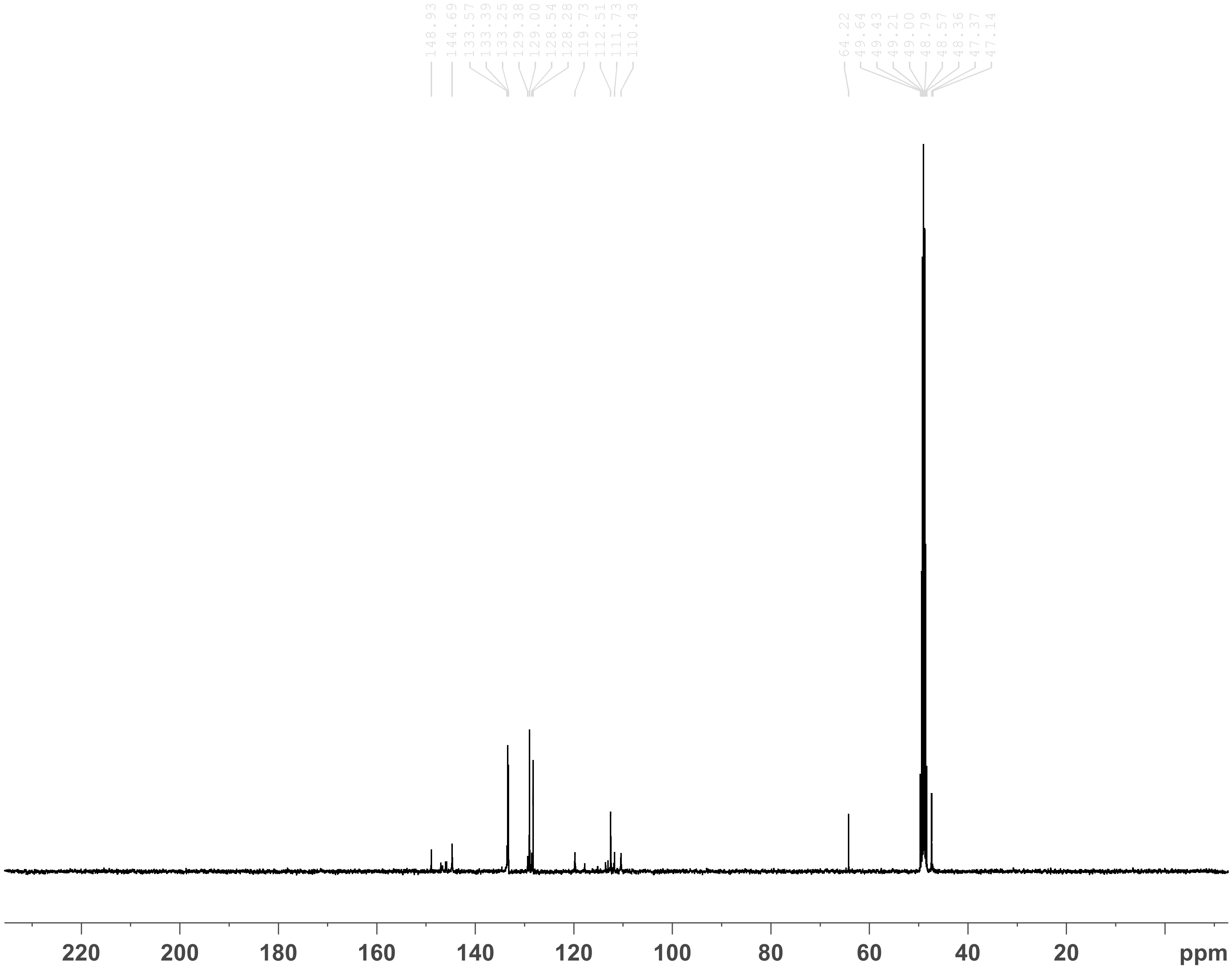


```
Current Data Parameters
NAME     IG-JM-200398-019-001
EXPNO                   3
PROCNO                  1

F2 - Acquisition Parameters
Date_            20161111
Time                12.23
INSTRUM             spect
PROBHD   5 mm PADUL 13C
PULPROG             udeft
TD                  17996
SOLVENT              MeOD
NS                    256
DS                      0
SWH             25000.000 Hz
FIDRES           1.389198 Hz
AQ              0.3599200 sec
RG                 196.14
DW                 20.000 usec
DE                   8.66 usec
TE                  298.2 K
D1             3.00000000 sec
D11            0.03000000 sec
D12            0.00002000 sec
D20          200.00000000 sec
TD0                     1

======== CHANNEL f1 ========
SFO1         100.6238346 MHz
NUC1                 13C
P1                 10.00 usec
P13              2000.00 usec
P26               500.00 usec
PLW1         36.00000000 W
SPNAM[5]     Crp60comp.4
SPOAL5             0.500
SPOFFS5  0 Hz
SPW5          5.50040007 W
SPNAM[8] Crp60,0.5,20.1
SPOAL8             0.500
SPOFFS8  0 Hz
SPW8          5.50040007 W

======== CHANNEL f2 ========
SFO2         400.1316005 MHz
NUC2                  1H
CPDPRG[2         waltz64
PCPD2              90.00 usec
PLW2         20.00000000 W
PLW12         0.24691001 W

F2 - Processing parameters
SI                262144
SF           100.6126291 MHz
WDW                   EM
SSB      0
LB                  2.00 Hz
GB       0
PC                  1.40
```

Compound 19

Data Analysis Report

Analysis Info

Analysis Name	D:\Data\james\JM-200398-019-P_6181_1-C,7_01_10758.d	Acquisition Date	10/11/2016 15:37:20	
Method	01-microtof-1-Identify Compounds LCMS Pos 5-95.m	Operator	Dundee	
Sample Name	JM-200398-019-P_6181	Instrument / Ser#	micrOTOF	213750.00101
Comment				

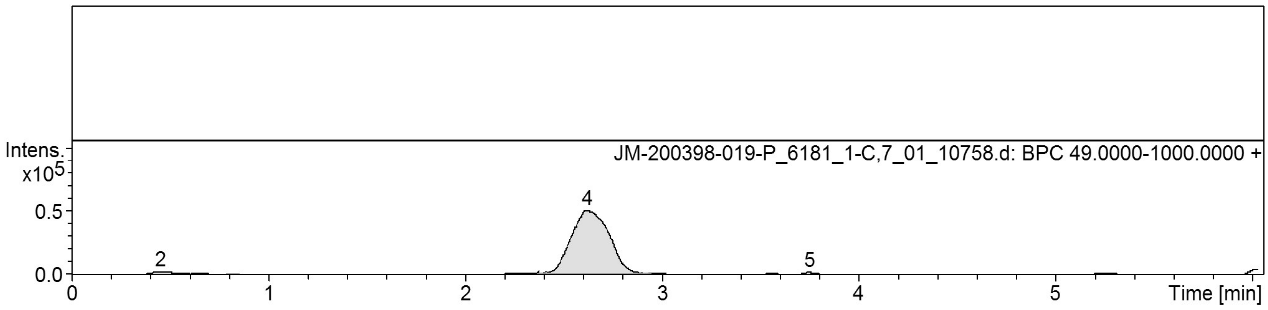

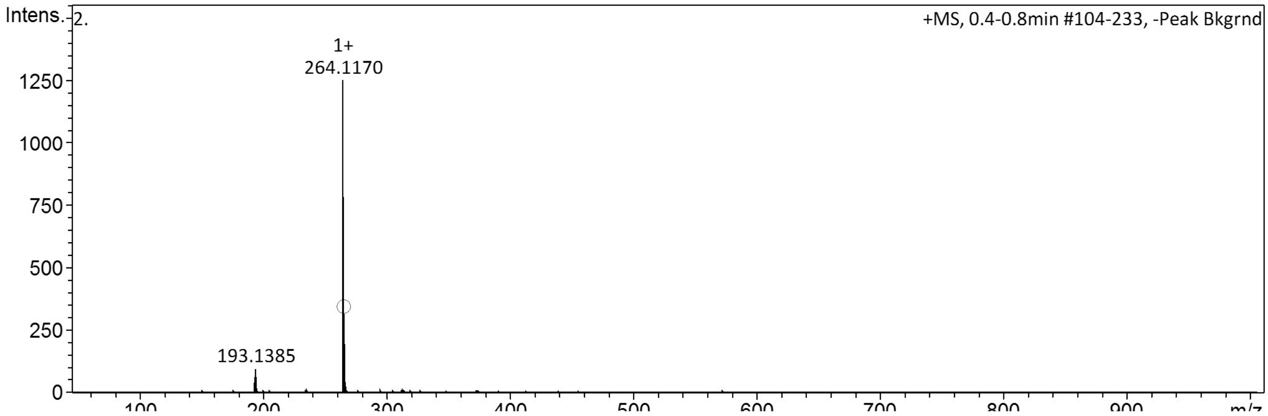

#	RT [min]	Area	Area Frac. %	Chromatogram
1	0.3	68.817	7.73	UV Chromatogram, 254 nm
2	0.4	26486.832	3.73	BPC 49.0000-1000.0000 +
3	2.6	821.464	92.27	UV Chromatogram, 254 nm
4	2.6	677051.438	95.23	BPC 49.0000-1000.0000 +
5	3.7	7448.531	1.05	BPC 49.0000-1000.0000 +

Compound Verification Report (Compass OpenAccess/QC)

Sample-ID	JM-200398-019-P	Station	Microtof-2
Submitter	James Martin	Supervisor	System Administrator
Analysis Name	JM-200398-019-P_9095_RC6_01_9777.d	Acquisition Date	15/11/2016 14:25:30
Sample Description		Method	2-microtof-2 verify compounds lcms pos 5-95.m

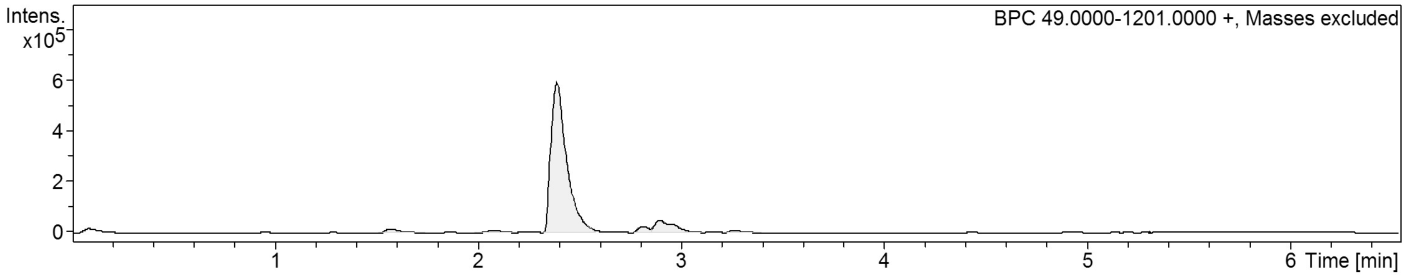

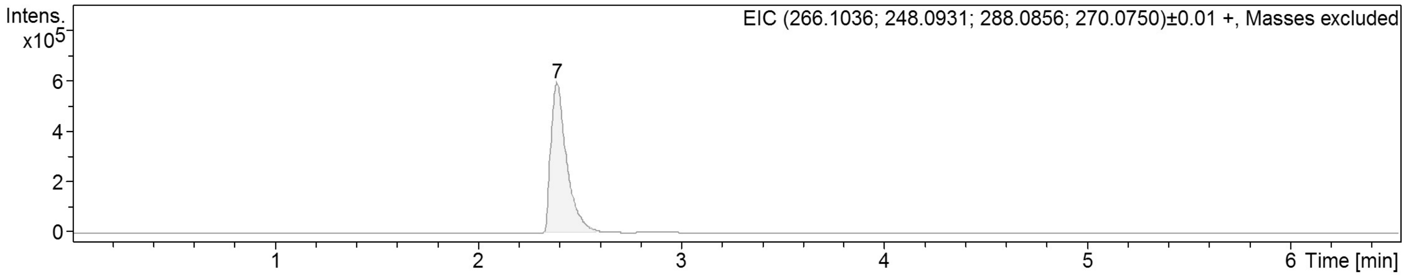

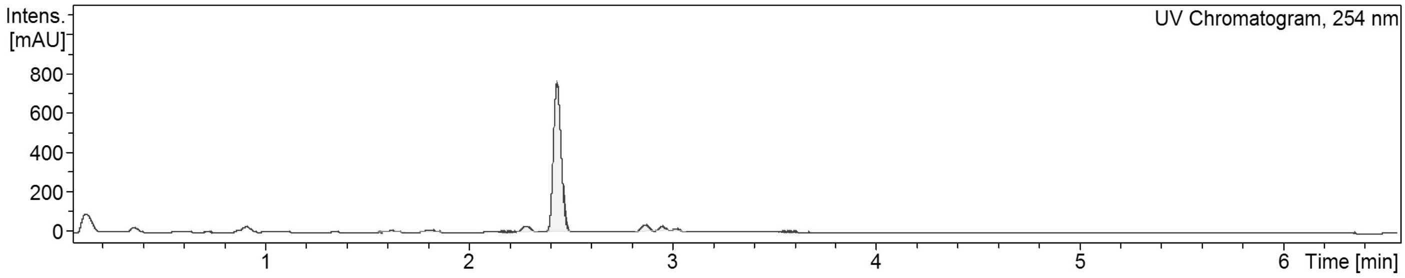

SmartFormula Settings

Tolerance	mSigma Limit	Electron Conf.
10 ppm	60	even

Adduction(s): H, Na **Neutral Loss(es):** H_2O

Compound Verification Results

Expected Formula: C14H11N5O

#	meas. m/z	theo. m/z	|err| [ppm]	mSigma	Formula	Modification	Purity(UVC)[%]	Purity(BPC)[%]
7	266.1049	266.1036	4.7	51	C14 H12 N5 O	(M+H)+	0.0	85.3

Note: mSigma values <20 indicate high probability of correct molecular formula

Compound Verification Report (Compass OpenAccess/QC)

Cmpd 7, 2.4 min

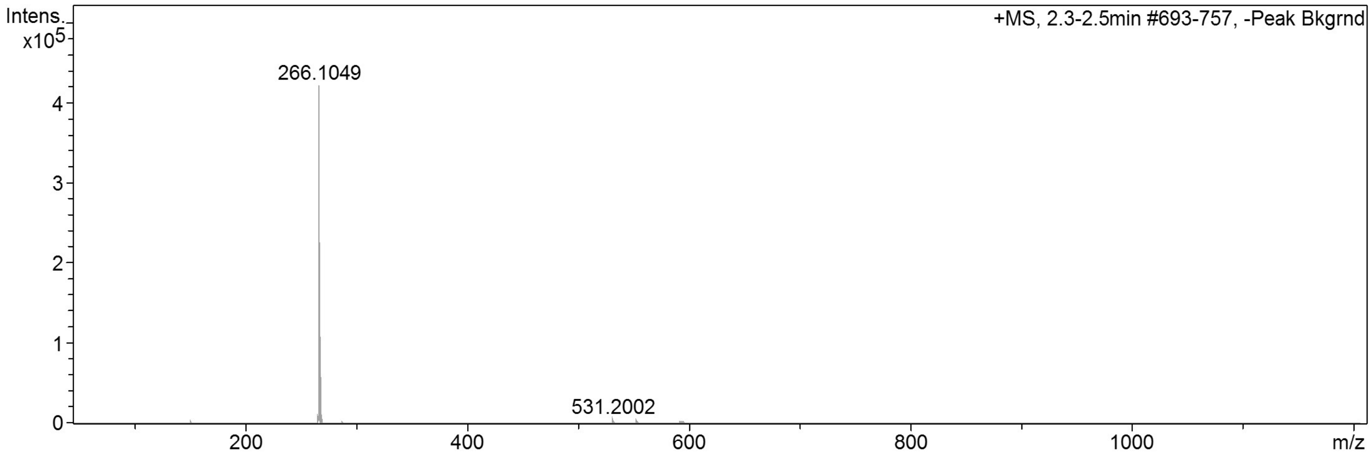

Compound 21

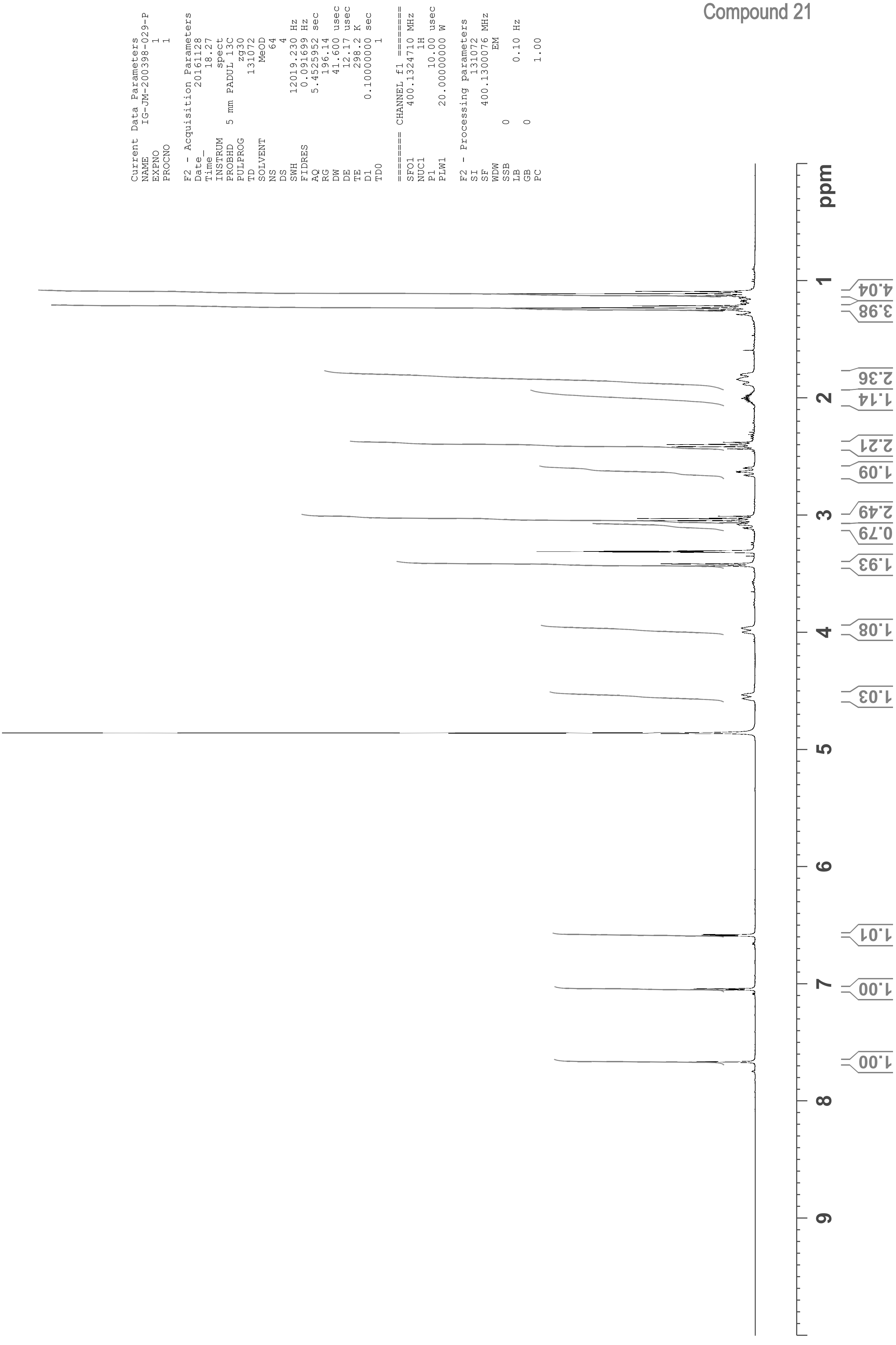
Current Data Parameters
NAME IG-JM-200398-029-P
EXPNO 1
PROCNO 1
F2 - Acquisition Parameters
Date_ 20161128
Time 18.27
INSTRUM spect
PROBHD 5 mm PADUL 13C
PULPROG zg30
TD 131072
SOLVENT MeOD
NS 64
DS 4
SWH 12019.230 Hz
FIDRES 0.091699 Hz
AQ 5.4525952 sec
RG 196.14
DW 41.600 usec
DE 12.17 usec
TE 298.2 K
D1 0.10000000 sec
TD0 1
======== CHANNEL f1 ========
SFO1 400.1324710 MHz
NUC1 1H
P1 10.00 usec
PLW1 20.00000000 W
F2 - Processing parameters
SI 131072
SF 400.1300076 MHz
WDW EM
SSB 0
LB 0.10 Hz
GB 0
PC 1.00
4.04
3.98
2.36
1.14
2.21
1.09
2.49
0.79
1.93
1.08
1.03
1.01
1.00
1.00
ppm
1
2
3
4
5
6
7
8
9

Compound 21

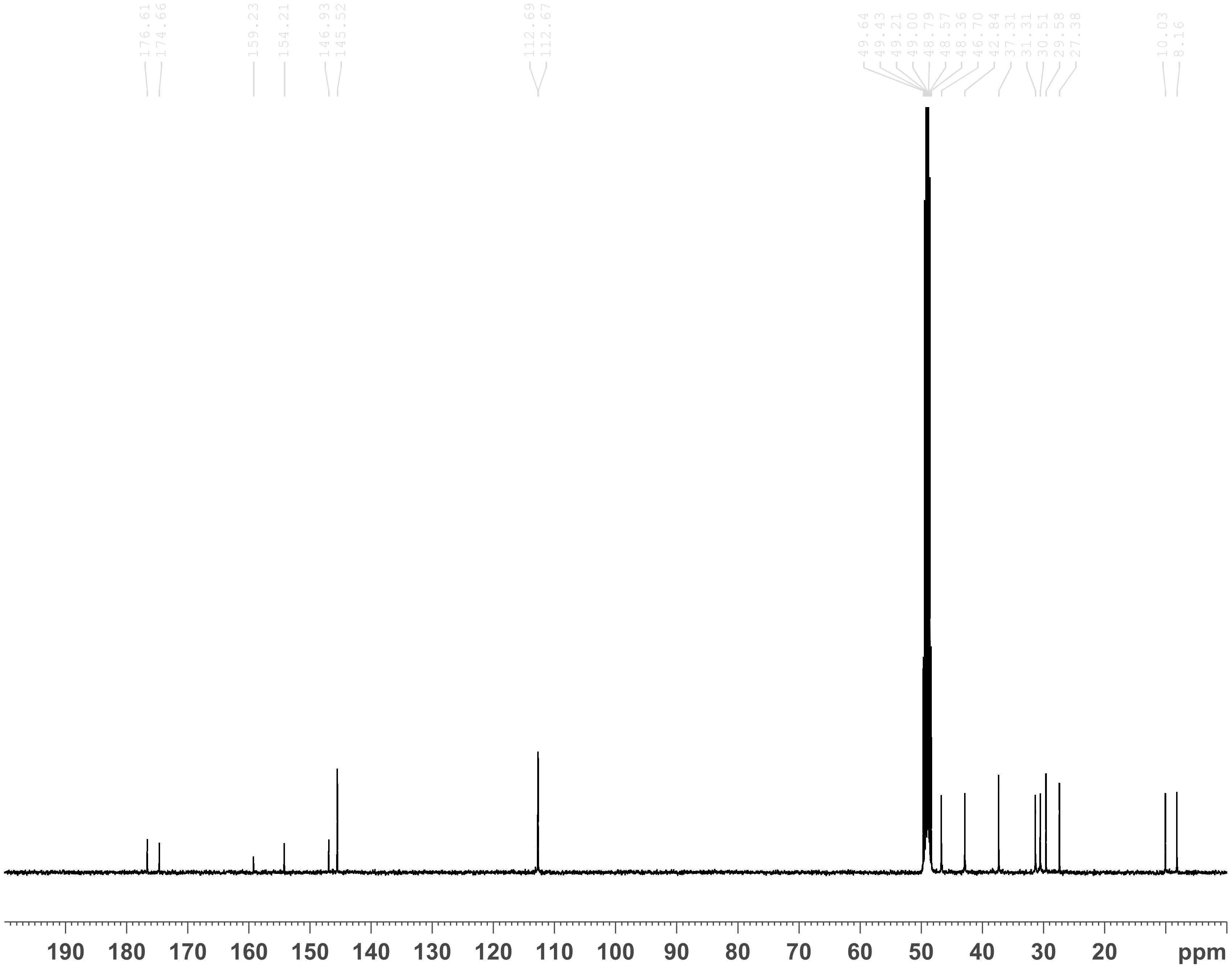


```
Current Data Parameters
NAME       IG-JM-200398-029-P
EXPNO                      2
PROCNO                     1

F2 - Acquisition Parameters
Date_            20161128
Time                20.16
INSTRUM             spect
PROBHD   5 mm PADUL 13C
PULPROG             udeft
TD                  17996
SOLVENT              MeOD
NS                   1024
DS                      0
SWH             25000.000 Hz
FIDRES           1.389198 Hz
AQ              0.3599200 sec
RG                 196.14
DW                 20.000 usec
DE                   8.66 usec
TE                  298.2 K
D1             3.00000000 sec
D11            0.03000000 sec
D12            0.00002000 sec
D20          200.00000000 sec
TD0                     1

======== CHANNEL f1 ========
SFO1         100.6238346 MHz
NUC1                 13C
P1                 10.00 usec
P13              2000.00 usec
P26               500.00 usec
PLW1         36.00000000 W
SPNAM[5]     Crp60comp.4
SPOAL5             0.500
SPOFFS5  0 Hz
SPW5          5.50040007 W
SPNAM[8] Crp60,0.5,20.1
SPOAL8             0.500
SPOFFS8  0 Hz
SPW8          5.50040007 W

======== CHANNEL f2 ========
SFO2         400.1316005 MHz
NUC2                  1H
CPDPRG[2         waltz64
PCPD2              90.00 usec
PLW2         20.00000000 W
PLW12         0.24691001 W

F2 - Processing parameters
SI                262144
SF          100.6126277 MHz
WDW                   EM
SSB      0
LB                  2.00 Hz
GB       0
PC                  1.40
```

Compound Verification Report (Compass OpenAccess/QC)

Sample-ID	JM-200398-029	Station	Microtof-2
Submitter	James Martin	Supervisor	System Administrator
Analysis Name	JM-200398-029_9394_RB3_01_10081.d	Acquisition Date	24/11/2016 11:28:42
Sample Description		Method	2-microtof-2 verify compounds lcms pos 5-95.m

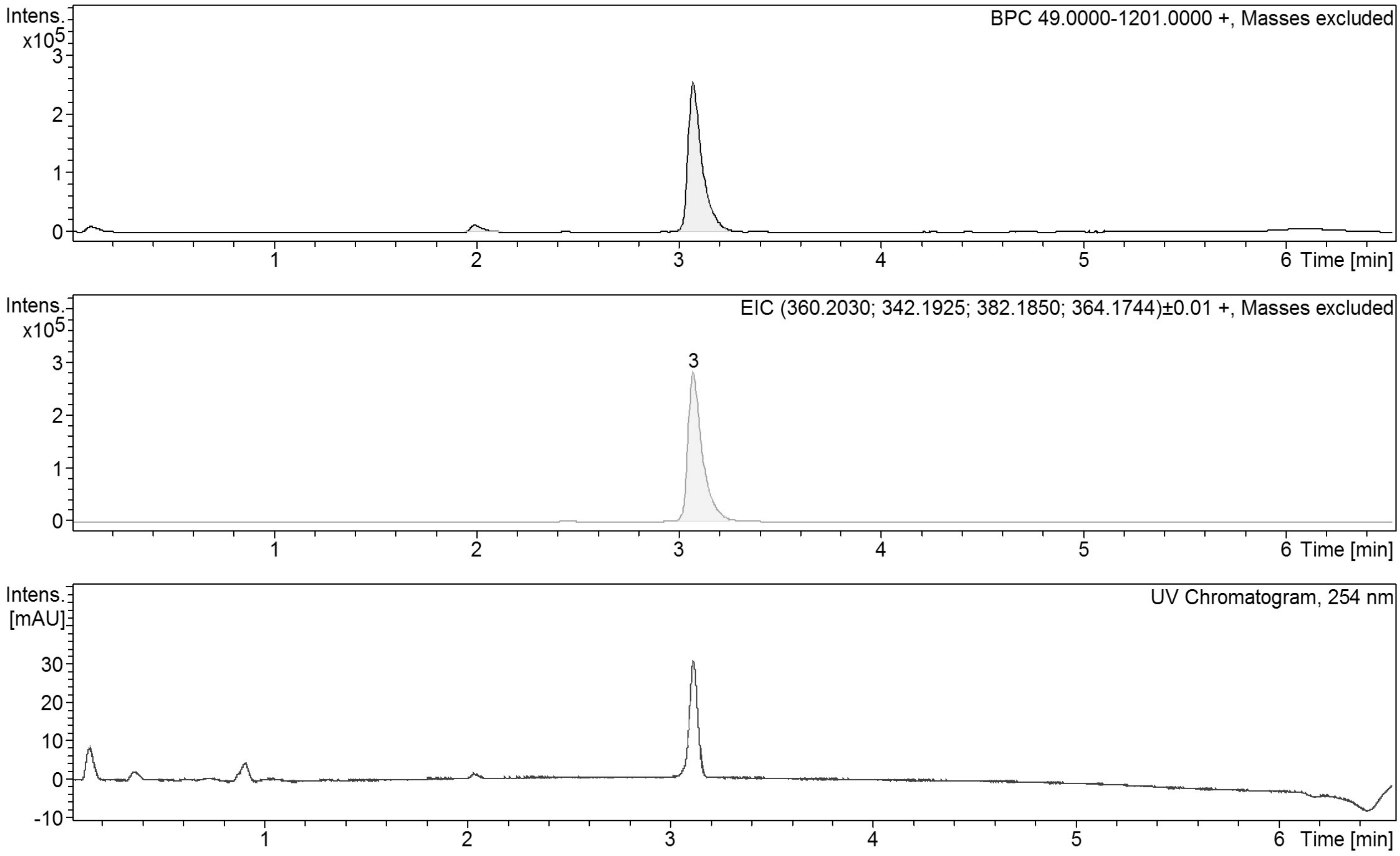

SmartFormula Settings

Tolerance	mSigma Limit	Electron Conf.
10 ppm	60	even

Adduction(s): H, Na **Neutral Loss(es):** H2O

Compound Verification Results

Expected Formula: C18H25N5O3

#	meas. m/z	theo. m/z	|err| [ppm]	mSigma	Formula	Modification	Purity(UVC)[%]	Purity(BPC)[%]
3	360.2028	360.2030	0.5	4	C18 H26 N5 O3	(M+H)+	0.0	96.3

Note: mSigma values <20 indicate high probability of correct molecular formula

Compound Verification Report (Compass OpenAccess/QC)

Cmpd 3, 3.1 min

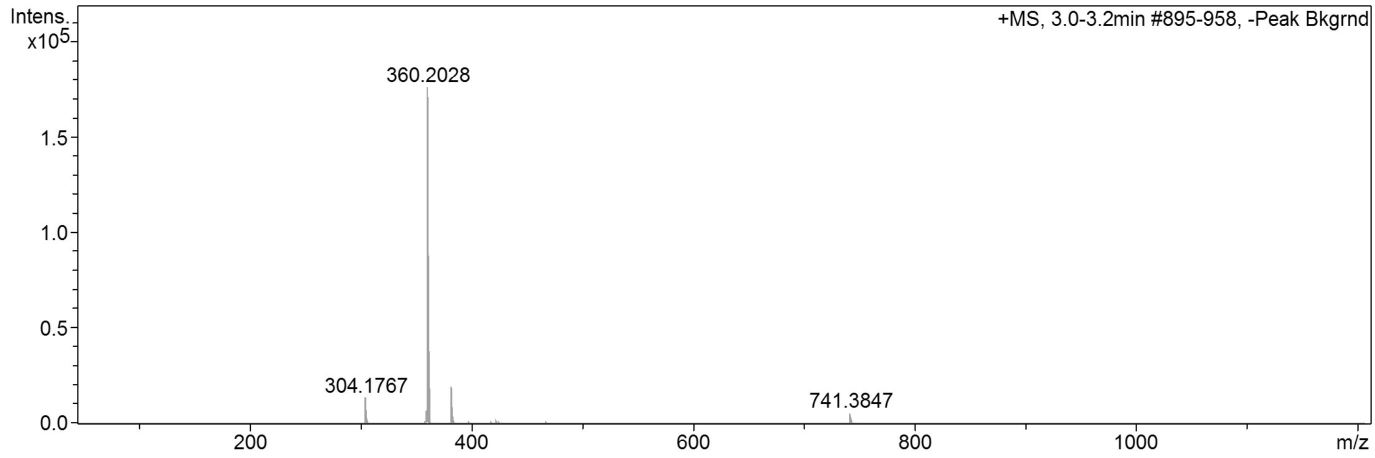

PROTON.d CDCl3 {C:\Bruker\TopSpin3.2} IG500 4

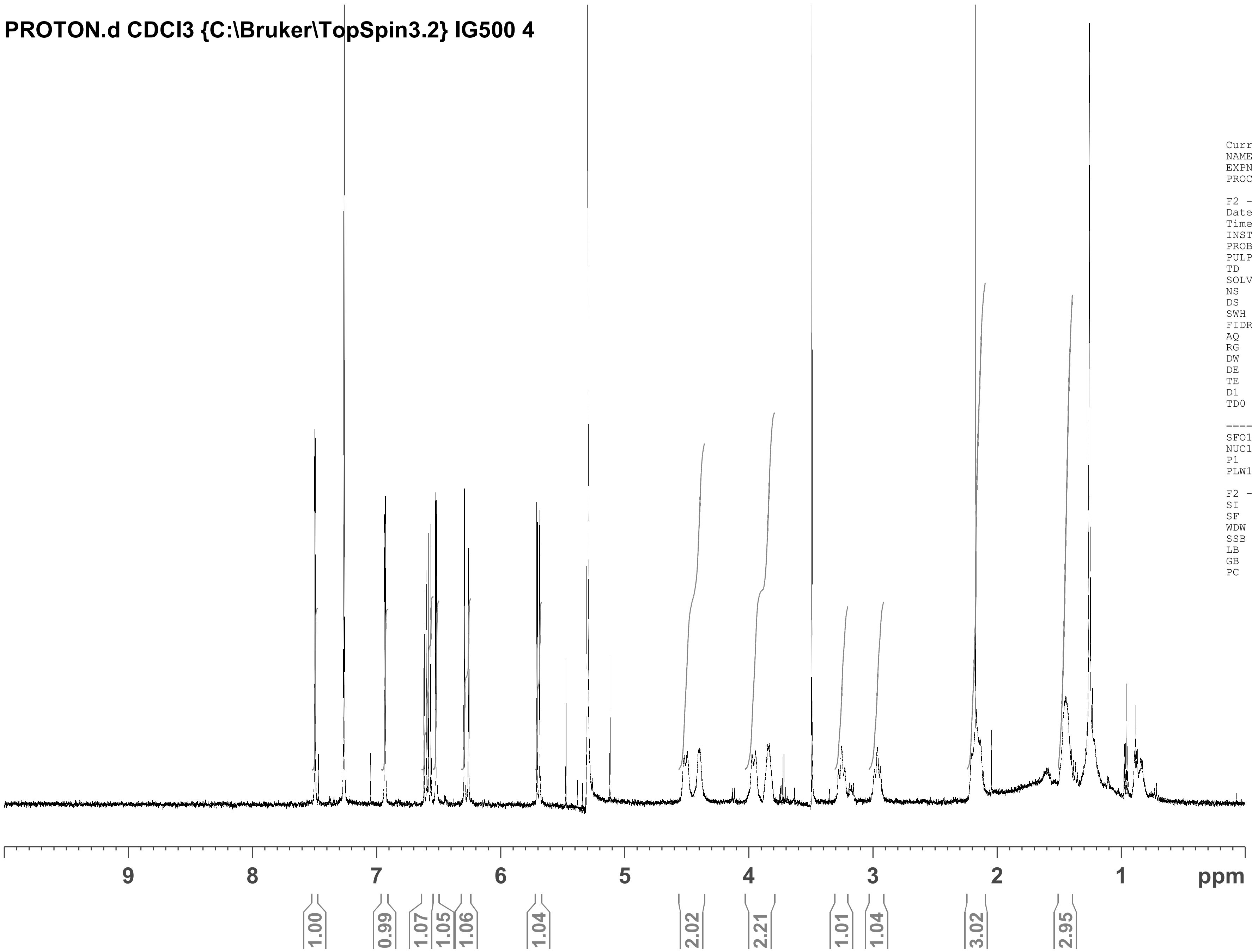


```
Current Data Parameters
NAME     IG-JM-200398-014-001
EXPNO                   1
PROCNO                  1

F2 - Acquisition Parameters
Date_          20161012
Time              12.18
INSTRUM           spect
PROBHD   5 mm QNP 1H/13
PULPROG            zg30
TD                65536
SOLVENT           CDCl3
NS                   16
DS                    2
SWH           10000.000 Hz
FIDRES         0.152588 Hz
AQ            3.2767999 sec
RG                  575
DW               50.000 usec
DE                 6.50 usec
TE                298.2 K
D1           1.00000000 sec
TD0                   1

======== CHANNEL f1 ========
SFO1        500.1330885 MHz
NUC1                 1H
P1                10.00 usec
PLW1        25.00000000 W

F2 - Processing parameters
SI                65536
SF          500.1300134 MHz
WDW                  EM
SSB      0
LB                 0.30 Hz
GB       0
PC                 1.00
```

Compound 22

Compound Verification Report (Compass OpenAccess/QC)

Sample-ID	200398-014-001	Station	Microtof-2
Submitter	James Martin	Supervisor	System Administrator
Analysis Name	200398-014-001_8754_RC7_01_9373.d	Acquisition Date	31/10/2016 17:12:04
Sample Description		Method	5-LC-HRMS-ISOCRATIC-GE8.m

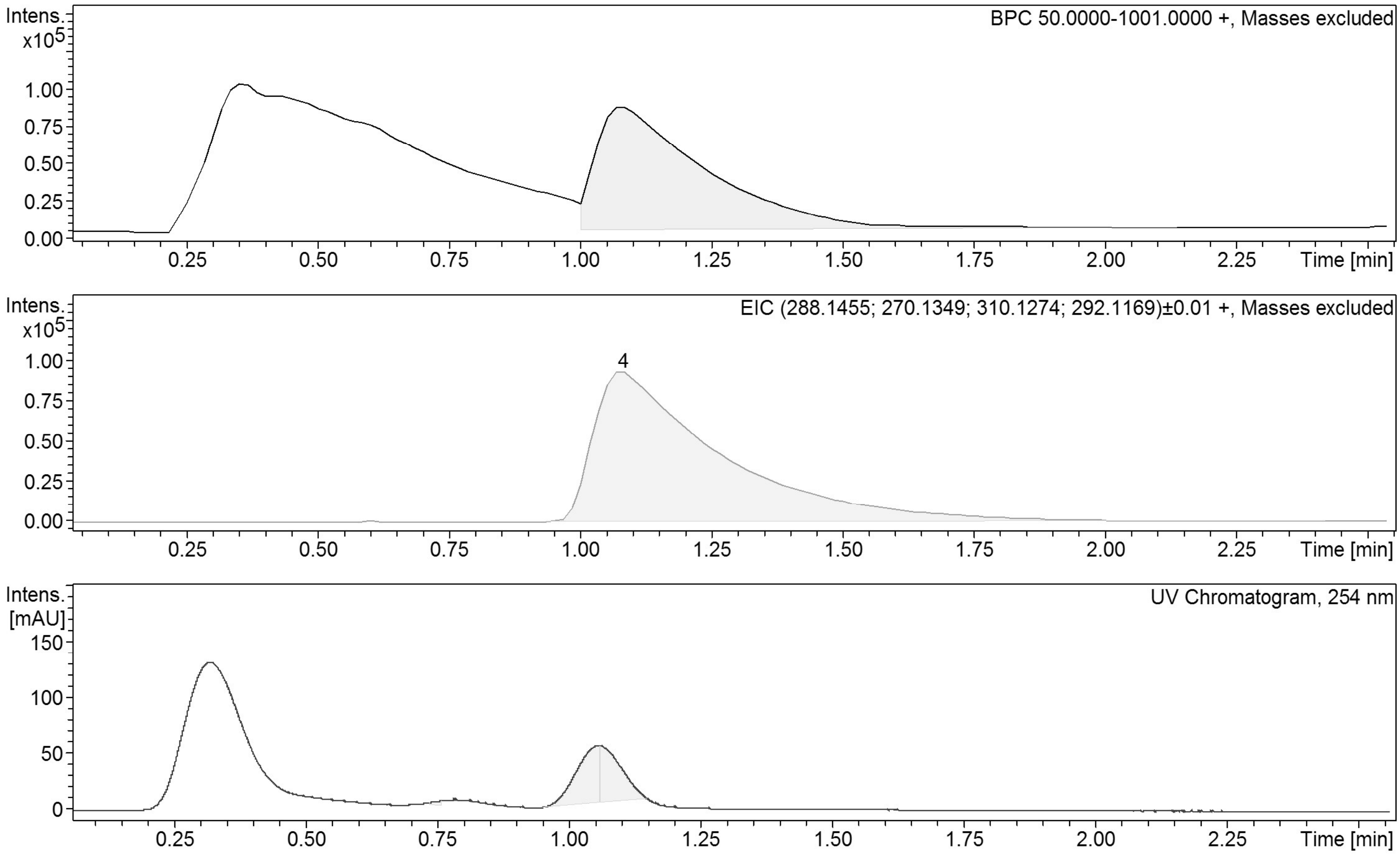

SmartFormula Settings

Tolerance	mSigma Limit	Electron Conf.
10 ppm	60	even

Adduction(s): H, Na **Neutral Loss(es):** H_2O

Compound Verification Results

Expected Formula: $C_{14}H_{17}N_5O_2$

#	meas. m/z	theo. m/z	|err| [ppm]	mSigma	Formula	Modification	Purity(UVC)[%]	Purity(BPC)[%]
4	288.1453	288.1455	0.5	23	C14 H18 N5 O2	(M+H)+	48.4	100.0

Note: mSigma values <20 indicate high probability of correct molecular formula

Compound Verification Report (Compass OpenAccess/QC)

Cmpd 4, 1.1 min

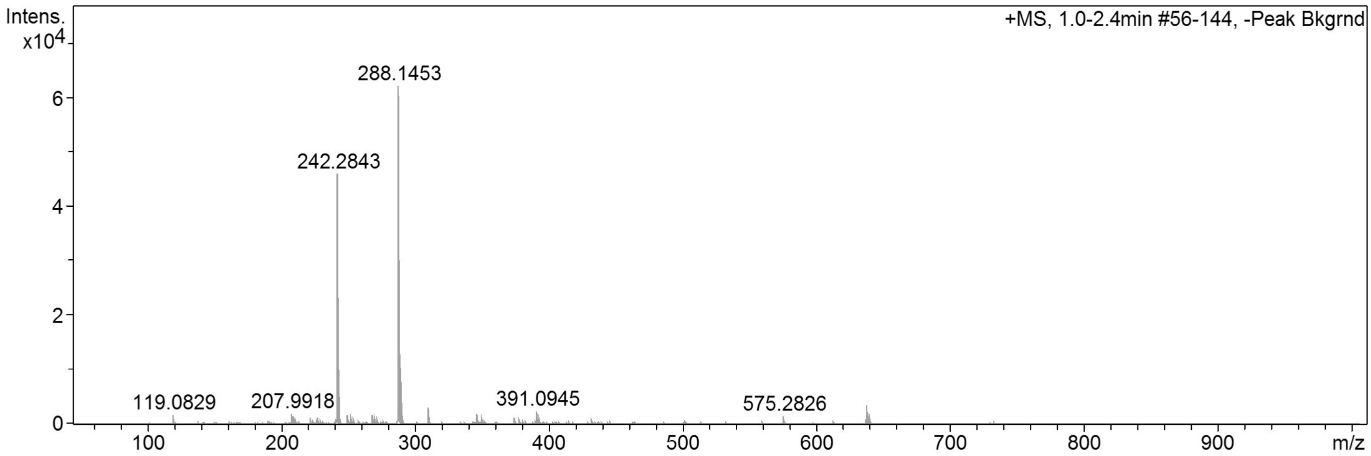

Compass OpenAccess LC-MS Identification Report

Sample-ID	200398-014-001	Station	Microtof-2
Submitter	James Martin	Supervisor	System Administrator
Analysis Name	200398-014-001_8763_RD8_01_9383.d	Acquisition Date	01/11/2016 10:20:58
Sample Description		Method	1-microtof-2 Identify Compounds LCMS Pos 5-95.m

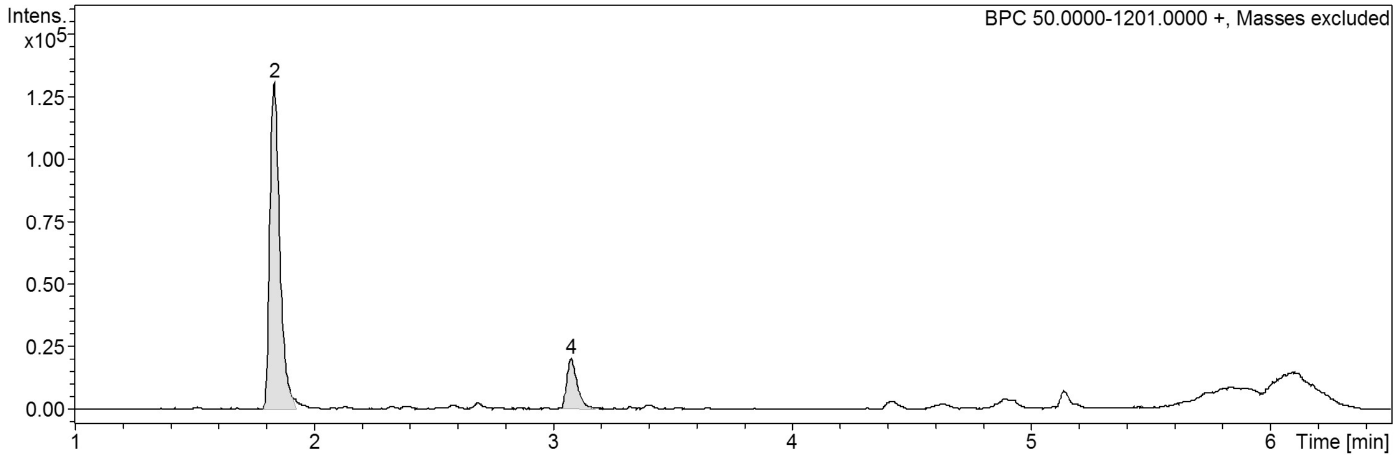

Sample Description

Method 1-microtof-2 Identify Compounds LCMS Pos 5-95.m

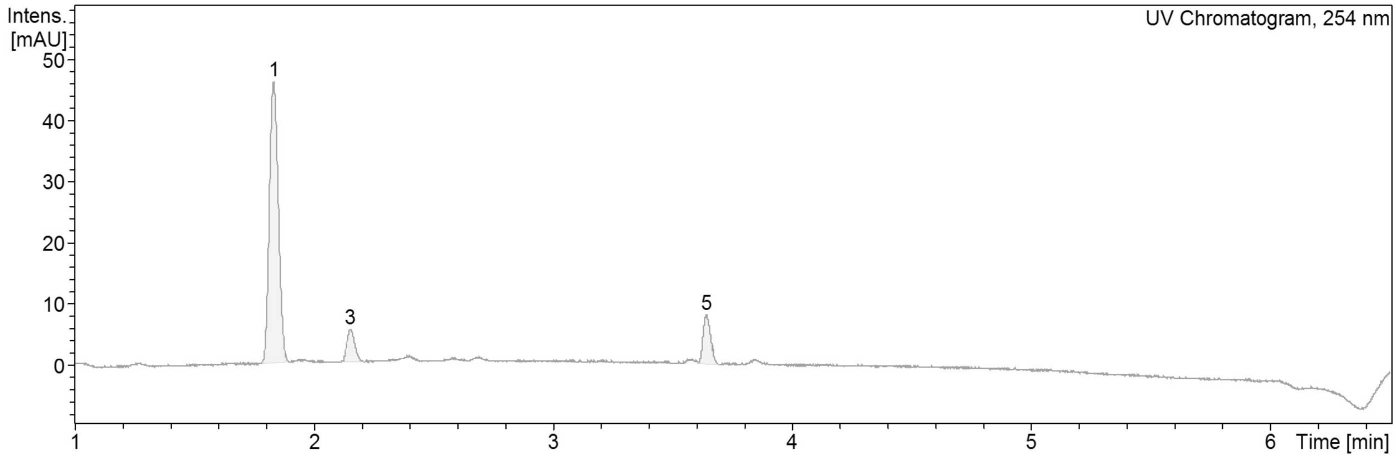

#	RT [min]	Area Frac. %	Chromatogram
1	1.8	79.599	UV Chromatogram, 254 nm
2	1.8	86.077	BPC 50.0000-1201.0000 +, Masses excluded
3	2.2	8.490	UV Chromatogram, 254 nm
4	3.1	13.923	BPC 50.0000-1201.0000 +, Masses excluded
5	3.6	11.912	UV Chromatogram, 254 nm

SmartFormula Settings

Tolerance	mSigma Limit	Electron Conf.
20 ppm	60	even

Compass OpenAccess LC-MS Identification Report

Adduction(s): **Neutral Loss(es):**

SmartFormula Results

FormulaMin: C14H18N5O2 **FormulaMax:** Na

#	meas. m/z	theo. m/z	|err| [ppm]	mSigma	Formula	Purity(UVC)[%]	Purity(BPC)[%]
2	575.2794	575.2853	10.3	3	C31 H36 N8 Na O2	79.6	86.1
		575.2741	9.2	6	C32 H36 N6 Na O3		
		597.2528	14.1	5	C26 H33 N10 O7		
		597.2643	5.1	6	C27 H38 N6 Na O8		

Note: mSigma values <30 indicate high probability of correct molecular formula

Cmpd 1,
1.8 min

Undefined context.
Component not placed in a spectrum iterator.

Cmpd 2,
1.8 min

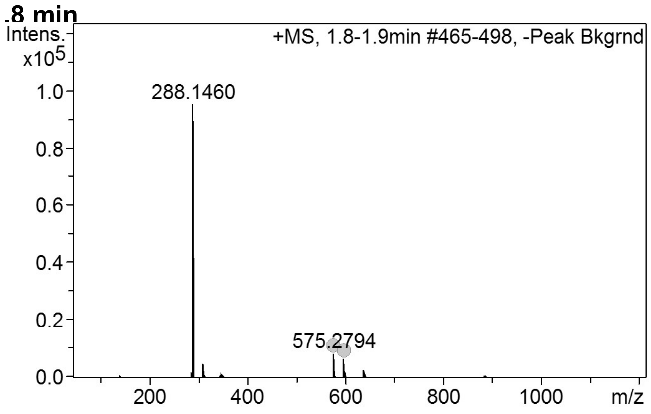

Cmpd 3,
2.2 min

Undefined context.
Component not placed in a spectrum iterator.

Cmpd 4,
3.1 min

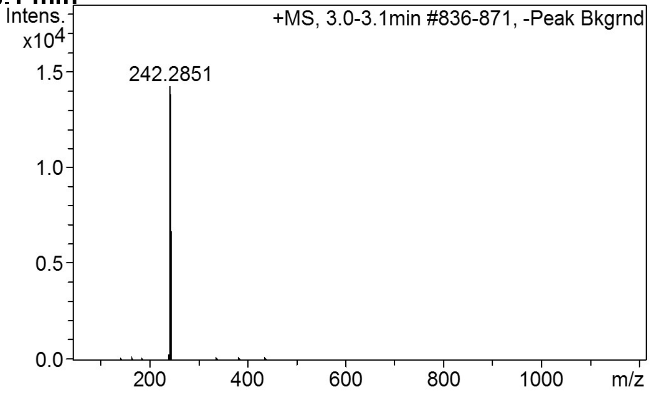

Cmpd 5,

Compass OpenAccess LC-MS Identification Report

3.6 min

Undefined context.
Component not placed in a spectrum iterator.

Compound 23

PROTON256 DMSO {C:\Bruker\TopSpin3.2} IG500 1

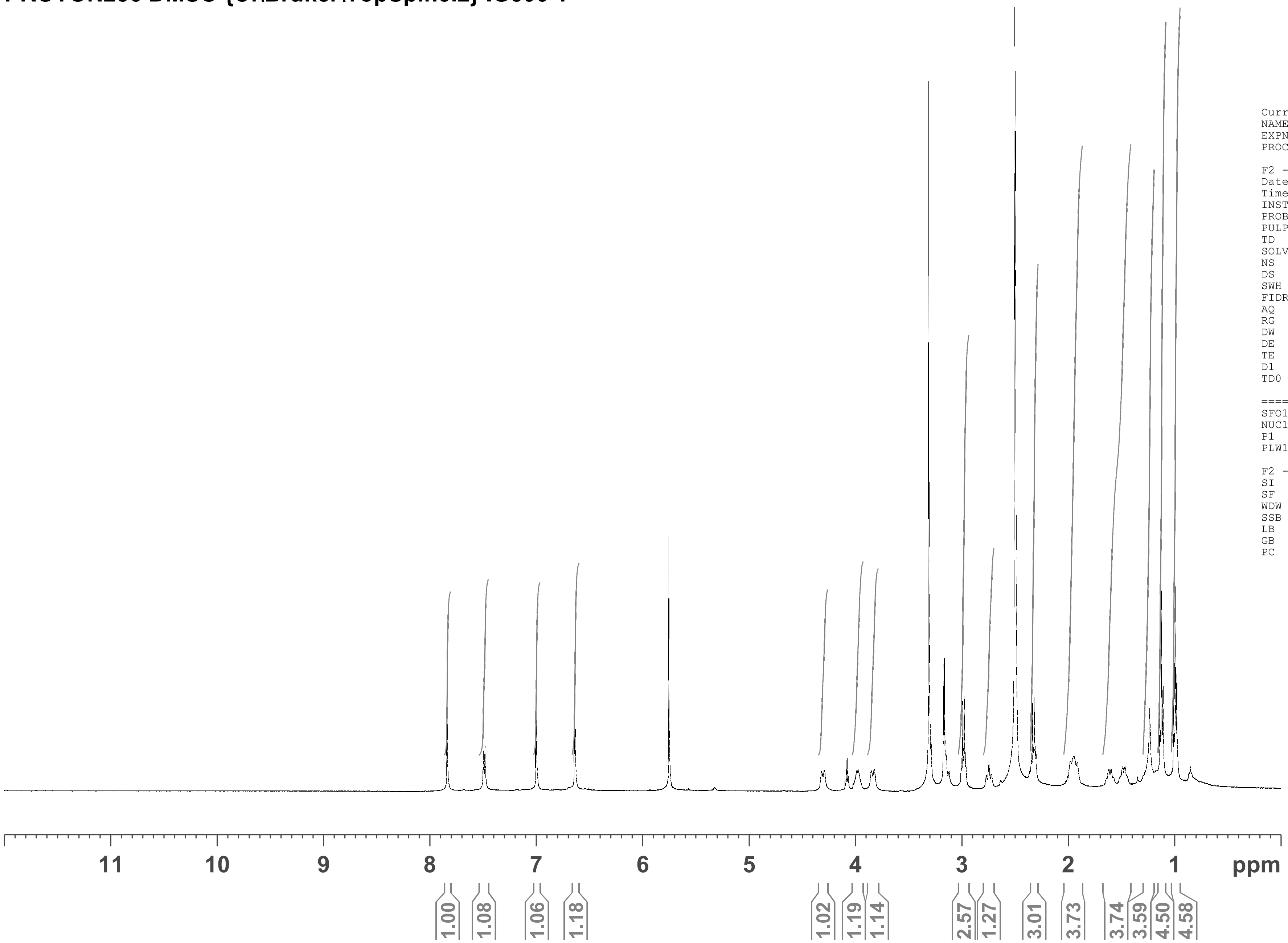


```
Current Data Parameters
NAME      IG-JM-200398-027-001
EXPNO                    1
PROCNO                   1

F2 - Acquisition Parameters
Date_             20161118
Time                 11.25
INSTRUM              spect
PROBHD    5 mm QNP 1H/13
PULPROG               zg30
TD                   65536
SOLVENT               DMSO
NS                     256
DS                       2
SWH              10000.000 Hz
FIDRES            0.152588 Hz
AQ               3.2767999 sec
RG                     322
DW                  50.000 usec
DE                    6.50 usec
TE                   298.2 K
D1              1.00000000 sec
TD0                      1

======== CHANNEL f1 ========
SFO1           500.1330885 MHz
NUC1                    1H
P1                   10.00 usec
PLW1           25.00000000 W

F2 - Processing parameters
SI                   65536
SF             500.1300052 MHz
WDW                     EM
SSB       0
LB                    0.30 Hz
GB        0
PC                    1.00
```

Compound 23

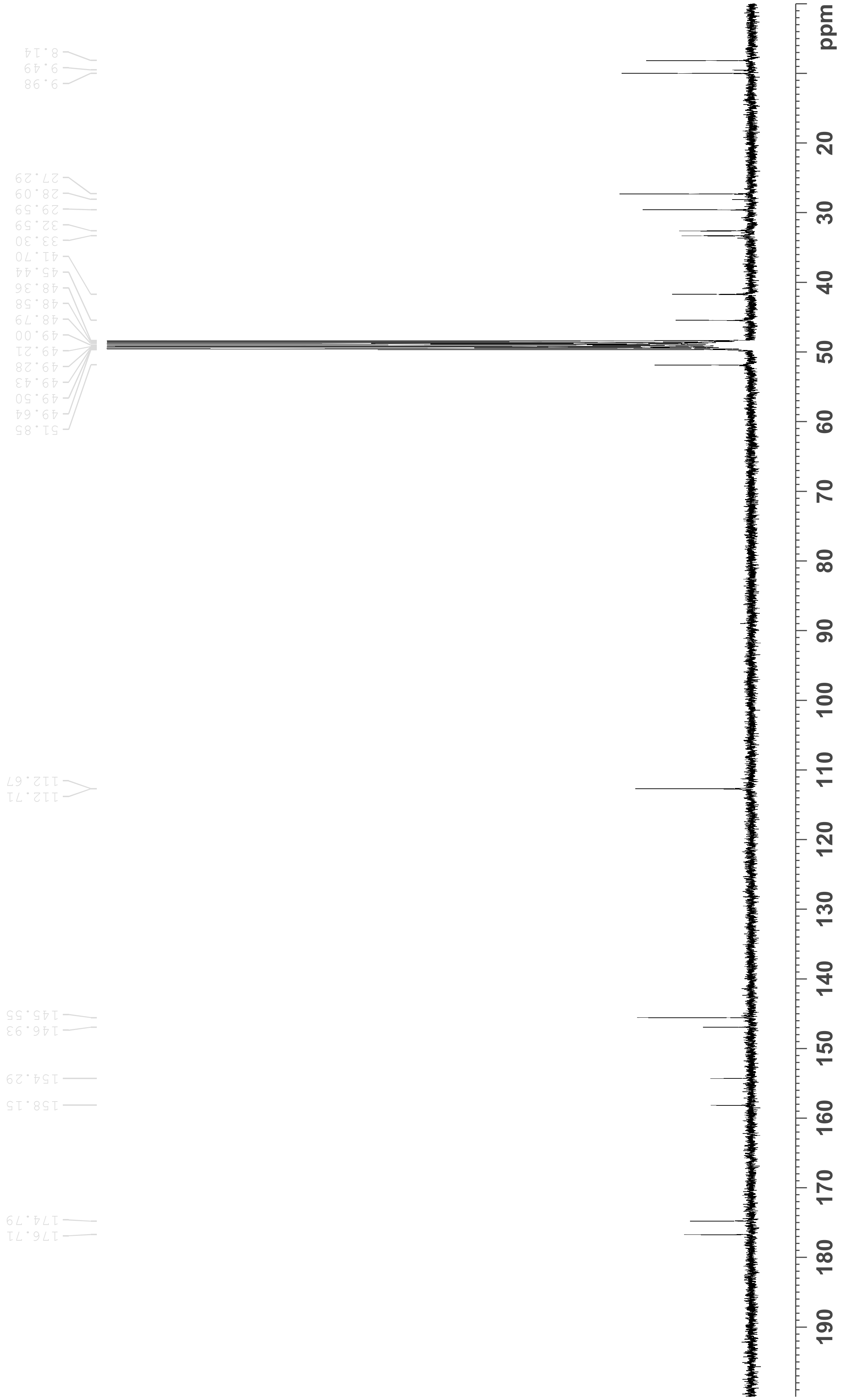
ppm
8.14
9.49
9.98
27.29
28.09
29.59
32.59
33.30
41.70
45.44
48.36
48.58
48.79
49.00
49.21
49.28
49.43
49.50
49.64
51.85
112.67
112.71
145.55
146.93
154.29
158.15
174.79
176.71

Compound Verification Report (Compass OpenAccess/QC)

Sample-ID	JM-200398-028-001	Station	Microtof-2
Submitter	James Martin	Supervisor	System Administrator
Analysis Name	JM-200398-028-001_9140_RD1_01_9828.d	Acquisition Date	17/11/2016 13:47:16
Sample Description		Method	2-microtof-2 verify compounds lcms pos 5-95.m

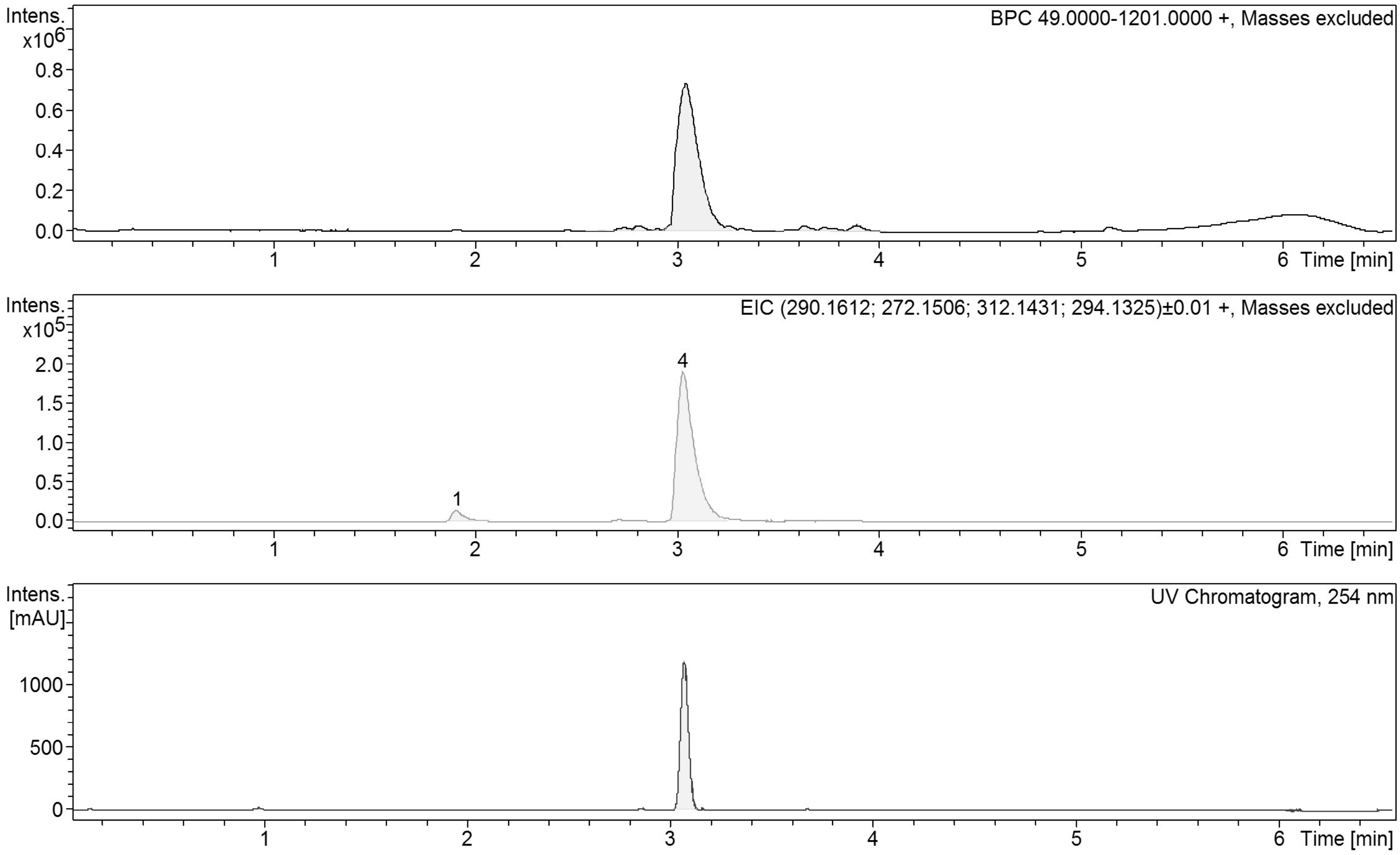

SmartFormula Settings

Tolerance	mSigma Limit	Electron Conf.
10 ppm	60	even

Concentration too high. Dilute sample!

Adduction(s): H, Na **Neutral Loss(es):** H2O

Compound Verification Results

Expected Formula: C14H19N5O2

#	meas. m/z	theo. m/z	|err| [ppm]	mSigma	Formula	Modification	Purity(UVC)[%]	Purity(BPC)[%]
1	290.1609	290.1612	0.9	2	C14 H20 N5 O2	(M+H)+	0.0	0.0

Note: mSigma values <20 indicate high probability of correct molecular formula

Compound Verification Report (Compass OpenAccess/QC)

Cmpd 1, 1.9 min

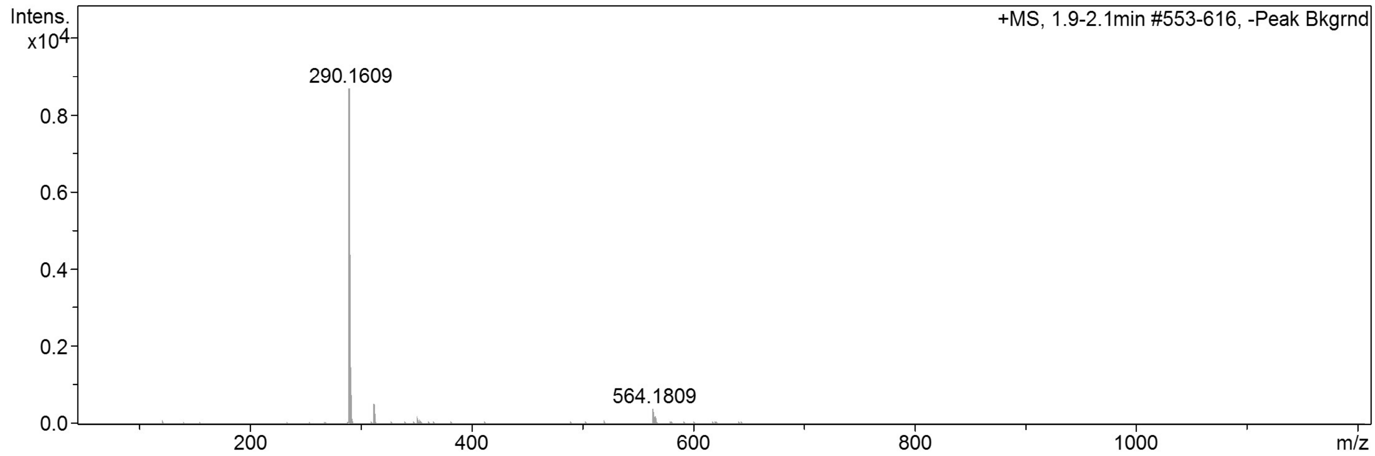

Cmpd 4, 3.0 min

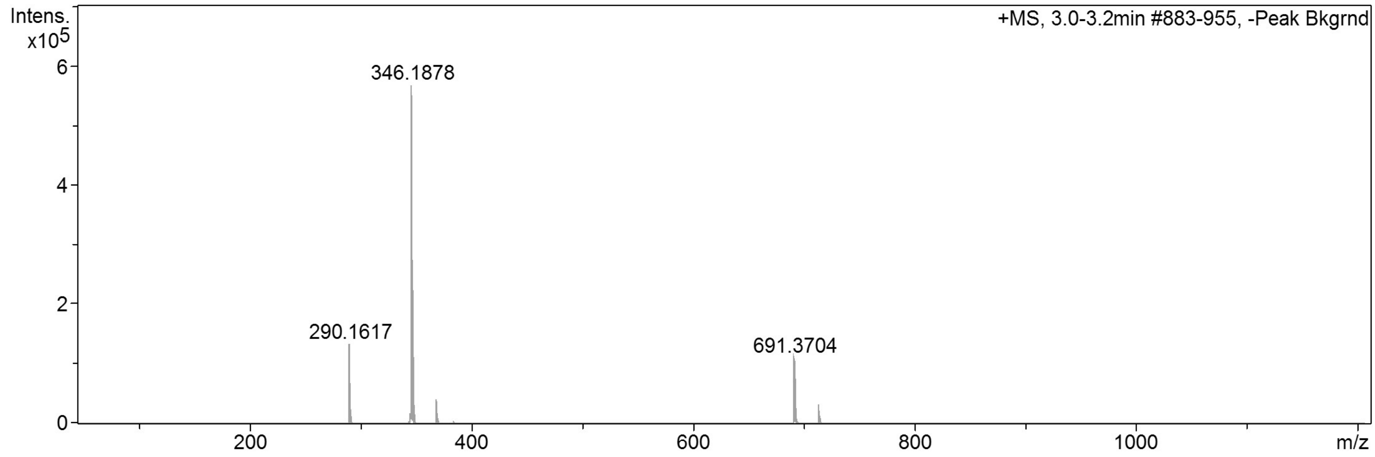

PROTON256 DMSO {C:\Bruker\TopSpin3.2} IG500 6

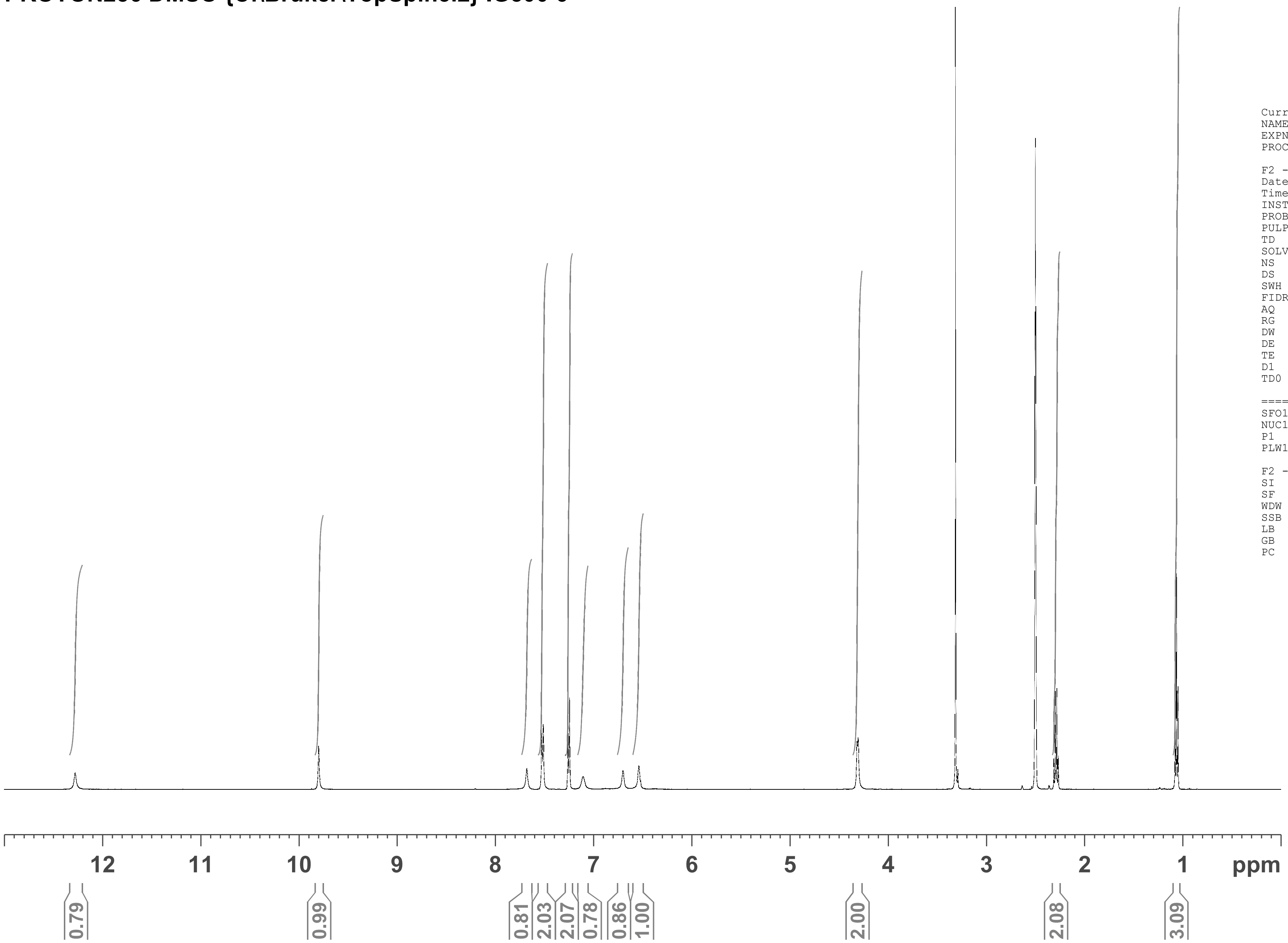


```
Current Data Parameters
NAME     IG-JM-200398-045-001
EXPNO                   1
PROCNO                  1

F2 - Acquisition Parameters
Date_          20161214
Time              17.05
INSTRUM           spect
PROBHD   5 mm QNP 1H/13
PULPROG            zg30
TD                65536
SOLVENT            DMSO
NS                  256
DS                    2
SWH           10000.000 Hz
FIDRES         0.152588 Hz
AQ            3.2767999 sec
RG                   32
DW               50.000 usec
DE                 6.50 usec
TE                298.2 K
D1           1.00000000 sec
TD0                   1

======== CHANNEL f1 ========
SFO1        500.1330885 MHz
NUC1                 1H
P1                10.00 usec
PLW1        25.00000000 W

F2 - Processing parameters
SI                65536
SF          500.1295230 MHz
WDW                  EM
SSB      0
LB                 0.30 Hz
GB       0
PC                 1.00
```

Compound 24

C13CPD6.d DMSO {C:\Bruker\TopSpin3.2} IG500 21

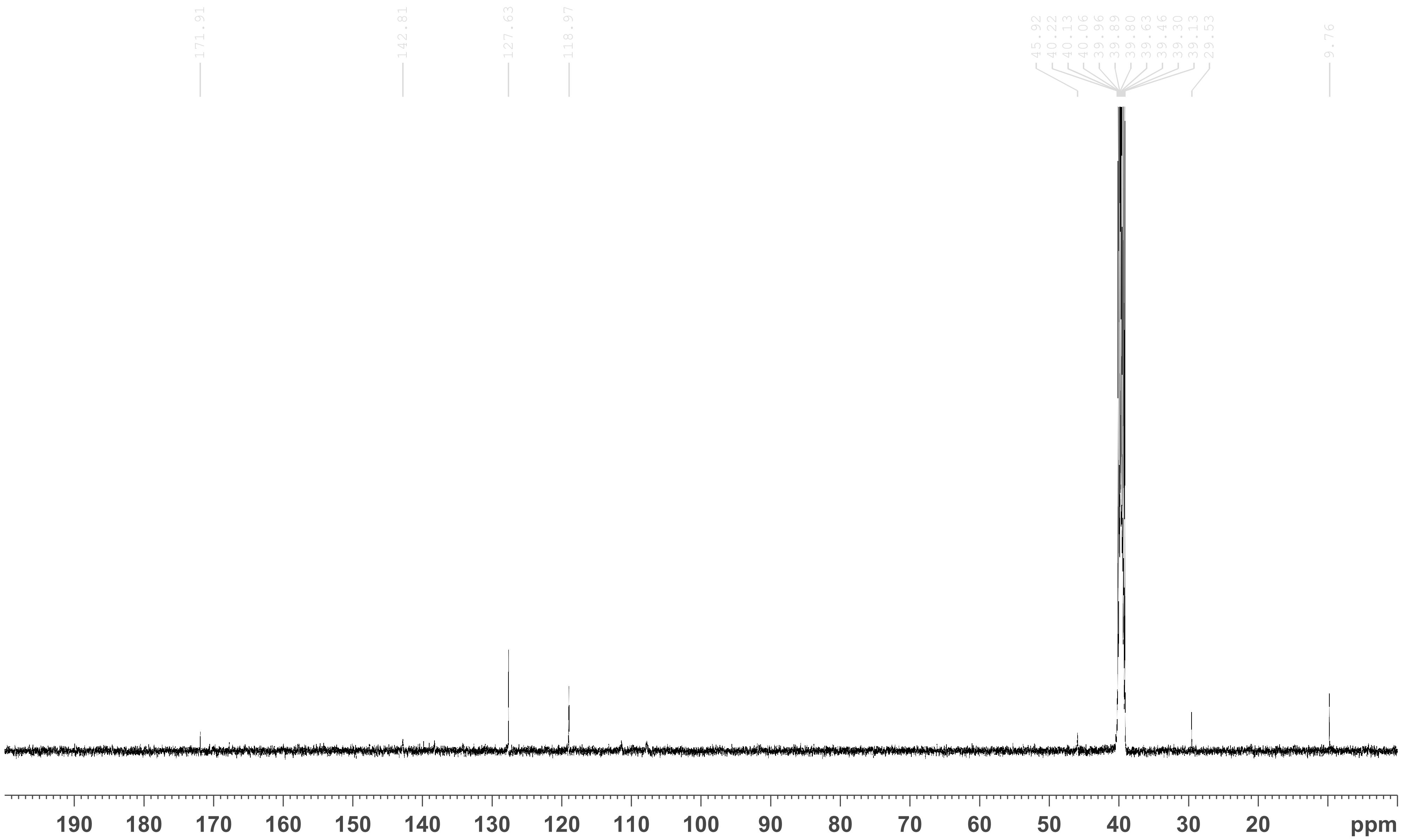

Compound 24

Compound Verification Report (Compass OpenAccess/QC)

Sample-ID	200398-045-001	Station	Microtof-2
Submitter	James Martin	Supervisor	System Administrator
Analysis Name	200398-045-001_10231_RB1_01_11131.d	Acquisition Date	09/01/2017 10:21:59
Sample Description		Method	2-microtof-2 verify compounds lcms pos 5-95.m

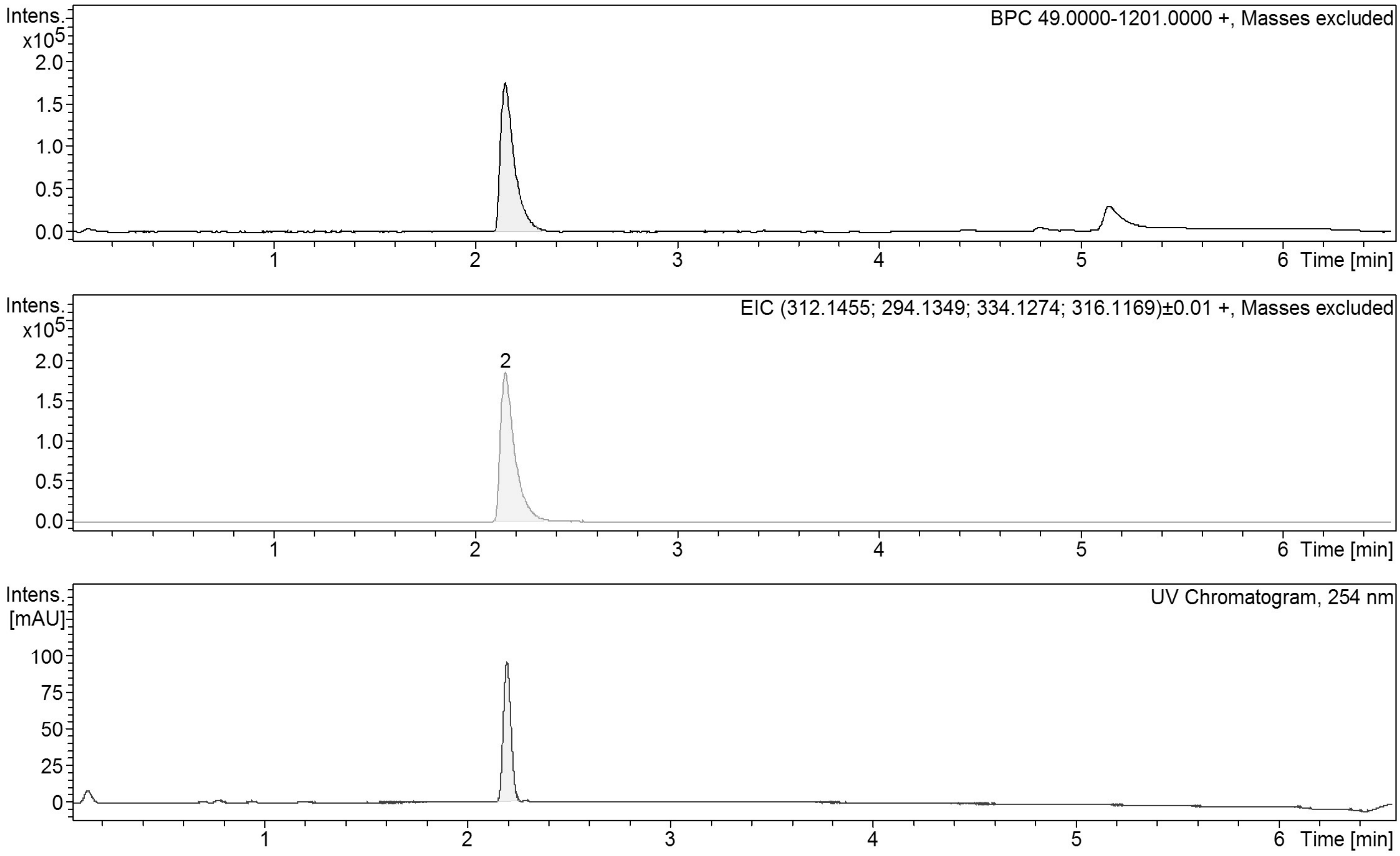

SmartFormula Settings

Tolerance	mSigma Limit	Electron Conf.
10 ppm	60	even

Adduction(s): H, Na **Neutral Loss(es):** H2O

Compound Verification Results

Expected Formula: C16H17N5O2

#	meas. m/z	theo. m/z	|err| [ppm]	mSigma	Formula	Modification	Purity(UVC)[%]	Purity(BPC)[%]
2	312.1459	312.1455	1.3	5	C16 H18 N5 O2	(M+H)+	0.0	100.0

Note: mSigma values <20 indicate high probability of correct molecular formula

Compound Verification Report (Compass OpenAccess/QC)

Cmpd 2, 2.2 min

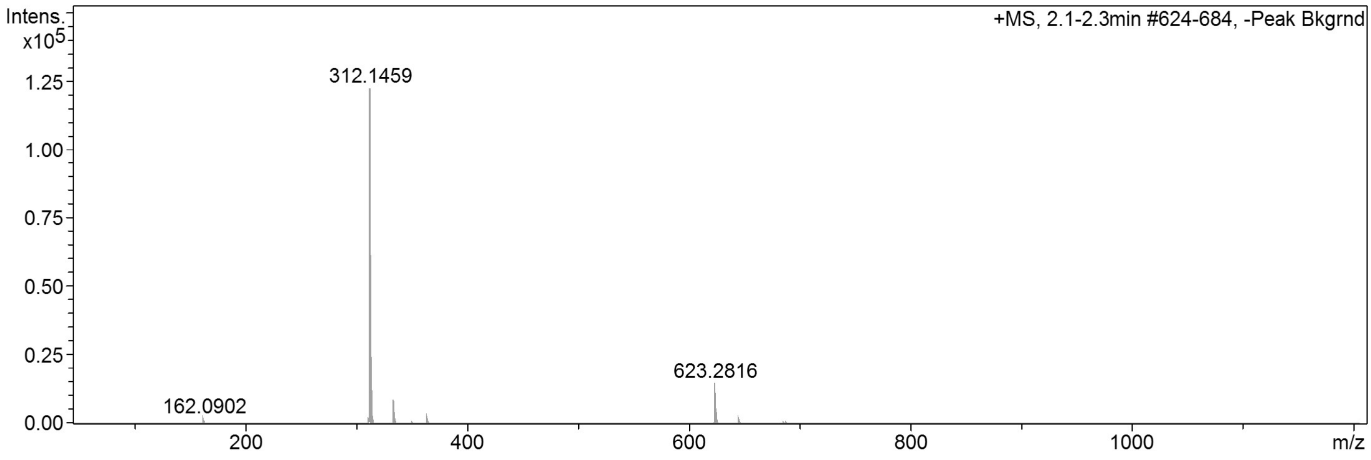

Compound 26

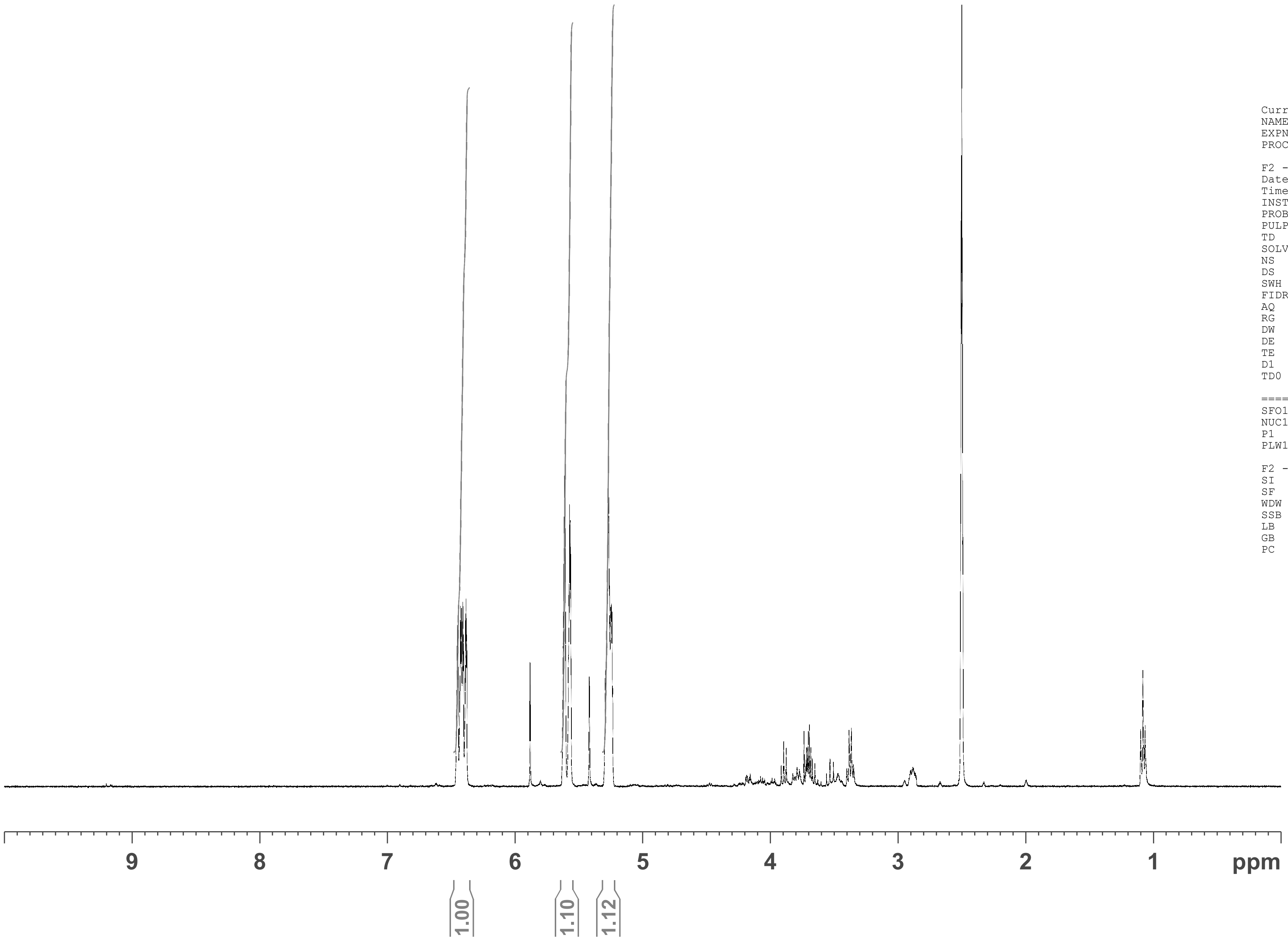


```
Current Data Parameters
NAME     IG-JM-200398-046-P3
EXPNO                  1
PROCNO                 1

F2 - Acquisition Parameters
Date_          20161215
Time              16.13
INSTRUM           spect
PROBHD   5 mm PADUL 13C
PULPROG            zg30
TD               131072
SOLVENT            DMSO
NS                   16
DS                    4
SWH           12019.230 Hz
FIDRES         0.091699 Hz
AQ            5.4525952 sec
RG               196.14
DW               41.600 usec
DE                12.17 usec
TE                298.1 K
D1           0.10000000 sec
TD0                   1

======== CHANNEL f1 ========
SFO1        400.1324710 MHz
NUC1                 1H
P1                10.00 usec
PLW1        20.00000000 W

F2 - Processing parameters
SI               131072
SF          400.1300029 MHz
WDW                  EM
SSB      0
LB                 0.10 Hz
GB       0
PC                 1.00
```

PROTON256 DMSO {C:\Bruker\TopSpin3.2} IG500 23

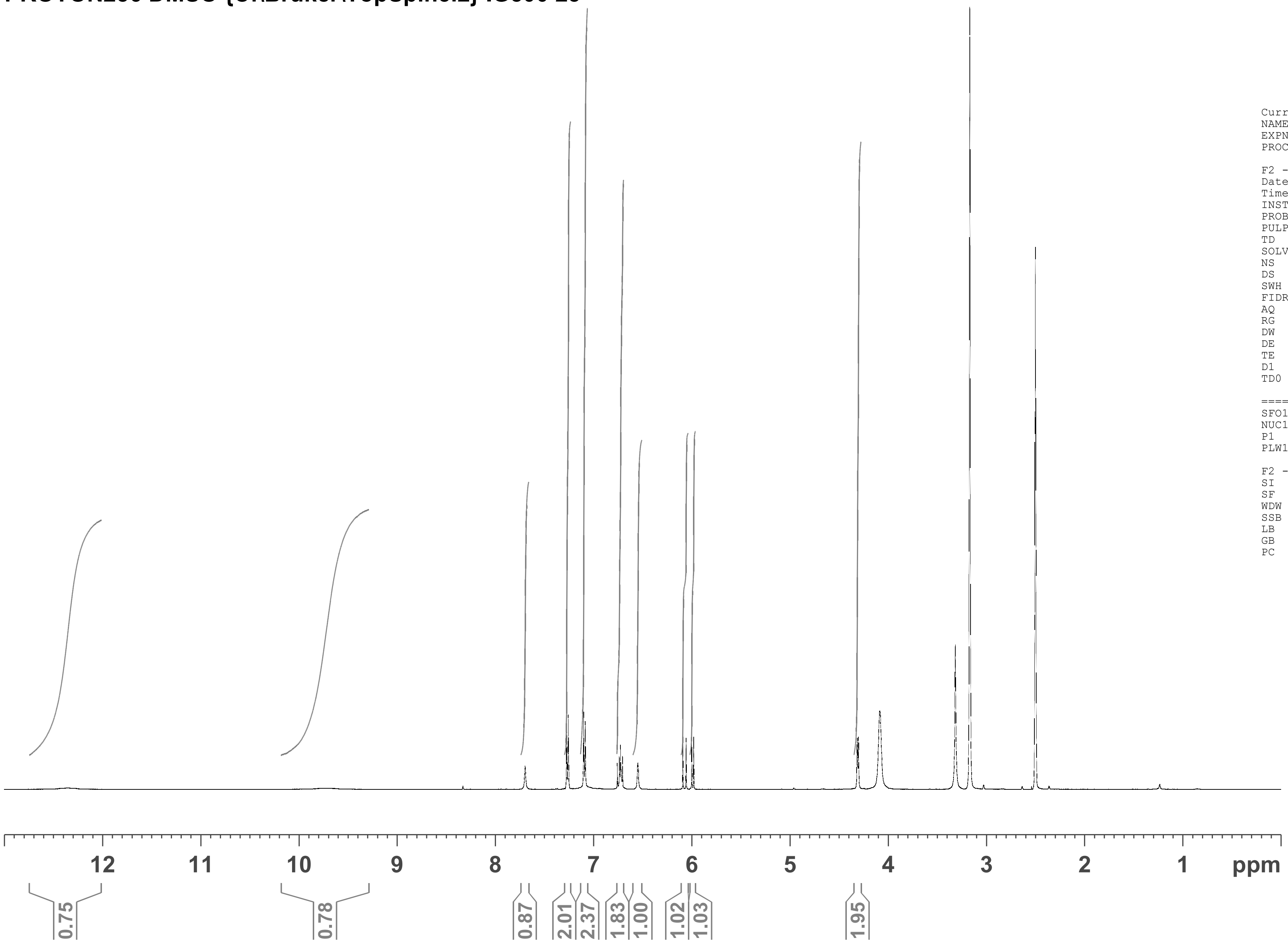


```
Current Data Parameters
NAME     IG-JM-200398-048-001
EXPNO                   1
PROCNO                  1

F2 - Acquisition Parameters
Date_            20161217
Time                12.06
INSTRUM             spect
PROBHD   5 mm QNP 1H/13
PULPROG              zg30
TD                  65536
SOLVENT              DMSO
NS                    256
DS                      2
SWH             10000.000 Hz
FIDRES           0.152588 Hz
AQ              3.2767999 sec
RG                     32
DW                 50.000 usec
DE                   6.50 usec
TE                  298.2 K
D1             1.00000000 sec
TD0                     1

======== CHANNEL f1 ========
SFO1        500.1330885 MHz
NUC1                   1H
P1                  10.00 usec
PLW1        25.00000000 W

F2 - Processing parameters
SI                  65536
SF          500.1295267 MHz
WDW                    EM
SSB      0
LB                   0.30 Hz
GB       0
PC                   1.00
```

Compound 28

Compound 28

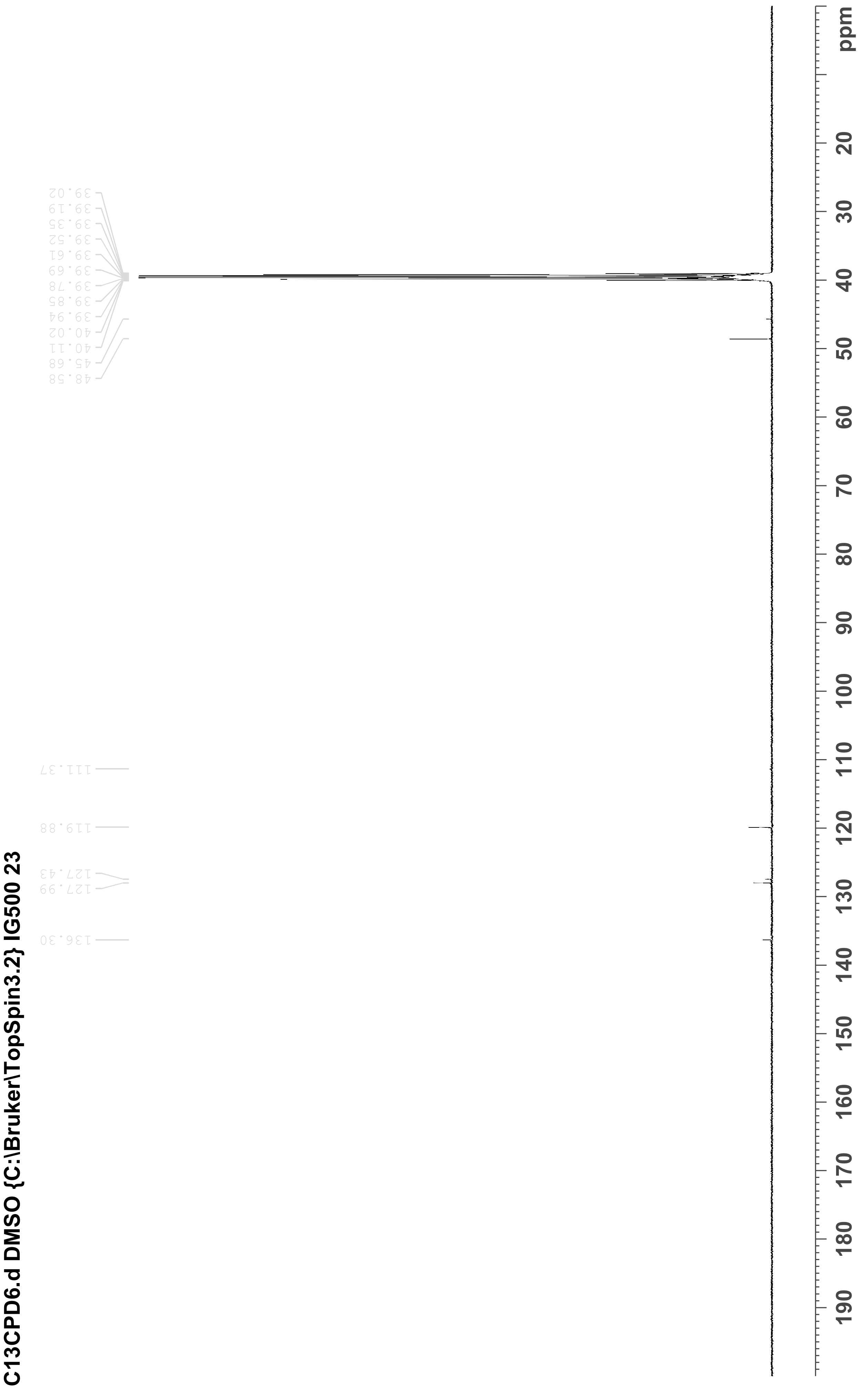
C13CPD6.d DMSO {C:\Bruker\TopSpin3.2} IG500 23
39.02
39.19
39.35
39.52
39.61
39.69
39.78
39.85
39.94
40.02
40.11
45.68
48.58
111.37
119.88
127.43
127.99
136.30
ppm
20
30
40
50
60
70
80
90
100
110
120
130
140
150
160
170
180
190

Compound 28

Compound Verification Report (Compass OpenAccess/QC)

Sample-ID	200398-048-001	Station	Microtof-2
Submitter	James Martin	Supervisor	System Administrator
Analysis Name	200398-048-001_10233_RB3_01_11133.d	Acquisition Date	09/01/2017 10:38:56
Sample Description		Method	2-microtof-2 verify compounds lcms pos 5-95.m

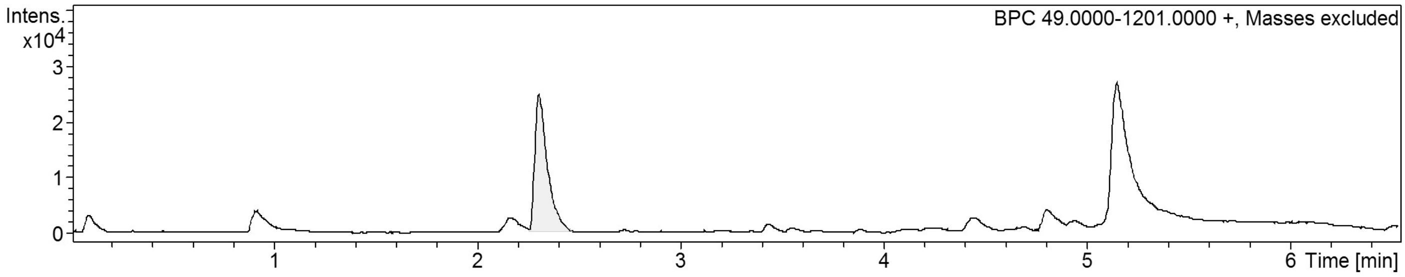

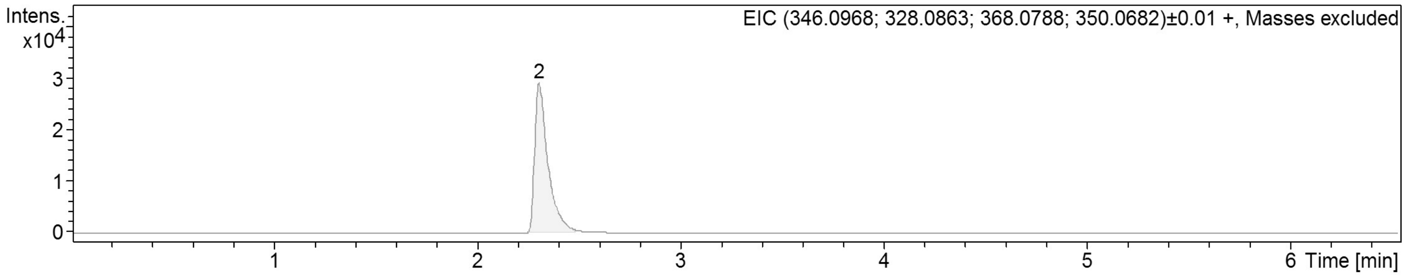

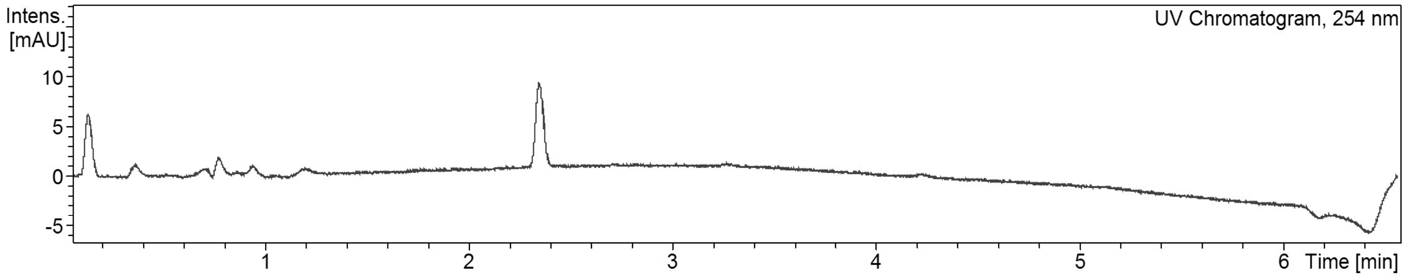

SmartFormula Settings

Tolerance	mSigma Limit	Electron Conf.
10 ppm	60	even

Adduction(s): H, Na **Neutral Loss(es):** H2O

Compound Verification Results

Expected Formula: C15H15N5O3S

#	meas. m/z	theo. m/z	|err| [ppm]	mSigma	Formula	Modification	Purity(UVC)[%]	Purity(BPC)[%]
2	346.0969	346.0968	0.2	1	C15 H16 N5 O3 S	(M+H)+	0.0	100.0

Note: mSigma values <20 indicate high probability of correct molecular formula

Compound Verification Report (Compass OpenAccess/QC)

Cmpd 2, 2.3 min

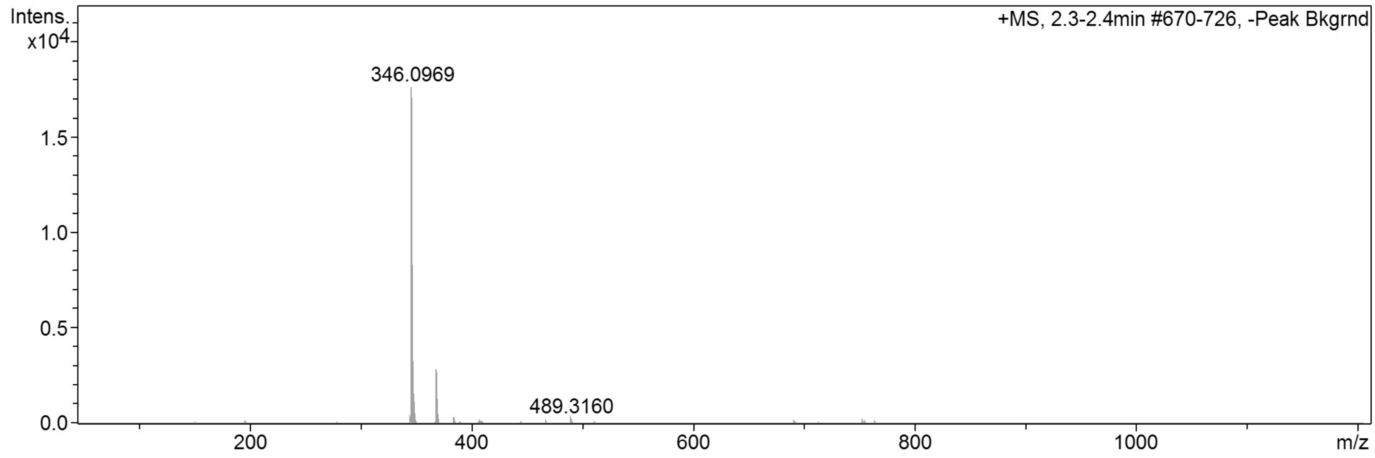

Compass OpenAccess LC-MS Identification Report

Sample-ID	200398-048-001	Station	Microtof-2
Submitter	James Martin	Supervisor	System Administrator
Analysis Name	200398-048-001_10239_RC1_01_11139.d	Acquisition Date	09/01/2017 11:31:58
Sample Description		Method	3-microtof-2 Identify Compounds BASIC LCMS Pos 5-95.m

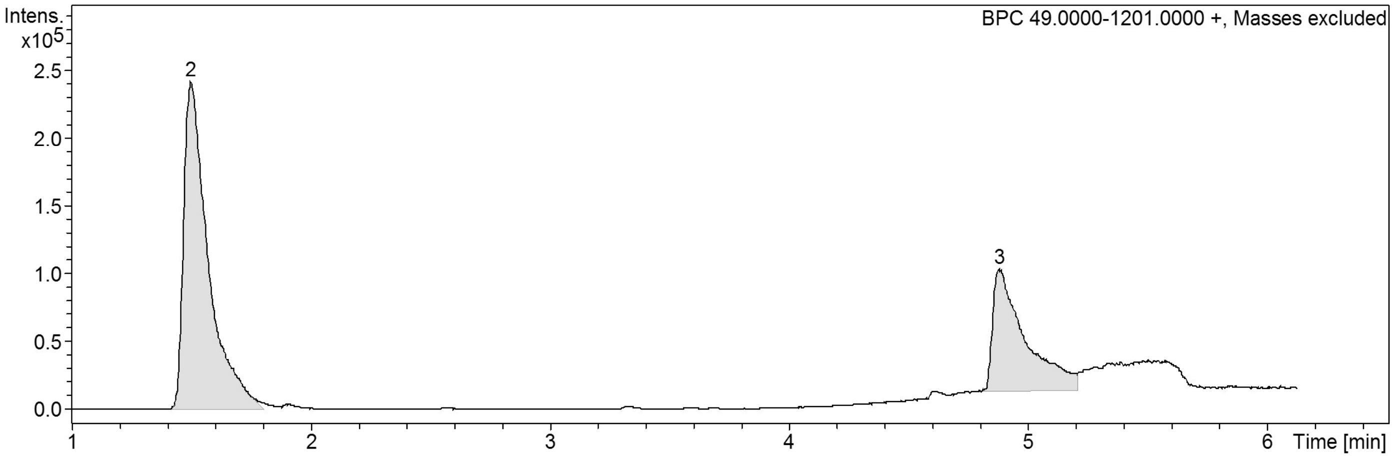

Sample Description		Method	3-microtof-2 Identify Compounds BASIC LCMS Pos 5-95.m

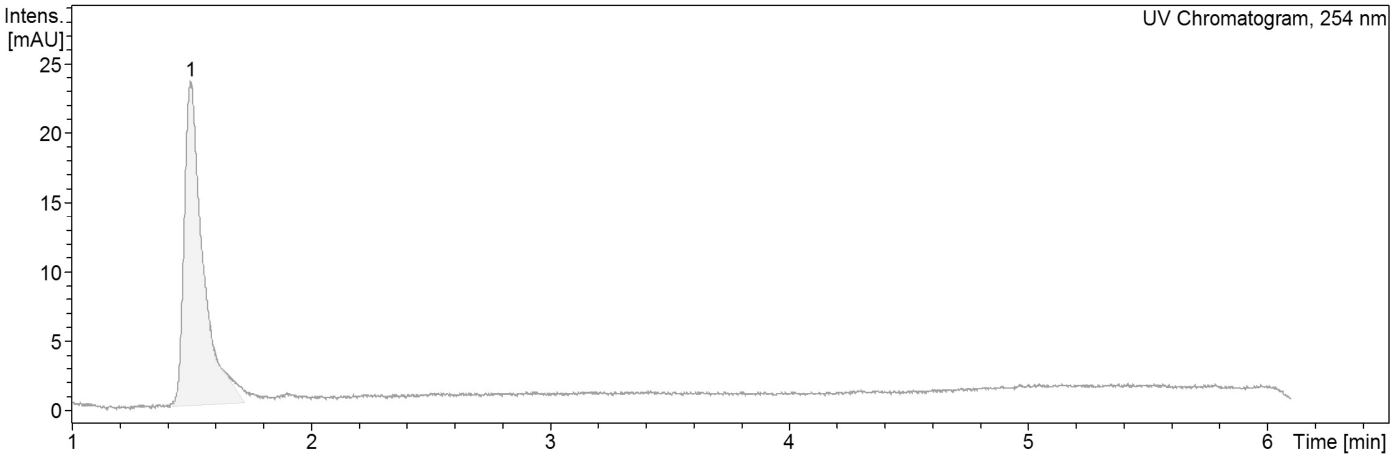

#	RT [min]	Area Frac. %	Chromatogram
1	1.5	100.000	UV Chromatogram, 254 nm
2	1.5	66.642	BPC 49.0000-1201.0000 +, Masses excluded
3	4.9	33.358	BPC 49.0000-1201.0000 +, Masses excluded

SmartFormula Settings

Tolerance	mSigma Limit	Electron Conf.
10 ppm	60	even

Compass OpenAccess LC-MS Identification Report

Calibration failed!

Adduction(s): **Neutral Loss(es):**

SmartFormula Results

FormulaMin: C1H1 **FormulaMax:** Na

#	meas. m/z	theo. m/z	|err| [ppm]	mSigma	Formula	Purity(UVC)[%]	Purity(BPC)[%]
2	345.1103	345.1097	1.7	-1,000	C20 H18 Na O4	100.0	66.6
		345.1111	2.2	-1,000	C21 H14 N4 Na		
		346.1034	6.0	23	C16 H16 N3 O6		
		346.1047	9.8	26	C17 H12 N7 O2		
3	282.2839	282.2864	8.5	29	C12 H36 N5 O2	0.0	33.4
		366.3340	3.0	15	C19 H40 N7		
		366.3316	9.5	17	C17 H41 N7 Na		
		563.5598	5.0	6	C33 H72 N4 Na O		
		563.5609	3.1	10	C34 H75 O5		

Note: mSigma values <30 indicate high probability of correct molecular formula

Cmpd 1, 1.5 min

Undefined context.
Component not placed in a spectrum iterator.

Cmpd 2, 1.5 min

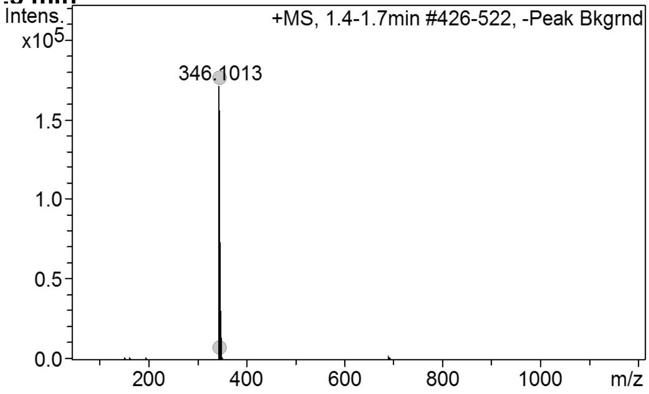

Cmpd 3, 4.9 min

Compass OpenAccess LC-MS Identification Report

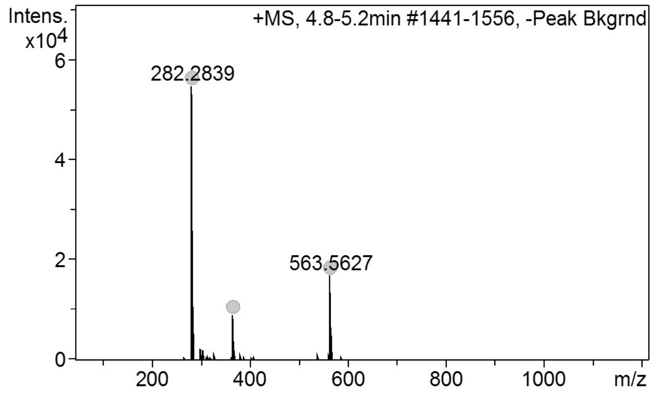

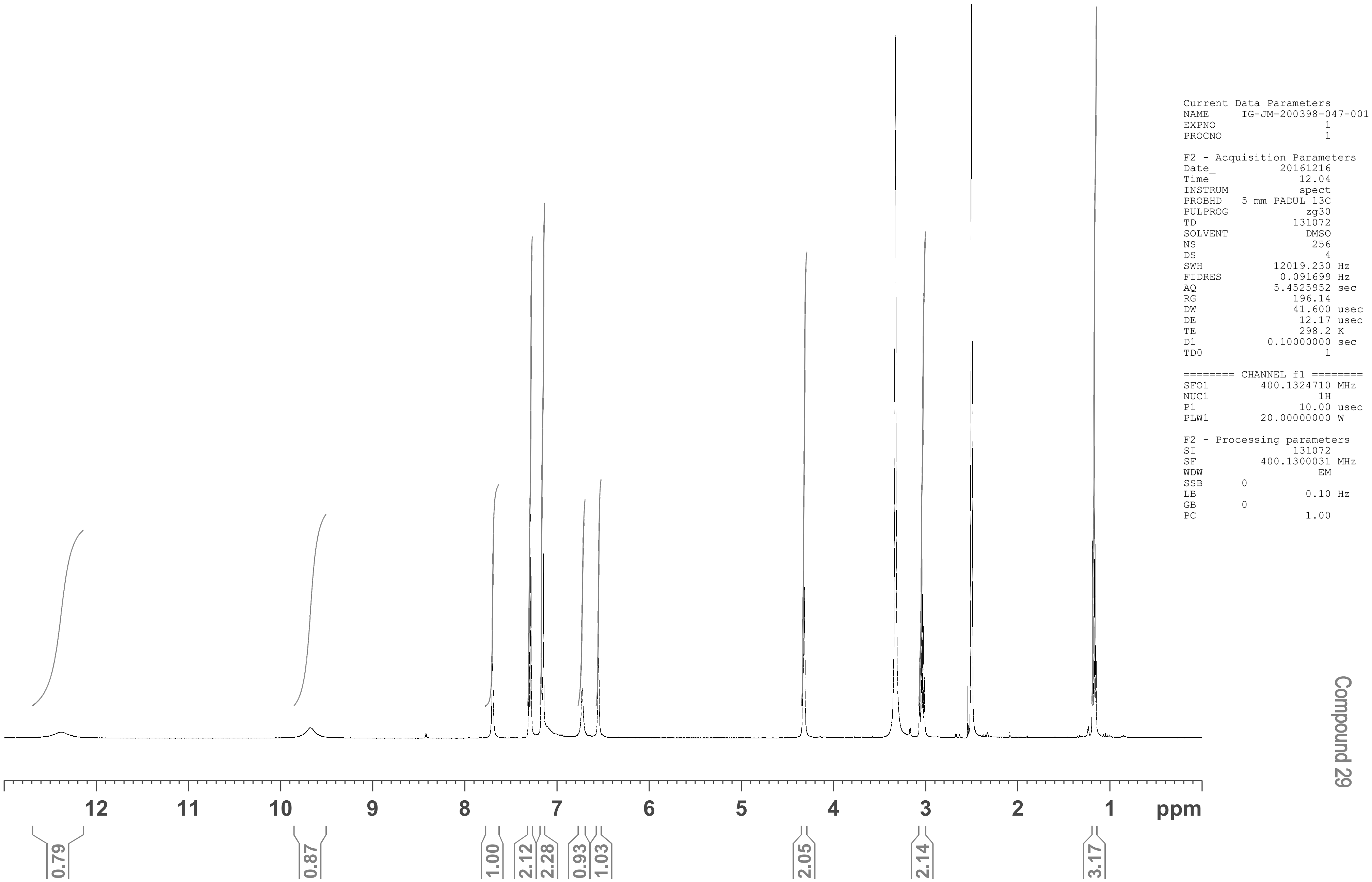
Current Data Parameters
NAME IG-JM-200398-047-001
EXPNO 1
PROCNO 1
F2 - Acquisition Parameters
Date_ 20161216
Time 12.04
INSTRUM spect
PROBHD 5 mm PADUL 13C
PULPROG zg30
TD 131072
SOLVENT DMSO
NS 256
DS 4
SWH 12019.230 Hz
FIDRES 0.091699 Hz
AQ 5.4525952 sec
RG 196.14
DW 41.600 usec
DE 12.17 usec
TE 298.2 K
D1 0.10000000 sec
TD0 1
======== CHANNEL f1 ========
SFO1 400.1324710 MHz
NUC1 1H
P1 10.00 usec
PLW1 20.00000000 W
F2 - Processing parameters
SI 131072
SF 400.1300031 MHz
WDW EM
SSB 0
LB 0.10 Hz
GB 0
PC 1.00
12
11
10
9
8
7
6
5
4
3
2
1
ppm
0.79
0.87
1.00
2.12
2.28
0.93
1.03
2.05
2.14
3.17

Compound 29

Compound 29

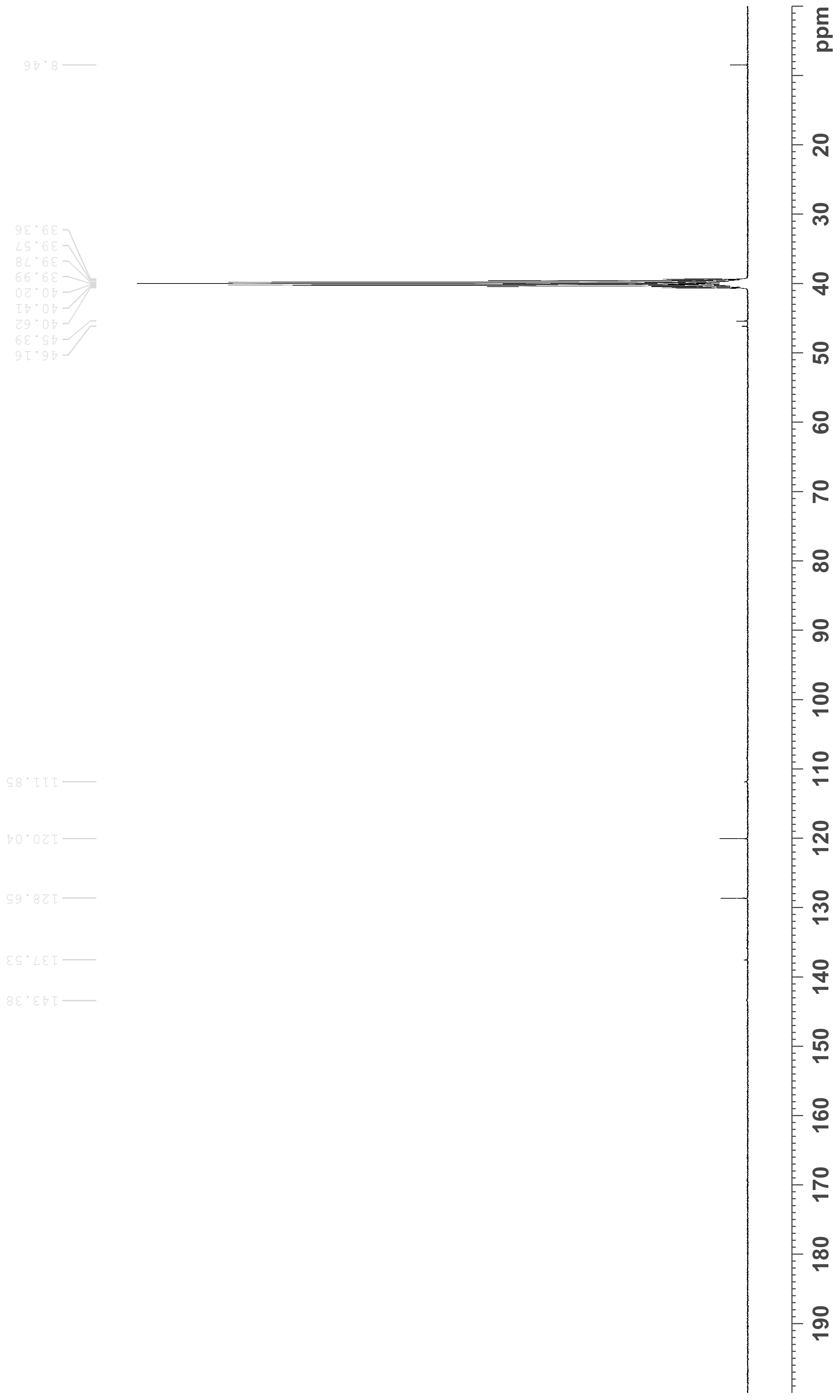

8.46
39.36
39.57
39.78
39.99
40.20
40.41
40.62
45.39
46.16
111.85
120.04
128.65
137.53
143.38
ppm
20
30
40
50
60
70
80
90
100
110
120
130
140
150
160
170
180
190

Compound Verification Report (Compass OpenAccess/QC)

Sample-ID	200398-047-001	Station	Microtof-2
Submitter	James Martin	Supervisor	System Administrator
Analysis Name	200398-047-001_10232_RB2_01_11132.d	Acquisition Date	09/01/2017 10:30:28
Sample Description		Method	2-microtof-2 verify compounds lcms pos 5-95.m

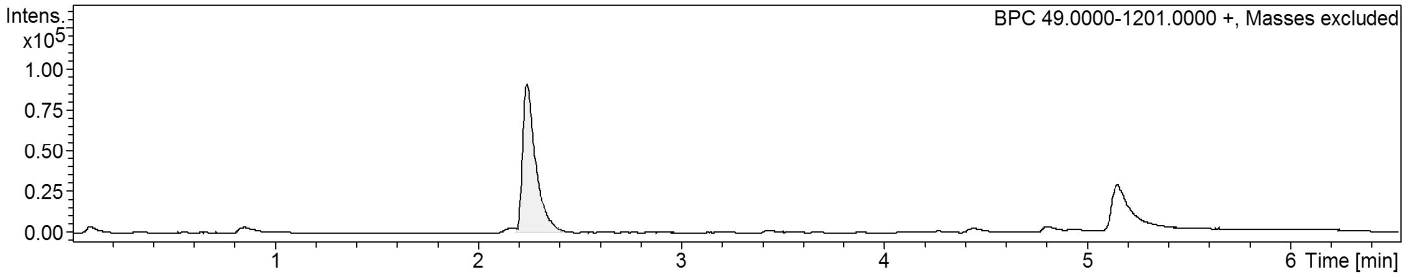

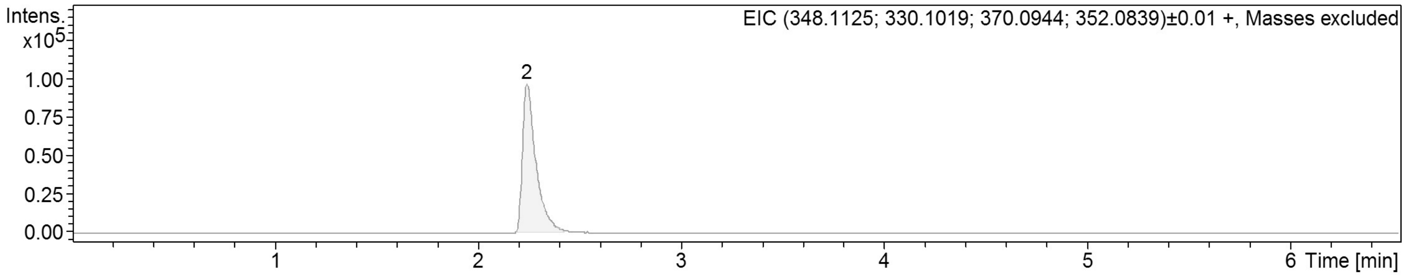

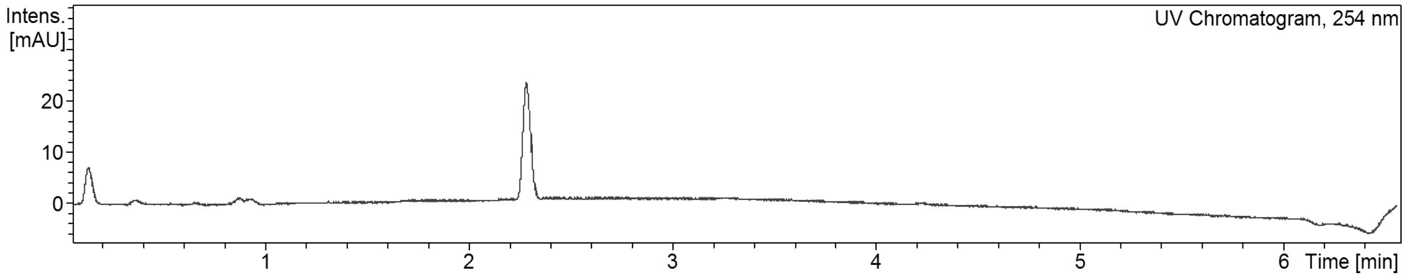

SmartFormula Settings

Tolerance	mSigma Limit	Electron Conf.
10 ppm	60	even

Adduction(s): H, Na **Neutral Loss(es):** H2O

Compound Verification Results

Expected Formula: C15H17N5O3S

#	meas. m/z	theo. m/z	|err| [ppm]	mSigma	Formula	Modification	Purity(UVC)[%]	Purity(BPC)[%]
2	348.1129	348.1125	1.1	4	C15 H18 N5 O3 S	(M+H)+	0.0	100.0

Note: mSigma values <20 indicate high probability of correct molecular formula

Compound Verification Report (Compass OpenAccess/QC)

Cmpd 2, 2.2 min

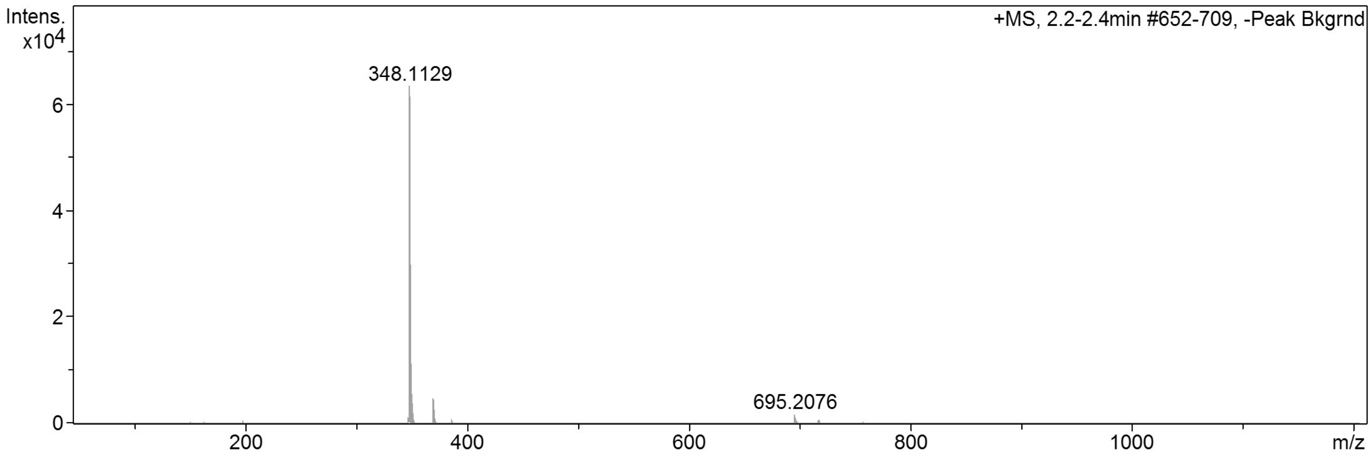

Compound 29

Compass OpenAccess LC-MS Identification Report

Sample-ID	200398-047-001	Station	Microtof-2
Submitter	James Martin	Supervisor	System Administrator
Analysis Name	200398-047-001_10238_RB8_01_11138.d	Acquisition Date	09/01/2017 11:24:05
Sample Description		Method	3-microtof-2 Identify Compounds BASIC LCMS Pos 5-95.m

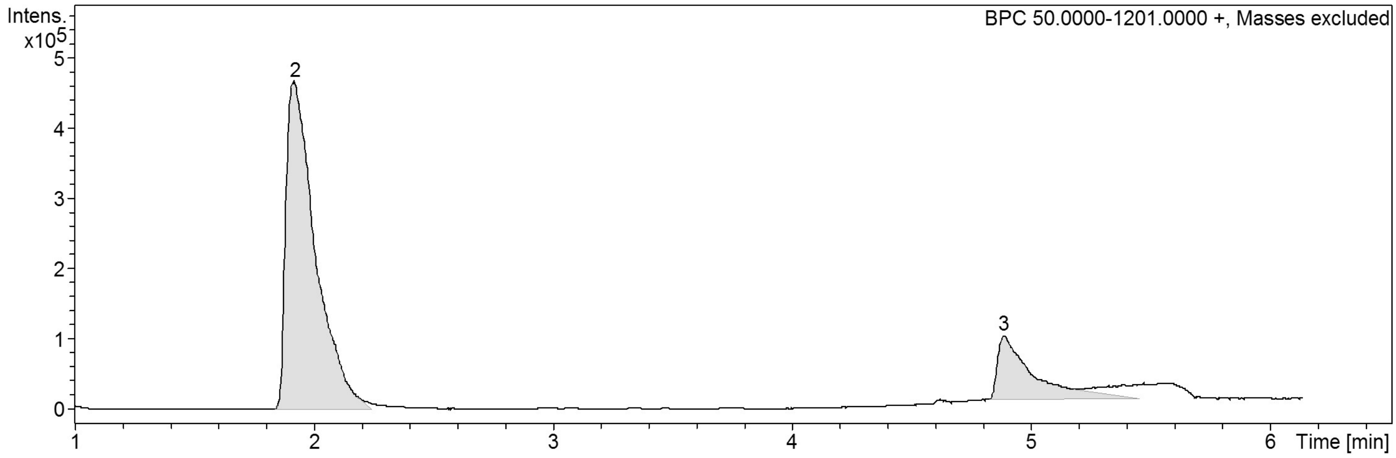

Sample Description		Method	3-microtof-2 Identify Compounds BASIC LCMS Pos 5-95.m

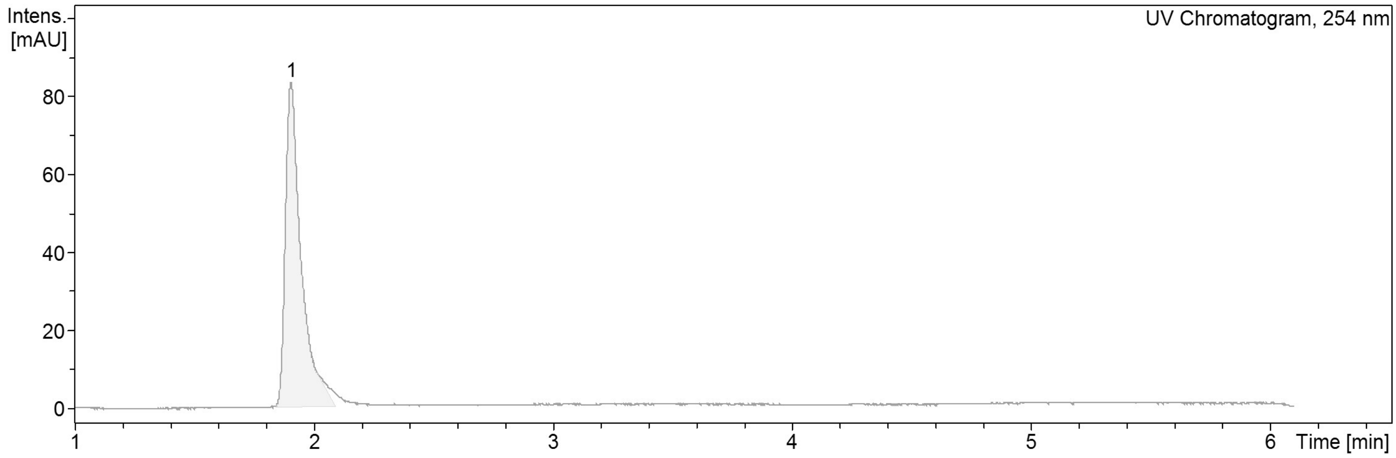

#	RT [min]	Area Frac. %	Chromatogram
1	1.9	100.00	UV Chromatogram, 254 nm
2	1.9	80.67	BPC 50.0000-1201.0000 +, Masses excluded
3	4.9	19.33	BPC 50.0000-1201.0000 +, Masses excluded

SmartFormula Settings

Tolerance	mSigma Limit	Electron Conf.
10 ppm	60	even

Compass OpenAccess LC-MS Identification Report

Calibration failed!

Adduction(s): **Neutral Loss(es):**

SmartFormula Results

FormulaMin: C1H1 **FormulaMax:** Na

#	meas. m/z	theo. m/z	|err| [ppm]	mSigma	Formula	Purity(UVC)[%]	Purity(BPC)[%]
2	348.1180	348.1206	7.6	25	C19 H19 N Na O4	100.0	80.7
		348.1203	6.8	27	C17 H14 N7 O2		
		695.2334	4.7	37	C36 H39 O14		
		695.2348	2.7	41	C37 H35 N4 O10		
3	282.2840	282.2864	8.3	30	C12 H36 N5 O2	0.0	19.3
		366.3340	3.9	16	C19 H40 N7		
		366.3343	3.1	18	C21 H45 N Na O2		
		563.5609	3.4	2	C34 H75 O5		
		563.5598	5.3	5	C33 H72 N4 Na O		

Note: mSigma values <30 indicate high probability of correct molecular formula

Cmpd 1, 1.9 min

Undefined context.
Component not placed in a spectrum iterator.

Cmpd 2, 1.9 min

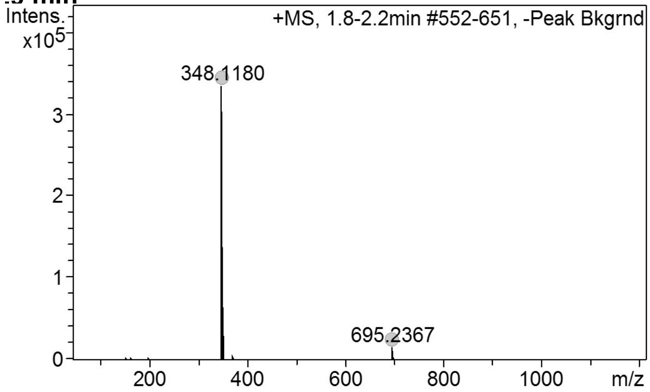

Cmpd 3, 4.9 min

Compass OpenAccess LC-MS Identification Report

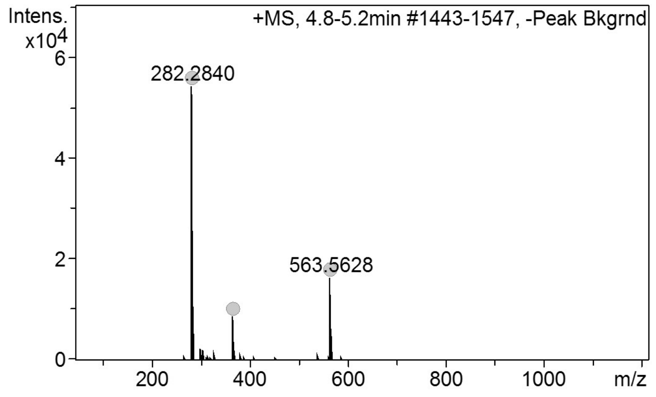

Compound 34

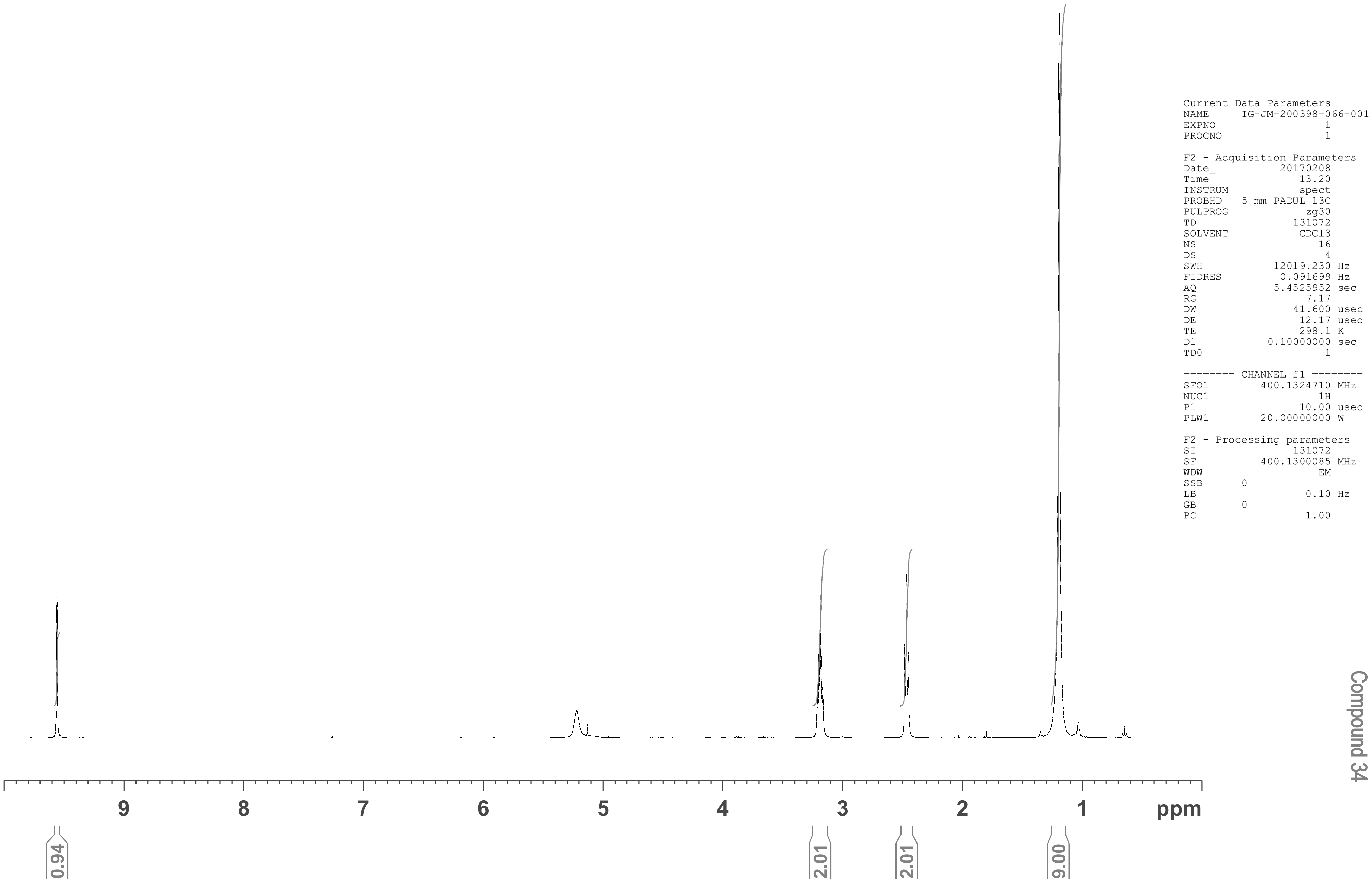
Current Data Parameters
NAME IG-JM-200398-066-001
EXPNO 1
PROCNO 1
F2 - Acquisition Parameters
Date_ 20170208
Time 13.20
INSTRUM spect
PROBHD 5 mm PADUL 13C
PULPROG zg30
TD 131072
SOLVENT CDCl3
NS 16
DS 4
SWH 12019.230 Hz
FIDRES 0.091699 Hz
AQ 5.4525952 sec
RG 7.17
DW 41.600 usec
DE 12.17 usec
TE 298.1 K
D1 0.10000000 sec
TD0 1
======== CHANNEL f1 ========
SFO1 400.1324710 MHz
NUC1 1H
P1 10.00 usec
PLW1 20.00000000 W
F2 - Processing parameters
SI 131072
SF 400.1300085 MHz
WDW EM
SSB 0
LB 0.10 Hz
GB 0
PC 1.00
9
8
7
6
5
4
3
2
1
ppm
0.94
2.01
2.01
9.00

PROTON.d CDCl3 {C:\Bruker\TopSpin3.2} IG500 17

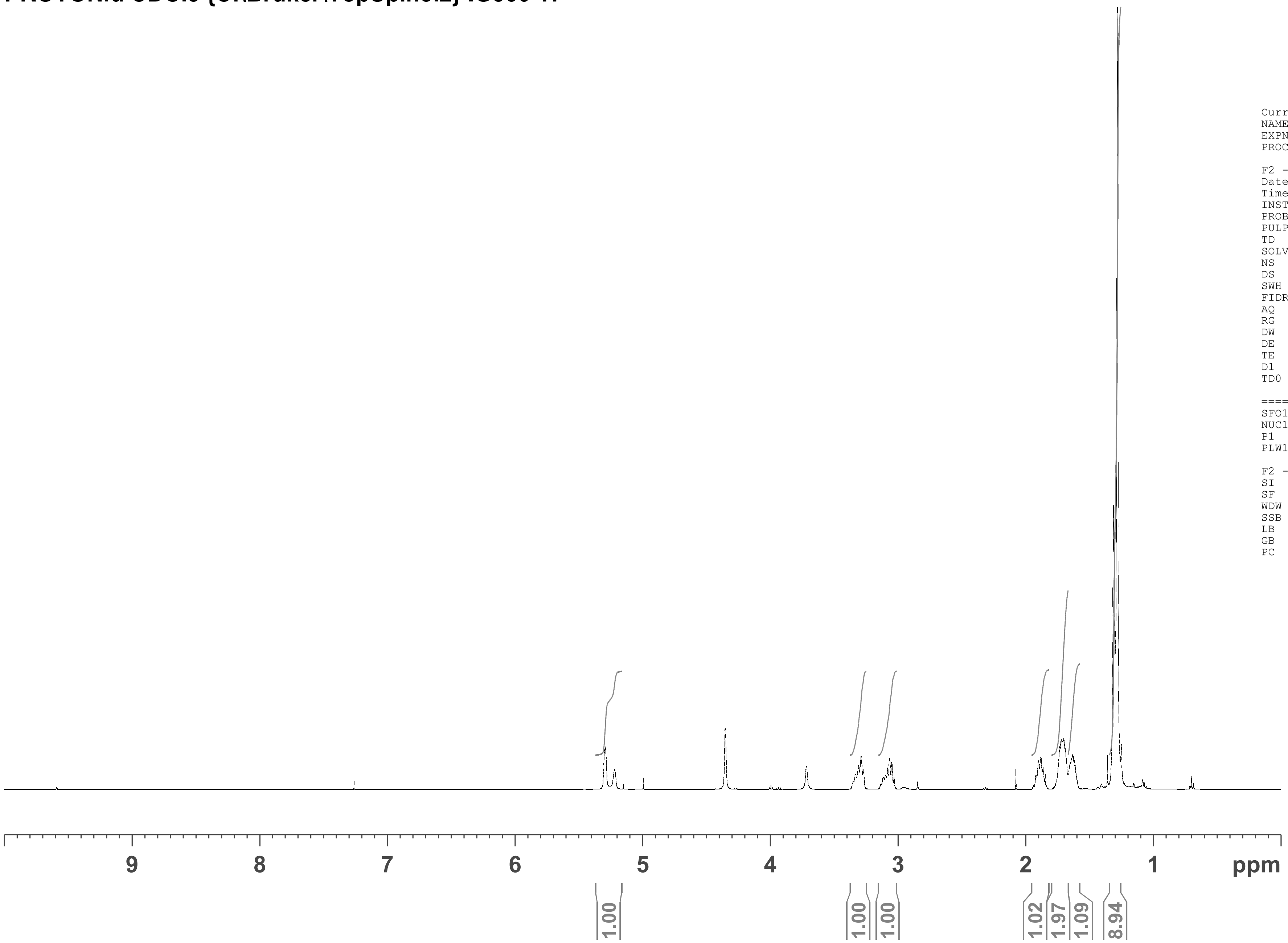


```
Current Data Parameters
NAME     IG-JM-200398-068-P
EXPNO                    1
PROCNO                   1

F2 - Acquisition Parameters
Date_          20170220
Time              11.41
INSTRUM           spect
PROBHD   5 mm QNP 1H/13
PULPROG            zg30
TD                65536
SOLVENT           CDCl3
NS                   16
DS                    2
SWH           10000.000 Hz
FIDRES         0.152588 Hz
AQ            3.2767999 sec
RG                 14.2
DW               50.000 usec
DE                 6.50 usec
TE                298.2 K
D1           1.00000000 sec
TD0                   1

======== CHANNEL f1 ========
SFO1        500.1330885 MHz
NUC1                 1H
P1                10.00 usec
PLW1        25.00000000 W

F2 - Processing parameters
SI                65536
SF          500.1300117 MHz
WDW                  EM
SSB      0
LB                 0.30 Hz
GB       0
PC                 1.00
```

Compound 35

PROTON.d CDCl3 {C:\Bruker\TopSpin3.2} IG500 19

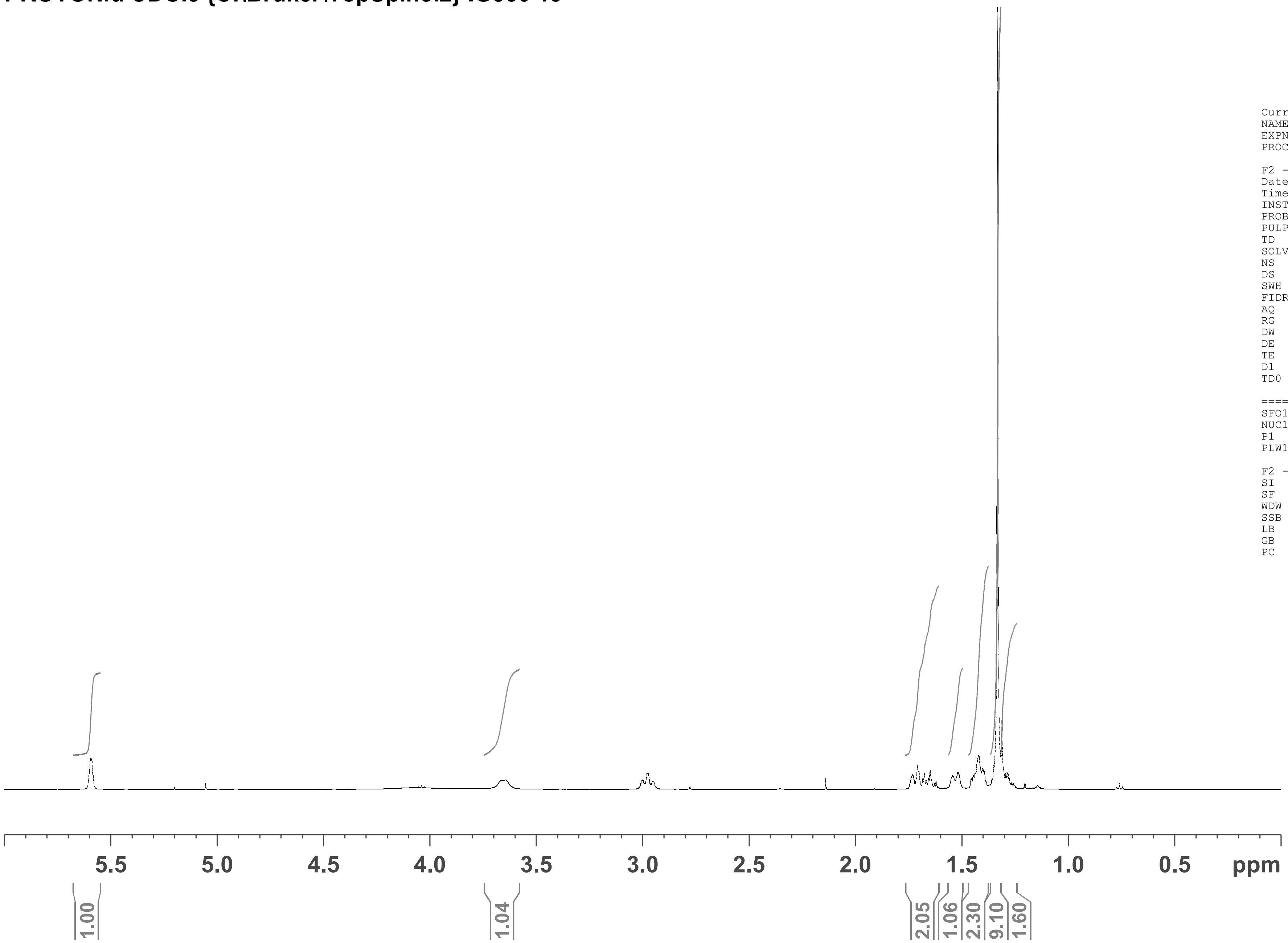


```
Current Data Parameters
NAME      IG-JM-200398-075-P
EXPNO                    1
PROCNO                   1

F2 - Acquisition Parameters
Date_           20170220
Time               13.19
INSTRUM            spect
PROBHD   5 mm QNP 1H/13
PULPROG             zg30
TD                 65536
SOLVENT            CDCl3
NS                    16
DS                     2
SWH            10000.000 Hz
FIDRES          0.152588 Hz
AQ             3.2767999 sec
RG                  14.2
DW                50.000 usec
DE                  6.50 usec
TE                 298.2 K
D1            1.00000000 sec
TD0                    1

======== CHANNEL f1 ========
SFO1         500.1330885 MHz
NUC1                  1H
P1                 10.00 usec
PLW1         25.00000000 W

F2 - Processing parameters
SI                 65536
SF           500.1300000 MHz
WDW                   EM
SSB      0
LB                  0.30 Hz
GB       0
PC                  1.00
```

Compound 36

PROTON.d CDCl3 {C:\Bruker\TopSpin3.2} IG500 8

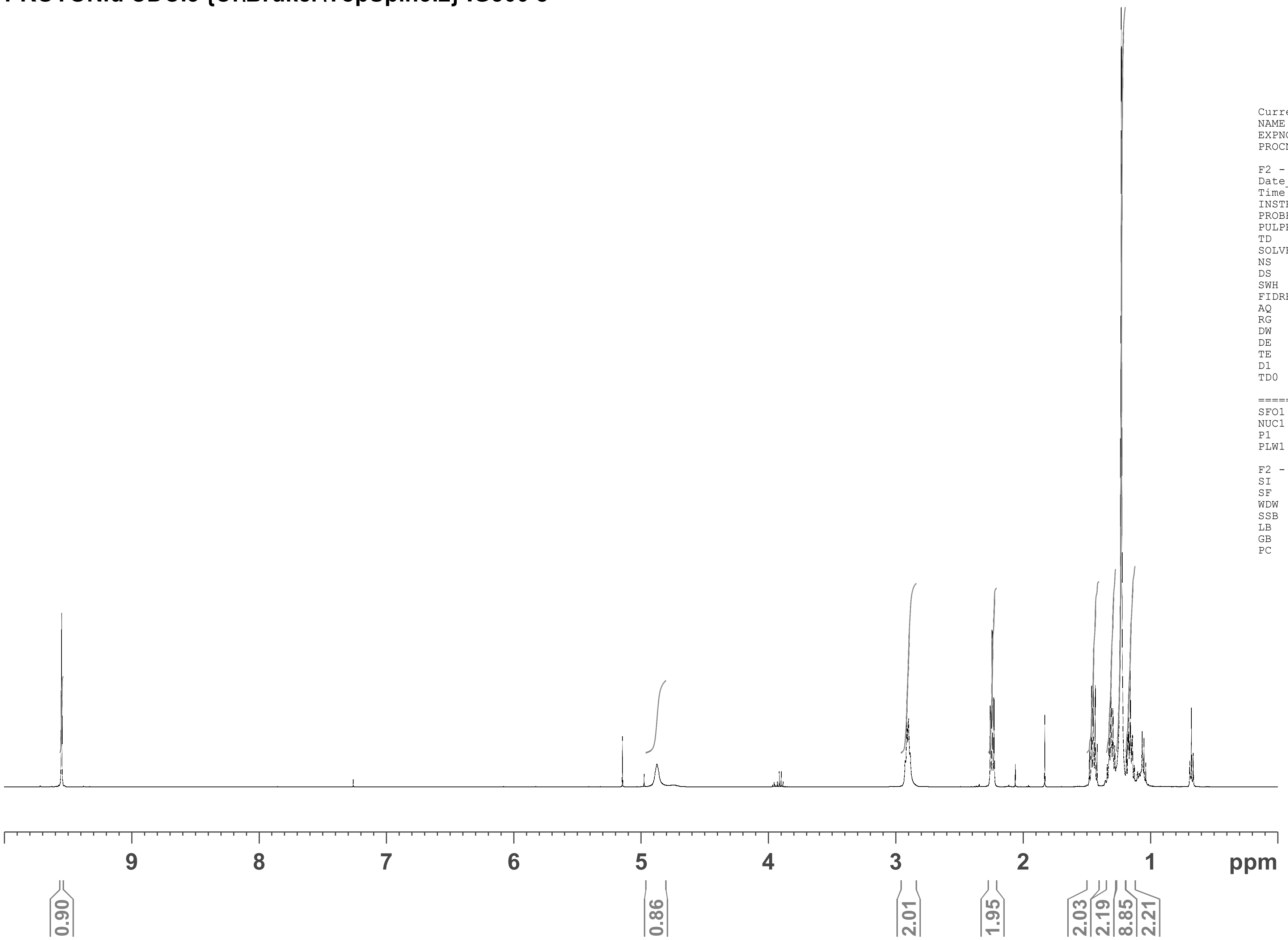


```
Current Data Parameters
NAME     IG-JM-200398-069-001
EXPNO                    1
PROCNO                   1

F2 - Acquisition Parameters
Date_           20170209
Time               13.48
INSTRUM            spect
PROBHD   5 mm QNP 1H/13
PULPROG             zg30
TD                 65536
SOLVENT            CDCl3
NS                    16
DS                     2
SWH          10000.000 Hz
FIDRES        0.152588 Hz
AQ           3.2767999 sec
RG                  12.7
DW              50.000 usec
DE                6.50 usec
TE               298.2 K
D1          1.00000000 sec
TD0                    1

======== CHANNEL f1 ========
SFO1        500.1330885 MHz
NUC1                 1H
P1                10.00 usec
PLW1        25.00000000 W

F2 - Processing parameters
SI                 65536
SF          500.1300117 MHz
WDW                   EM
SSB      0
LB                  0.30 Hz
GB       0
PC                  1.00
```

Compound 37

PROTON.d MeOD {C:\Bruker\TopSpin3.2} IG500 22

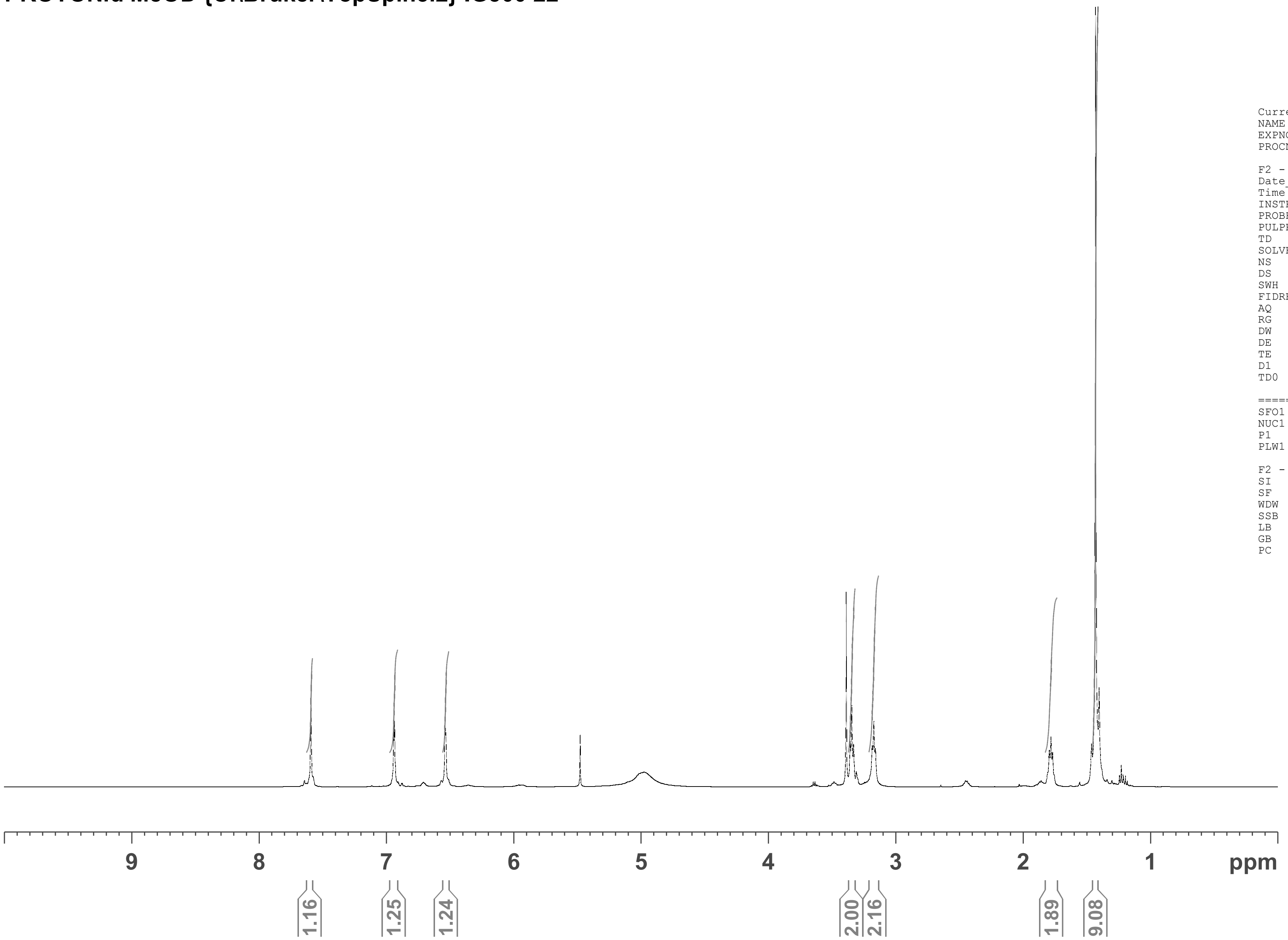


```
Current Data Parameters
NAME     IG-JM-200398-088-001
EXPNO                   1
PROCNO                  1

F2 - Acquisition Parameters
Date_           20170307
Time               13.05
INSTRUM            spect
PROBHD   5 mm QNP 1H/13
PULPROG             zg30
TD                 65536
SOLVENT             MeOD
NS                    16
DS                     2
SWH            10000.000 Hz
FIDRES          0.152588 Hz
AQ             3.2767999 sec
RG                  28.5
DW                50.000 usec
DE                  6.50 usec
TE                 298.2 K
D1            1.00000000 sec
TD0                    1

======== CHANNEL f1 ========
SFO1        500.1330885 MHz
NUC1                  1H
P1                 10.00 usec
PLW1        25.00000000 W

F2 - Processing parameters
SI                 65536
SF          500.1307313 MHz
WDW                   EM
SSB      0
LB                  0.30 Hz
GB       0
PC                  1.00
```

Compound 38

C13CPD.d MeOD {C:\Bruker\TopSpin3.2} IG500 22

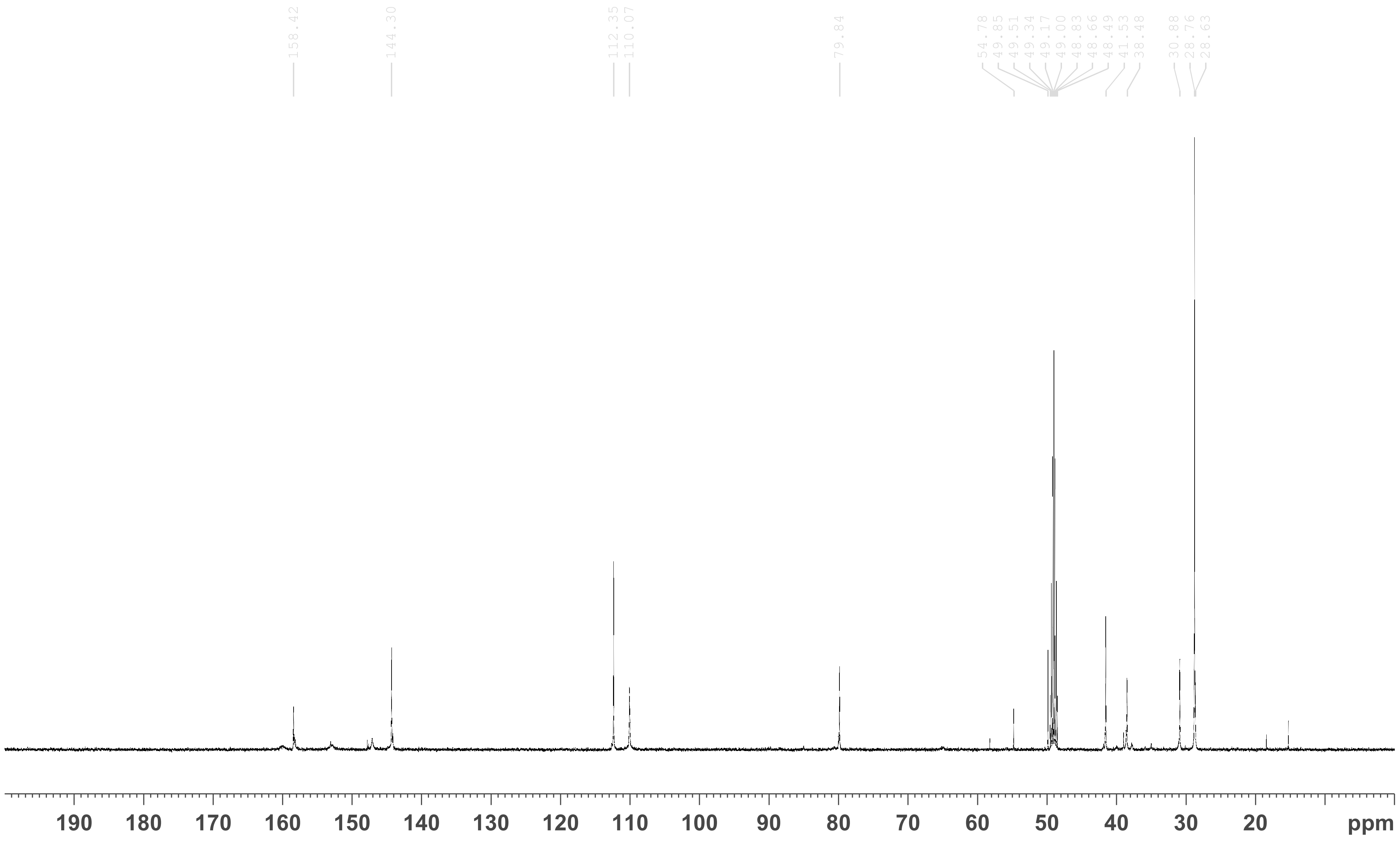

Compound 38

Analytical Studio Reviewer Report

Sample Name: JM-200398-088-T57
Location: 1,1:C,8
Acquired: 07-Mar-17 11:22 AM
Filename: JM-200398-088-T57_009113.D
Instrument: 5-90 MECN POS EZX_3M.M

Compound 38

Peak #	Time	Area % TIC(+)	Area % UV254	BPM
1	1.290	0.0	100.0	308.2
2	1.333	100.0	0.0	308.2

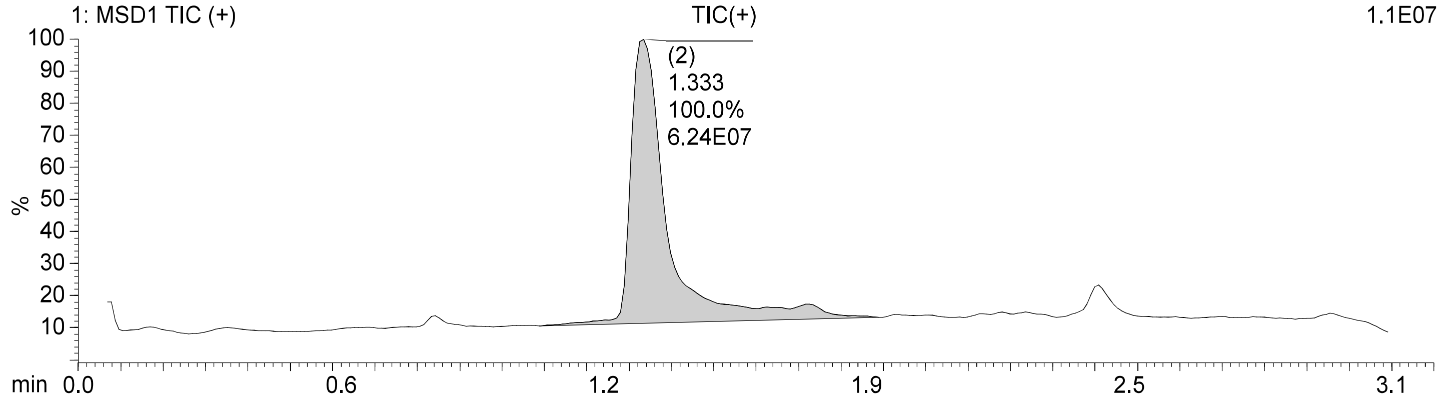

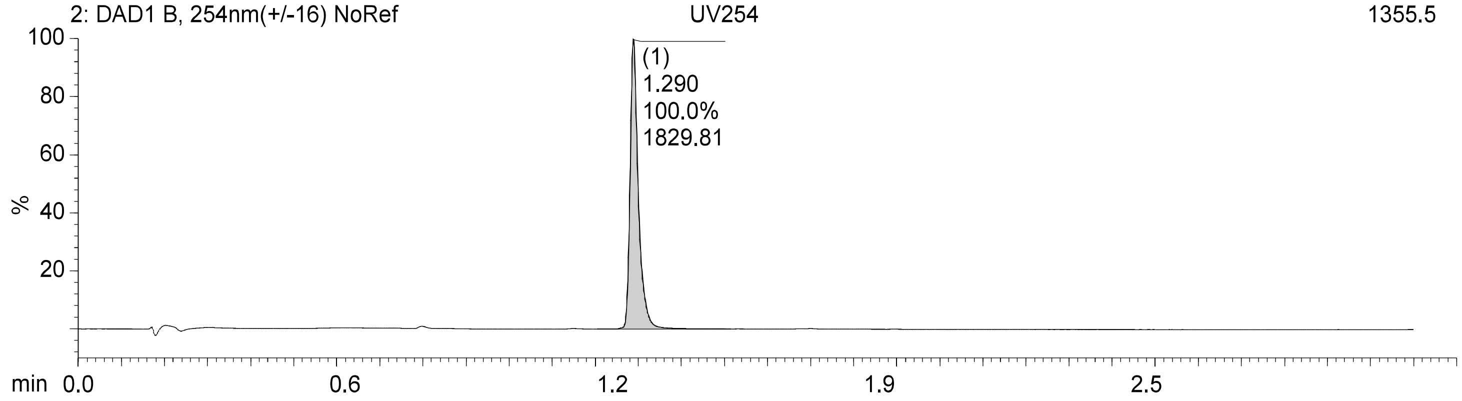

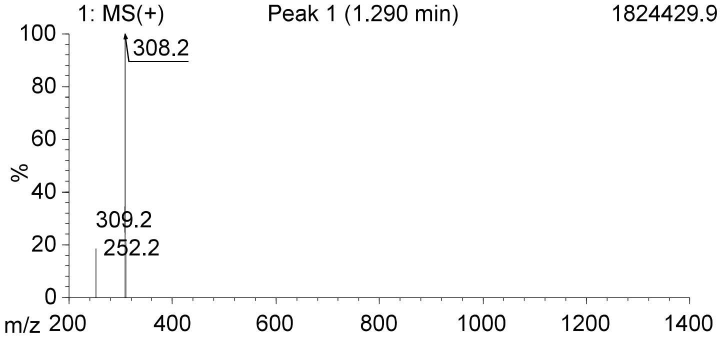

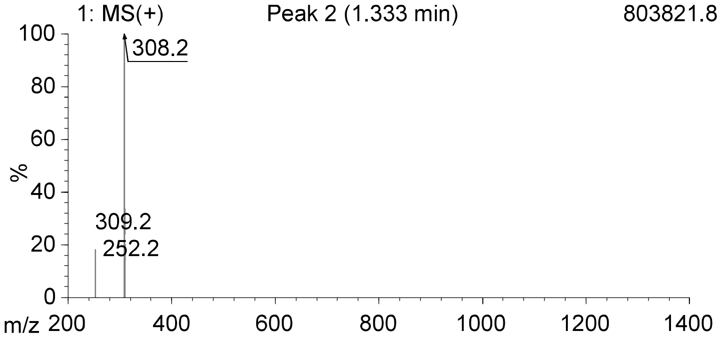

Page 1 of 1

Printed: 07-Mar-17 11:26 AM

Compound 39

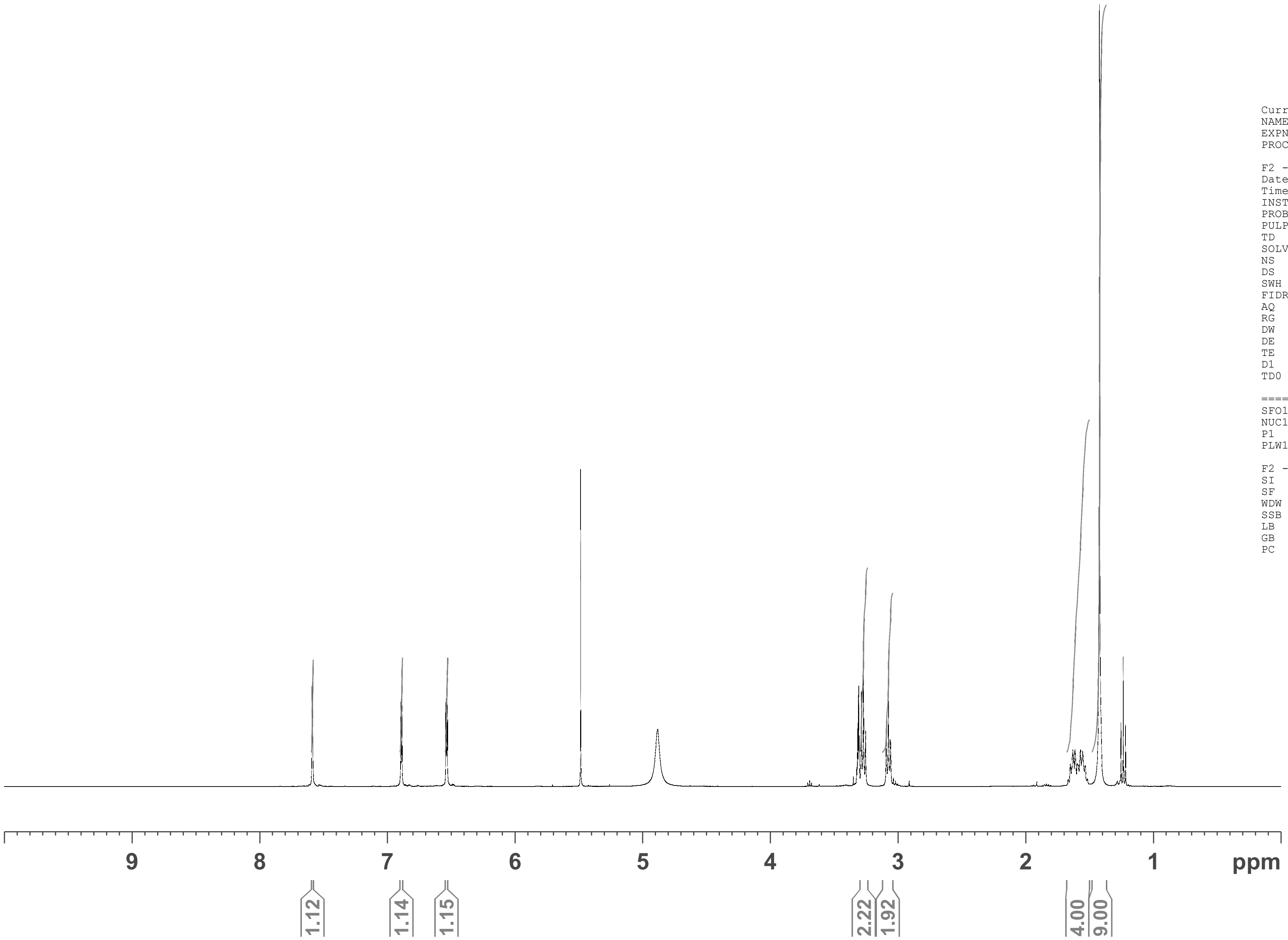


```
Current Data Parameters
NAME     IG-JM-200398-090-P
EXPNO                 1
PROCNO                1

F2 - Acquisition Parameters
Date_          20170309
Time              15.23
INSTRUM           spect
PROBHD   5 mm PADUL 13C
PULPROG            zg30
TD               131072
SOLVENT            MeOD
NS                   16
DS                    4
SWH           12019.230 Hz
FIDRES         0.091699 Hz
AQ            5.4525952 sec
RG                77.74
DW               41.600 usec
DE                12.17 usec
TE                298.2 K
D1           0.10000000 sec
TD0                   1

======== CHANNEL f1 ========
SFO1        400.1324710 MHz
NUC1                 1H
P1                10.00 usec
PLW1        20.00000000 W

F2 - Processing parameters
SI               131072
SF          400.1300077 MHz
WDW                  EM
SSB      0
LB                 0.10 Hz
GB       0
PC                 1.00
```

PROTON.d CDCl3 {C:\Bruker\TopSpin3.2} IG500 29

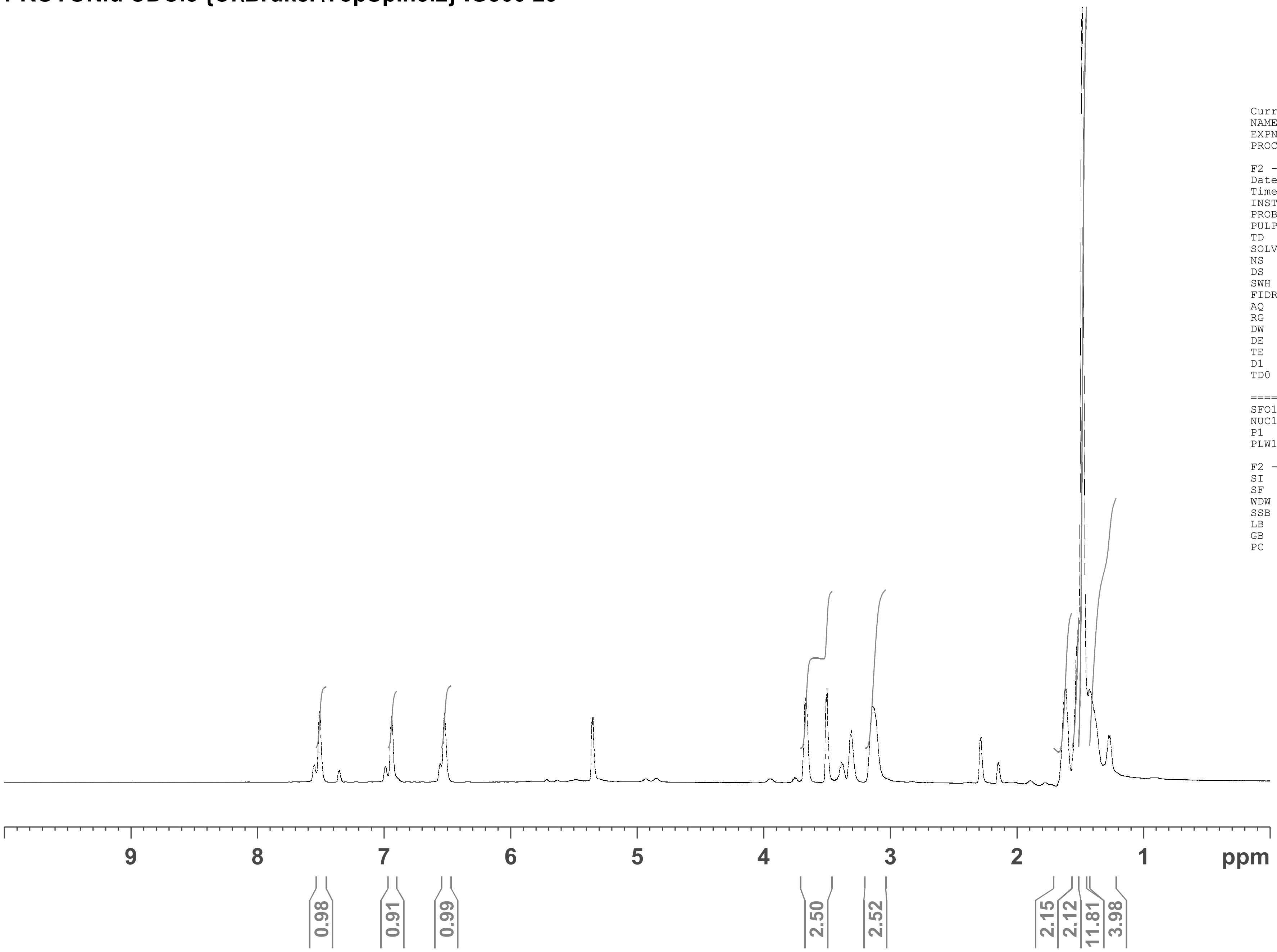


```
Current Data Parameters
NAME     IG-JM-200398-081-P1
EXPNO                      1
PROCNO                     1

F2 - Acquisition Parameters
Date_           20170224
Time               12.10
INSTRUM            spect
PROBHD   5 mm QNP 1H/13
PULPROG             zg30
TD                 65536
SOLVENT            CDCl3
NS                    16
DS                     2
SWH            10000.000 Hz
FIDRES          0.152588 Hz
AQ             3.2767999 sec
RG                  90.5
DW                50.000 usec
DE                  6.50 usec
TE                 298.2 K
D1            1.00000000 sec
TD0                    1

======== CHANNEL f1 ========
SFO1         500.1330885 MHz
NUC1                  1H
P1                 10.00 usec
PLW1         25.00000000 W

F2 - Processing parameters
SI                 65536
SF           500.1314040 MHz
WDW                   no
SSB      0
LB       0 Hz
GB       0
PC                  1.00
```

Compound 40

PROTON.d MeOD {C:\Bruker\TopSpin3.2} IG500 32

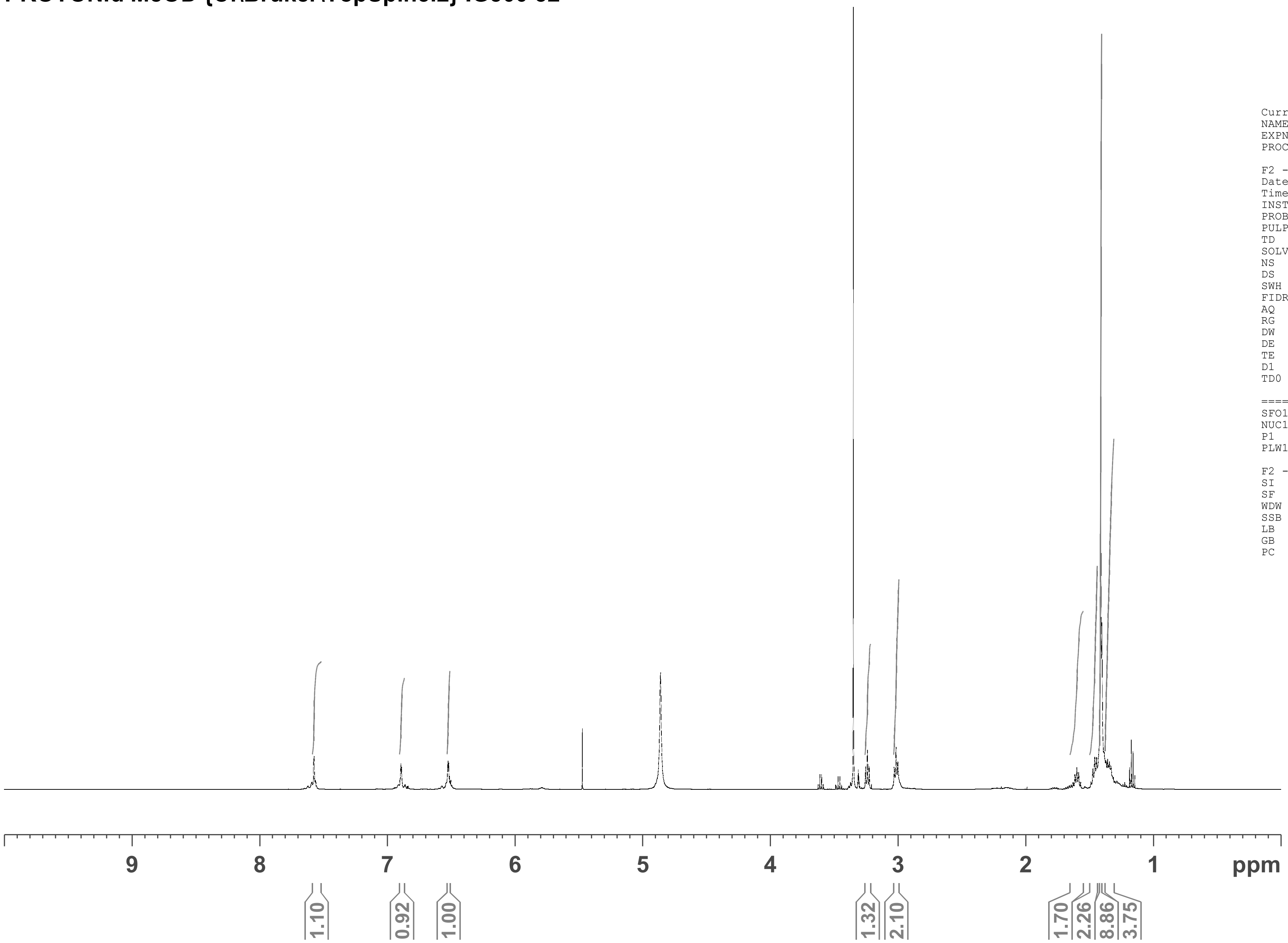


```
Current Data Parameters
NAME     IG-JM-200398-073-001
EXPNO                   1
PROCNO                  1

F2 - Acquisition Parameters
Date_          20170216
Time              13.03
INSTRUM           spect
PROBHD   5 mm QNP 1H/13
PULPROG            zg30
TD                65536
SOLVENT            MeOD
NS                   16
DS                    2
SWH           10000.000 Hz
FIDRES         0.152588 Hz
AQ            3.2767999 sec
RG                   57
DW               50.000 usec
DE                 6.50 usec
TE                298.2 K
D1           1.00000000 sec
TD0                   1

======== CHANNEL f1 ========
SFO1        500.1330885 MHz
NUC1                 1H
P1                10.00 usec
PLW1        25.00000000 W

F2 - Processing parameters
SI                65536
SF          500.1307511 MHz
WDW                  EM
SSB      0
LB                 0.30 Hz
GB       0
PC                 1.00
```

Compound 41

Compound 41

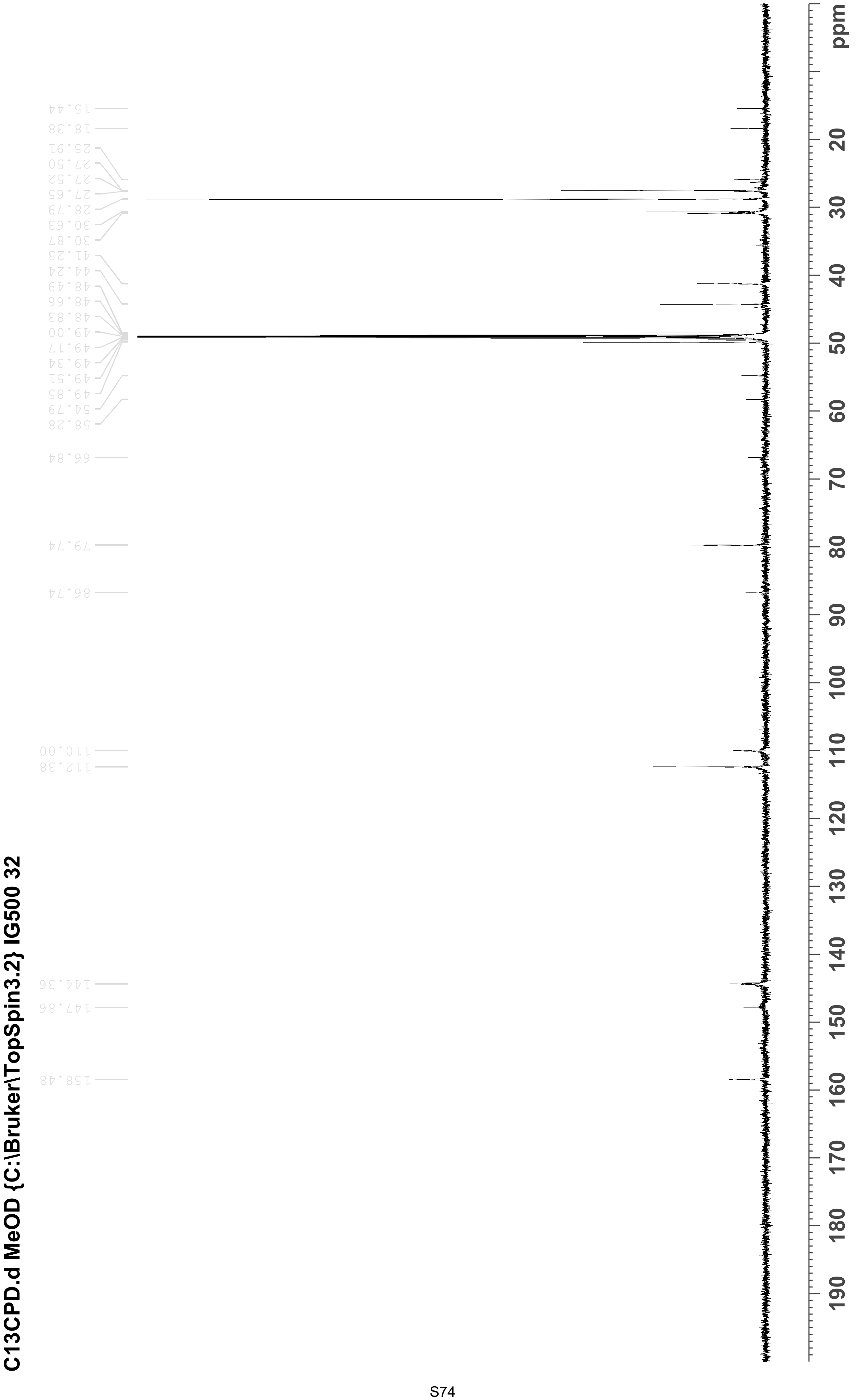
C13CPD.d MeOD {C:\Bruker\TopSpin3.2} IG500 32
15.44
18.38
25.91
27.50
27.52
27.65
28.79
30.63
30.87
41.23
44.24
48.49
48.66
48.83
49.00
49.17
49.34
49.51
49.85
54.79
58.28
66.84
79.74
86.74
110.00
112.38
144.36
147.86
158.48
ppm
20 30 40 50 60 70 80 90 100 110 120 130 140 150 160 170 180 190

Compound 41

Data Analysis Report

Analysis Info

Analysis Name	D:\Data\james\JM-200398-073-P_8043_1-E,7_01_12564.d	Acquisition Date	16/02/2017 12:31:13
Method	01-microtof-1-Identify Compounds LCMS Pos 5-95.m	Operator	Dundee
Sample Name	JM-200398-073-P_8043	Instrument / Ser#	micrOTOF 213750.00101
Comment			

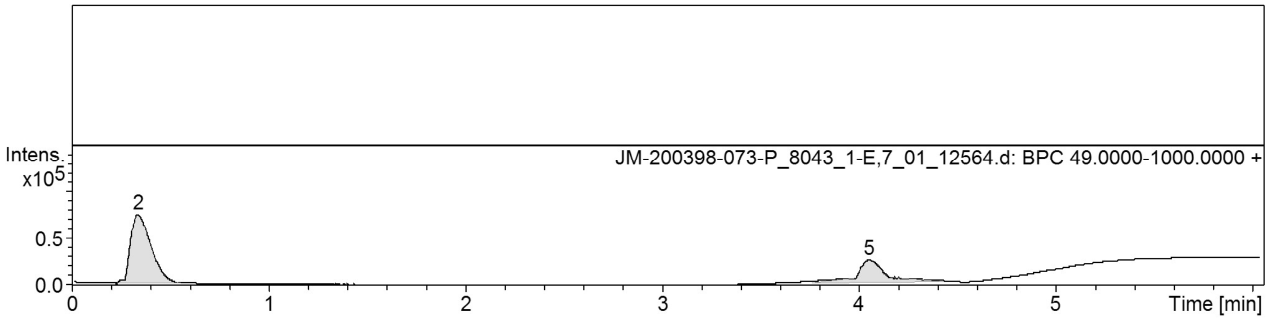

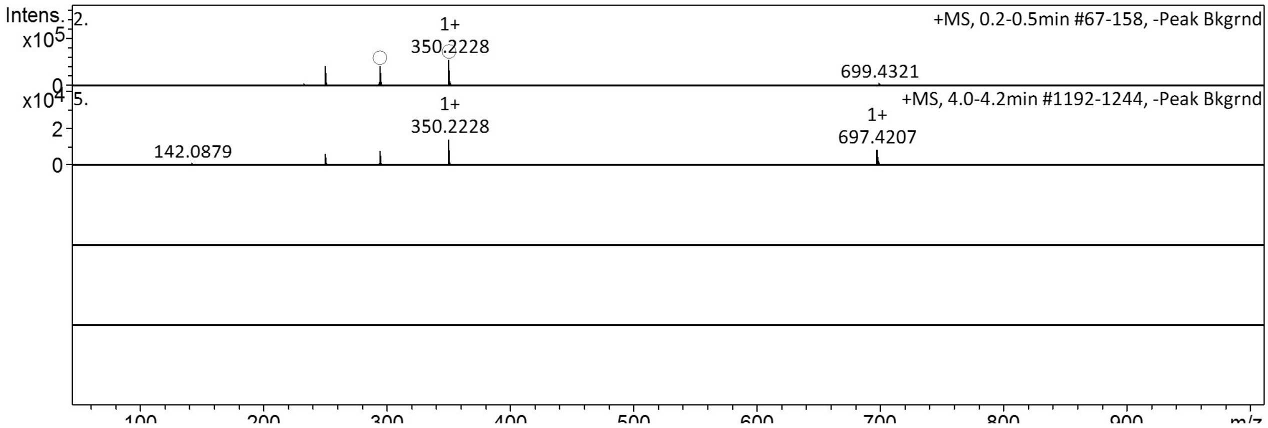

#	RT [min]	Area	Area Frac. %	Chromatogram
1	0.3	1193.772	68.570	UV Chromatogram, 254 nm
2	0.3	532767.188	68.417	BPC 49.0000-1000.0000 +
3	0.6	161.606	9.283	UV Chromatogram, 254 nm
4	4.0	299.212	17.187	UV Chromatogram, 254 nm
5	4.1	245937.859	31.583	BPC 49.0000-1000.0000 +
6	4.5	36.830	2.115	UV Chromatogram, 254 nm
7	4.7	49.528	2.845	UV Chromatogram, 254 nm

PROTON.d MeOD {C:\Bruker\TopSpin3.2} IG500 10

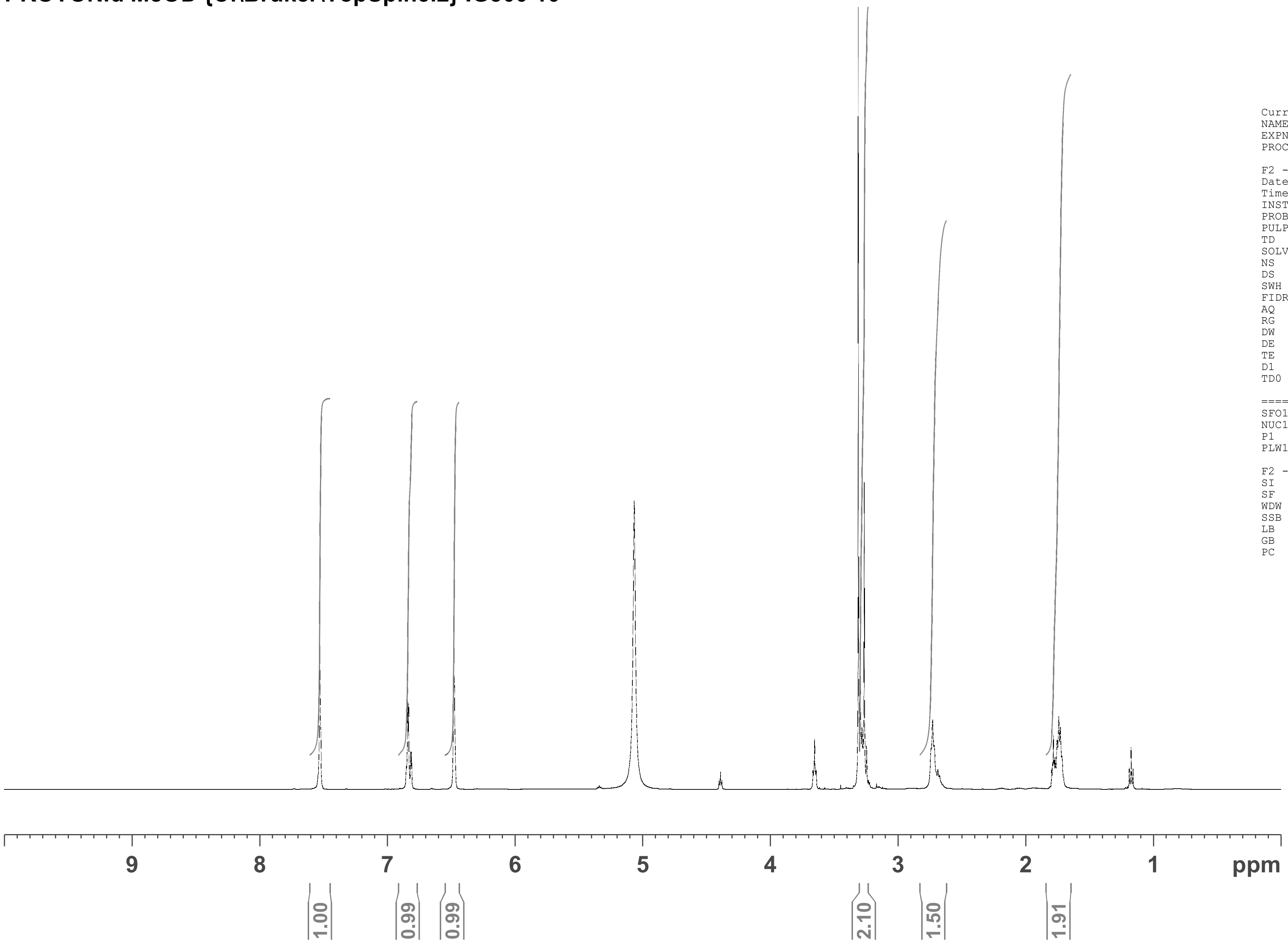


```
Current Data Parameters
NAME      IG-JM-200398-093-P
EXPNO                      1
PROCNO                     1

F2 - Acquisition Parameters
Date_           20170313
Time               15.02
INSTRUM            spect
PROBHD   5 mm QNP 1H/13
PULPROG             zg30
TD                 65536
SOLVENT             MeOD
NS                    16
DS                     2
SWH            10000.000 Hz
FIDRES          0.152588 Hz
AQ             3.2767999 sec
RG                    57
DW                50.000 usec
DE                  6.50 usec
TE                 298.2 K
D1            1.00000000 sec
TD0                    1

======== CHANNEL f1 ========
SFO1         500.1330885 MHz
NUC1                  1H
P1                 10.00 usec
PLW1         25.00000000 W

F2 - Processing parameters
SI                 65536
SF           500.1307703 MHz
WDW                   EM
SSB       0
LB                  0.30 Hz
GB        0
PC                  1.00
```

Compound 42

PROTON.d MeOD {C:\Bruker\TopSpin3.2} IG500 10

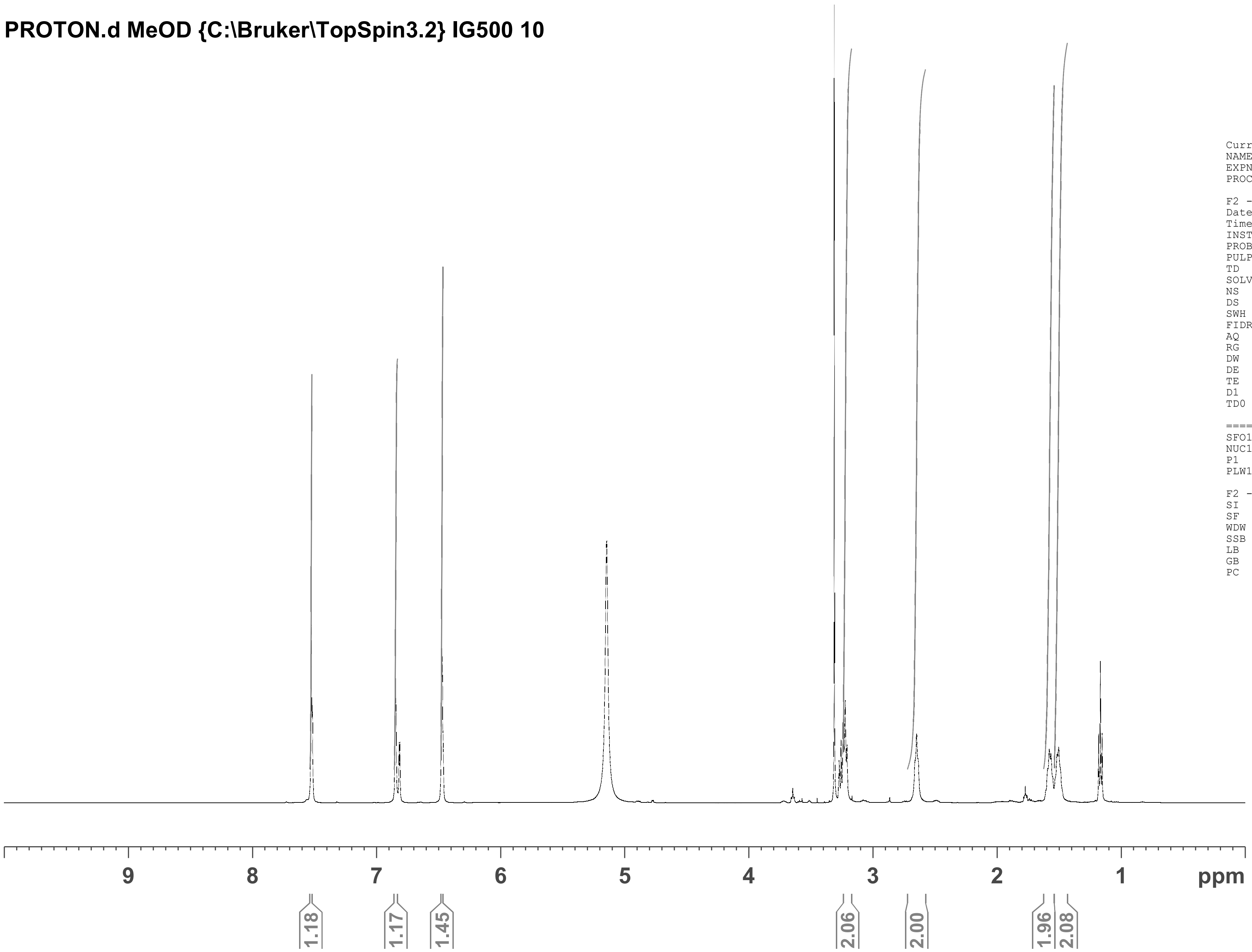


```
Current Data Parameters
NAME       IG-JM-200398-105-P
EXPNO                       1
PROCNO                      1

F2 - Acquisition Parameters
Date_             20170316
Time                 13.38
INSTRUM              spect
PROBHD    5 mm QNP 1H/13
PULPROG               zg30
TD                   65536
SOLVENT               MeOD
NS                      16
DS                       2
SWH              10000.000 Hz
FIDRES            0.152588 Hz
AQ               3.2767999 sec
RG                      57
DW                  50.000 usec
DE                    6.50 usec
TE                   298.2 K
D1              1.00000000 sec
TD0                      1

======== CHANNEL f1 ========
SFO1          500.1330885 MHz
NUC1                    1H
P1                   10.00 usec
PLW1           25.00000000 W

F2 - Processing parameters
SI                   65536
SF            500.1307703 MHz
WDW                     EM
SSB       0
LB                    0.30 Hz
GB        0
PC                    1.00
```

Compound 43

PROTON.d MeOD {C:\Bruker\TopSpin3.2} IG500 11

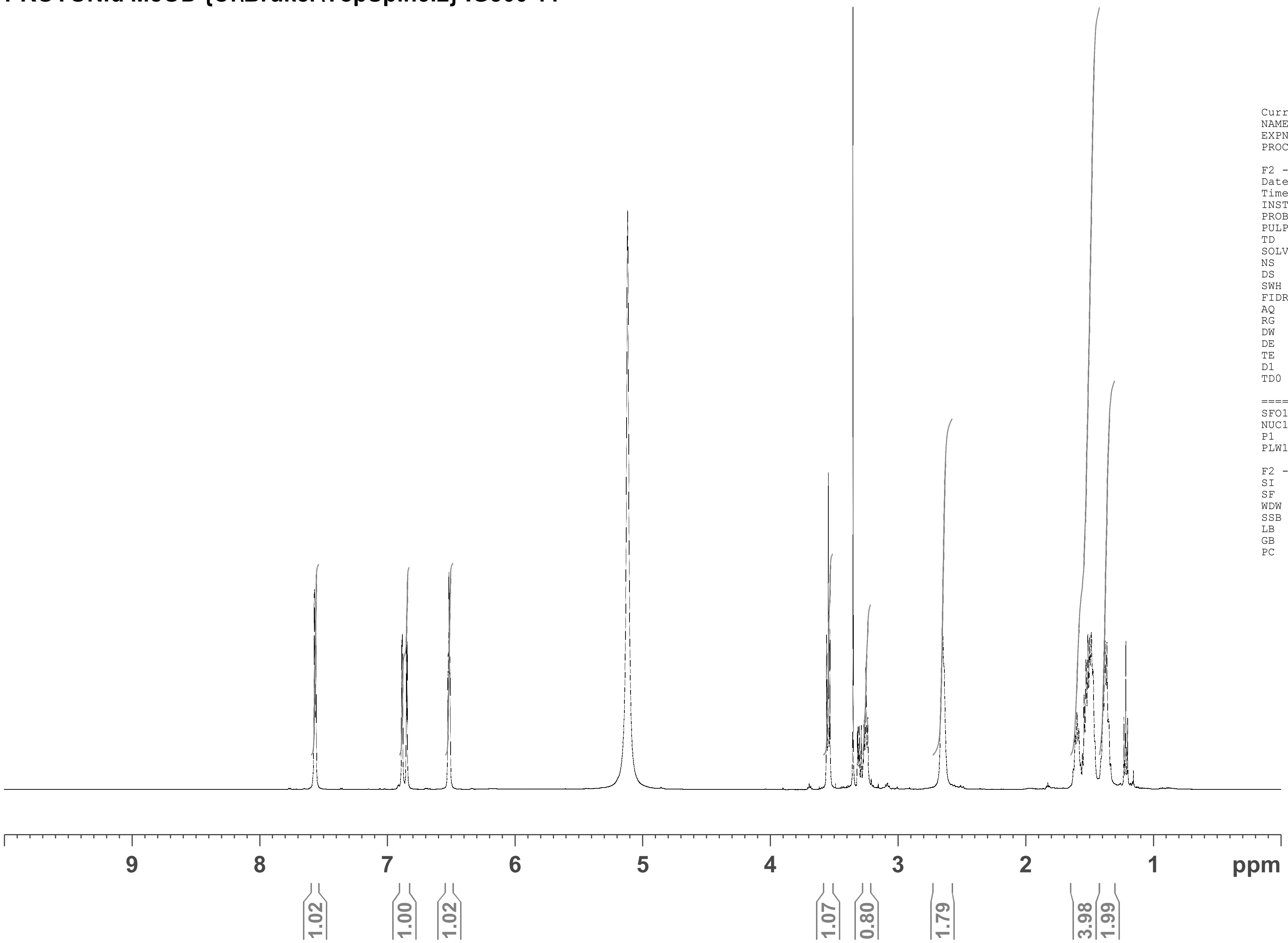


```
Current Data Parameters
NAME        IG-JM-200398-106-P
EXPNO                    1
PROCNO                   1

F2 - Acquisition Parameters
Date_             20170316
Time                 13.55
INSTRUM              spect
PROBHD    5 mm QNP 1H/13
PULPROG               zg30
TD                   65536
SOLVENT               MeOD
NS                      16
DS                       2
SWH           10000.000 Hz
FIDRES         0.152588 Hz
AQ            3.2767999 sec
RG                    50.8
DW               50.000 usec
DE                 6.50 usec
TE                298.2 K
D1           1.00000000 sec
TD0                      1

======== CHANNEL f1 ========
SFO1        500.1330885 MHz
NUC1                    1H
P1                10.00 usec
PLW1        25.00000000 W

F2 - Processing parameters
SI                   65536
SF          500.1307498 MHz
WDW                     EM
SSB      0
LB                   0.30 Hz
GB       0
PC                    1.00
```

Compound 44

PROTON.d MeOD {C:\Bruker\TopSpin3.2} IG500 11

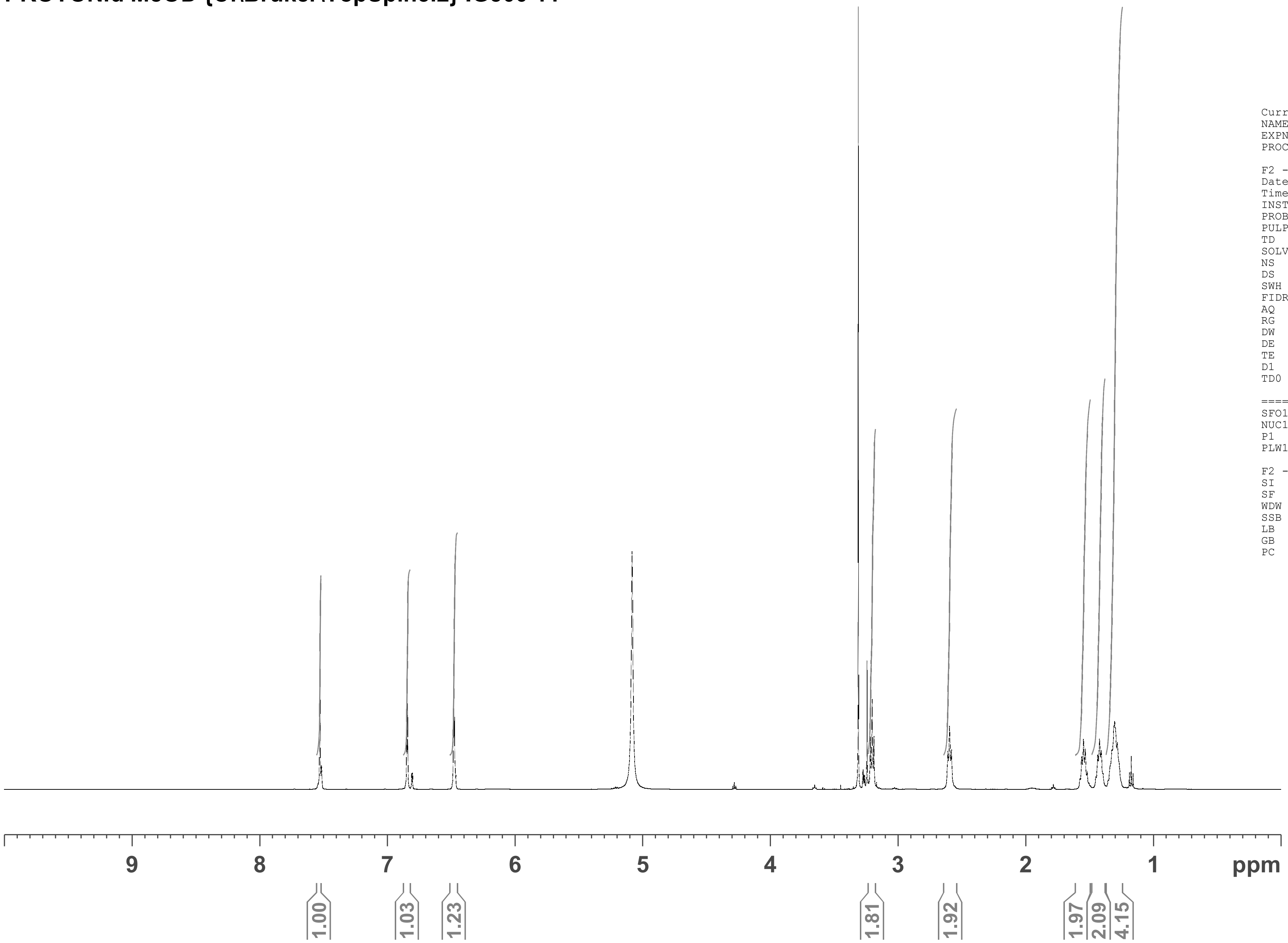


```
Current Data Parameters
NAME     IG-JM-200398-094-P
EXPNO                     1
PROCNO                    1

F2 - Acquisition Parameters
Date_          20170313
Time              15.27
INSTRUM           spect
PROBHD   5 mm QNP 1H/13
PULPROG            zg30
TD                65536
SOLVENT            MeOD
NS                   16
DS                    2
SWH           10000.000 Hz
FIDRES         0.152588 Hz
AQ            3.2767999 sec
RG                 45.2
DW               50.000 usec
DE                 6.50 usec
TE                298.2 K
D1           1.00000000 sec
TD0                   1

======== CHANNEL f1 ========
SFO1        500.1330885 MHz
NUC1                 1H
P1                10.00 usec
PLW1        25.00000000 W

F2 - Processing parameters
SI                65536
SF          500.1307705 MHz
WDW                  EM
SSB      0
LB                 0.30 Hz
GB       0
PC                 1.00
```

Compound 45

Compound 45

Compass OpenAccess LC-MS Identification Report

Sample-ID	JM-200398-094-E	Station	microtof-1
Submitter	James Martin	Supervisor	System Administrator
Analysis Name	JM-200398-094-E_8503_1-B,1_01_13014.d	Acquisition Date	13/03/2017 13:14:58
Sample Description		Method	01-microtof-1-Identify Compounds LCMS Pos 5-95.m

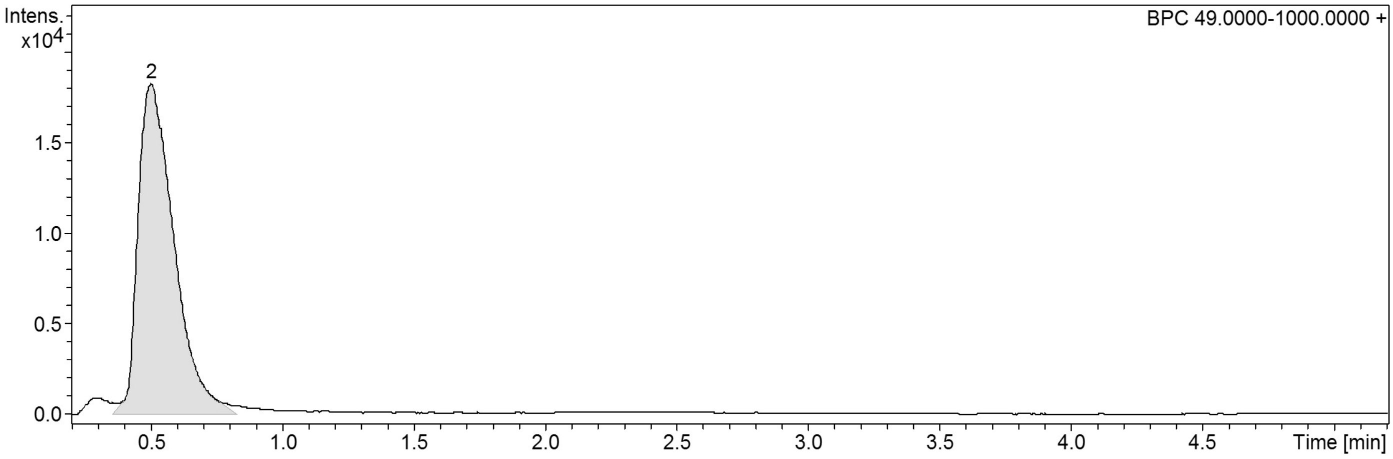

Sample Description

Method 01-microtof-1-Identify Compounds LCMS Pos 5-95.m

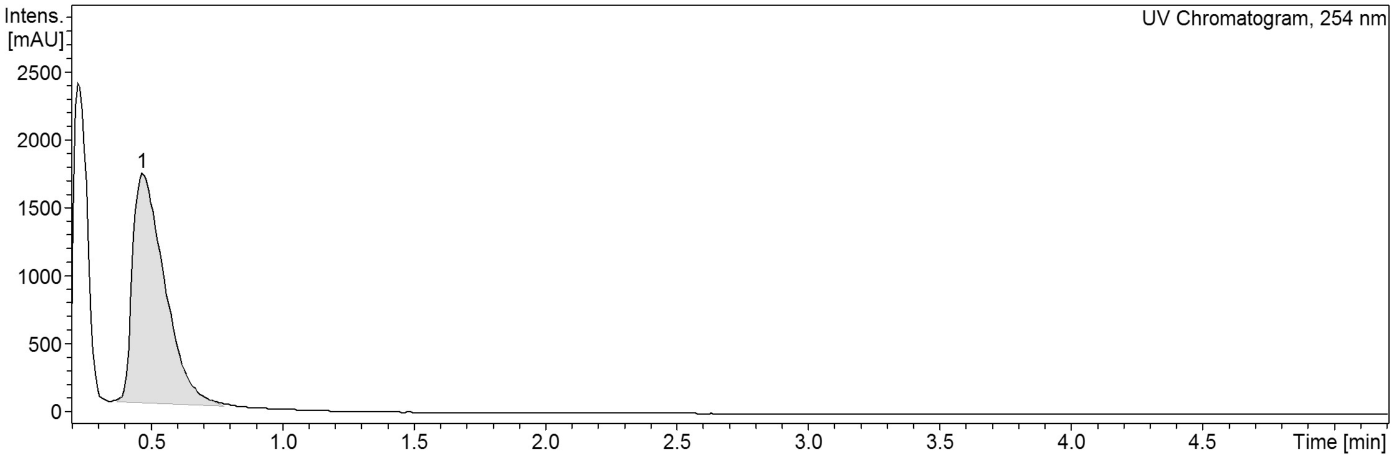

#	RT [min]	Area	Area Frac. %	Chromatogram
1	0.5	14310	100.00	UV Chromatogram, 254 nm
2	0.5	172520	100.00	BPC 49.0000-1000.0000 +

SmartFormula Settings

Tolerance	mSigma Limit	Electron Conf.
2 mDa	20	even

Adduction(s): **Neutral Loss(es):**

Compass OpenAccess LC-MS Identification Report

SmartFormula Results

FormulaMin: C1H1 **FormulaMax:** Na

#	meas. m/z	theo. m/z	|err| [ppm]	mSigma	Formula	Purity(UVC)[%]	Purity(BPC)[%]
	No Results!						

Note: mSigma values <30 indicate high probability of correct molecular formula

Cmpd 1, 0.5 min

Undefined context.
Component not placed in a spectrum iterator.

Cmpd 2, 0.5 min

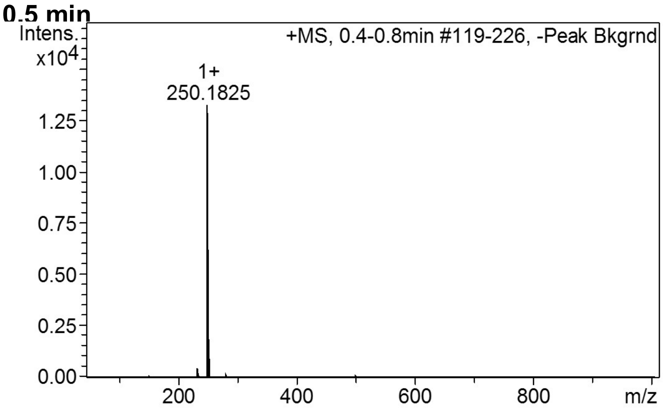

PROTON.d MeOD {C:\Bruker\TopSpin3.2} IG500 18

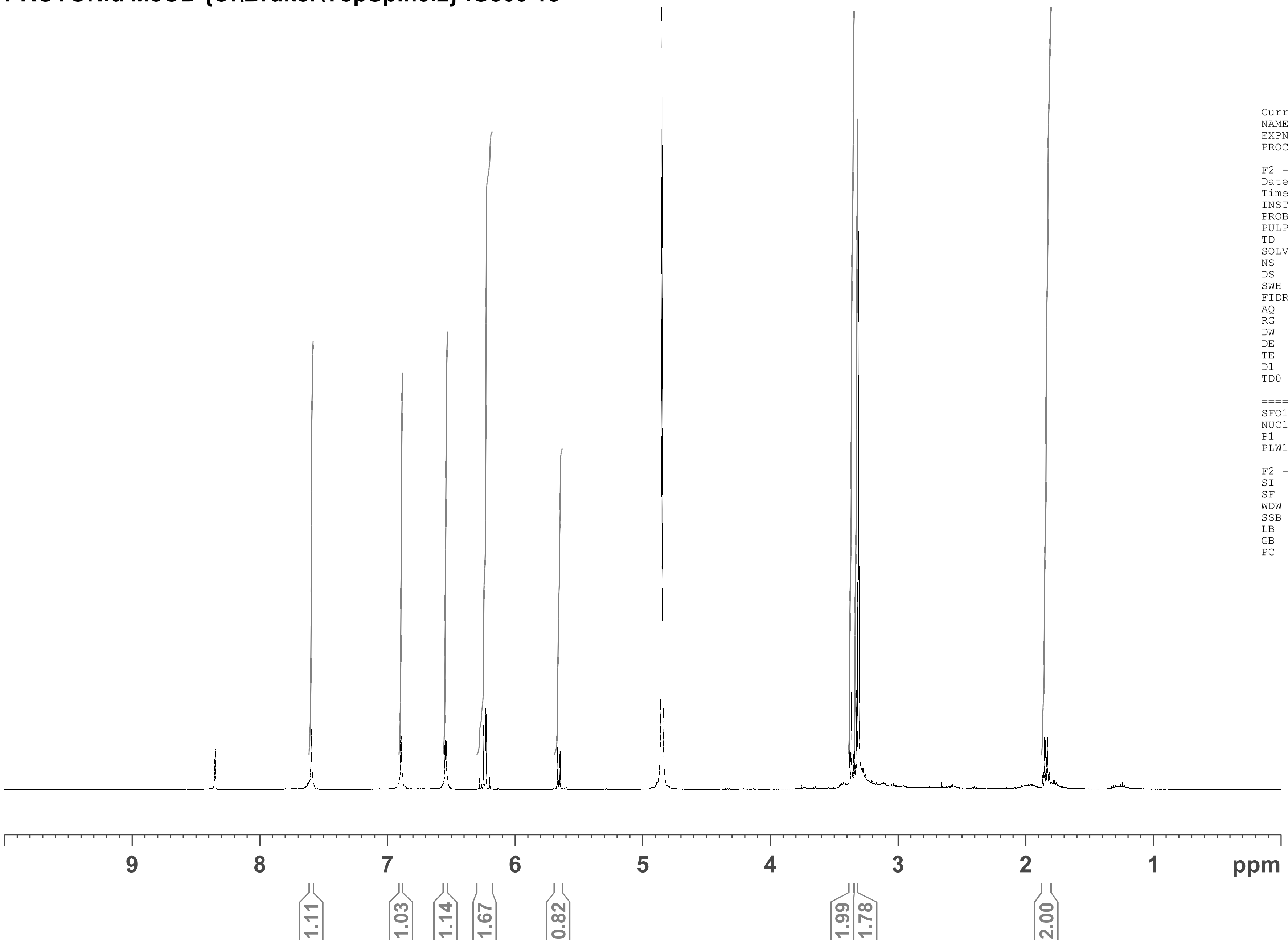


```
Current Data Parameters
NAME      IG-JM-200398-115-P
EXPNO                     1
PROCNO                    1

F2 - Acquisition Parameters
Date_              20170322
Time                   1.03
INSTRUM               spect
PROBHD    5 mm QNP 1H/13
PULPROG                zg30
TD                    65536
SOLVENT                MeOD
NS                       32
DS                        2
SWH              10000.000 Hz
FIDRES            0.152588 Hz
AQ               3.2767999 sec
RG                      456
DW                   50.000 usec
DE                     6.50 usec
TE                    298.2 K
D1               1.00000000 sec
TD0                       1

======== CHANNEL f1 ========
SFO1          500.1330885 MHz
NUC1                     1H
P1                    10.00 usec
PLW1            25.00000000 W

F2 - Processing parameters
SI                    65536
SF            500.1299892 MHz
WDW                      EM
SSB      0
LB                     0.30 Hz
GB       0
PC                     1.00
```

Compound 46

C13CPD.d MeOD {C:\Bruker\TopSpin3.2} IG500 18

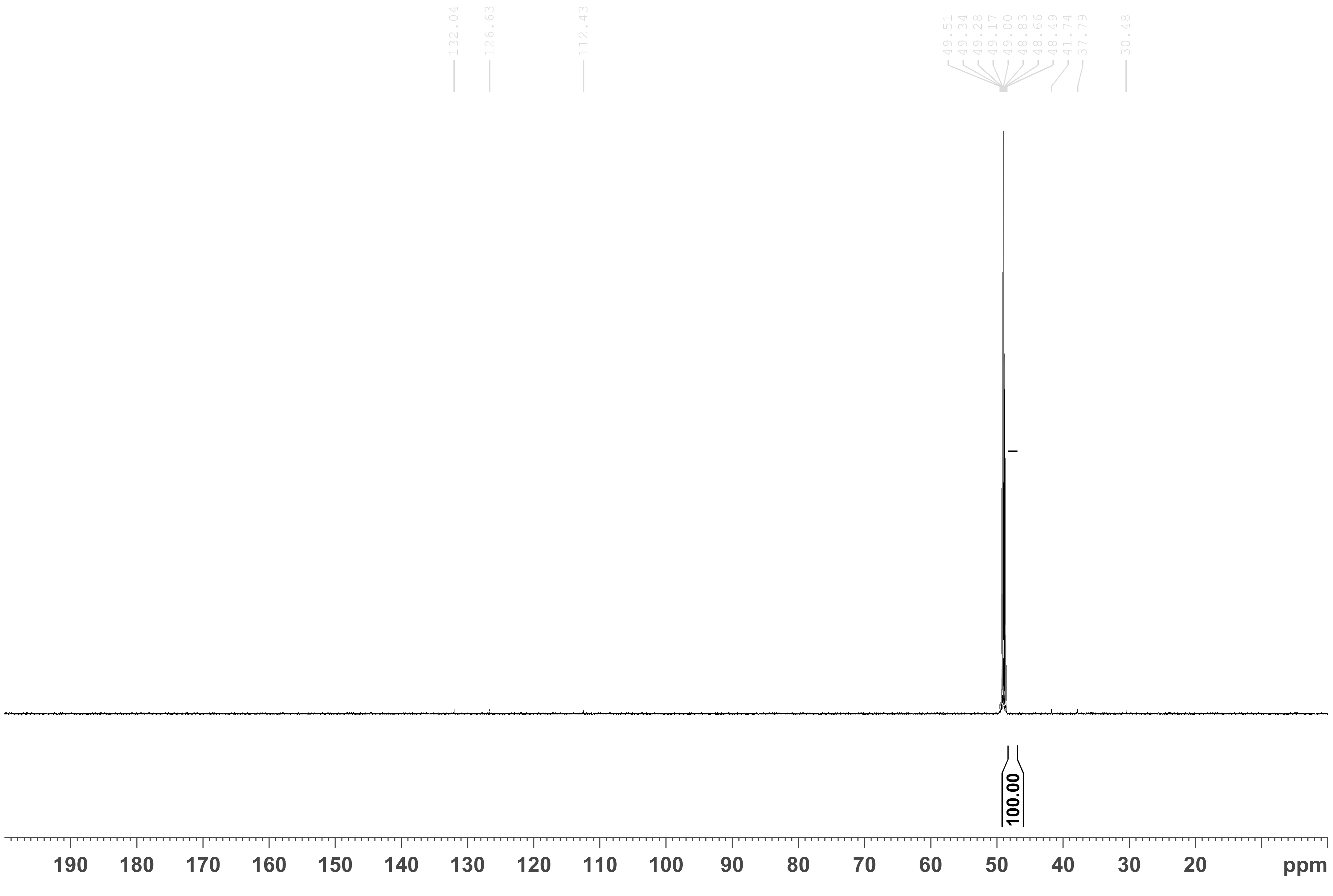

Compound 46

Compound 46

Mass Spectrum SmartFormula Report

**Analysis Info**		Acquisition Date	23/03/2017 17:23:31	
Analysis Name	D:\Data\james\JM-200398-115-001_11982_RE5_01_13368.d			
Method	1-microtof-2 Identify Compounds LCMS Pos 5-95.m	Operator	Dundee University	
Sample Name	JM-200398-115-001_11982	Instrument / Ser#	micrOTOF II	8213750.10435
Comment				

Acquisition Parameter

Source Type	ESI	Ion Polarity	Positive	Set Nebulizer	0.8 Bar
Focus	Not active			Set Dry Heater	210 °C
Scan Begin	75 m/z	Set Capillary	4500 V	Set Dry Gas	8.0 l/min
Scan End	1200 m/z	Set End Plate Offset	-500 V	Set Divert Valve	Waste

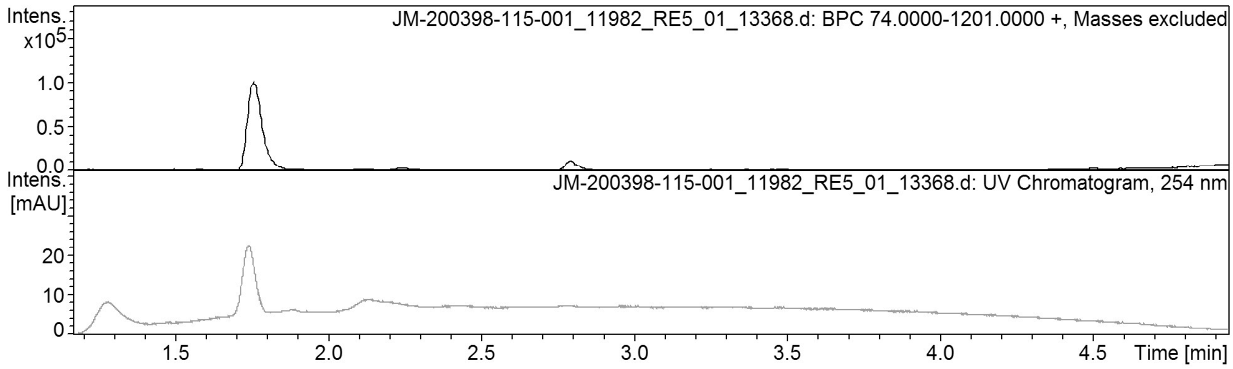

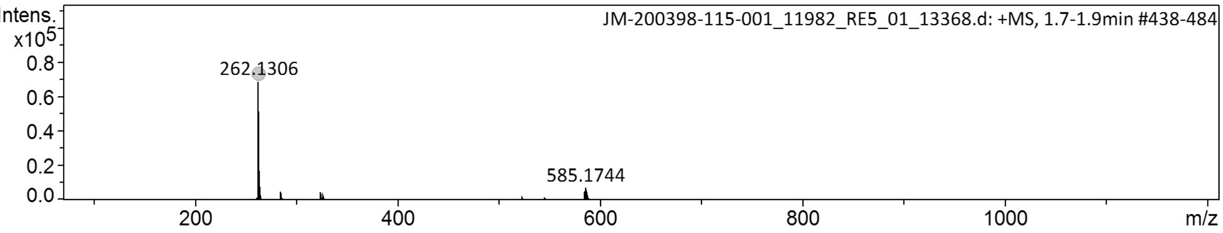

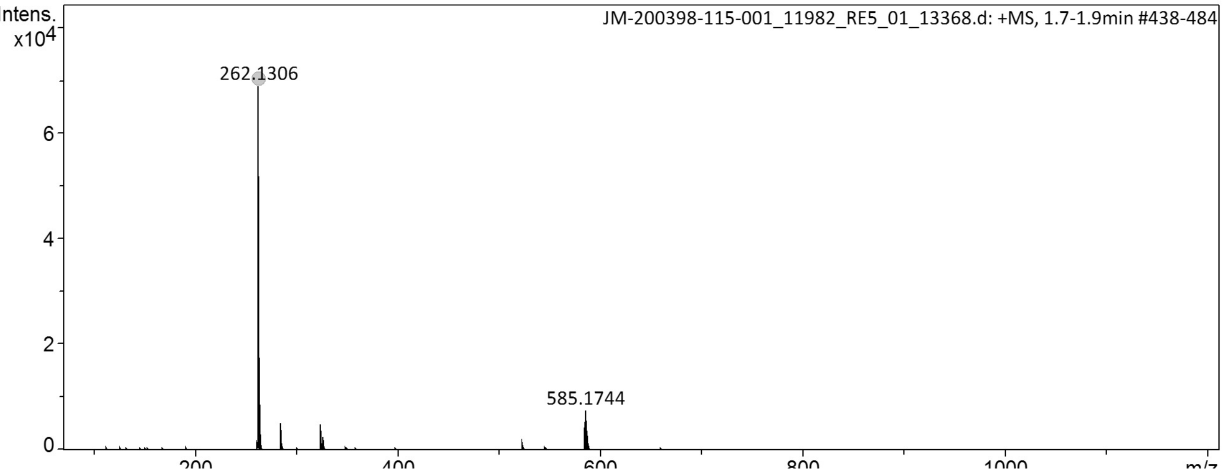

Meas. m/z	#	Ion Formula	m/z	err [mDa]	err [ppm]	Mean err [ppm]	rdb	N-Rule	e¯ Conf	mSigma
262.130574	1	C12H16N5O2	262.129851	-0.7	-2.8	-4.8	8.0	ok	even	8.0

PROTON.d MeOD {C:\Bruker\TopSpin3.2} IG500 10

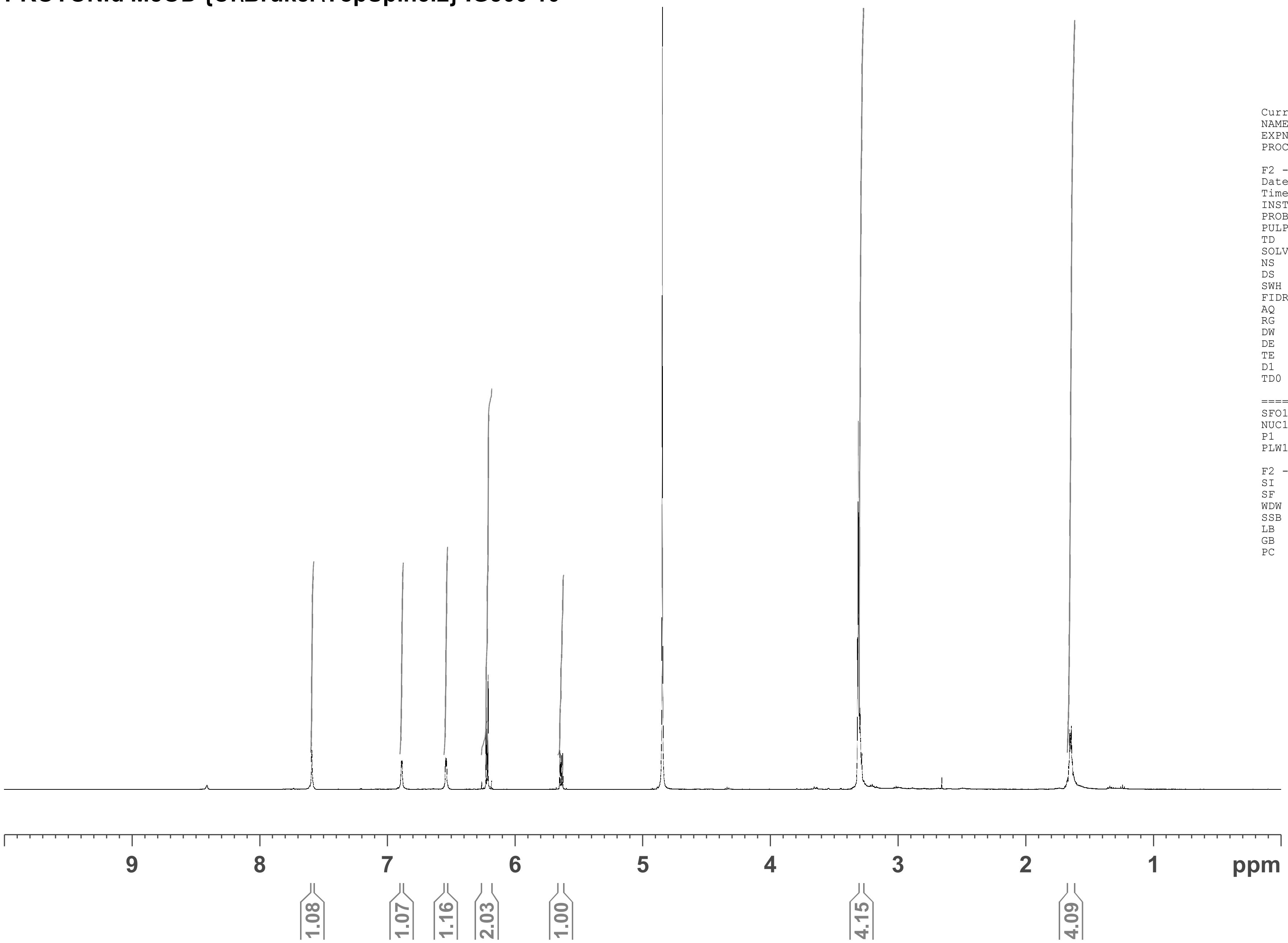


```
Current Data Parameters
NAME      IG-JM-200398-107-P
EXPNO                     1
PROCNO                    1

F2 - Acquisition Parameters
Date_              20170321
Time                  17.21
INSTRUM               spect
PROBHD    5 mm QNP 1H/13
PULPROG                zg30
TD                    65536
SOLVENT                MeOD
NS                       32
DS                        2
SWH             10000.000 Hz
FIDRES           0.152588 Hz
AQ              3.2767999 sec
RG                      456
DW                   50.000 usec
DE                     6.50 usec
TE                    298.2 K
D1             1.00000000 sec
TD0                       1

======== CHANNEL f1 ========
SFO1          500.1330885 MHz
NUC1                     1H
P1                    10.00 usec
PLW1          25.00000000 W

F2 - Processing parameters
SI                    65536
SF            500.1307509 MHz
WDW                      EM
SSB       0
LB                     0.30 Hz
GB        0
PC                     1.00
```

Compound 47

C13CPD.d MeOD {C:\Bruker\TopSpin3.2} IG500 10

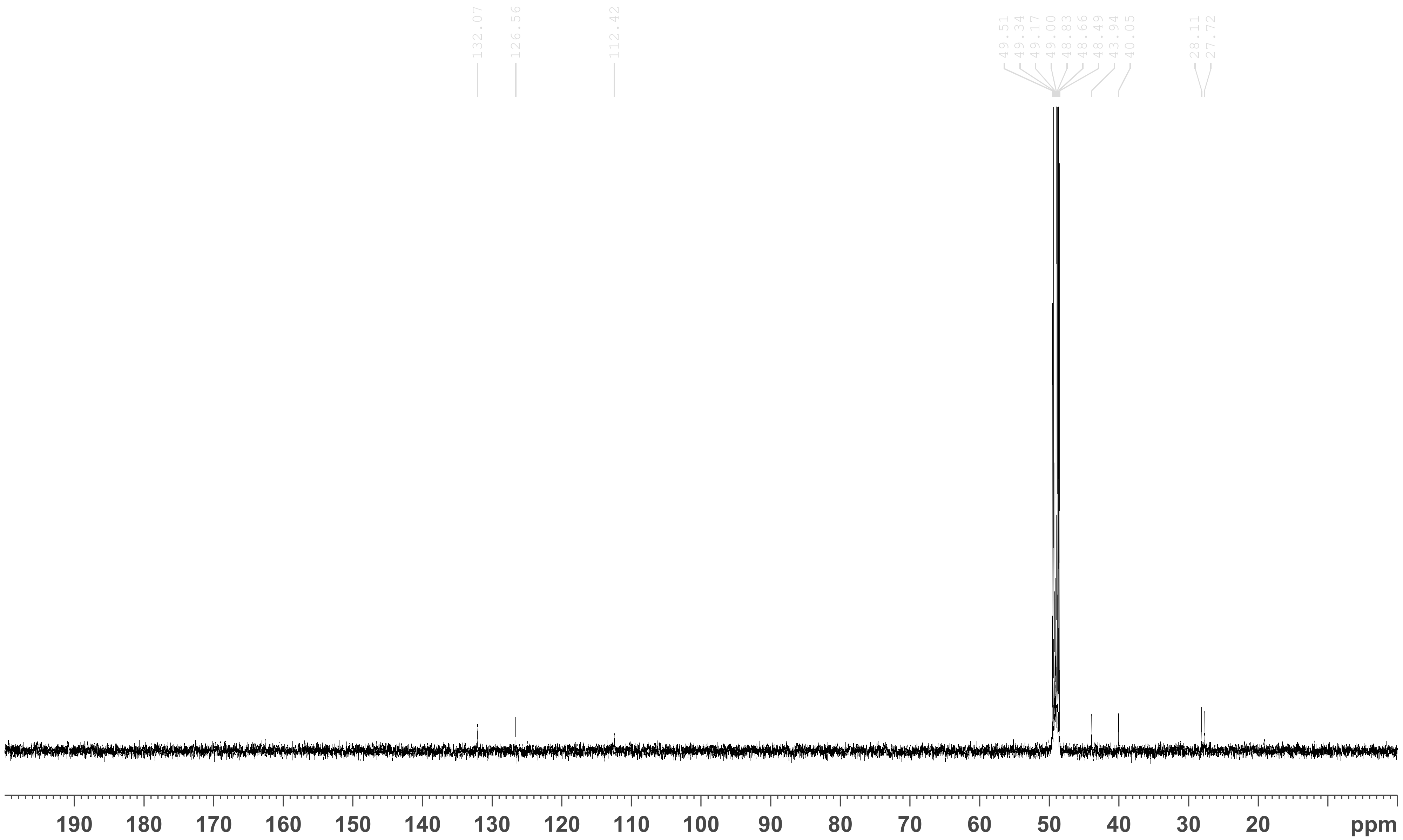

Compound 47

Compound 47

Compound Verification Report (Compass OpenAccess/QC)

Sample-ID	JM-200398-107-001	Station	Microtof-2
Submitter	James Martin	Supervisor	System Administrator
Analysis Name	JM-200398-107-001_12308_RA3_01_50.d	Acquisition Date	4/27/2017 4:24:16 PM
Sample Description			

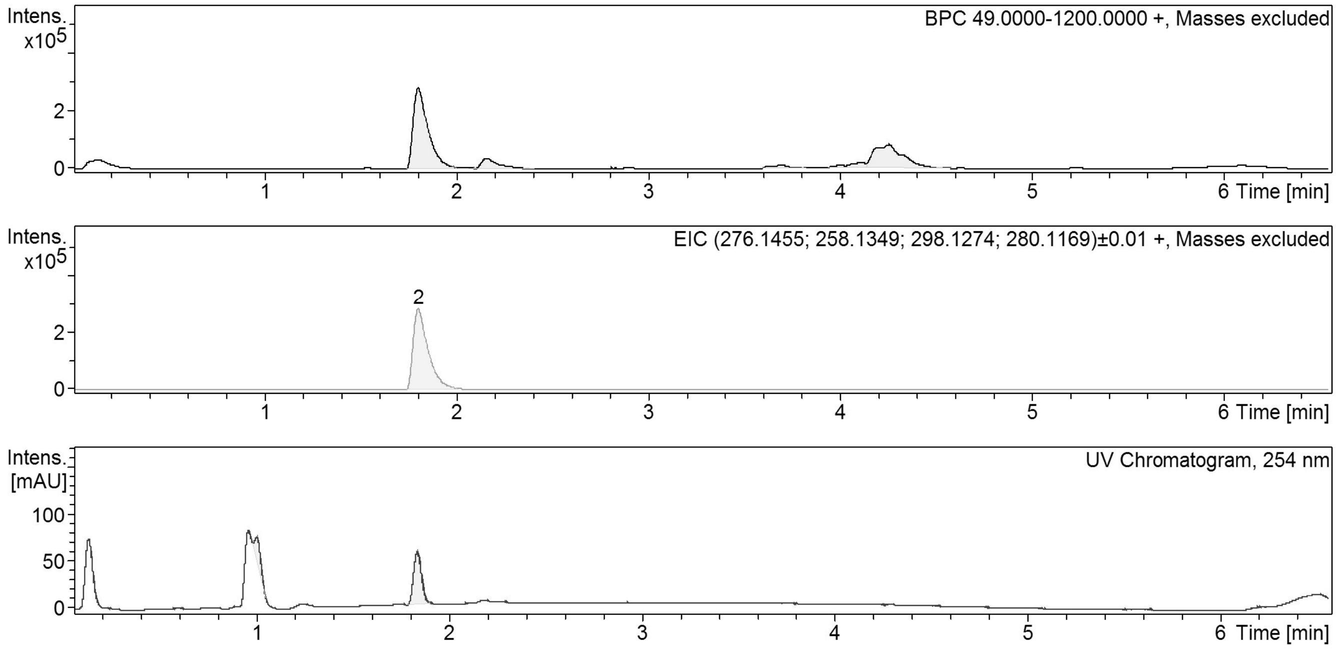

SmartFormula Settings

Tolerance	mSigma Limit	Electron Conf.
10 ppm	60	even

Adduction(s): H, Na **Neutral Loss(es):** H2O

Compound Verification Results

Expected Formula: C13H17N5O2

#	meas. m/z	theo. m/z	|err| [ppm]	mSigma	Formula	Modification	Purity(UVC)[%]	Purity(BPC)[%]
2	276.1467	276.1455	4.4	7	C13 H18 N5 O2	(M+H)+	74.6	54.0

Note: mSigma values <20 indicate high probability of correct molecular formula

Compound Verification Report (Compass OpenAccess/QC)

Sample-ID	JM-200398-108-001	Station	Microtof-2
Submitter	James Martin	Supervisor	System Administrator
Analysis Name	JM-200398-108-001_12311_RA4_01_51.d	Acquisition Date	4/27/2017 4:32:43 PM
Sample Description			

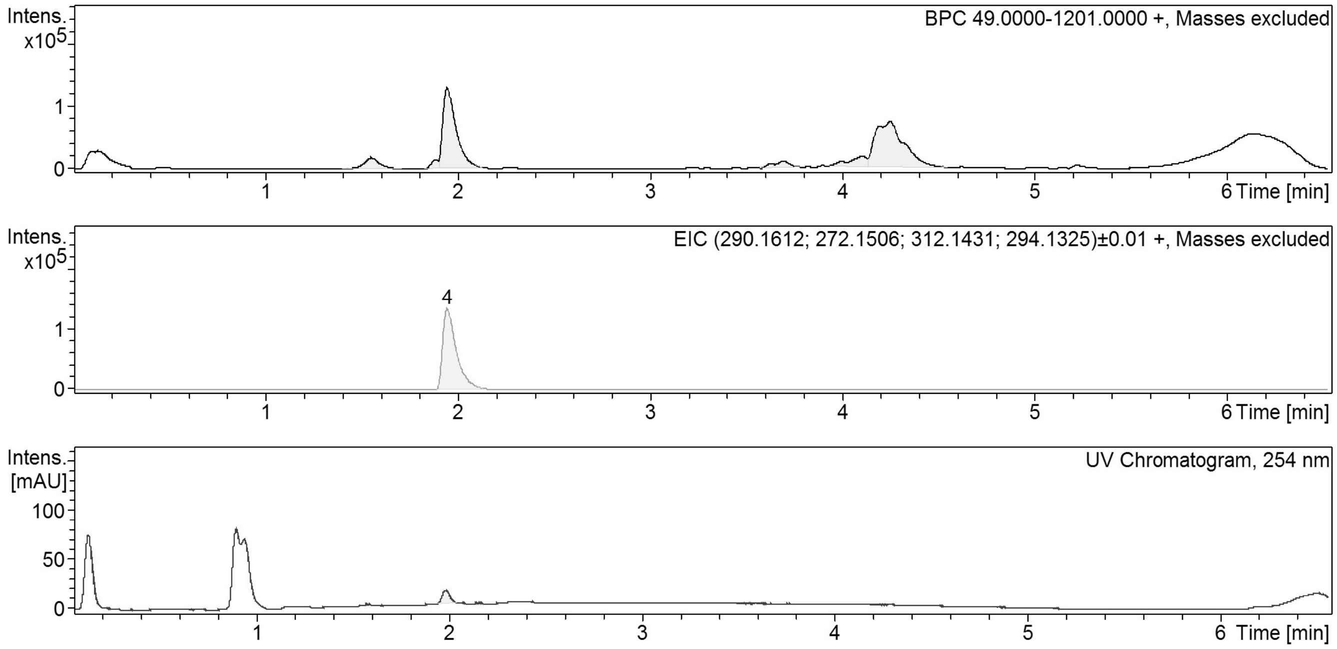

SmartFormula Settings

Tolerance	mSigma Limit	Electron Conf.
10 ppm	60	even

Adduction(s): H, Na **Neutral Loss(es):** H2O

Compound Verification Results

Expected Formula: C14H19N5O2

#	meas. m/z	theo. m/z	|err| [ppm]	mSigma	Formula	Modification	Purity(UVC)[%]	Purity(BPC)[%]
4	290.1616	290.1612	1.5	30	C14 H20 N5 O2	(M+H)+	100.0	36.5

Note: mSigma values <20 indicate high probability of correct molecular formula

PROTON.d MeOD {C:\Bruker\TopSpin3.2} IG500 34

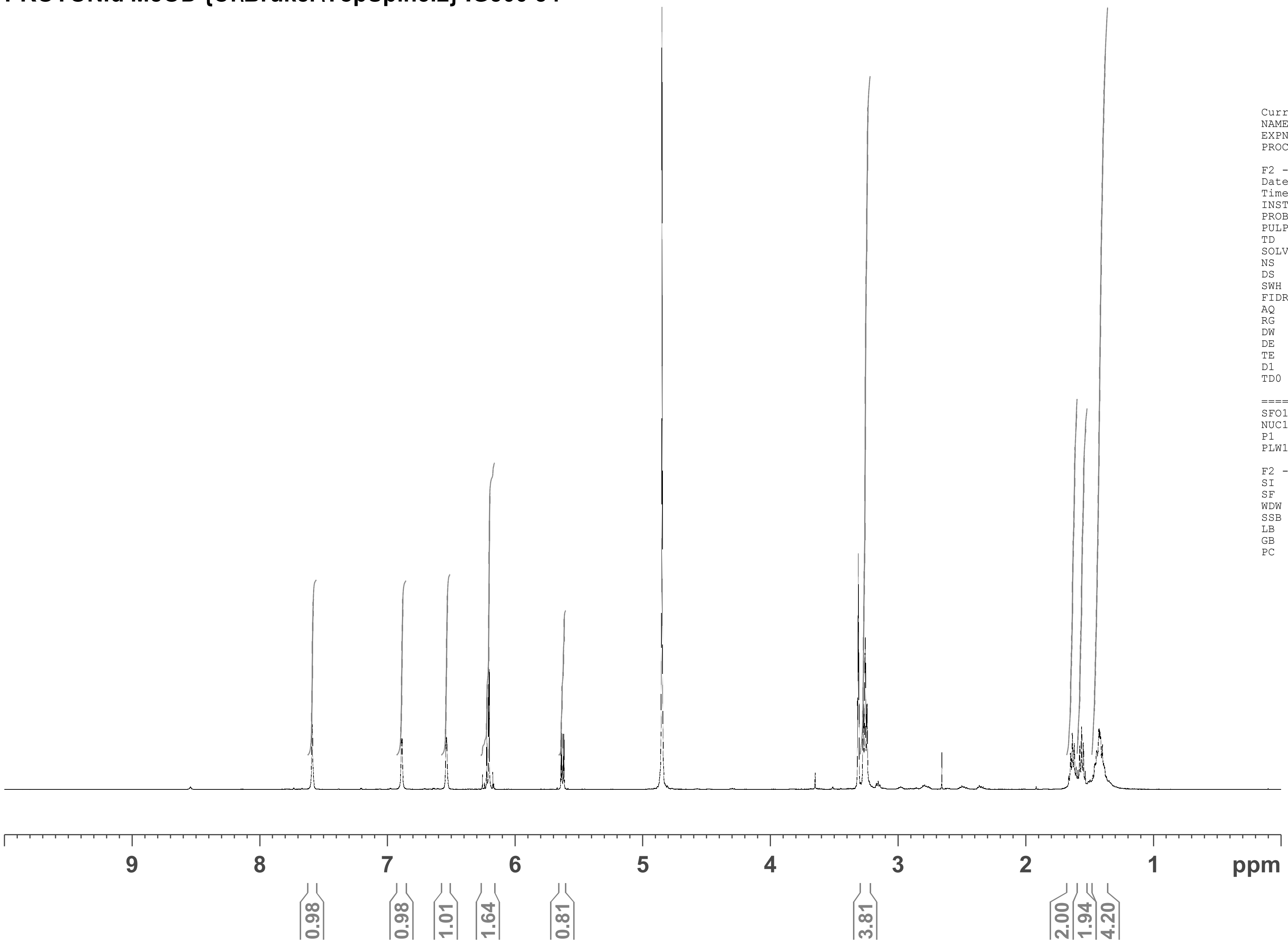


```
Current Data Parameters
NAME     IG-JM-200398-098-001
EXPNO                   1
PROCNO                  1

F2 - Acquisition Parameters
Date_          20170320
Time              10.09
INSTRUM           spect
PROBHD   5 mm QNP 1H/13
PULPROG            zg30
TD                65536
SOLVENT            MeOD
NS                   16
DS                    2
SWH           10000.000 Hz
FIDRES         0.152588 Hz
AQ            3.2767999 sec
RG                  362
DW               50.000 usec
DE                 6.50 usec
TE                298.2 K
D1           1.00000000 sec
TD0                   1

======== CHANNEL f1 ========
SFO1        500.1330885 MHz
NUC1                 1H
P1                10.00 usec
PLW1        25.00000000 W

F2 - Processing parameters
SI                65536
SF          500.1299900 MHz
WDW                  EM
SSB      0
LB                 0.30 Hz
GB       0
PC                 1.00
```

Compound 49

C13CPD.d MeOD {C:\Bruker\TopSpin3.2} IG500 34

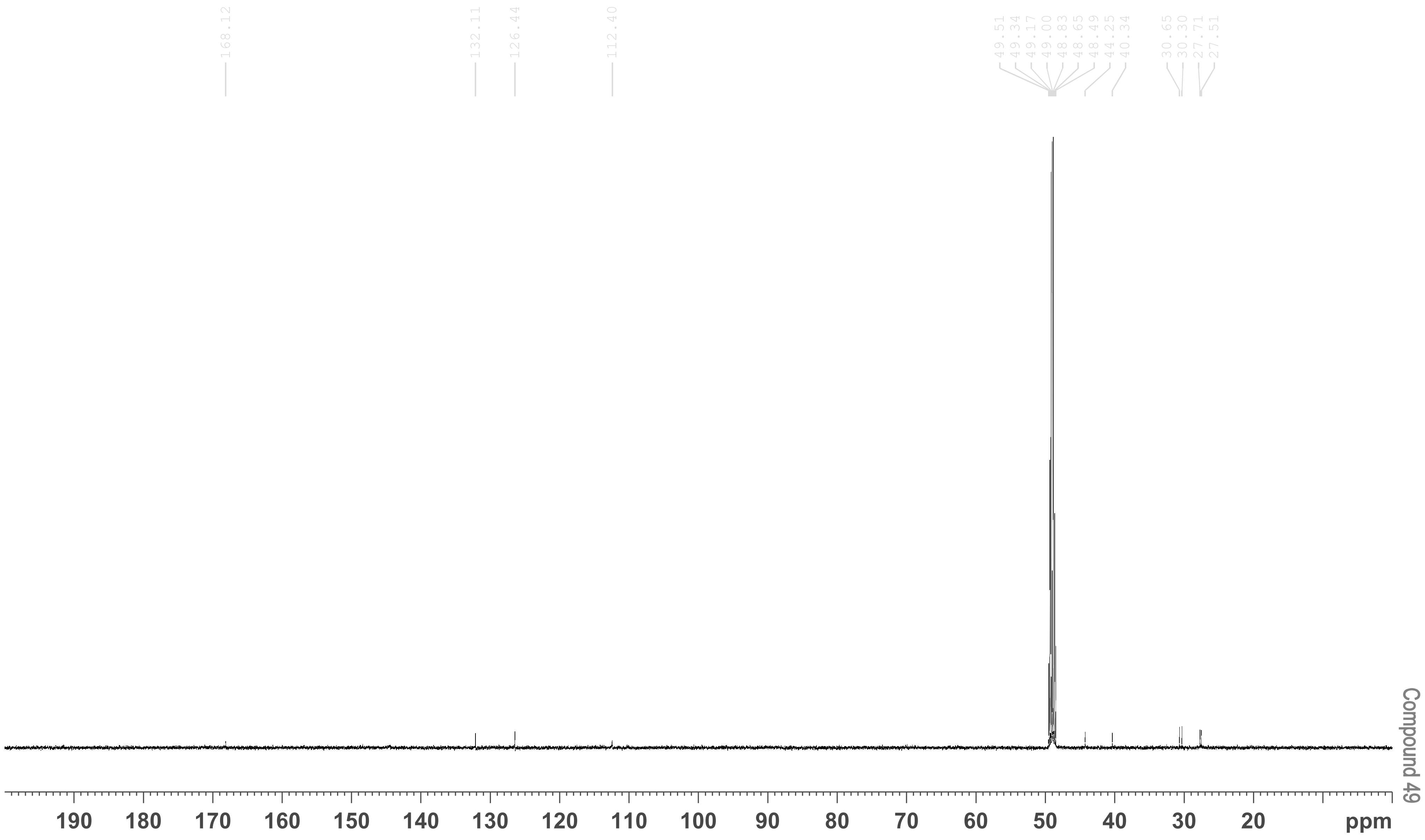

Compound Verification Report (Compass OpenAccess/QC)

Sample-ID	JM-200398-098-001	Station	Microtof-2
Submitter	James Martin	Supervisor	System Administrator
Analysis Name	JM-200398-098-001_11960_RB7_01_13343.d	Acquisition Date	23/03/2017 13:49:16
Sample Description		Method	2-microtof-2 verify compounds lcms pos 5-95.m

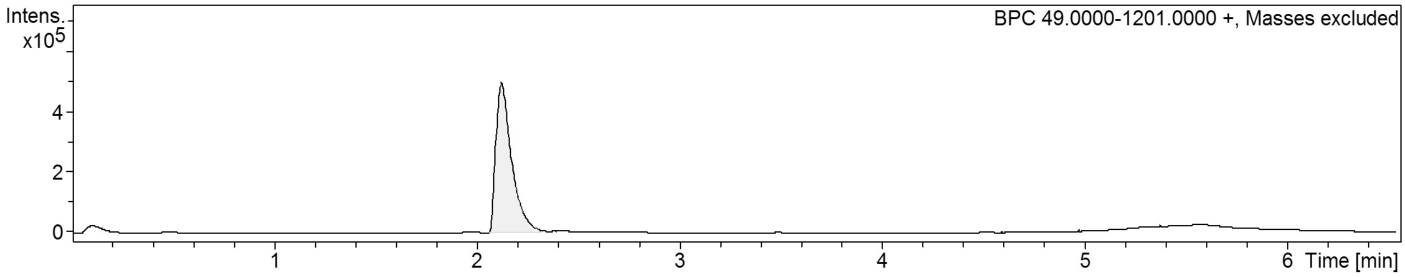

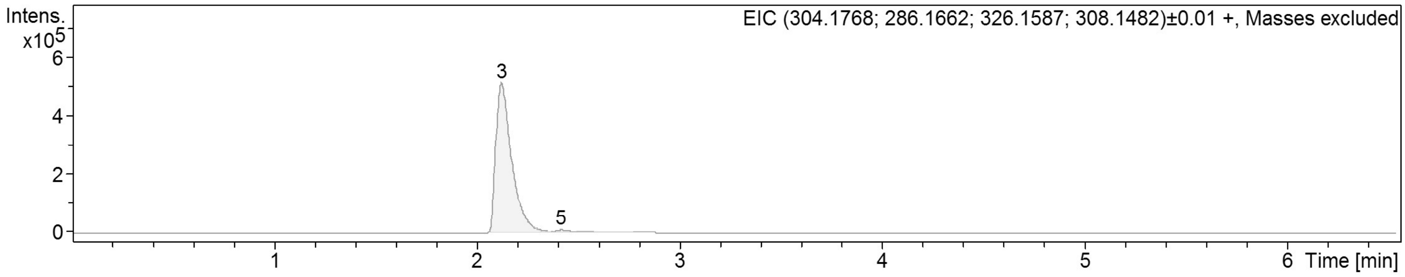

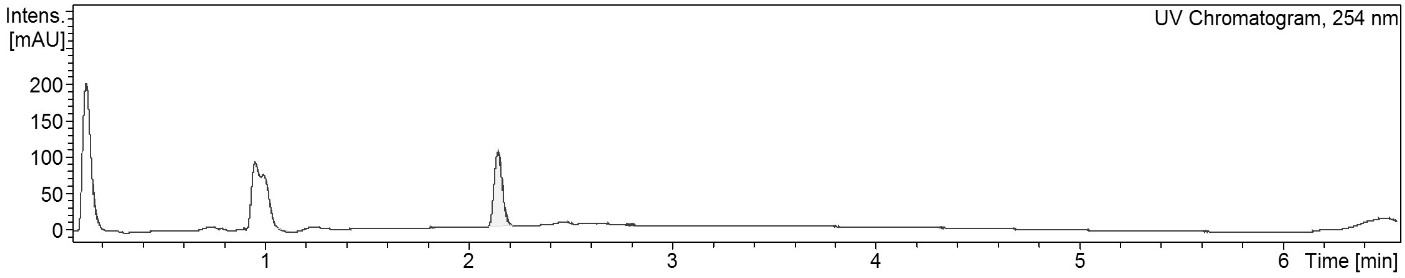

SmartFormula Settings

Tolerance	mSigma Limit	Electron Conf.
10 ppm	60	even

Adduction(s): H, Na **Neutral Loss(es):** H2O

Compound Verification Results

Expected Formula: C15H21N5O2

#	meas. m/z	theo. m/z	|err| [ppm]	mSigma	Formula	Modification	Purity(UVC)[%]	Purity(BPC)[%]
3	304.1778	304.1768	3.4	30	C15 H22 N5 O2	(M+H)+	97.9	100.0
5	304.1765	304.1768	1.0	55	C15 H22 N5 O2	(M+H)+	0.0	0.0

Note: mSigma values <20 indicate high probability of correct molecular formula

Compound Verification Report (Compass OpenAccess/QC)

Cmpd 3, 2.1 min

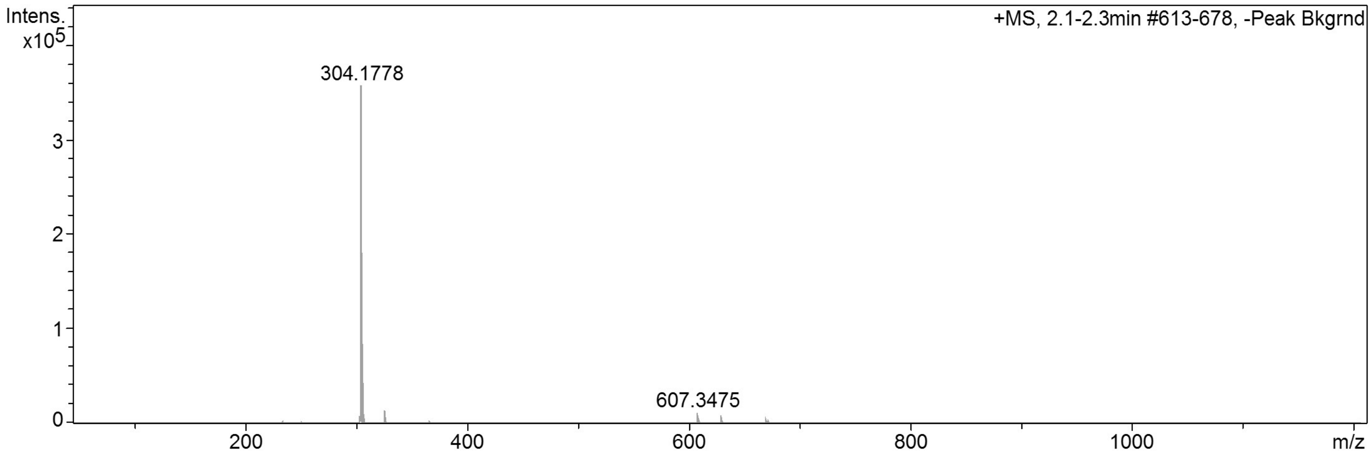

Cmpd 5, 2.4 min

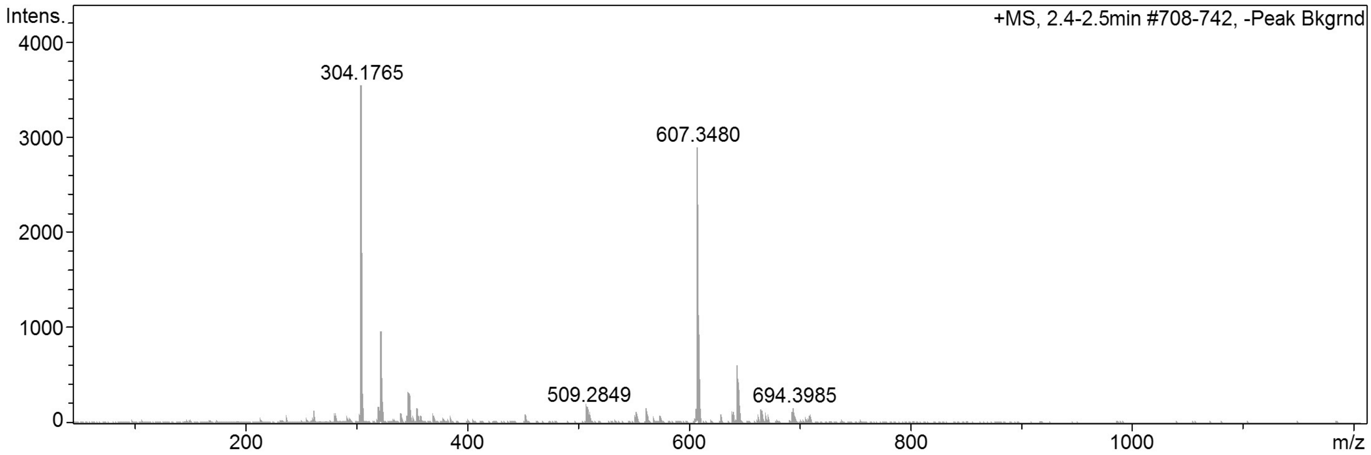

PROTON.d MeOD {C:\Bruker\TopSpin3.2} IG500 2

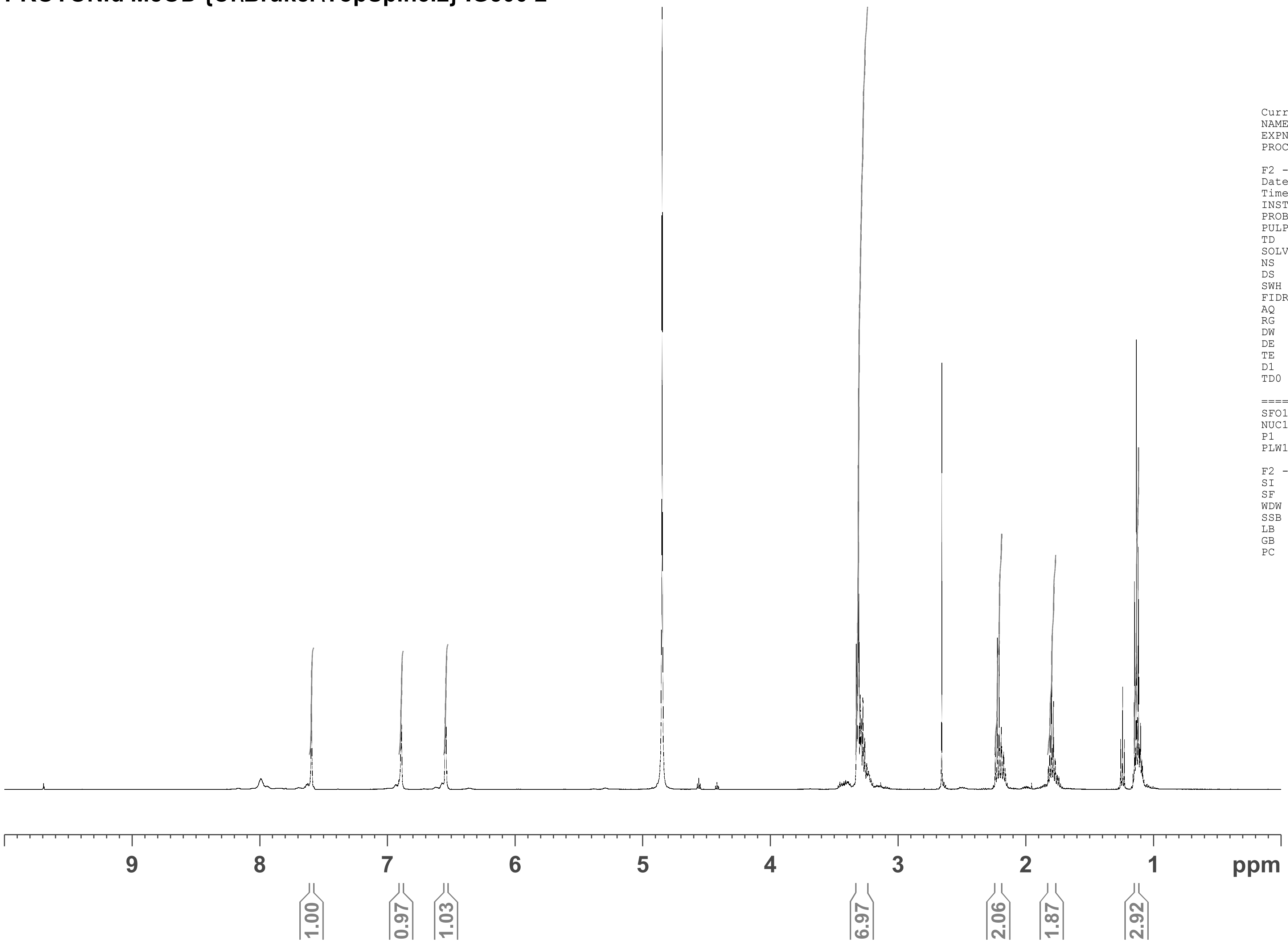


```
Current Data Parameters
NAME     IG-JM-200398-099-P
EXPNO                     1
PROCNO                    1

F2 - Acquisition Parameters
Date_          20170320
Time              16.43
INSTRUM           spect
PROBHD   5 mm QNP 1H/13
PULPROG            zg30
TD                65536
SOLVENT            MeOD
NS                   32
DS                    2
SWH           10000.000 Hz
FIDRES         0.152588 Hz
AQ            3.2767999 sec
RG                  322
DW               50.000 usec
DE                 6.50 usec
TE                298.2 K
D1           1.00000000 sec
TD0                   1

======== CHANNEL f1 ========
SFO1        500.1330885 MHz
NUC1                 1H
P1                10.00 usec
PLW1        25.00000000 W

F2 - Processing parameters
SI                65536
SF          500.1307509 MHz
WDW                  EM
SSB      0
LB                 0.30 Hz
GB       0
PC                 1.00
```

Compound 50

C13CPD.d MeOD {C:\Bruker\TopSpin3.2} IG500 2

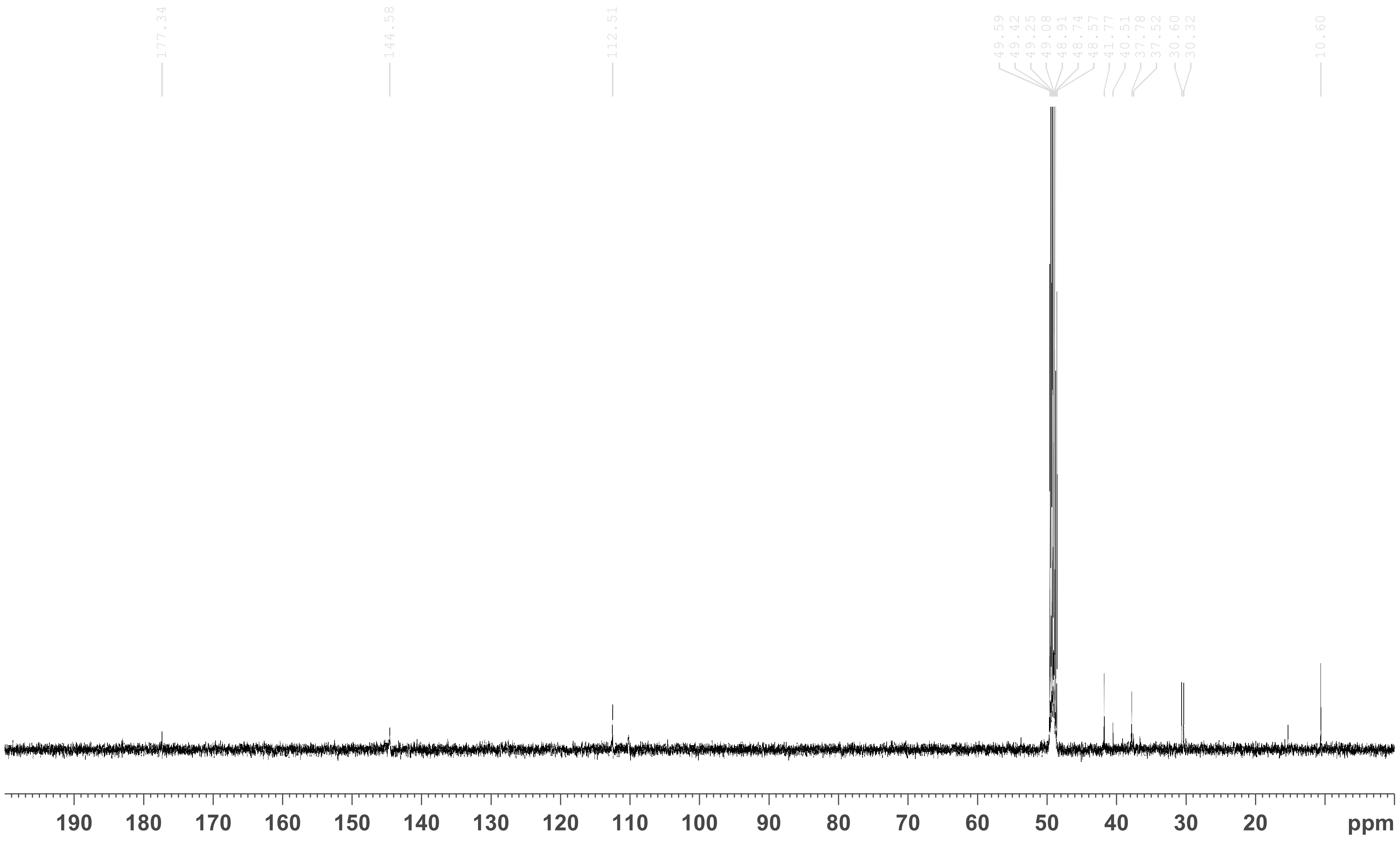

Compound 50

Compound Verification Report (Compass OpenAccess/QC)

Sample-ID	JM-200398-099-001	Station	Microtof-2
Submitter	James Martin	Supervisor	System Administrator
Analysis Name	JM-200398-099-001_11961_RB8_01_13344.d	Acquisition Date	23/03/2017 13:57:48
Sample Description		Method	2-microtof-2 verify compounds lcms pos 5-95.m

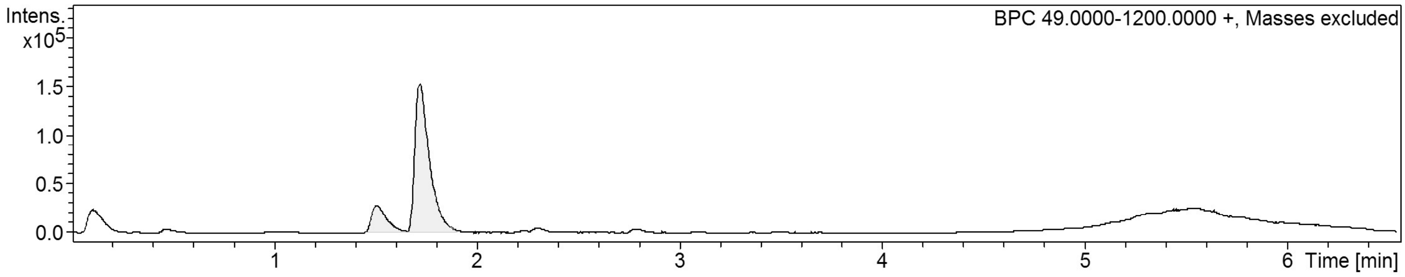

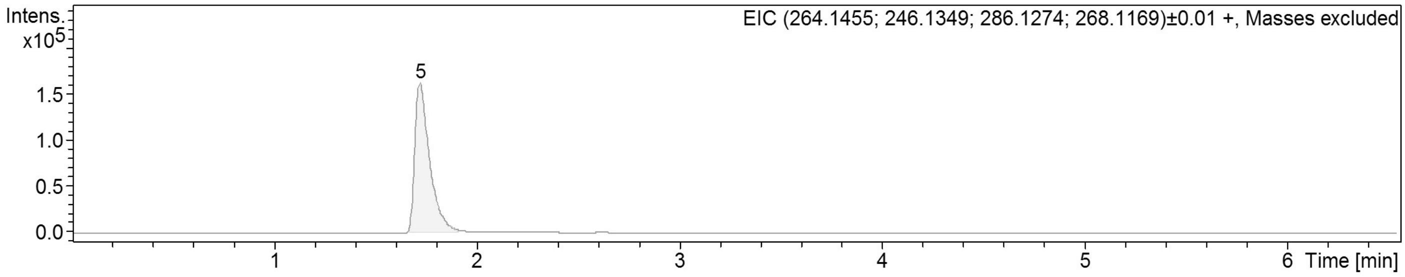

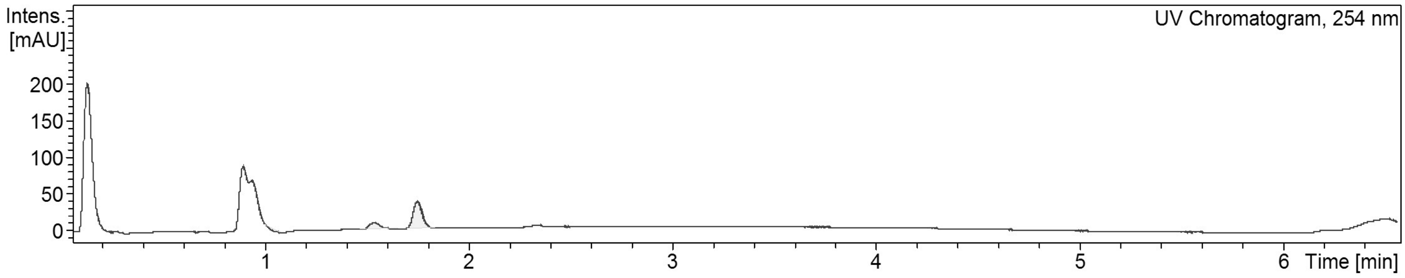

SmartFormula Settings

Tolerance	mSigma Limit	Electron Conf.
10 ppm	60	even

Adduction(s): H, Na **Neutral Loss(es):** H2O

Compound Verification Results

Expected Formula: C12H17N5O2

#	meas. m/z	theo. m/z	|err| [ppm]	mSigma	Formula	Modification	Purity(UVC)[%]	Purity(BPC)[%]
5	264.1452	264.1455	1.0	20	C12 H18 N5 O2	(M+H)+	71.5	83.9

Note: mSigma values <20 indicate high probability of correct molecular formula

Compound Verification Report (Compass OpenAccess/QC)

Cmpd 5, 1.7 min

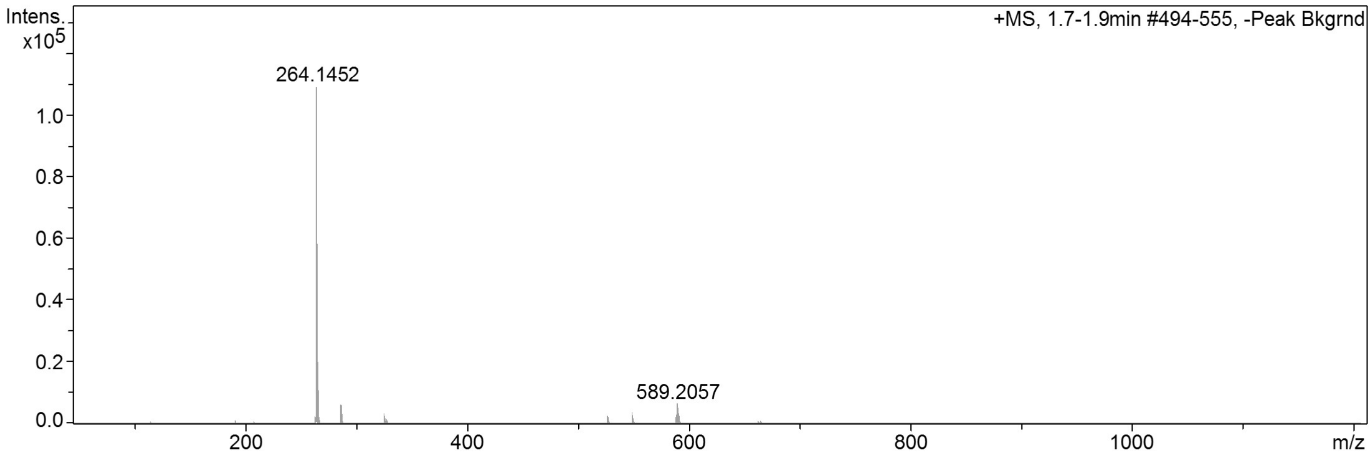

Analysis Name D:\Data\james\JM-200398-099-001_11961_RB8_01_13344.d

PROTON.d MeOD {C:\Bruker\TopSpin3.2} IG500 12

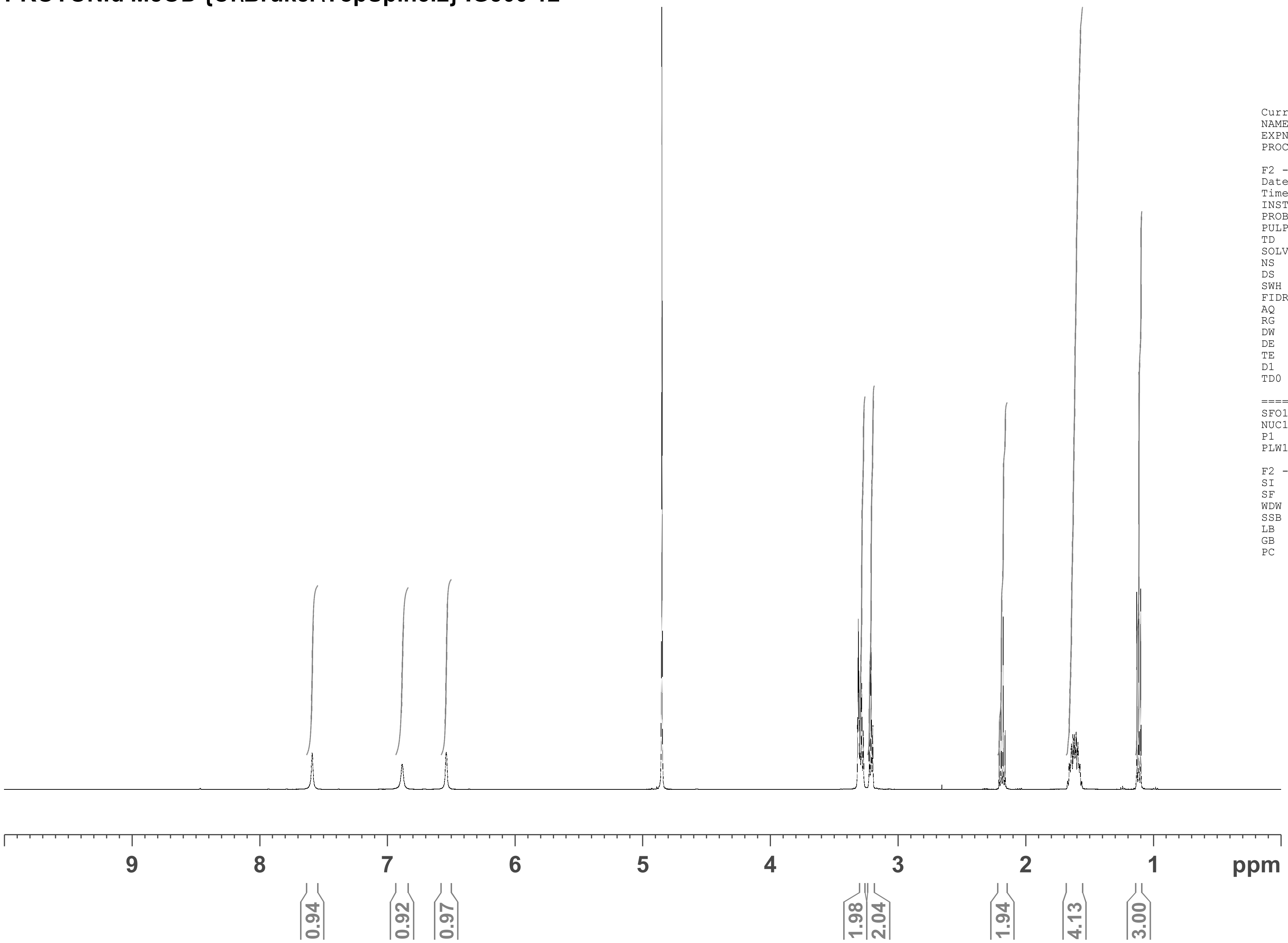


```
Current Data Parameters
NAME     IG-JM-200398-109-P
EXPNO                    1
PROCNO                   1

F2 - Acquisition Parameters
Date_          20170321
Time              19.16
INSTRUM           spect
PROBHD   5 mm QNP 1H/13
PULPROG            zg30
TD                65536
SOLVENT            MeOD
NS                   32
DS                    2
SWH           10000.000 Hz
FIDRES         0.152588 Hz
AQ            3.2767999 sec
RG                  362
DW               50.000 usec
DE                 6.50 usec
TE                298.2 K
D1           1.00000000 sec
TD0                   1

======== CHANNEL f1 ========
SFO1        500.1330885 MHz
NUC1                 1H
P1                10.00 usec
PLW1        25.00000000 W

F2 - Processing parameters
SI                65536
SF          500.1299908 MHz
WDW                  EM
SSB      0
LB                 0.30 Hz
GB       0
PC                 1.00
```

Compound 51

C13CPD.d MeOD {C:\Bruker\TopSpin3.2} IG500 12

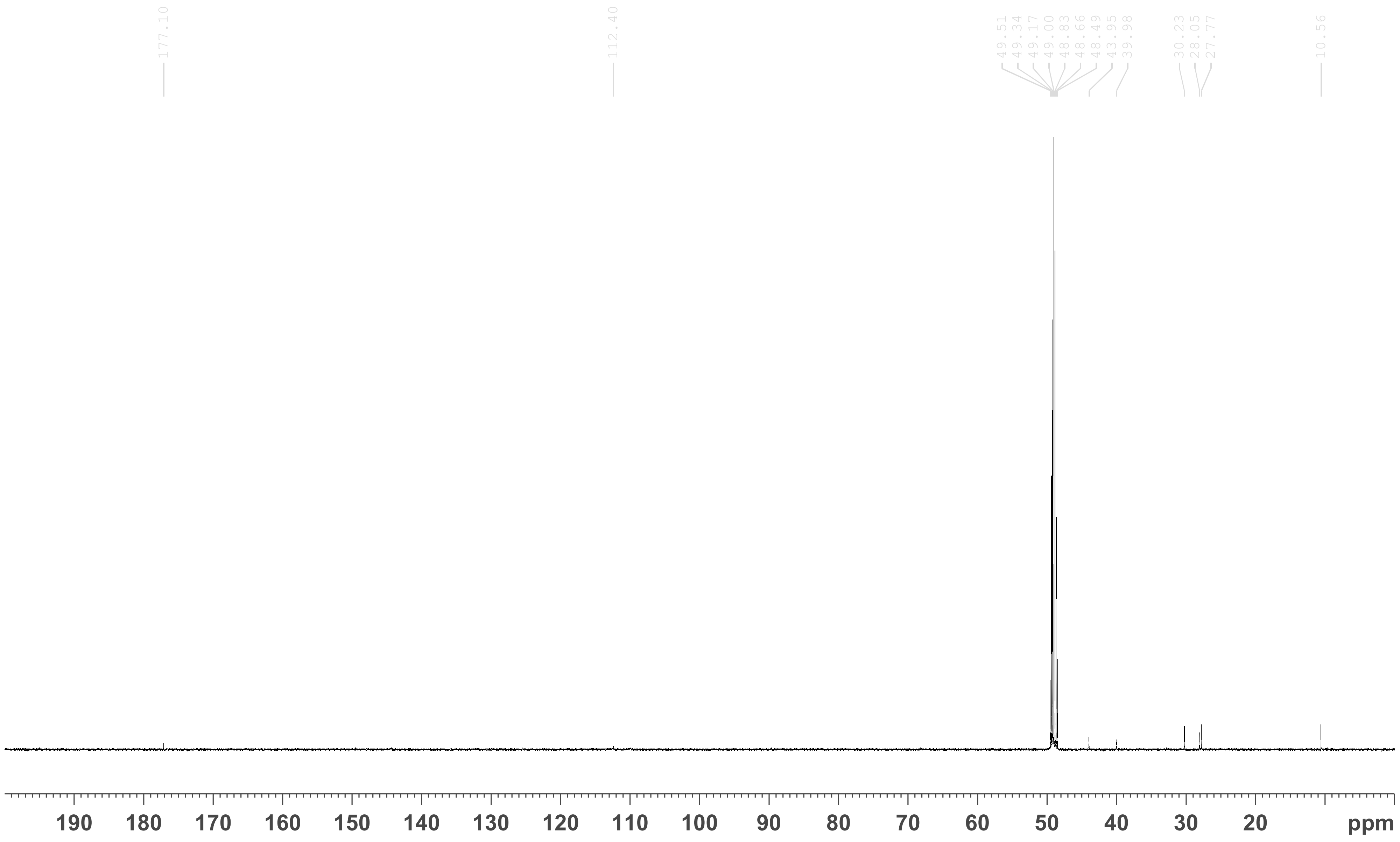

Compound 51

Compound 51

Compound Verification Report (Compass OpenAccess/QC)

Sample-ID	JM-200398-109-001	Station	Microtof-2
Submitter	James Martin	Supervisor	System Administrator
Analysis Name	JM-200398-109-001_12310_RA5_01_52.d	Acquisition Date	4/27/2017 4:41:11 PM
Sample Description			

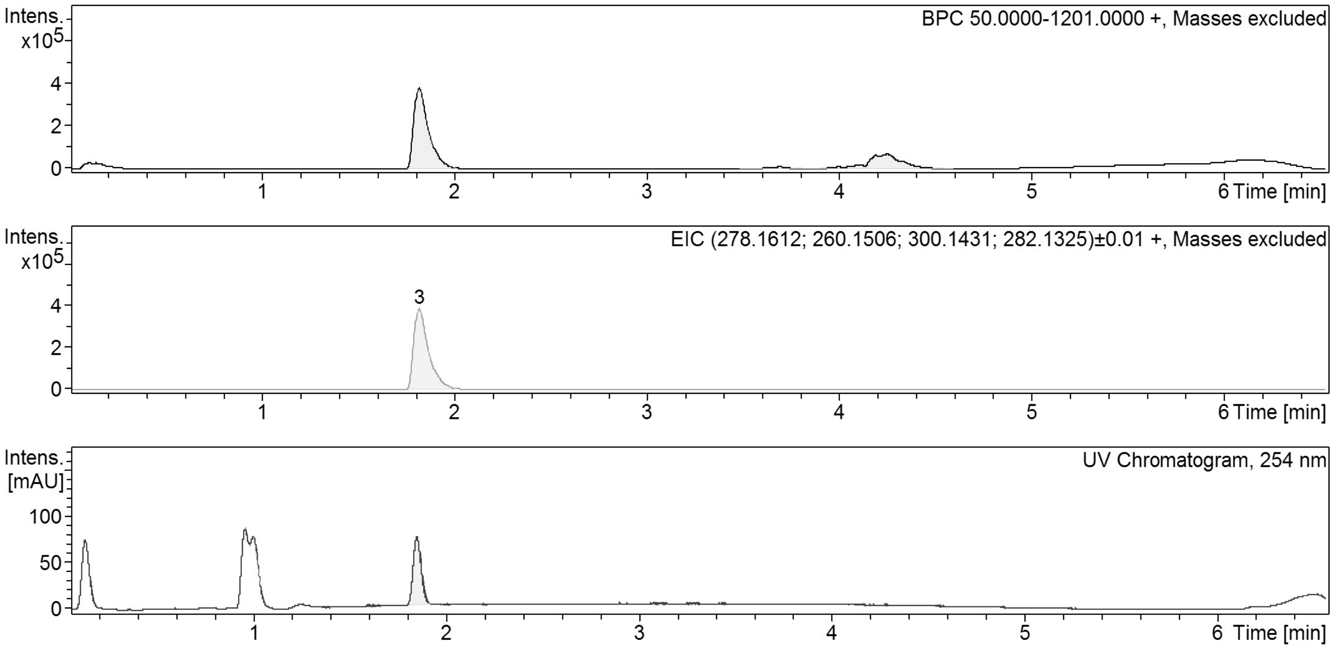

SmartFormula Settings

Tolerance	mSigma Limit	Electron Conf.
10 ppm	60	even

Adduction(s): H, Na **Neutral Loss(es):** H2O

Compound Verification Results

Expected Formula: C13H19N5O2

#	meas. m/z	theo. m/z	|err| [ppm]	mSigma	Formula	Modification	Purity(UVC)[%]	Purity(BPC)[%]
3	278.1619	278.1612	2.8	59	C13 H20 N5 O2	(M+H)+	95.7	67.5

Note: mSigma values <20 indicate high probability of correct molecular formula

PROTON.d MeOD {C:\Bruker\TopSpin3.2} IG500 13

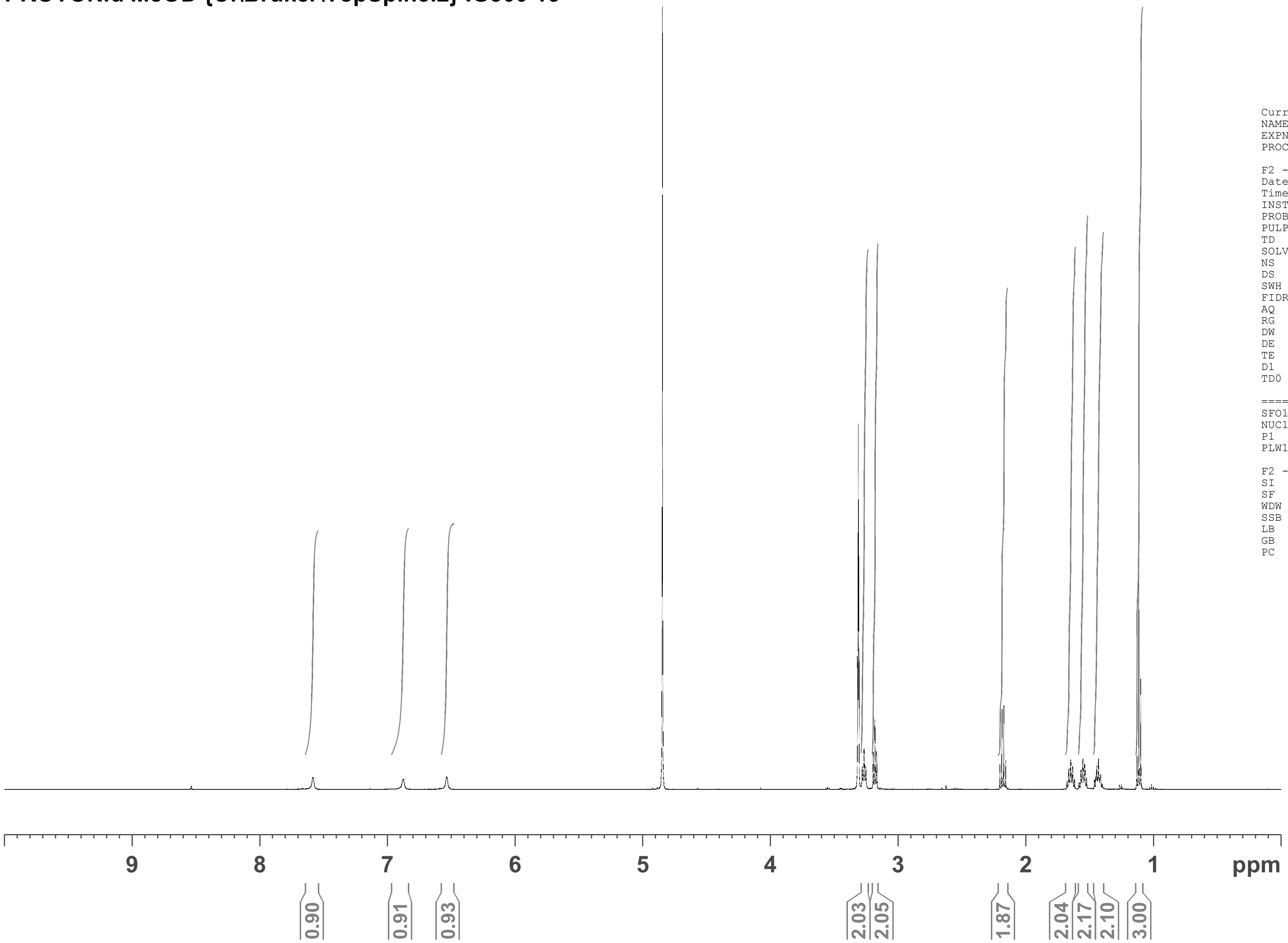


```
Current Data Parameters
NAME      IG-JM-200398-110-P
EXPNO                    1
PROCNO                   1

F2 - Acquisition Parameters
Date_             20170321
Time                 20.14
INSTRUM              spect
PROBHD   5 mm QNP 1H/13
PULPROG               zg30
TD                   65536
SOLVENT               MeOD
NS                      32
DS                       2
SWH              10000.000 Hz
FIDRES            0.152588 Hz
AQ               3.2767999 sec
RG                     512
DW                  50.000 usec
DE                    6.50 usec
TE                   298.2 K
D1              1.00000000 sec
TD0                      1

======== CHANNEL f1 ========
SFO1           500.1330885 MHz
NUC1                    1H
P1                   10.00 usec
PLW1           25.00000000 W

F2 - Processing parameters
SI                   65536
SF             500.1307511 MHz
WDW                     EM
SSB      0
LB                    0.30 Hz
GB       0
PC                    1.00
```

Compound 52

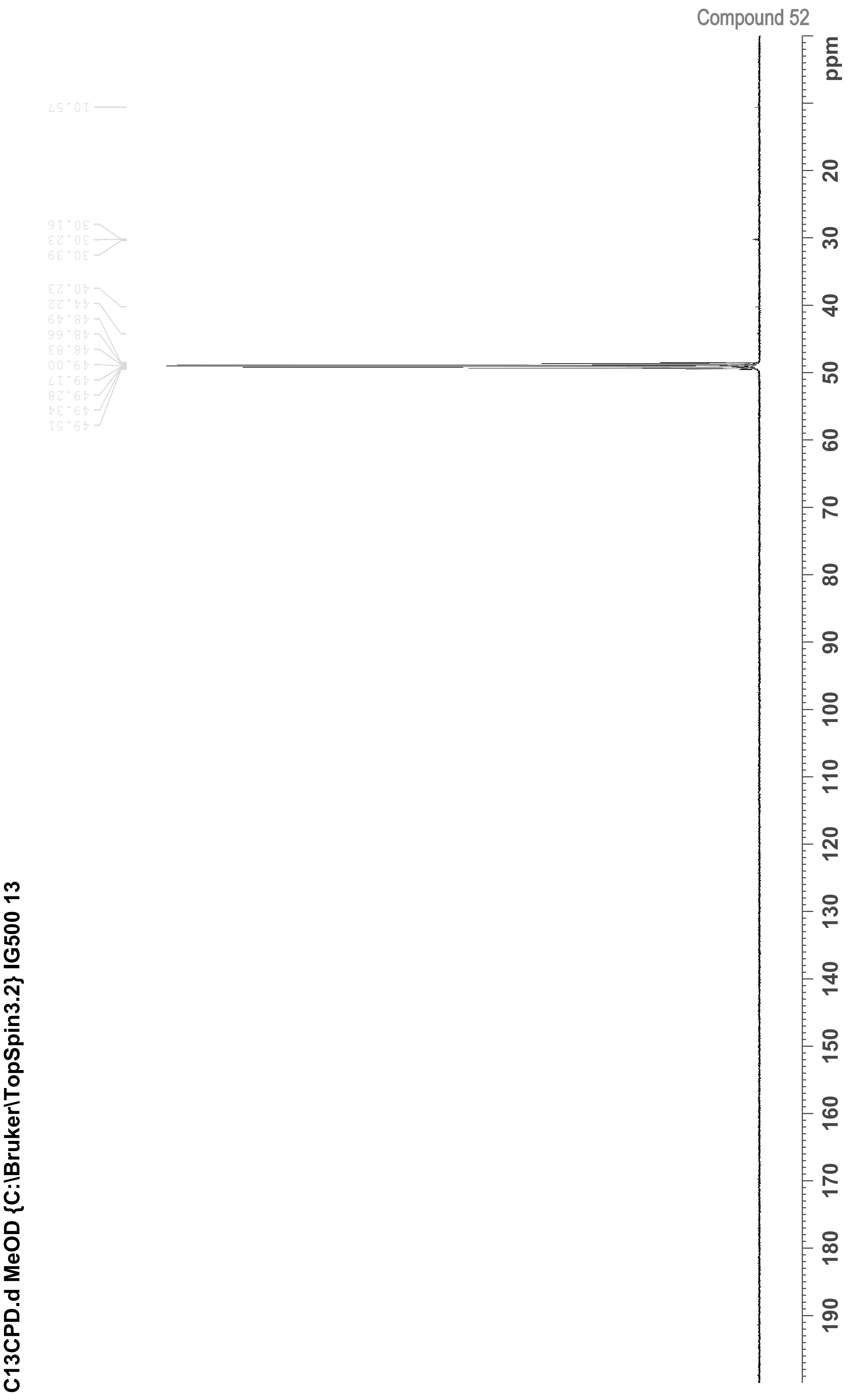
Compound 52
C13CPD.d MeOD {C:\Bruker\TopSpin3.2} IG500 13
ppm
10.57
30.16
30.23
30.39
40.23
44.22
48.49
48.66
48.83
49.00
49.17
49.28
49.34
49.51
20
30
40
50
60
70
80
90
100
110
120
130
140
150
160
170
180
190

Compound 52

Mass Spectrum SmartFormula Report

Analysis Info

Analysis Name	D:\Data\james\JM-200398-110-001_11974_RD5_01_13357.d	Acquisition Date	23/03/2017 15:49:55	
Method	2-microtof-2 verify compounds lcms pos 5-95.m	Operator	Dundee University	
Sample Name	JM-200398-110-001_11974	Instrument / Ser#	micrOTOF II	8213750.10435
Comment				

Acquisition Parameter

Source Type	ESI	Ion Polarity	Positive	Set Nebulizer	1.0 Bar
Focus	Not active			Set Dry Heater	200 °C
Scan Begin	50 m/z	Set Capillary	4500 V	Set Dry Gas	10.0 l/min
Scan End	1200 m/z	Set End Plate Offset	-500 V	Set Divert Valve	Source

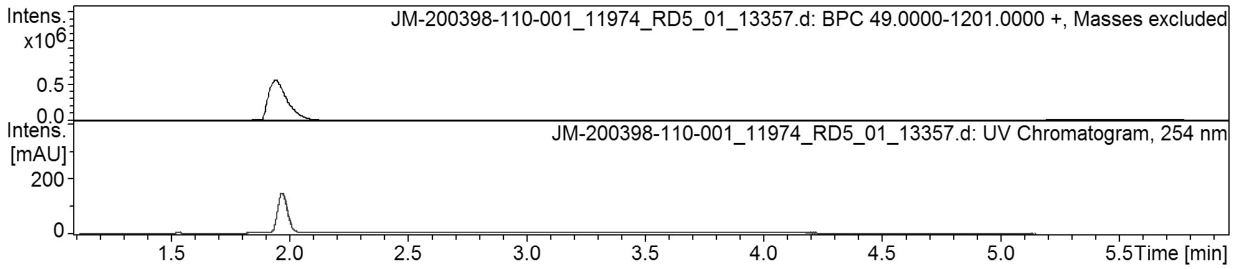

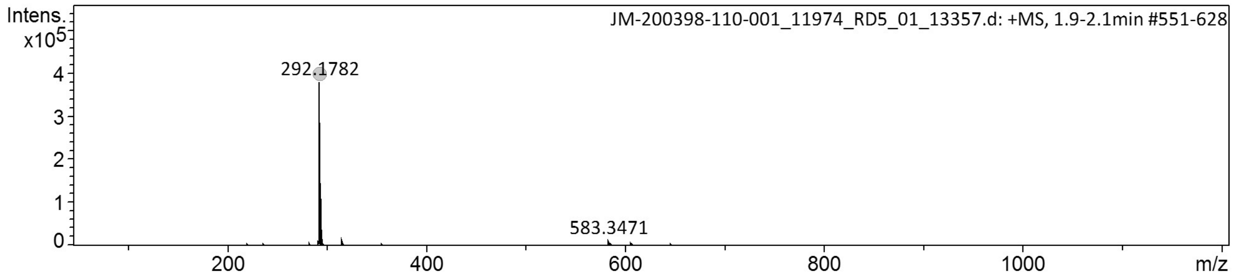

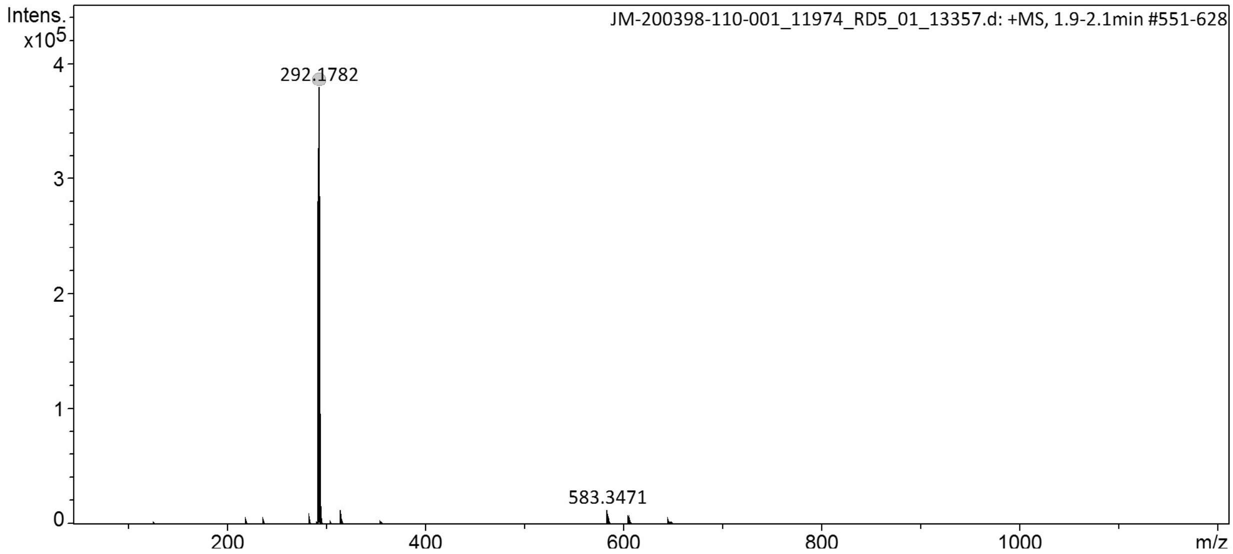

Meas. m/z	#	Ion Formula	m/z	err [mDa]	err [ppm]	Mean err [ppm]	rdb	N-Rule	e⁻ Conf	mSigma
292.178217	1	C14H22N5O2	292.176801	-1.4	-4.8	-5.6	7.0	ok	even	125.0

PROTON.d MeOD {C:\Bruker\TopSpin3.2} IG500 3

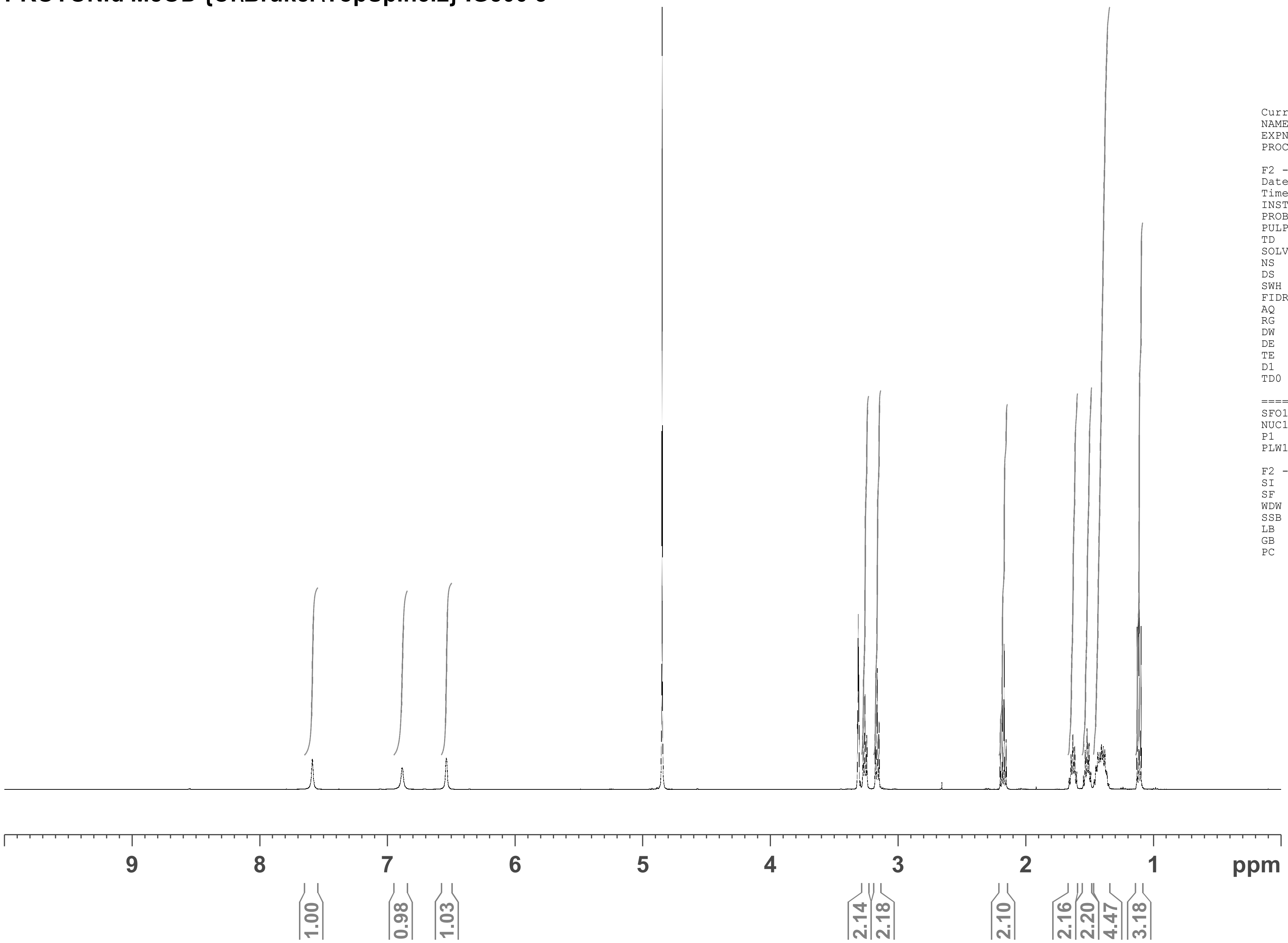


```
Current Data Parameters
NAME       IG-JM-200398-100-P
EXPNO                       1
PROCNO                      1

F2 - Acquisition Parameters
Date_              20170320
Time                  17.49
INSTRUM               spect
PROBHD   5 mm QNP 1H/13
PULPROG                zg30
TD                    65536
SOLVENT                MeOD
NS                       32
DS                        2
SWH            10000.000 Hz
FIDRES          0.152588 Hz
AQ             3.2767999 sec
RG                      362
DW                50.000 usec
DE                  6.50 usec
TE                 298.2 K
D1            1.00000000 sec
TD0                       1

======== CHANNEL f1 ========
SFO1         500.1330885 MHz
NUC1                     1H
P1                10.00 usec
PLW1        25.00000000 W

F2 - Processing parameters
SI                    65536
SF           500.1299916 MHz
WDW                      EM
SSB      0
LB                   0.30 Hz
GB       0
PC                     1.00
```

Compound 53

Compound 53

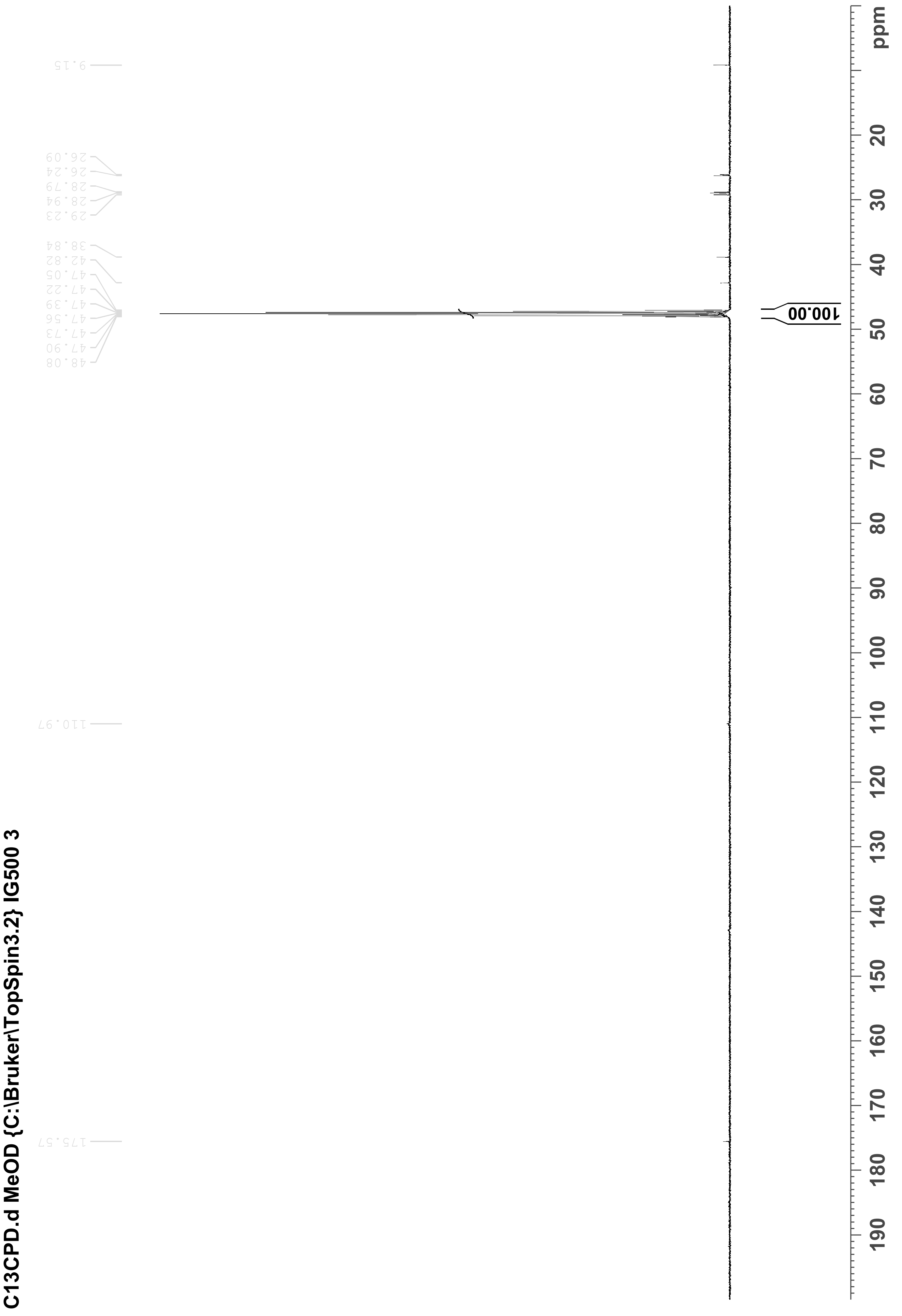
C13CPD.d MeOD {C:\Bruker\TopSpin3.2} IG500 3
9.15
26.09
26.24
28.79
28.94
29.23
38.84
42.82
47.05
47.22
47.39
47.56
47.73
47.90
48.08
110.97
175.57
100.00
ppm
20
30
40
50
60
70
80
90
100
110
120
130
140
150
160
170
180
190

Compound 53

Mass Spectrum SmartFormula Report

Analysis Info

Analysis Name	D:\Data\james\JM-200398-100-001_11962_RC1_01_13345.d	Acquisition Date	23/03/2017 14:06:22
Method	2-microtof-2 verify compounds lcms pos 5-95.m	Operator	Dundee University
Sample Name	JM-200398-100-001_11962	Instrument / Ser#	micrOTOF II 8213750.10435
Comment			

Acquisition Parameter

Source Type	ESI	Ion Polarity	Positive	Set Nebulizer	1.0 Bar
Focus	Not active			Set Dry Heater	220 °C
Scan Begin	50 m/z	Set Capillary	4500 V	Set Dry Gas	10.0 l/min
Scan End	1200 m/z	Set End Plate Offset	-500 V	Set Divert Valve	Waste

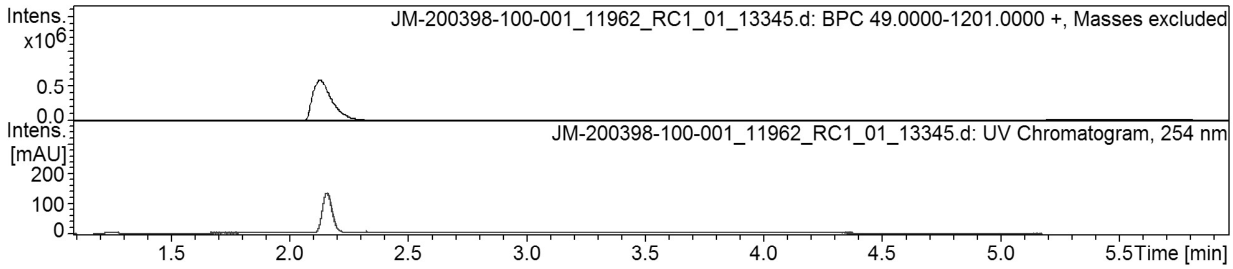

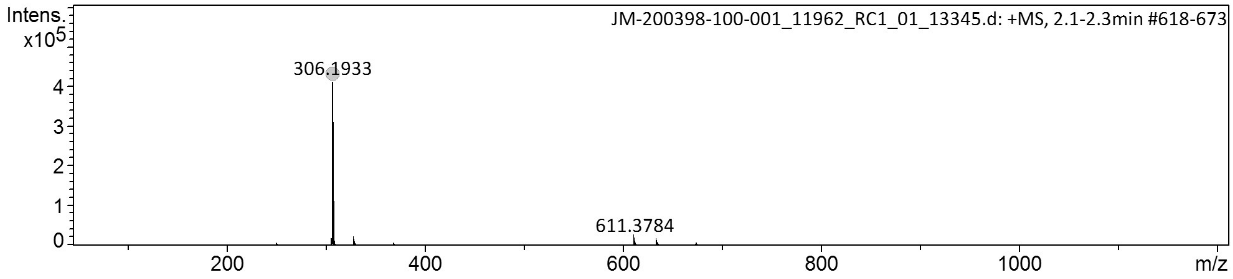

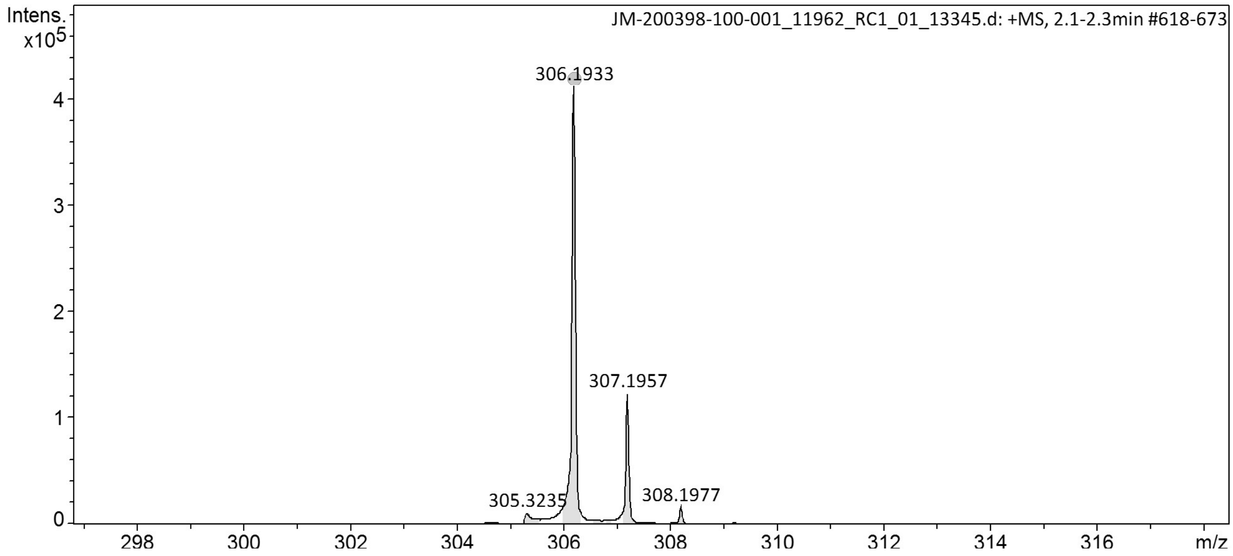

Meas. m/z	#	Ion Formula	m/z	err [mDa]	err [ppm]	Mean err [ppm]	rdb	N-Rule	e⁻ Conf	mSigma
306.193296	1	C15H24N5O2	306.192451	-0.8	-2.8	-1.9	7.0	ok	even	66.3

Compound 54

PROTON.d MeOD {C:\Bruker\TopSpin3.2} IG500 1

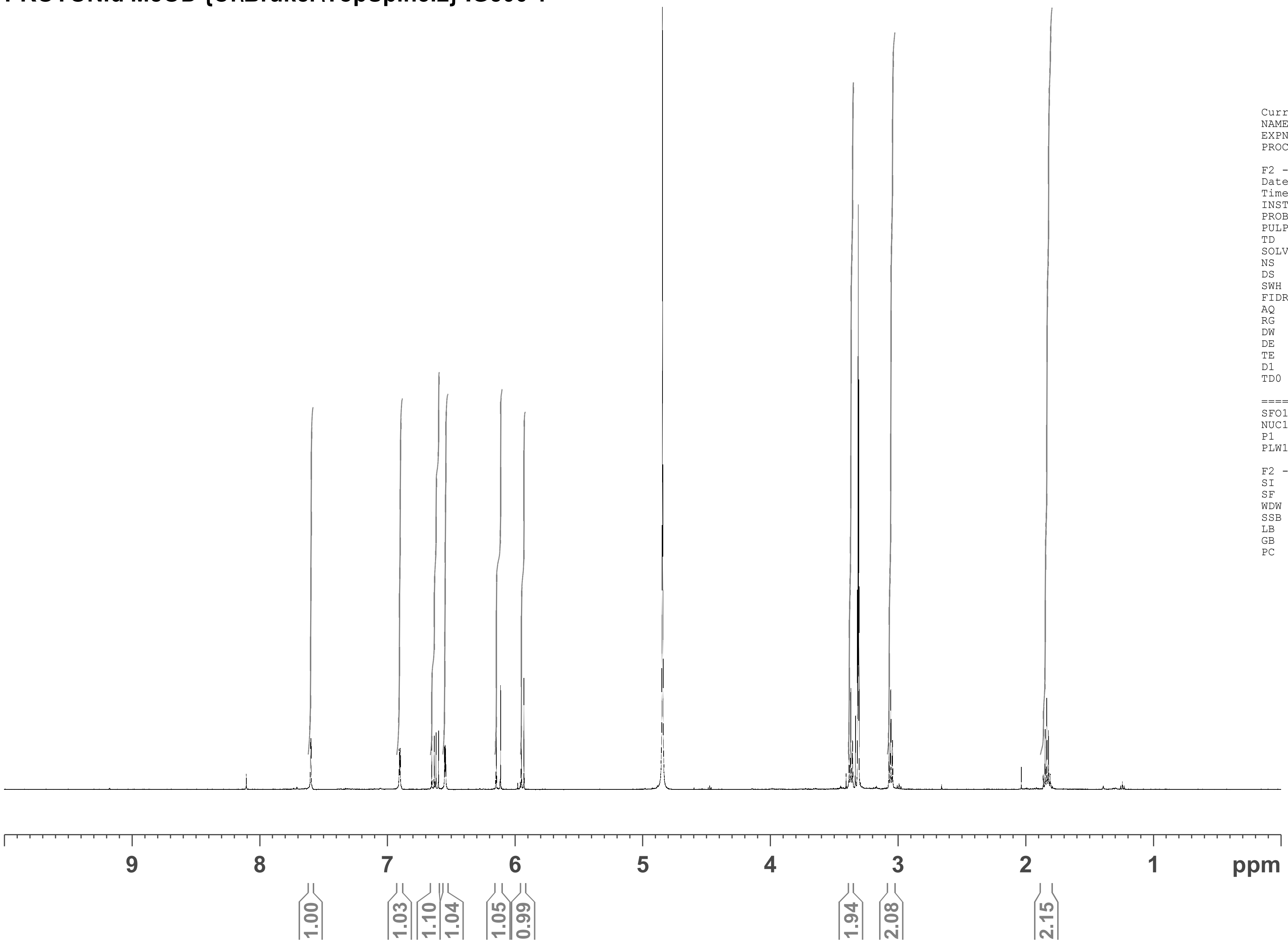


```
Current Data Parameters
NAME       IG-JM-200398-120-P2
EXPNO                     1
PROCNO                    1

F2 - Acquisition Parameters
Date_           20170405
Time               14.54
INSTRUM            spect
PROBHD   5 mm QNP 1H/13
PULPROG             zg30
TD                 65536
SOLVENT             MeOD
NS                    16
DS                     2
SWH            10000.000 Hz
FIDRES          0.152588 Hz
AQ             3.2767999 sec
RG                   512
DW                50.000 usec
DE                  6.50 usec
TE                 298.2 K
D1            1.00000000 sec
TD0                    1

======== CHANNEL f1 ========
SFO1        500.1330885 MHz
NUC1                 1H
P1                10.00 usec
PLW1        25.00000000 W

F2 - Processing parameters
SI                 65536
SF           500.1307509 MHz
WDW                   EM
SSB      0
LB                  0.30 Hz
GB       0
PC                  1.00
```

C13CPD.d MeOD {C:\Bruker\TopSpin3.2} IG500 1

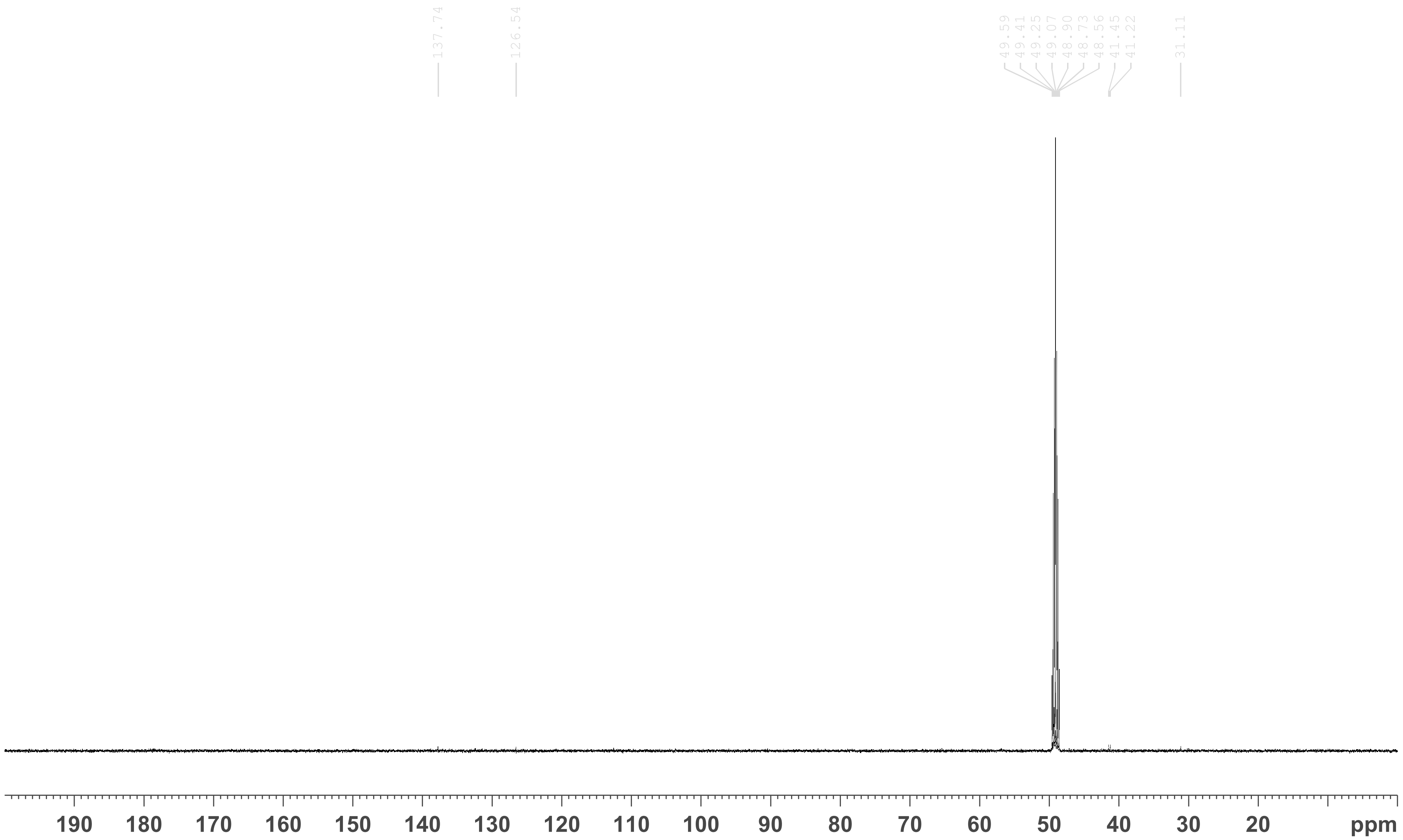

Compound 54

Compound Verification Report (Compass OpenAccess/QC)

Sample-ID	JM-200398-120-001	Station	Microtof-2
Submitter	James Martin	Supervisor	System Administrator
Analysis Name	JM-200398-120-001_12317_RB2_01_57.d	Acquisition Date	4/27/2017 5:23:54 PM
Sample Description			

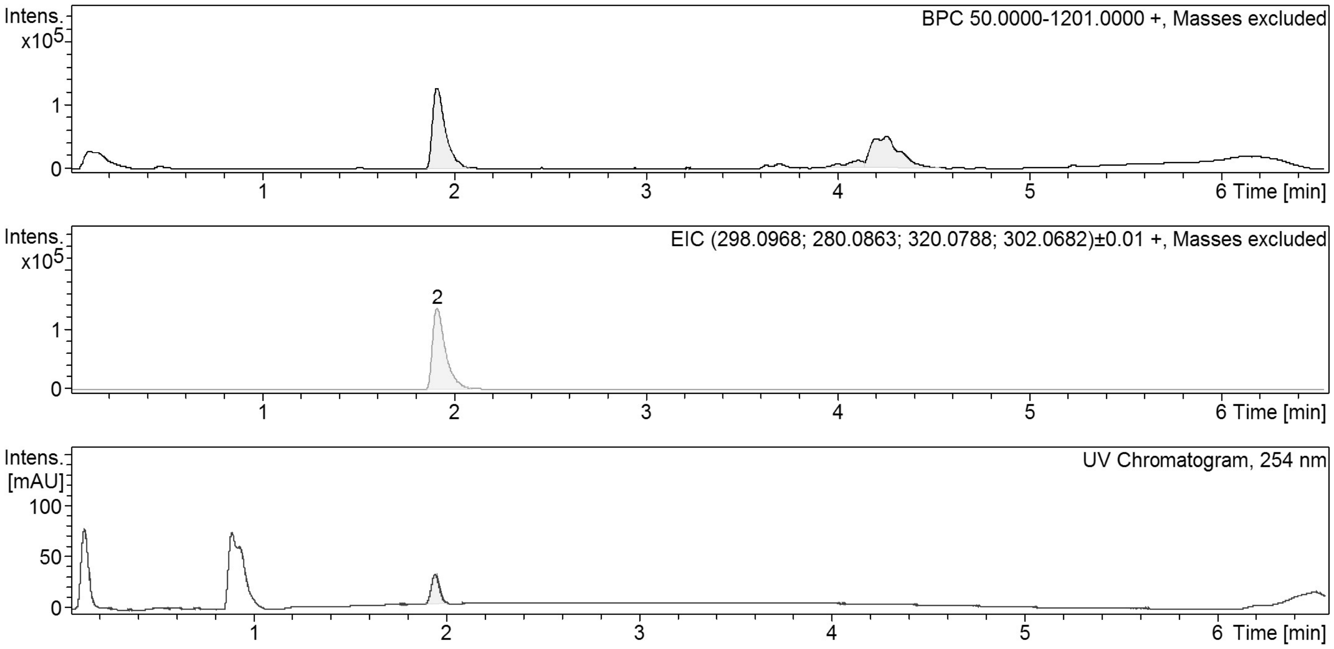

SmartFormula Settings

Tolerance	mSigma Limit	Electron Conf.
10 ppm	60	even

Adduction(s): H, Na **Neutral Loss(es):** H2O

Compound Verification Results

Expected Formula: C11H15N5O3S

#	meas. m/z	theo. m/z	|err| [ppm]	mSigma	Formula	Modification	Purity(UVC)[%]	Purity(BPC)[%]
2	298.0965	298.0968	1.3	5	C11 H16 N5 O3 S	(M+H)+	100.0	50.4

Note: mSigma values <20 indicate high probability of correct molecular formula

PROTON.d MeOD {C:\Bruker\TopSpin3.2} IG500 5

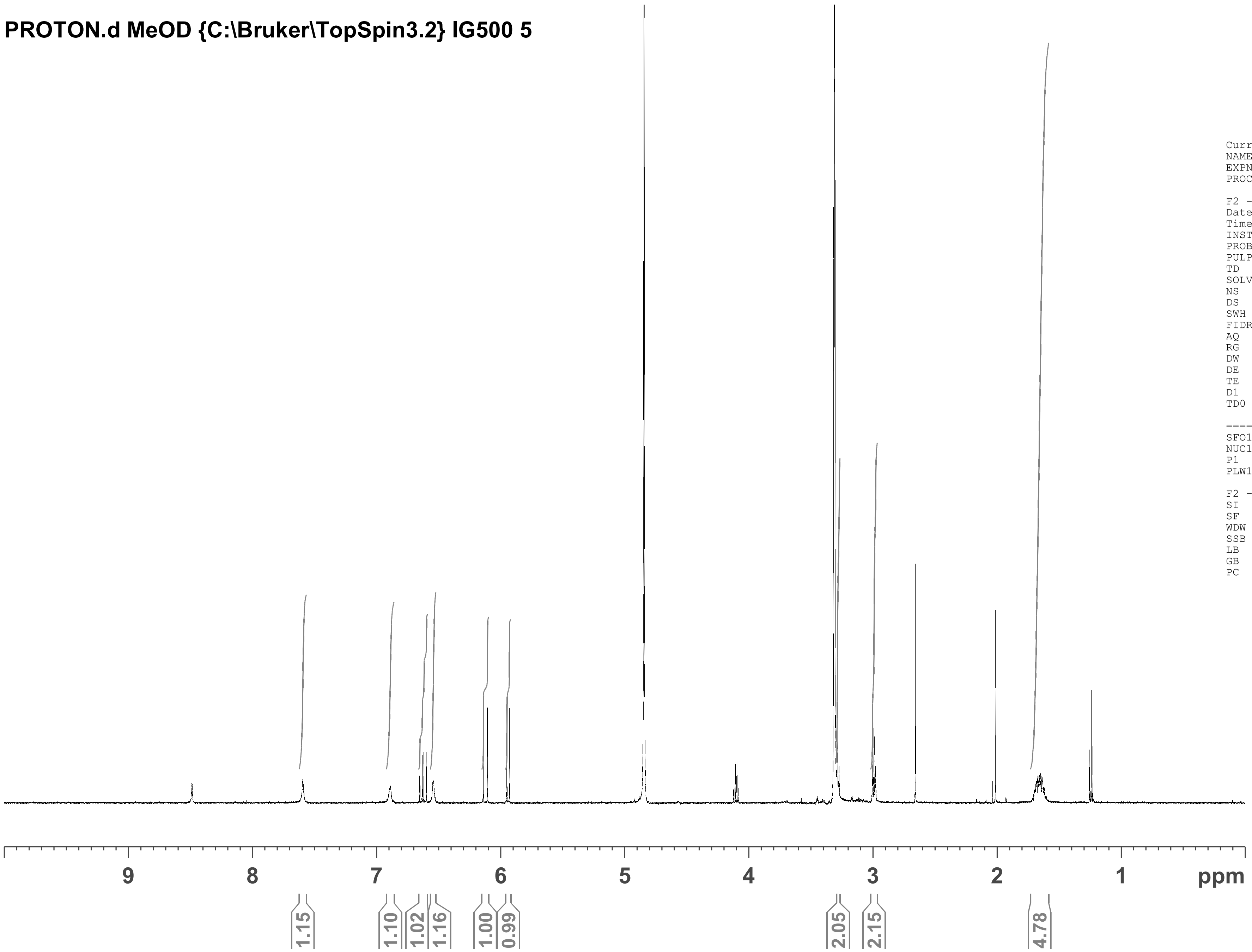


```
Current Data Parameters
NAME     IG-JM-200398-125-001
EXPNO                   1
PROCNO                  1

F2 - Acquisition Parameters
Date_           20170426
Time               13.53
INSTRUM            spect
PROBHD   5 mm QNP 1H/13
PULPROG             zg30
TD                 65536
SOLVENT             MeOD
NS                    16
DS                     2
SWH           10000.000 Hz
FIDRES         0.152588 Hz
AQ            3.2767999 sec
RG                   575
DW                50.000 usec
DE                  6.50 usec
TE                 298.2 K
D1           1.00000000 sec
TD0                    1

======== CHANNEL f1 ========
SFO1        500.1330885 MHz
NUC1                  1H
P1                 10.00 usec
PLW1        25.00000000 W

F2 - Processing parameters
SI                 65536
SF          500.1307510 MHz
WDW                   EM
SSB      0
LB                  0.30 Hz
GB       0
PC                  1.00
```

Compound 55

Compound Verification Report (Compass OpenAccess/QC)

Sample-ID	JM-200398-125-001	Station	Microtof-2
Submitter	James Martin	Supervisor	System Administrator
Analysis Name	JM-200398-125-001_12319_RB4_01_59.d	Acquisition Date	4/27/2017 5:40:50 PM
Sample Description			

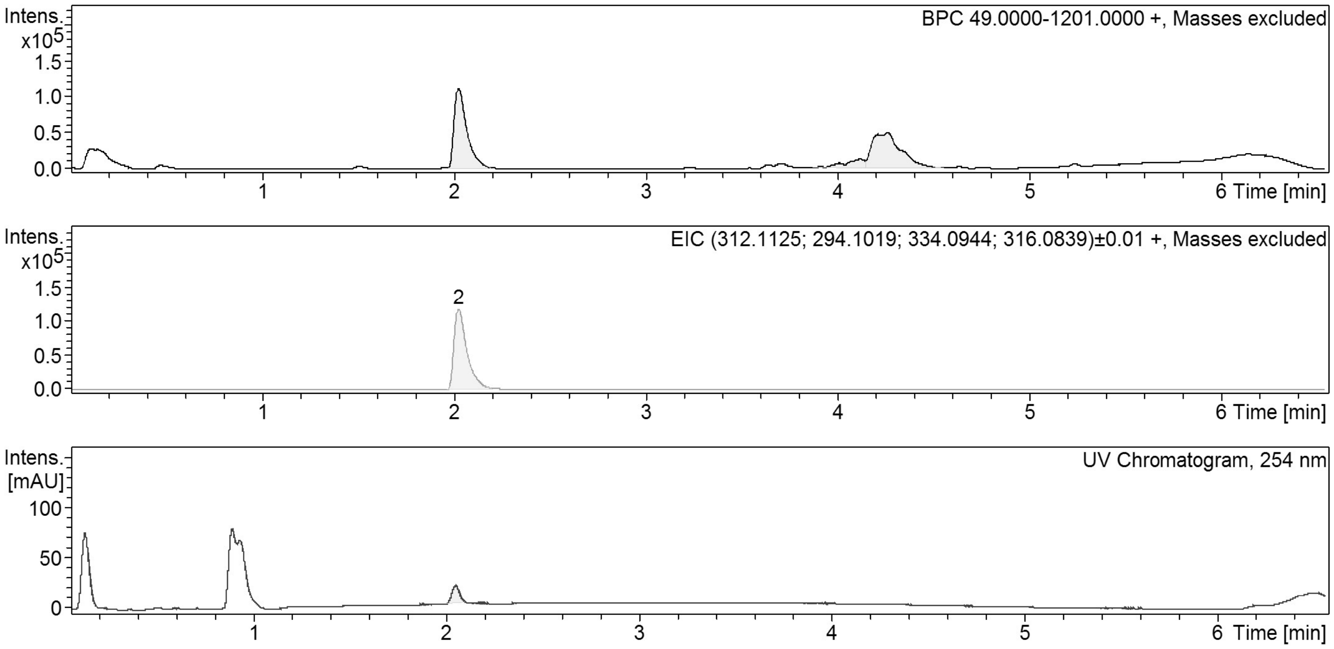

SmartFormula Settings

Tolerance	mSigma Limit	Electron Conf.
10 ppm	60	even

Adduction(s): H, Na **Neutral Loss(es):** H2O

Compound Verification Results

Expected Formula: C12H17N5O3S

#	meas. m/z	theo. m/z	|err| [ppm]	mSigma	Formula	Modification	Purity(UVC)[%]	Purity(BPC)[%]
2	312.1127	312.1125	0.8	1	C12 H18 N5 O3 S	(M+H)+	100.0	45.8

Note: mSigma values <20 indicate high probability of correct molecular formula

PROTON.d MeOD {C:\Bruker\TopSpin3.2} IG500 2

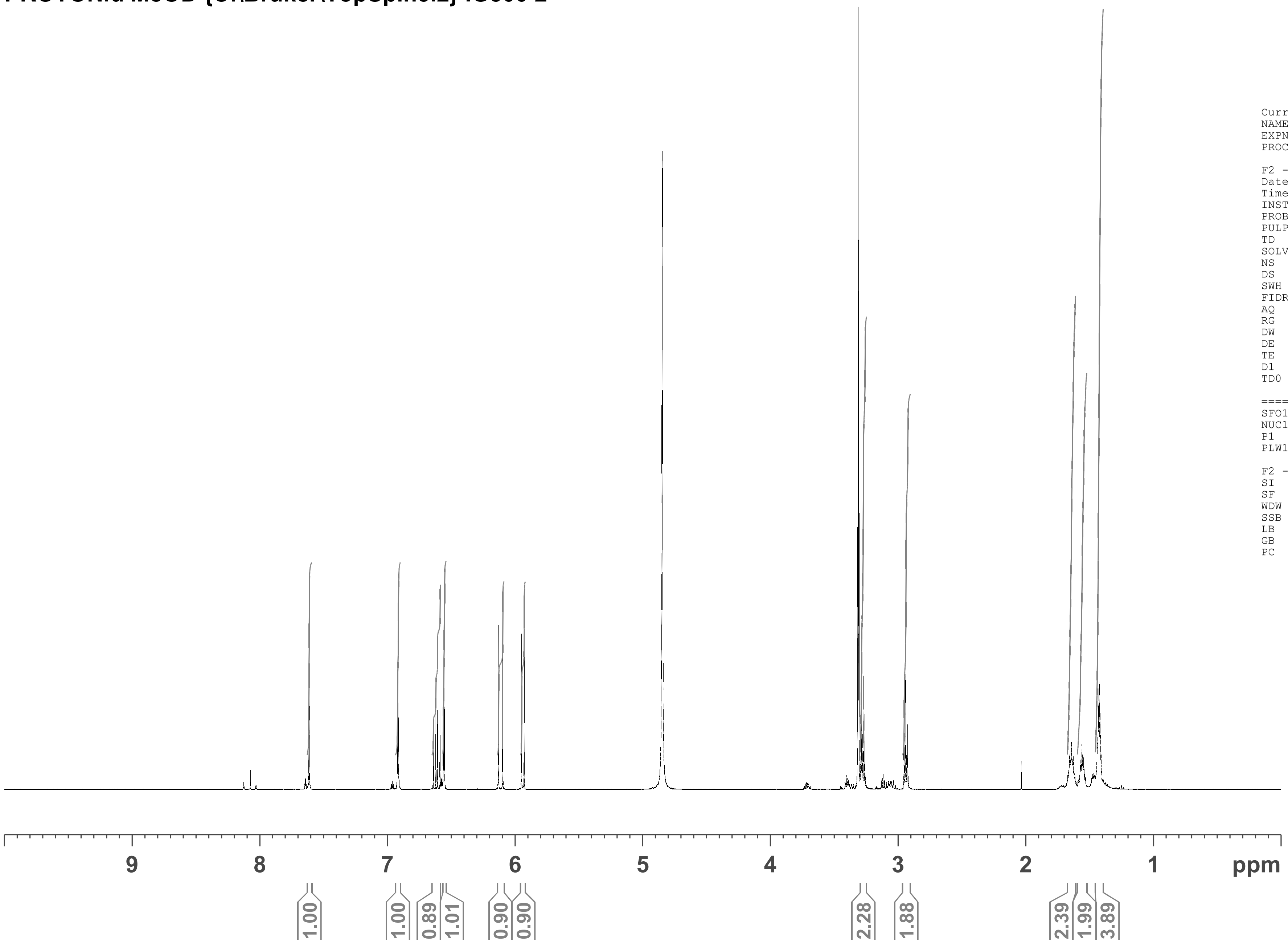


```
Current Data Parameters
NAME      IG-JM-200398-121-P2
EXPNO                   1
PROCNO                  1

F2 - Acquisition Parameters
Date_           20170405
Time               15.11
INSTRUM            spect
PROBHD   5 mm QNP 1H/13
PULPROG             zg30
TD                 65536
SOLVENT             MeOD
NS                    16
DS                     2
SWH            10000.000 Hz
FIDRES          0.152588 Hz
AQ             3.2767999 sec
RG                   456
DW                50.000 usec
DE                  6.50 usec
TE                 298.2 K
D1            1.00000000 sec
TD0                    1

======== CHANNEL f1 ========
SFO1         500.1330885 MHz
NUC1                  1H
P1                 10.00 usec
PLW1         25.00000000 W

F2 - Processing parameters
SI                 65536
SF           500.1307511 MHz
WDW                   EM
SSB       0
LB                  0.30 Hz
GB        0
PC                  1.00
```

Compound 57

C13CPD.d MeOD {C:\Bruker\TopSpin3.2} IG500 2

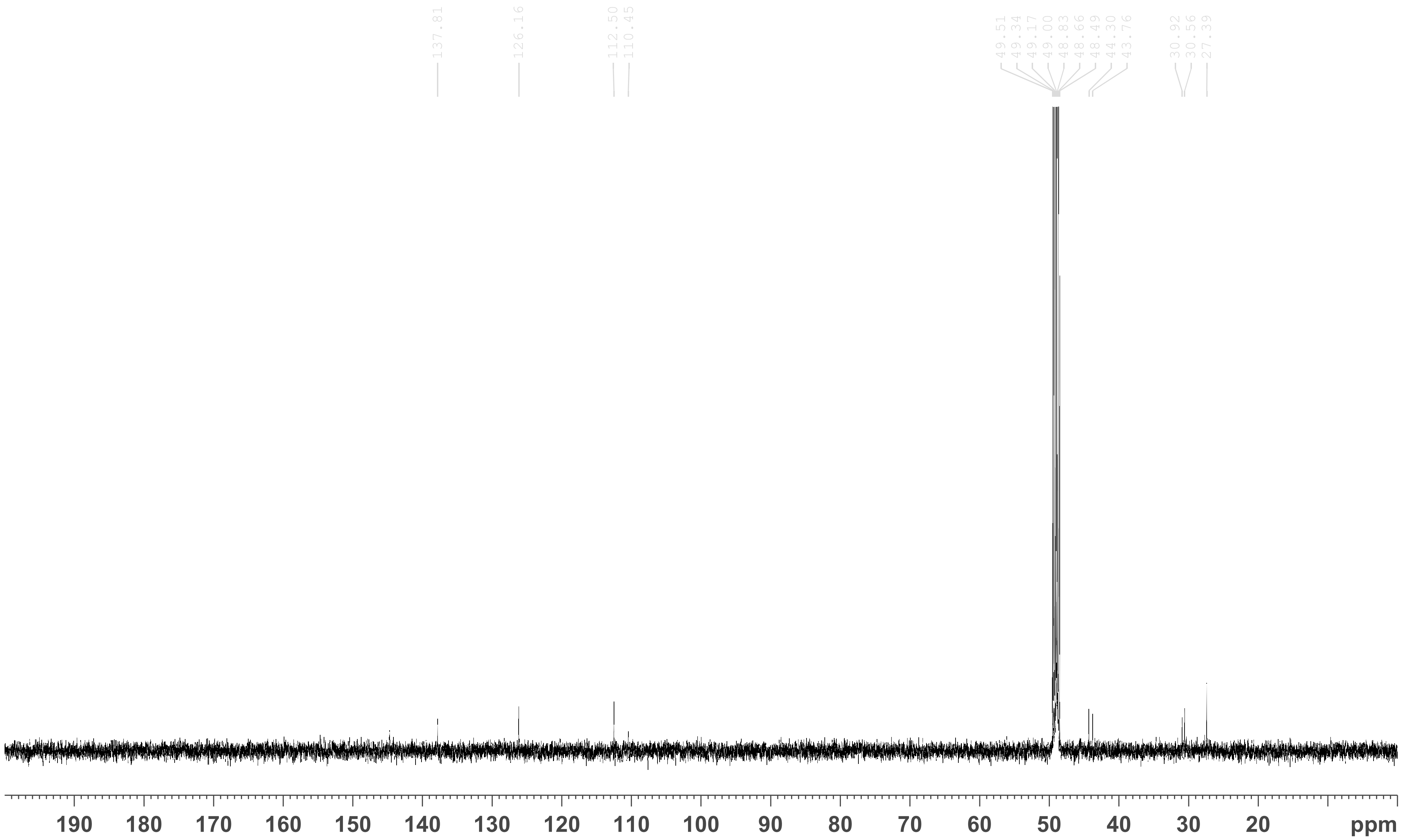

Compound 57

Compound Verification Report (Compass OpenAccess/QC)

Sample-ID	JM-200398-121-001	Station	Microtof-2
Submitter	James Martin	Supervisor	System Administrator
Analysis Name	JM-200398-121-001_12318_RB3_01_58.d	Acquisition Date	4/27/2017 5:32:23 PM
Sample Description			

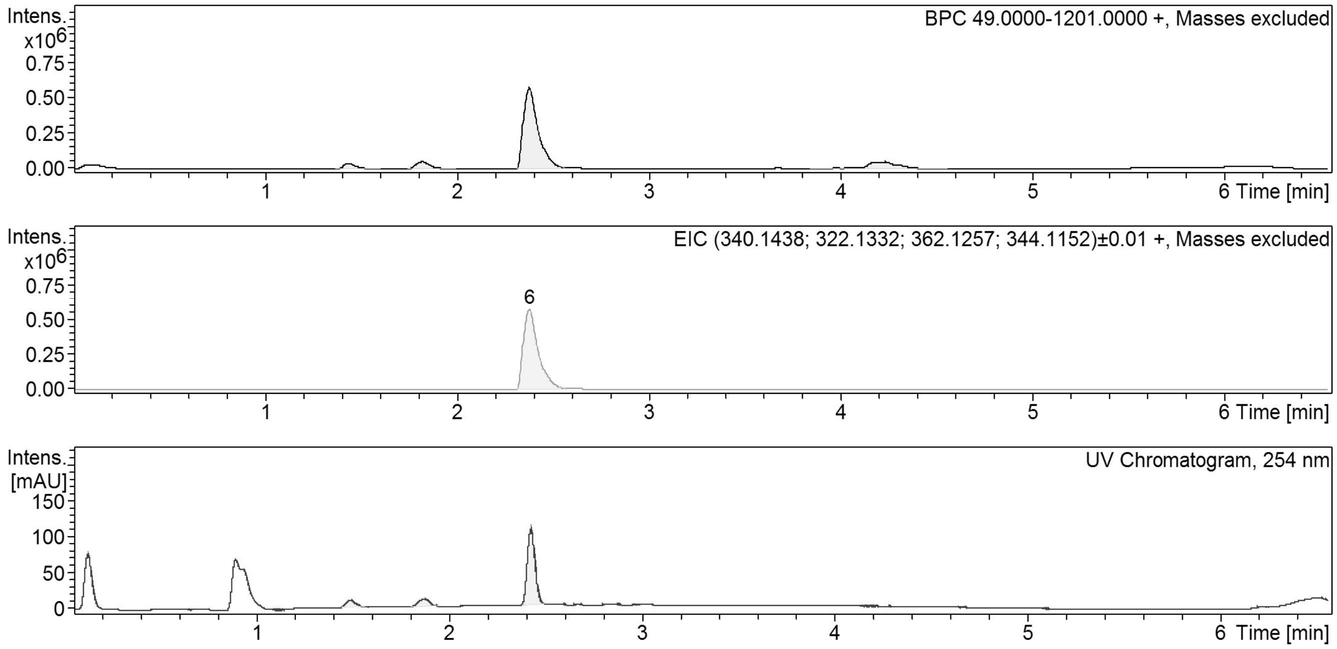

SmartFormula Settings

Tolerance	mSigma Limit	Electron Conf.
10 ppm	60	even

Adduction(s): H, Na **Neutral Loss(es):** H2O

Compound Verification Results

Expected Formula: C14H21N5O3S

#	meas. m/z	theo. m/z	|err| [ppm]	mSigma	Formula	Modification	Purity(UVC)[%]	Purity(BPC)[%]
6	340.1448	340.1438	3.0	45	C14 H22 N5 O3 S	(M+H)+	0.0	73.0

Note: mSigma values <20 indicate high probability of correct molecular formula

PROTON.d MeOD {C:\Bruker\TopSpin3.2} IG500 6

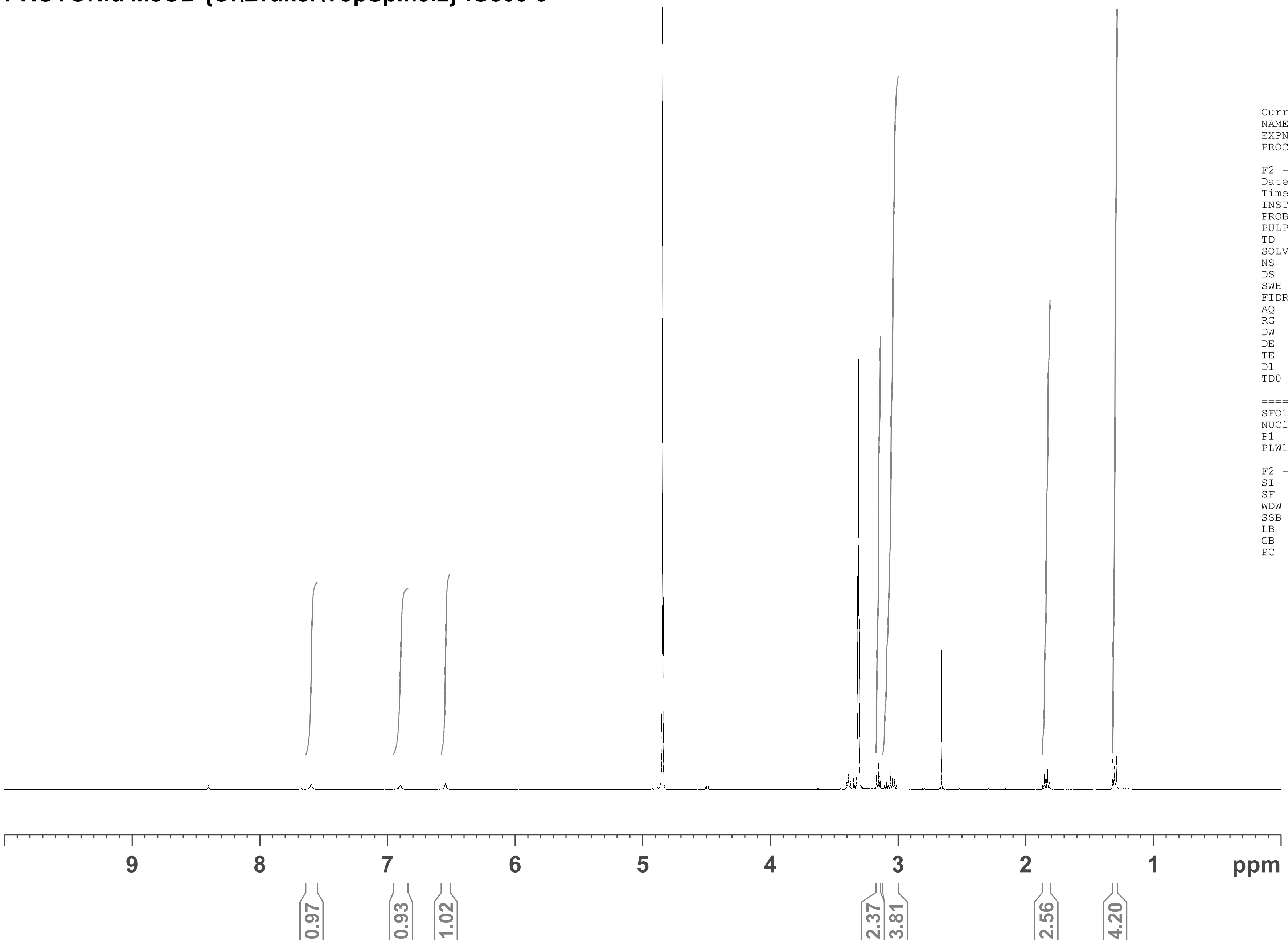


```
Current Data Parameters
NAME     IG-JM-200398-103-P
EXPNO                    1
PROCNO                   1

F2 - Acquisition Parameters
Date_          20170320
Time              20.42
INSTRUM           spect
PROBHD   5 mm QNP 1H/13
PULPROG            zg30
TD                65536
SOLVENT            MeOD
NS                   32
DS                    2
SWH           10000.000 Hz
FIDRES         0.152588 Hz
AQ            3.2767999 sec
RG                  645
DW               50.000 usec
DE                 6.50 usec
TE                298.2 K
D1           1.00000000 sec
TD0                   1

======== CHANNEL f1 ========
SFO1        500.1330885 MHz
NUC1                 1H
P1                10.00 usec
PLW1        25.00000000 W

F2 - Processing parameters
SI                65536
SF          500.1299936 MHz
WDW                  EM
SSB      0
LB                 0.30 Hz
GB       0
PC                 1.00
```

Compound 58

Mass Spectrum SmartFormula Report

Analysis Info

Analysis Name	D:\Data\james\JM-200398-103-001_11965_RC4_01_13348.d	Acquisition Date	23/03/2017 14:31:57
Method	2-microtof-2 verify compounds lcms pos 5-95.m	Operator	Dundee University
Sample Name	JM-200398-103-001_11965	Instrument / Ser#	micrOTOF II 8213750.10435
Comment			

Acquisition Parameter

Source Type	ESI	Ion Polarity	Positive	Set Nebulizer	1.0 Bar
Focus	Not active			Set Dry Heater	220 °C
Scan Begin	50 m/z	Set Capillary	4500 V	Set Dry Gas	10.0 l/min
Scan End	1200 m/z	Set End Plate Offset	-500 V	Set Divert Valve	Waste

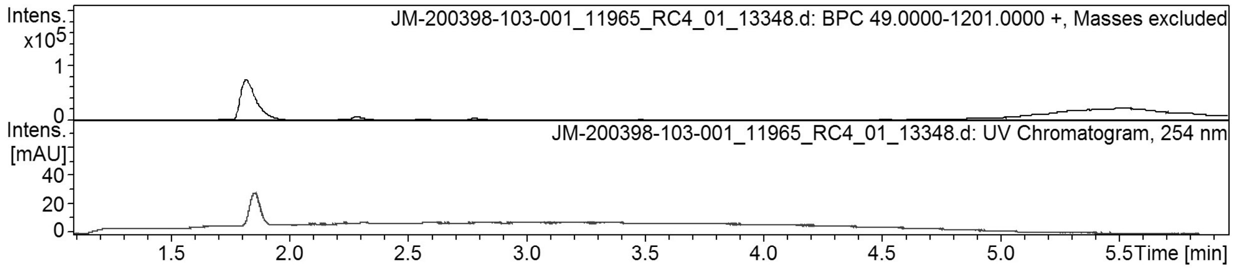

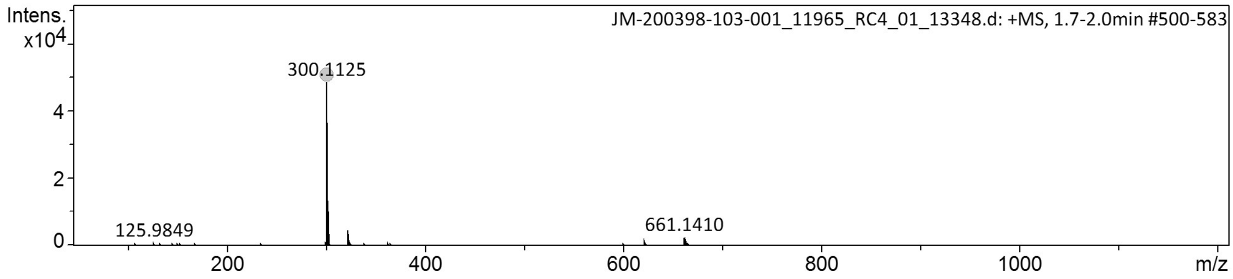

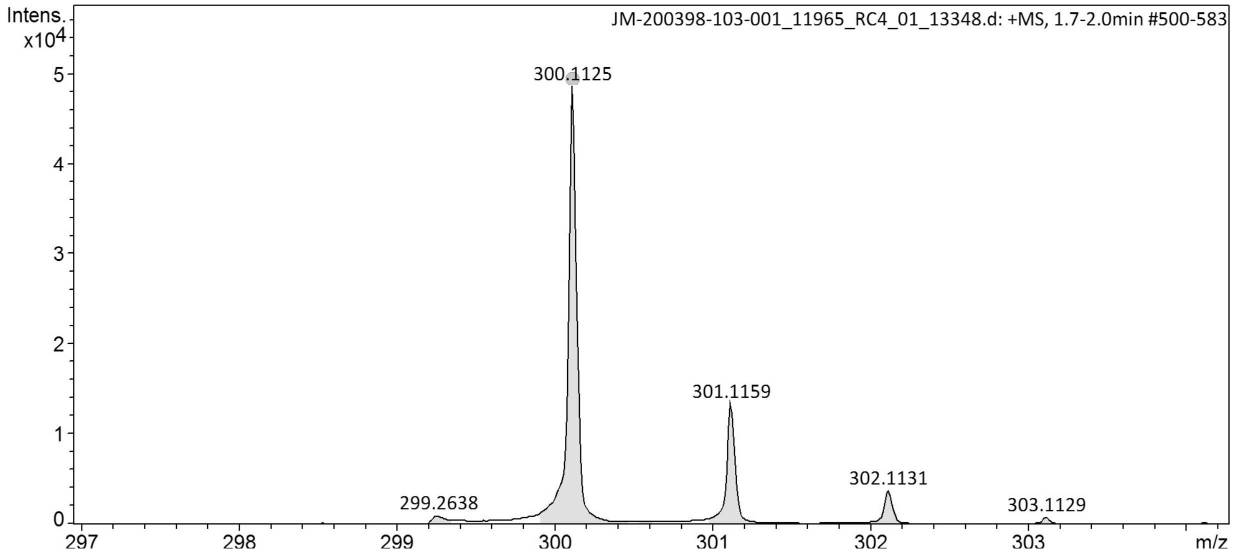

Meas. m/z	#	Ion Formula	m/z	err [mDa]	err [ppm]	Mean err [ppm]	rdb	N-Rule	e¯ Conf	mSigma
300.112514	1	C11H18N5O3S	300.112487	-0.0	-0.1	-2.5	6.0	ok	even	63.3

PROTON.d MeOD {C:\Bruker\TopSpin3.2} IG500 16

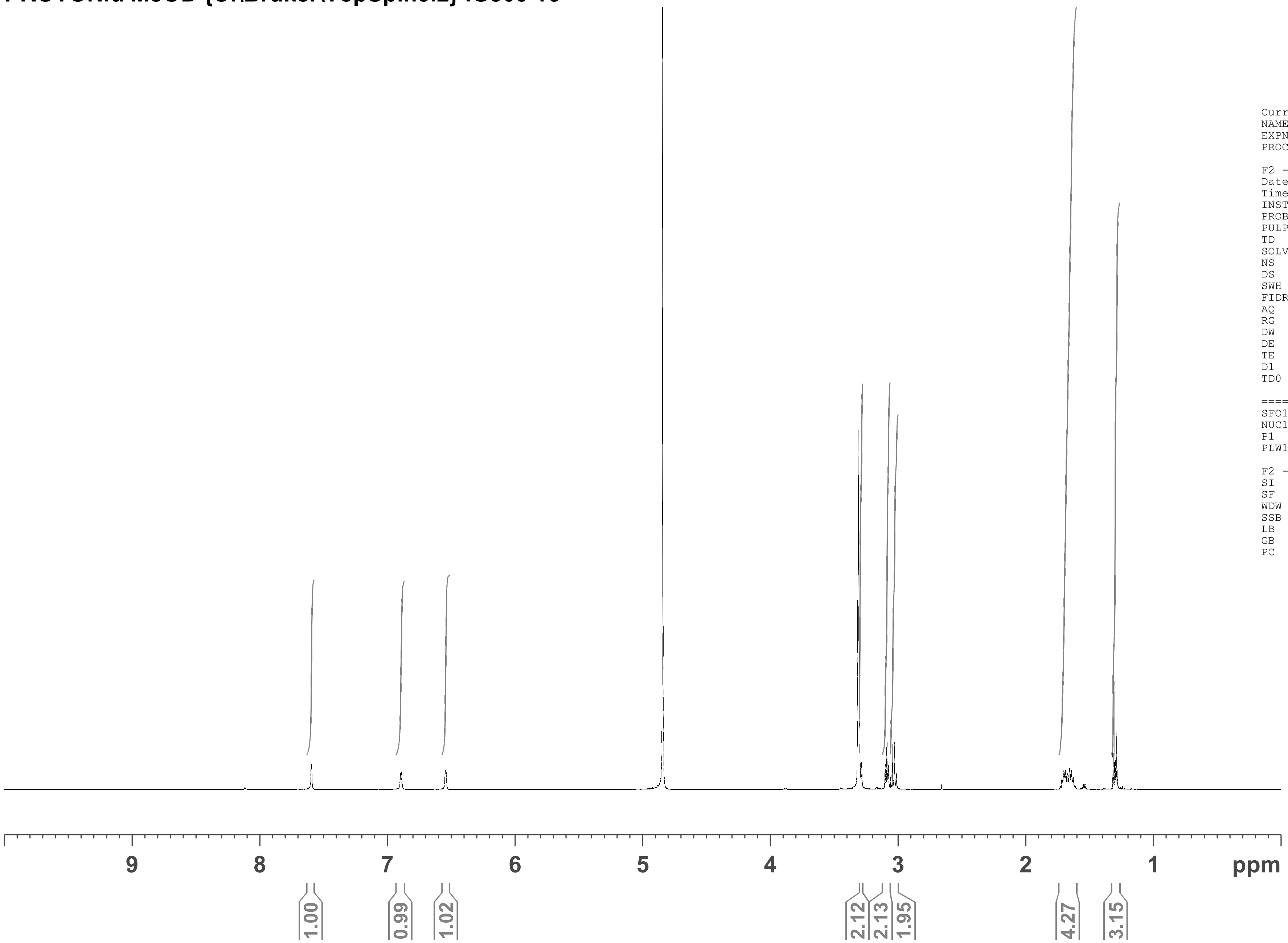


```
Current Data Parameters
NAME     IG-JM-200398-113-P
EXPNO                    1
PROCNO                   1

F2 - Acquisition Parameters
Date_            20170321
Time                23.08
INSTRUM             spect
PROBHD   5 mm QNP 1H/13
PULPROG              zg30
TD                  65536
SOLVENT              MeOD
NS                     32
DS                      2
SWH             10000.000 Hz
FIDRES           0.152588 Hz
AQ              3.2767999 sec
RG                    512
DW                 50.000 usec
DE                   6.50 usec
TE                  298.2 K
D1             1.00000000 sec
TD0                     1

======== CHANNEL f1 ========
SFO1          500.1330885 MHz
NUC1                   1H
P1                  10.00 usec
PLW1          25.00000000 W

F2 - Processing parameters
SI                  65536
SF            500.1307509 MHz
WDW                    EM
SSB      0
LB                   0.30 Hz
GB       0
PC                   1.00
```

Compound 59

C13CPD.d MeOD {C:\Bruker\TopSpin3.2} IG500 16

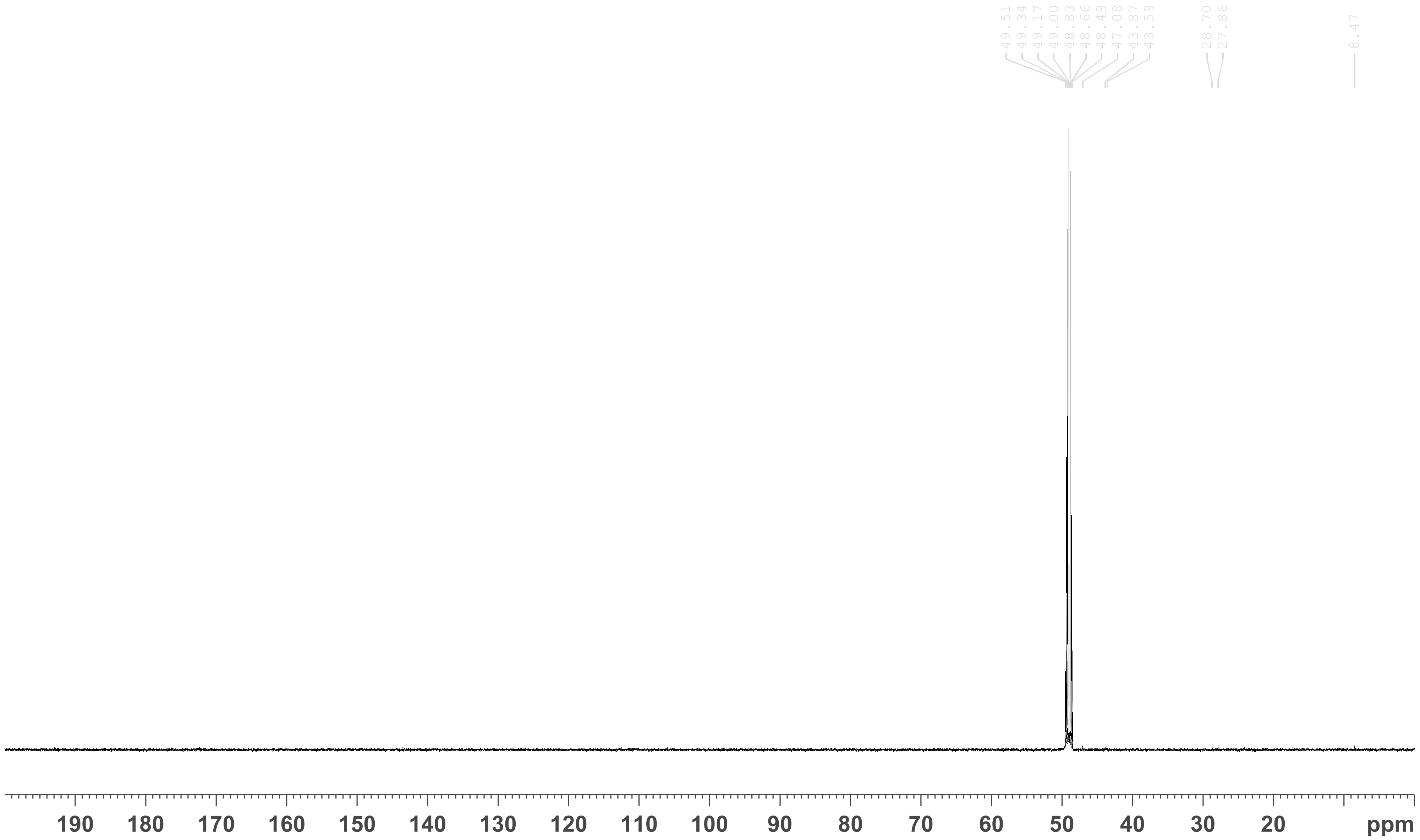

Compound 59

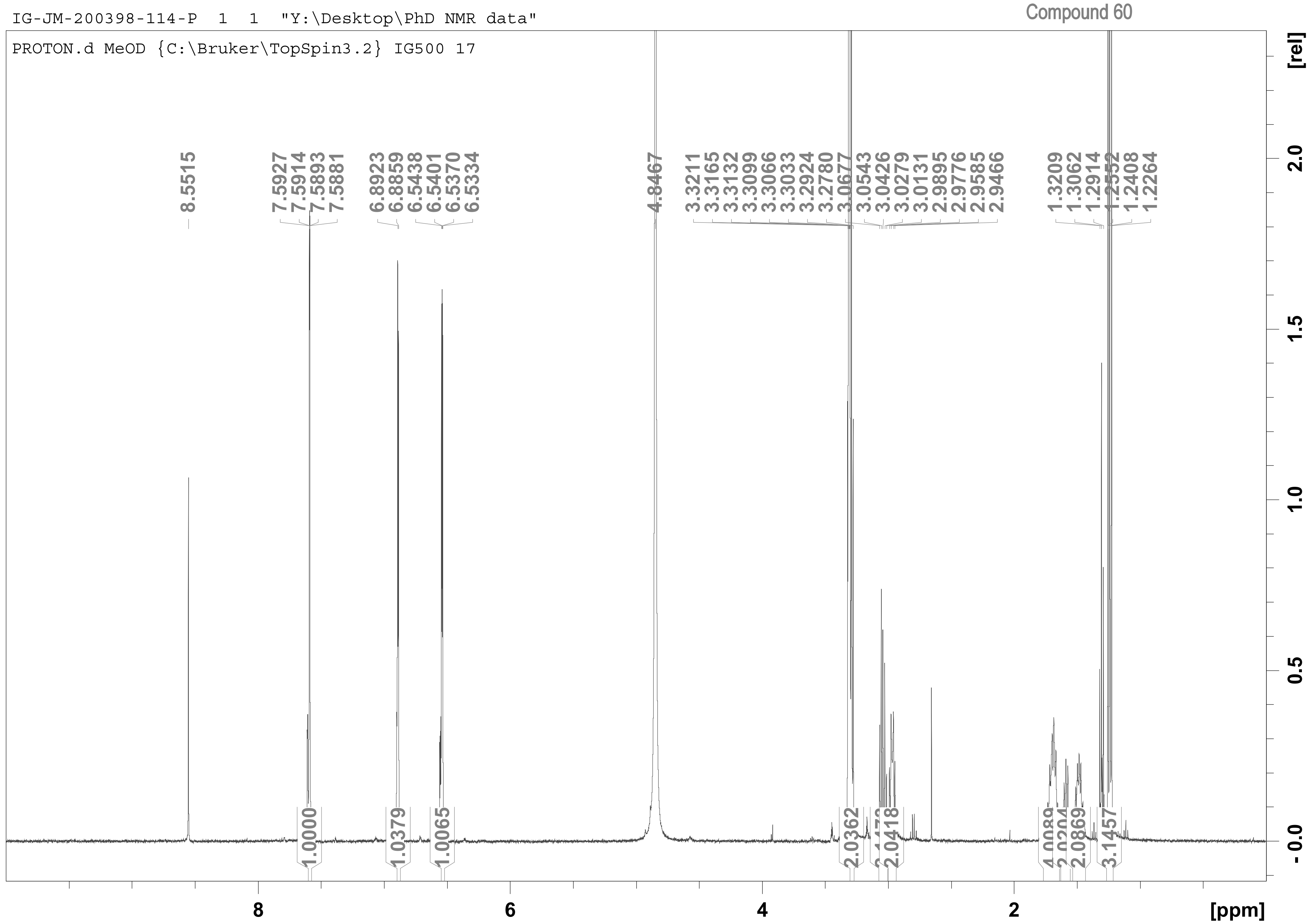

IG-JM-200398-114-P 1 1 "Y:\Desktop\PhD NMR data"
PROTON.d MeOD {C:\Bruker\TopSpin3.2} IG500 17
Compound 60
8.5515
7.5927
7.5914
7.5893
7.5881
6.8923
6.8859
6.5438
6.5401
6.5370
6.5334
4.8467
3.3211
3.3165
3.3132
3.3099
3.3066
3.3033
3.2924
3.2780
3.0677
3.0543
3.0426
3.0279
3.0131
2.9895
2.9776
2.9585
2.9466
1.3209
1.3062
1.2914
1.2552
1.2408
1.2264
1.0000
1.0379
1.0065
2.0362
2.0418
2.0869
3.1457
[rel]
2.0
1.5
1.0
0.5
-0.0
8
6
4
2
[ppm]

PROTON.d MeOD {C:\Bruker\TopSpin3.2} IG500 7

Compound 61

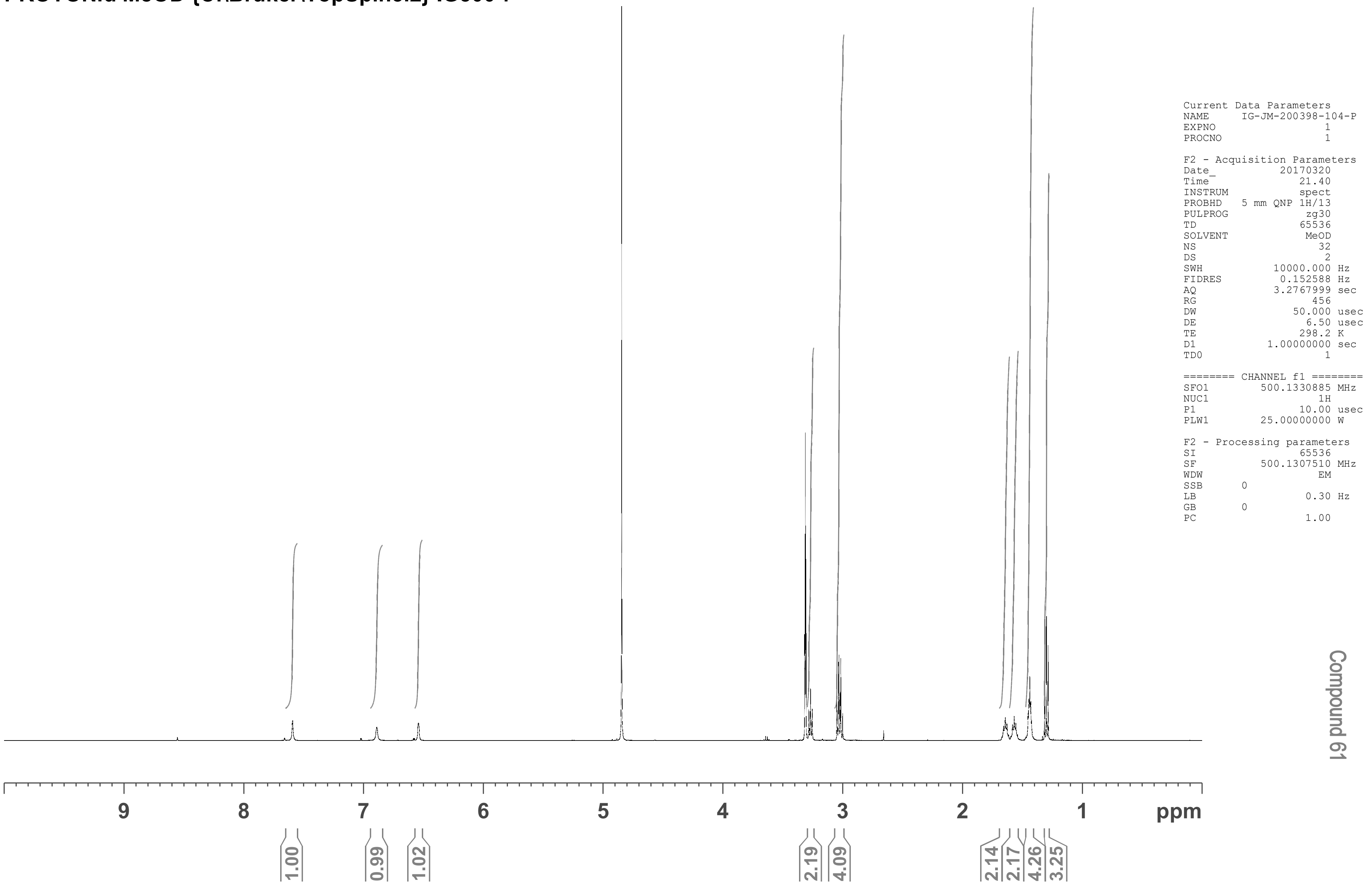

C13CPD.d MeOD {C:\Bruker\TopSpin3.2} IG500 7

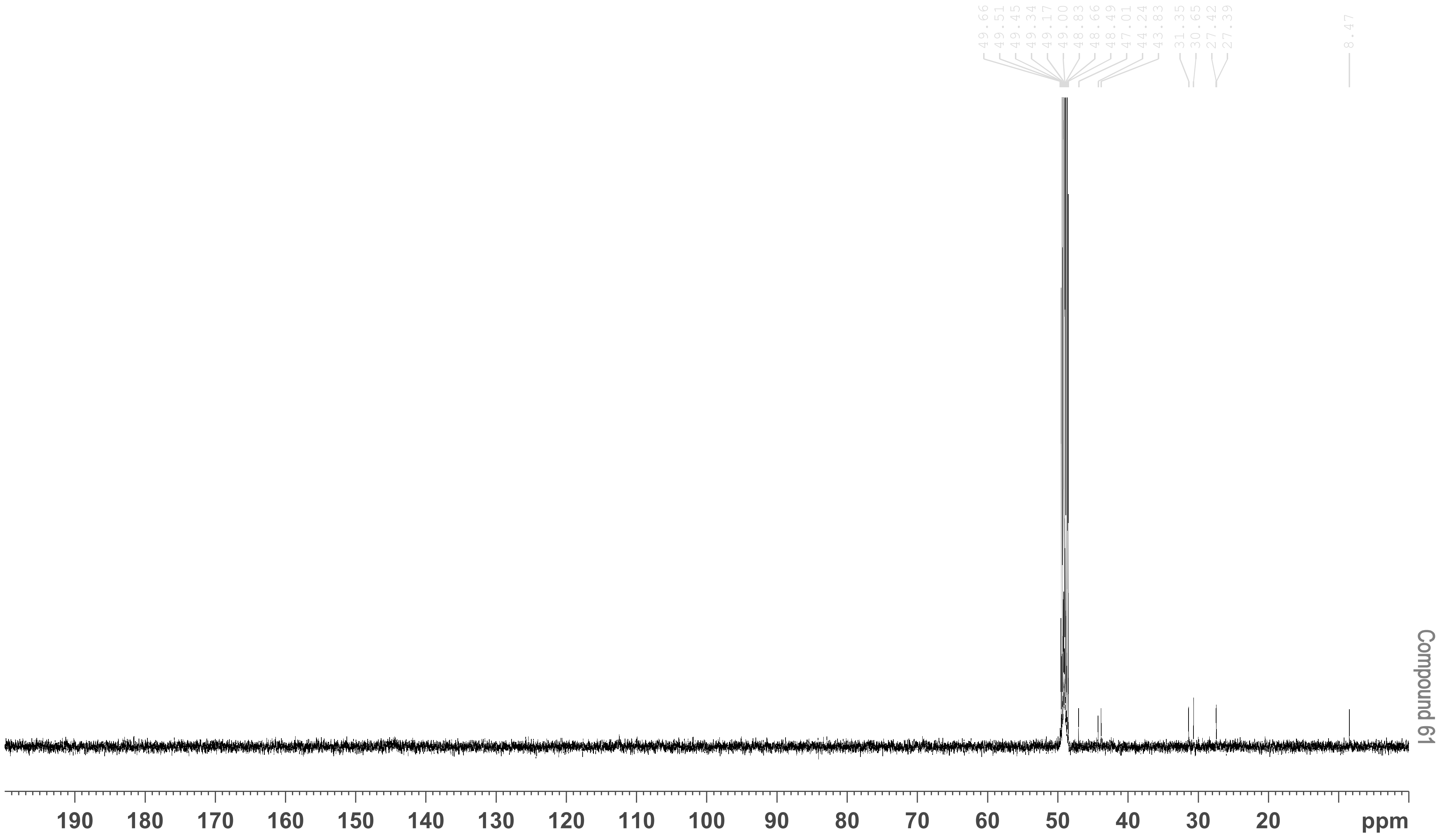

Mass Spectrum SmartFormula Report

Analysis Info

Analysis Name	D:\Data\james\JM-200398-104-001_11966_RC5_01_13349.d	Acquisition Date	23/03/2017 14:40:27
Method	2-microtof-2 verify compounds lcms pos 5-95.m	Operator	Dundee University
Sample Name	JM-200398-104-001_11966	Instrument / Ser#	micrOTOF II 8213750.10435
Comment			

Acquisition Parameter

Source Type	ESI	Ion Polarity	Positive	Set Nebulizer	1.0 Bar
Focus	Not active			Set Dry Heater	220 °C
Scan Begin	50 m/z	Set Capillary	4500 V	Set Dry Gas	10.0 l/min
Scan End	1200 m/z	Set End Plate Offset	-500 V	Set Divert Valve	Waste

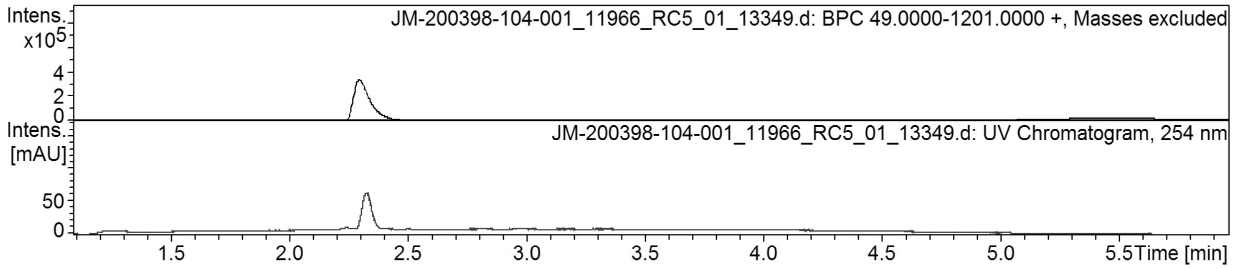

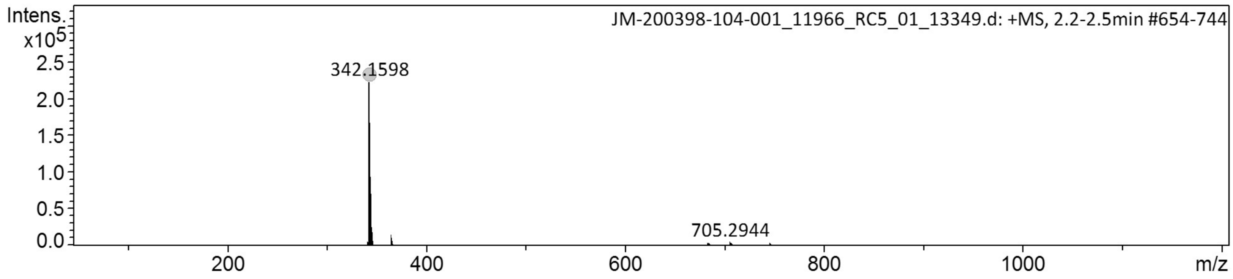

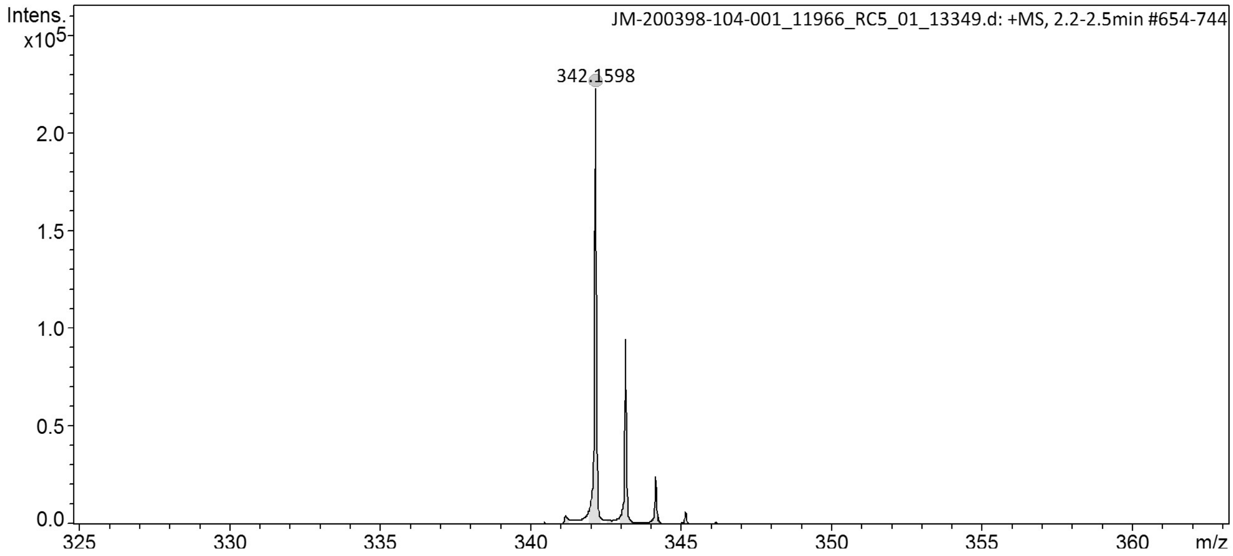

Meas. m/z	#	Ion Formula	m/z	err [mDa]	err [ppm]	Mean err [ppm]	rdb	N-Rule	e¯ Conf	mSigma
342.159757	1	C14H24N5O3S	342.159437	-0.3	-0.9	-4.2	6.0	ok	even	123.2

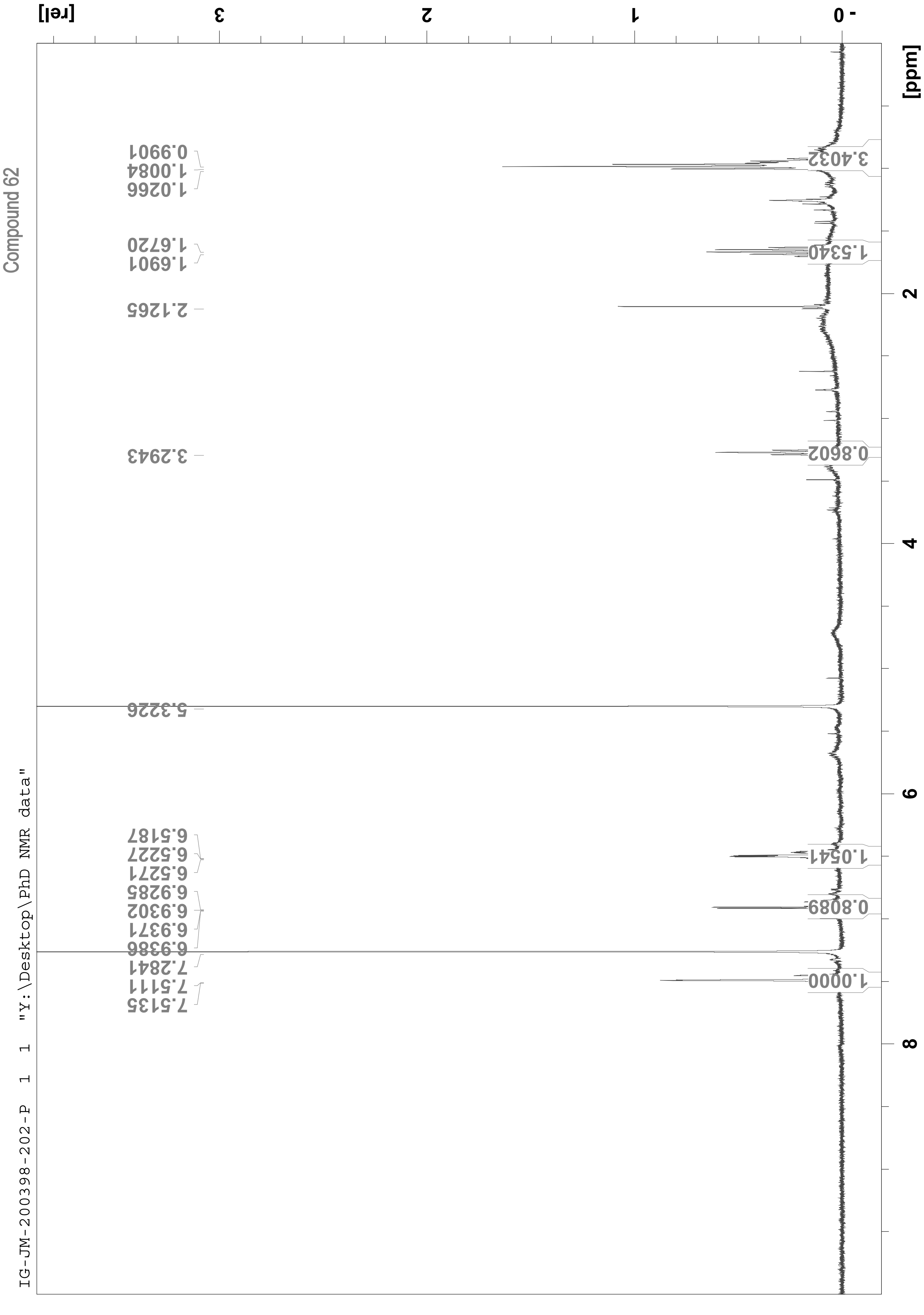

Compound 62
IG-JM-200398-202-P 1 1 "Y:\Desktop\PhD NMR data"
7.5135
7.5111
7.2841
6.9386
6.9371
6.9302
6.9285
6.5271
6.5227
6.5187
5.3226
3.2943
2.1265
1.6901
1.6720
1.0266
1.0084
0.9901
1.0000
0.8089
1.0541
0.8602
1.5340
3.4032
[rel]
[ppm]

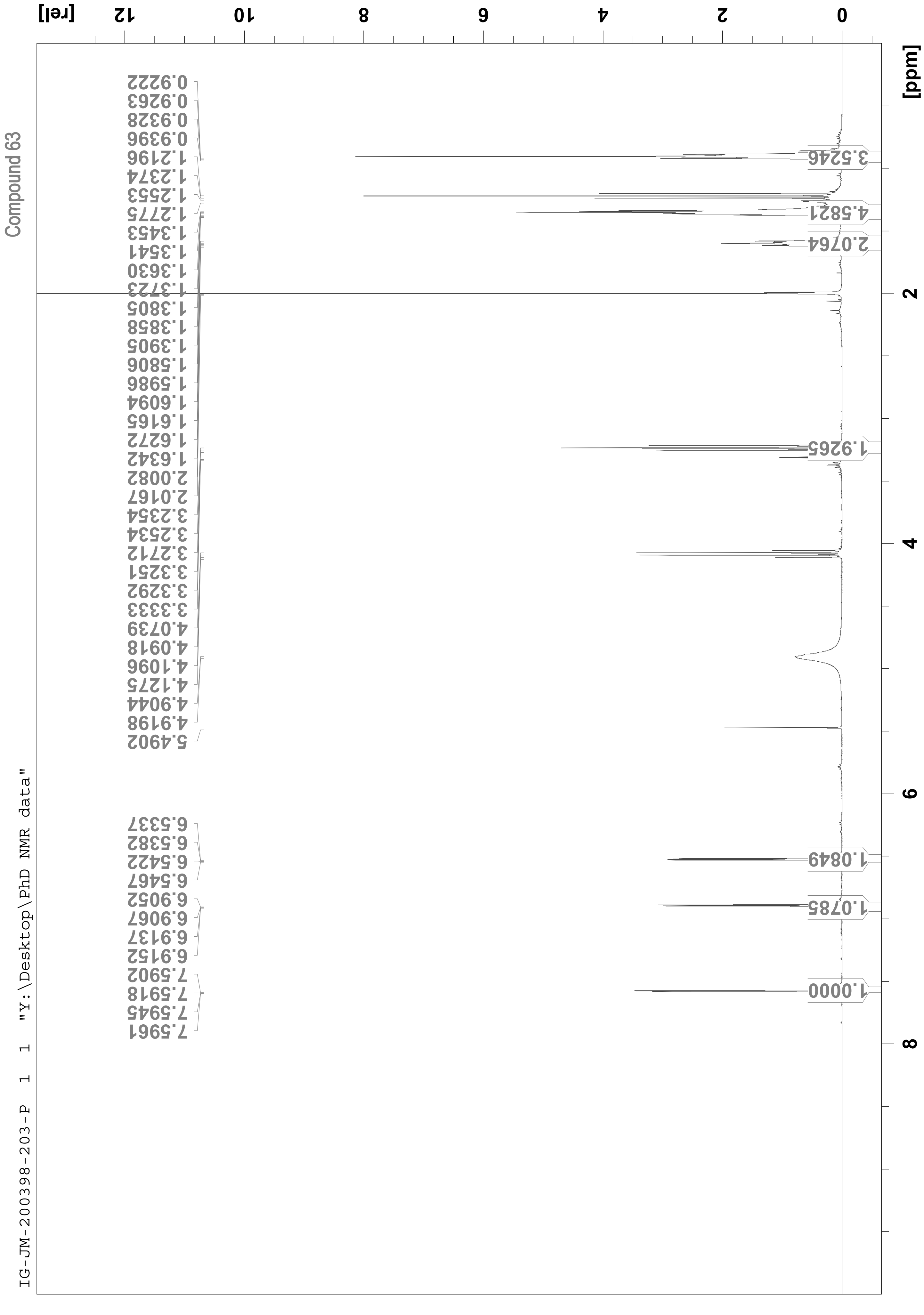
Compound 63
IG-JM-200398-203-P 1 1 "Y:\Desktop\PhD NMR data"
[rel]
[ppm]
0.9222
0.9263
0.9328
0.9396
1.2196
1.2374
1.2553
1.2775
1.3453
1.3541
1.3630
1.3723
1.3805
1.3858
1.3905
1.5806
1.5986
1.6094
1.6165
1.6272
1.6342
2.0082
2.0167
3.2354
3.2534
3.2712
3.3251
3.3292
3.3333
4.0739
4.0918
4.1096
4.1275
4.9044
4.9198
5.4902
6.5337
6.5382
6.5422
6.5467
6.9052
6.9067
6.9137
6.9152
7.5902
7.5918
7.5945
7.5961
3.5246
4.5821
2.0764
1.9265
1.0849
1.0785
1.0000

IG-JM-200398-309-001 1 1 "Y:\Desktop\PhD NMR data"

Compound 66

PROTON.DAY CDCl3 {C:\Bruker\TopSpin3.2} DDU500 17

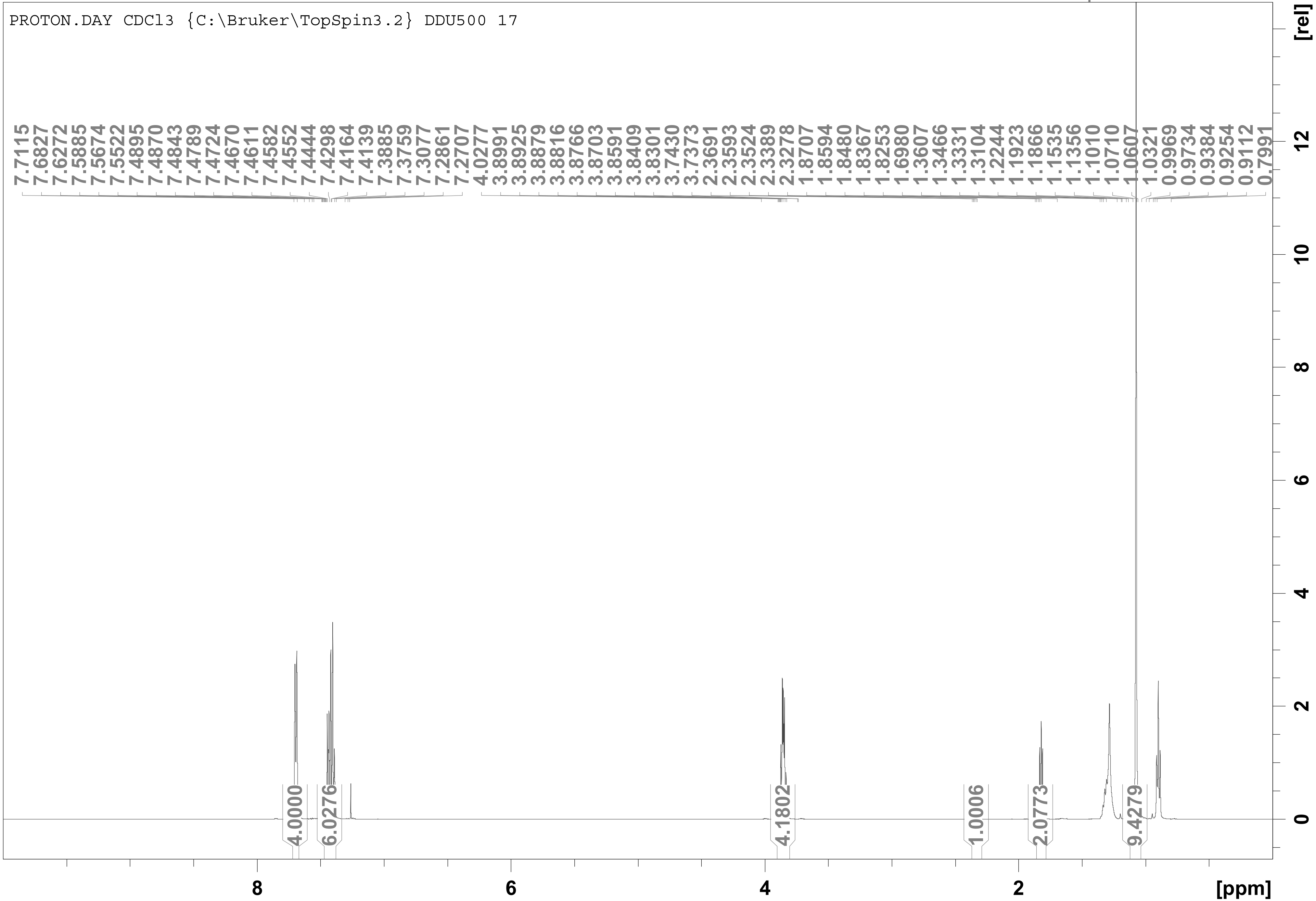

PROTON.DAY CDCl3 {C:\Bruker\TopSpin3.2} DDU500 20

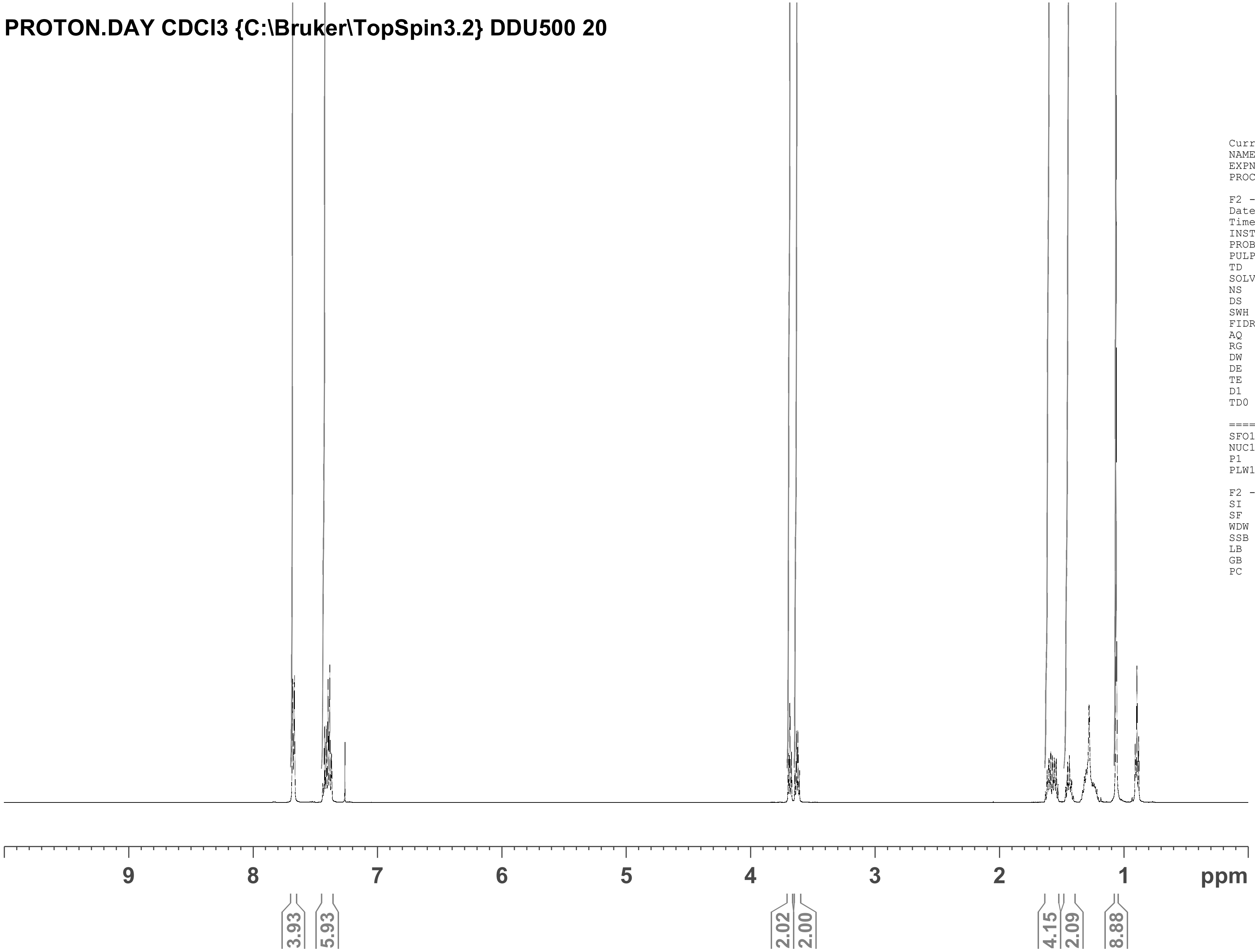


```
Current Data Parameters
NAME     IG-JM-200398-322-001
EXPNO                   1
PROCNO                  1

F2 - Acquisition Parameters
Date_          20180117
Time              14.46
INSTRUM           spect
PROBHD   5 mm PABBO BB/
PULPROG            zg30
TD                65536
SOLVENT           CDCl3
NS                   16
DS                    2
SWH           10000.000 Hz
FIDRES         0.152588 Hz
AQ            3.2767999 sec
RG                 80.6
DW               50.000 usec
DE                 6.50 usec
TE                303.2 K
D1           1.00000000 sec
TD0                   1

======== CHANNEL f1 ========
SFO1        500.1330885 MHz
NUC1                 1H
P1                10.00 usec
PLW1        20.85000038 W

F2 - Processing parameters
SI                65536
SF          500.1300131 MHz
WDW                  EM
SSB      0
LB                 0.30 Hz
GB       0
PC                 1.00
```

Compound 67

Compound 68

IG-JM-200398-312-001 1 1 "Y:\Desktop\PhD NMR data"

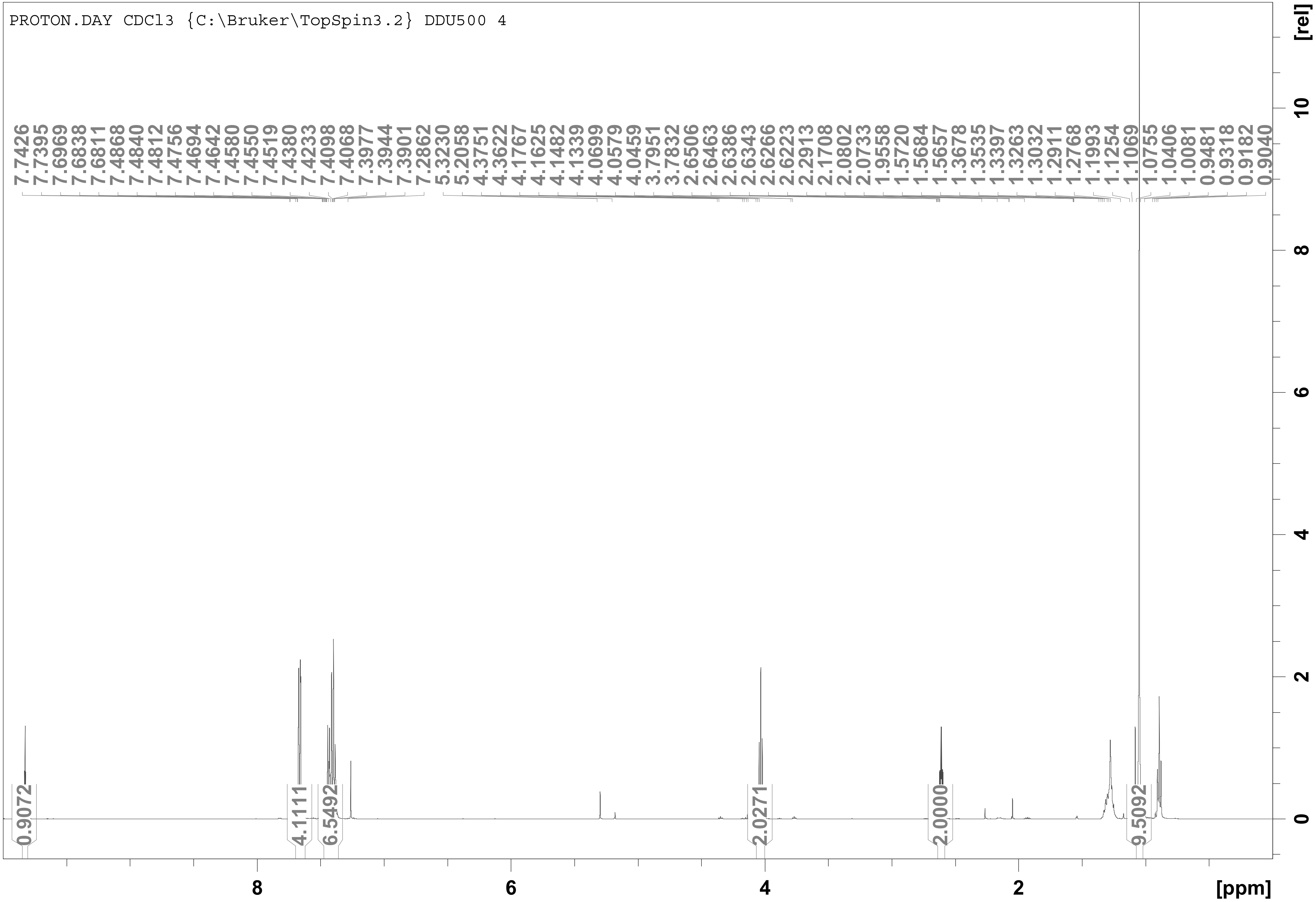

PROTON.DAY CDCl3 {C:\Bruker\TopSpin3.2} DDU400 5

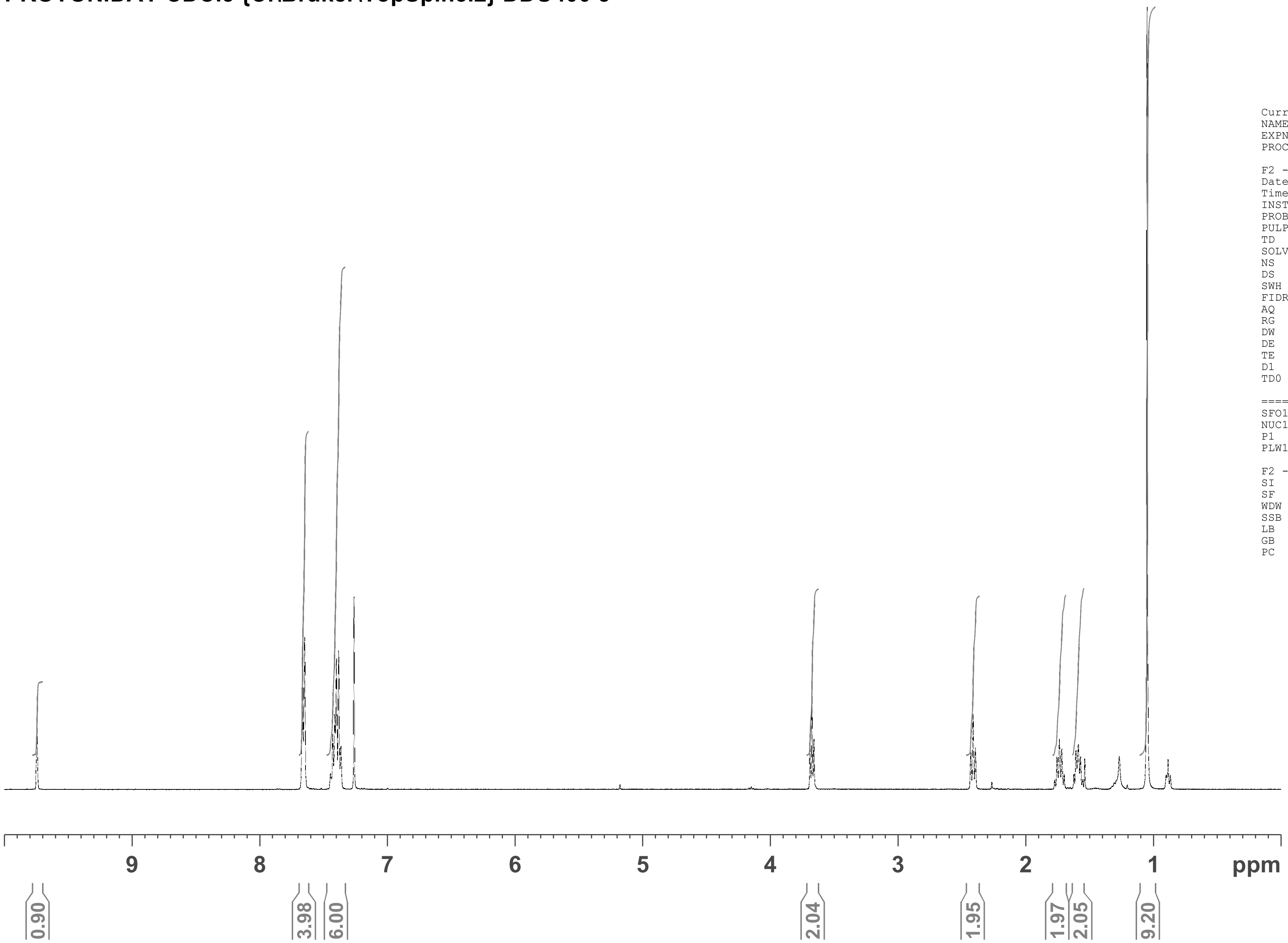


```
Current Data Parameters
NAME     IG-JM-200398-376-001
EXPNO                   1
PROCNO                  1

F2 - Acquisition Parameters
Date_           20190117
Time               10.07
INSTRUM            spect
PROBHD   5 mm PADUL 13C
PULPROG             zg30
TD                 65536
SOLVENT            CDCl3
NS                    16
DS                     2
SWH             8000.000 Hz
FIDRES          0.122070 Hz
AQ             4.0960002 sec
RG                196.14
DW                62.500 usec
DE                 11.07 usec
TE                 294.6 K
D1            1.00000000 sec
TD0                    1

======== CHANNEL f1 ========
SFO1         400.1324710 MHz
NUC1                  1H
P1                 10.00 usec
PLW1         20.00000000 W

F2 - Processing parameters
SI                 65536
SF           400.1300098 MHz
WDW                   EM
SSB      0
LB                  0.30 Hz
GB       0
PC                  1.00
```

Compound 69

327

IG-JM-200398-237-001 1 1 "Y:\Desktop\PhD NMR data"

Compound 70

PROTON.DAY CDCl3 {C:\Bruker\TopSpin3.2} DDU500 50

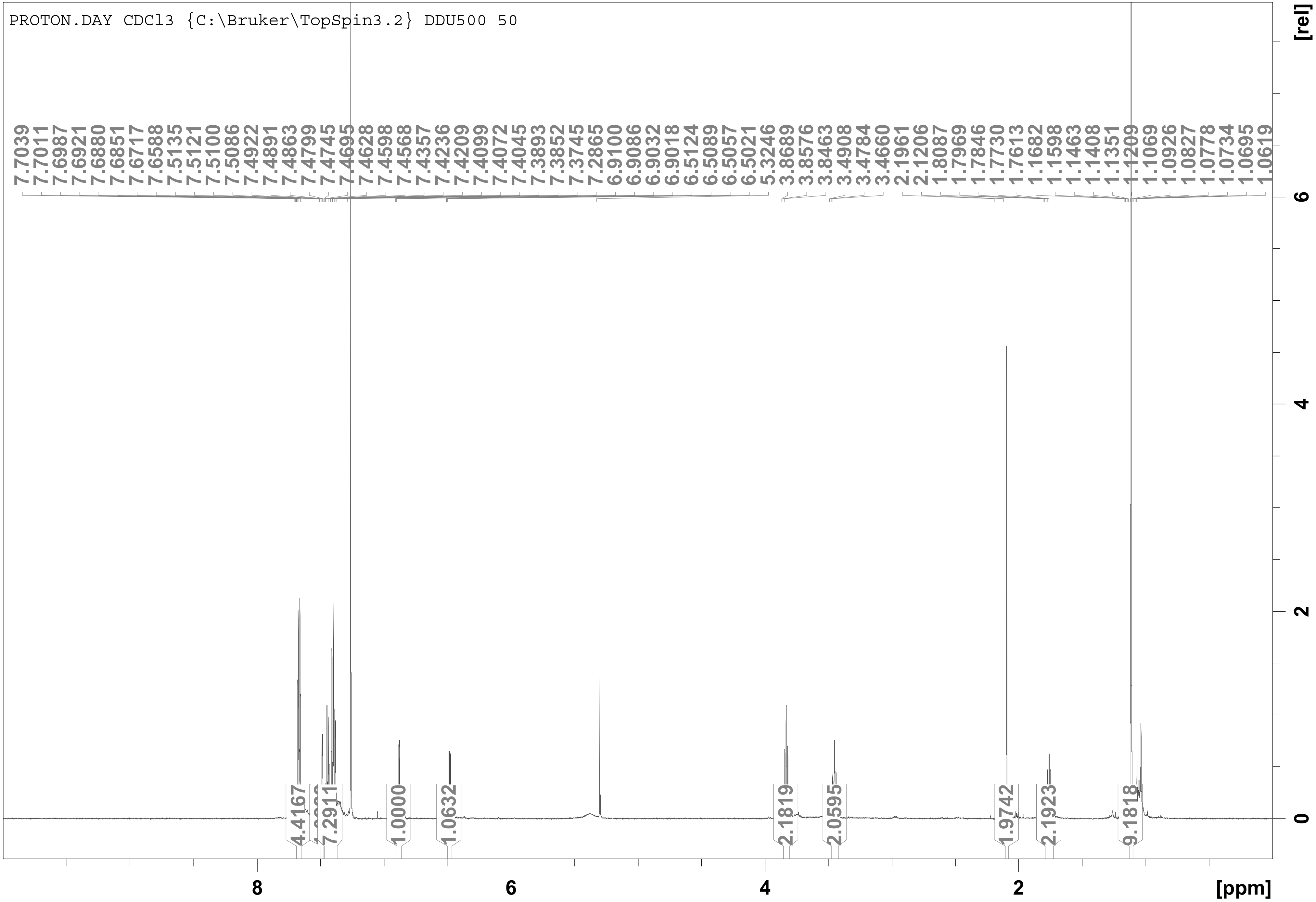

Analytical Studio Reviewer Report

Sample Name: JM-200398-327-001
Location: 1,1:D,2
Acquired: 30-Jan-18 11:42 AM
Filename: JM-200398-327-001_027555.D
Instrument: 5-90 MECN POS EZX_3M.M

Compound 70

Peak #	Time	TIC(+)	Area % UV254	UV210	BPM
1	0.174	10.0	62.3	63.0	130.2
2	0.383	57.1	0.0	0.0	100.2
3	0.452	0.0	0.0	35.3	100.2
4	1.931	32.9	37.7	1.7	447.3

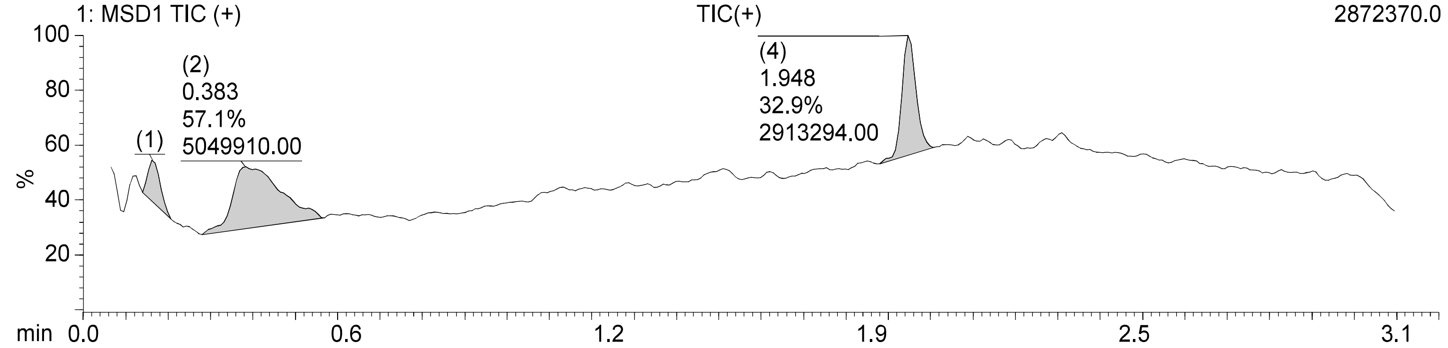

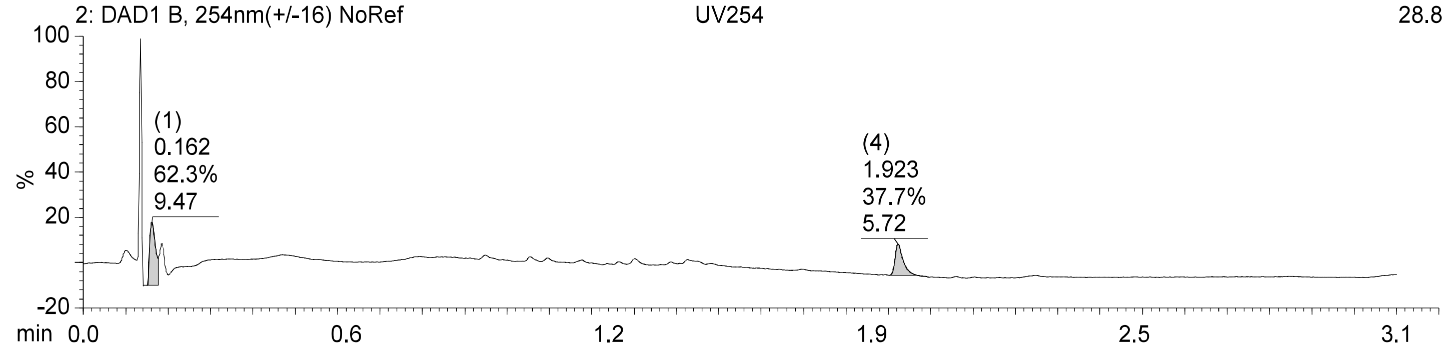

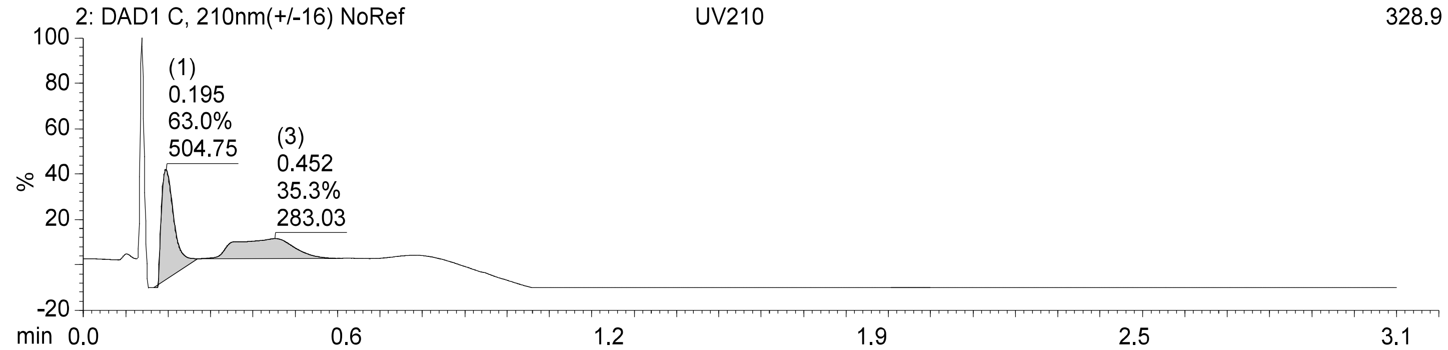

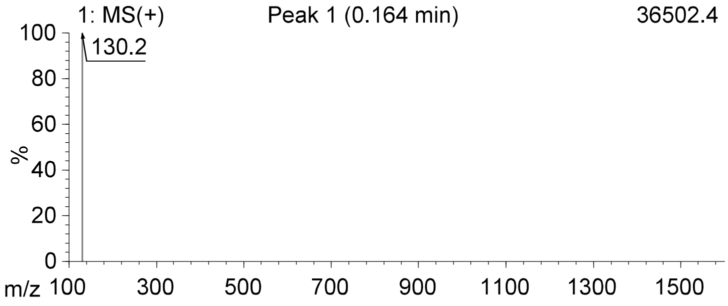

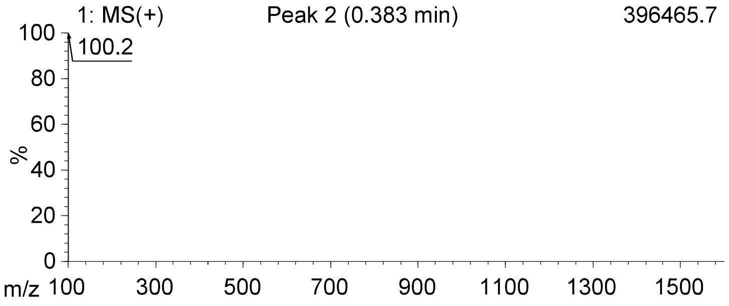

Printed: 30-Jan-18 11:45 AM

Analytical Studio Reviewer Report

Sample Name: JM-200398-327-001 **Acquired:** 30-Jan-18 11:42 AM **Instrument:** 5-90 MECN POS EZX_3M.M
Location: 1,1:D,2 **Filename:** JM-200398-327-001_027555.D

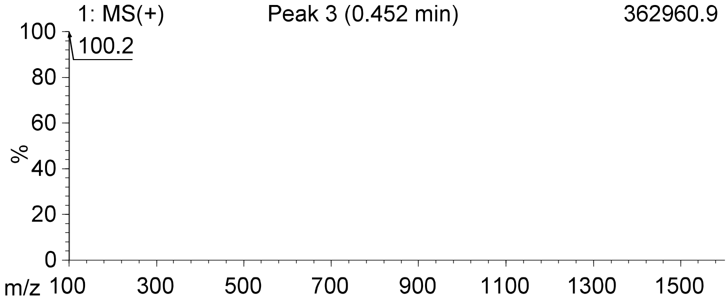

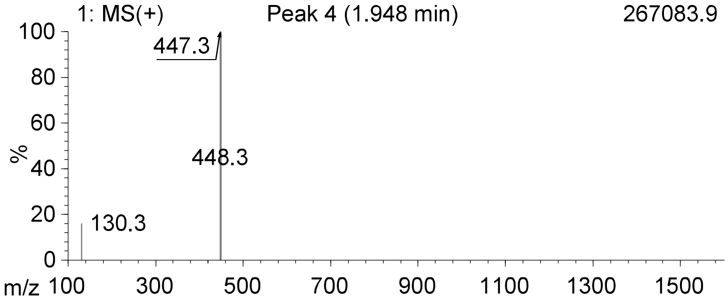

Printed: 30-Jan-18 11:45 AM

IG-JM-200398-328-001 1 1 "Y:\Desktop\PhD NMR data"

Compound 71

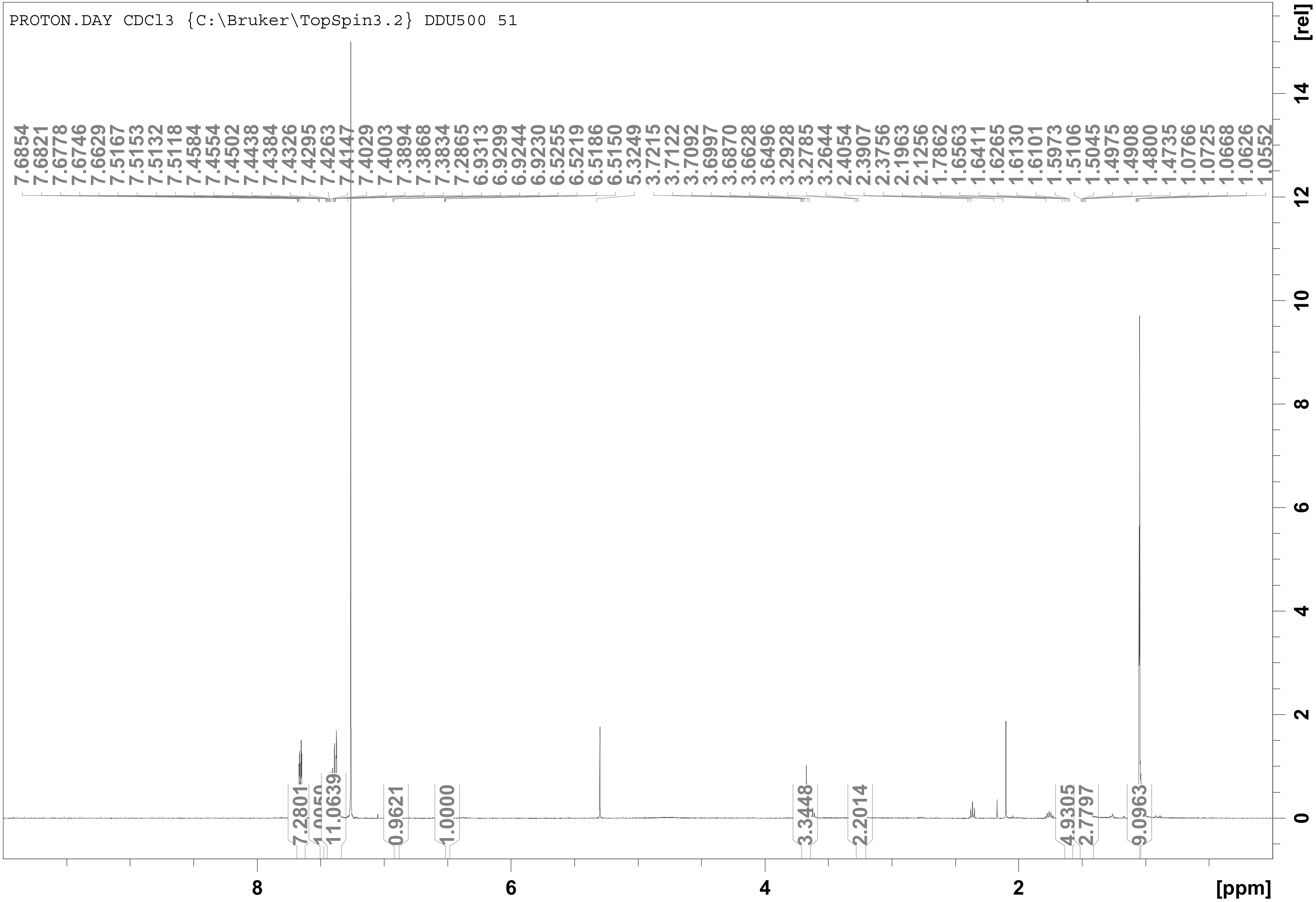

Analytical Studio Reviewer Report

Compound 71

Sample Name: JM-200398-328-001
Location: 1,1:D,3
Acquired: 30-Jan-18 11:46 AM
Filename: JM-200398-328-001_027556.D
Instrument: 5-90 MECN POS EZX_3M.M

		Area %			
Peak #	Time	TIC(+)	UV254	UV210	BPM
1	0.163	7.8	51.9	0.0	130.2
2	0.183	0.0	48.1	59.6	100.2
3	0.299	44.4	0.0	20.0	100.2
4	0.463	0.0	0.0	19.1	100.2
5	2.040	47.8	0.0	1.3	475.3

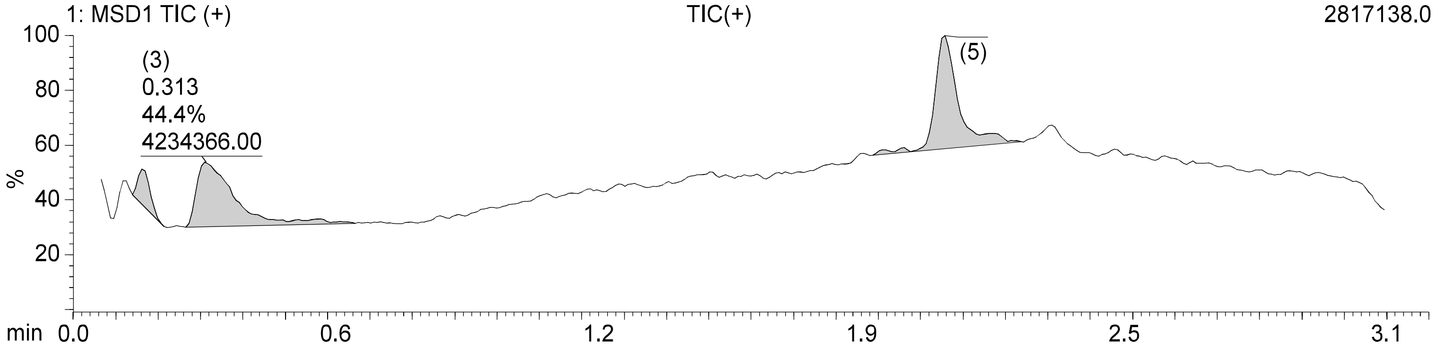

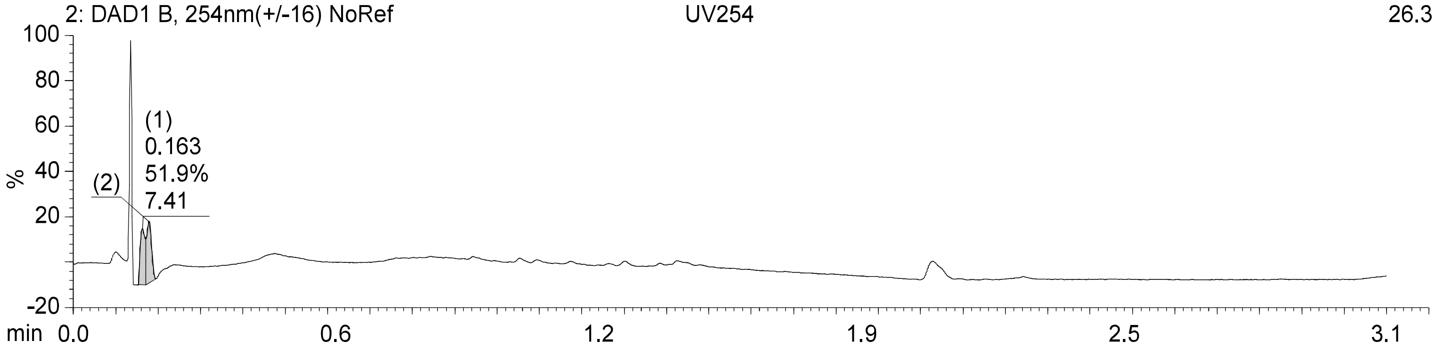

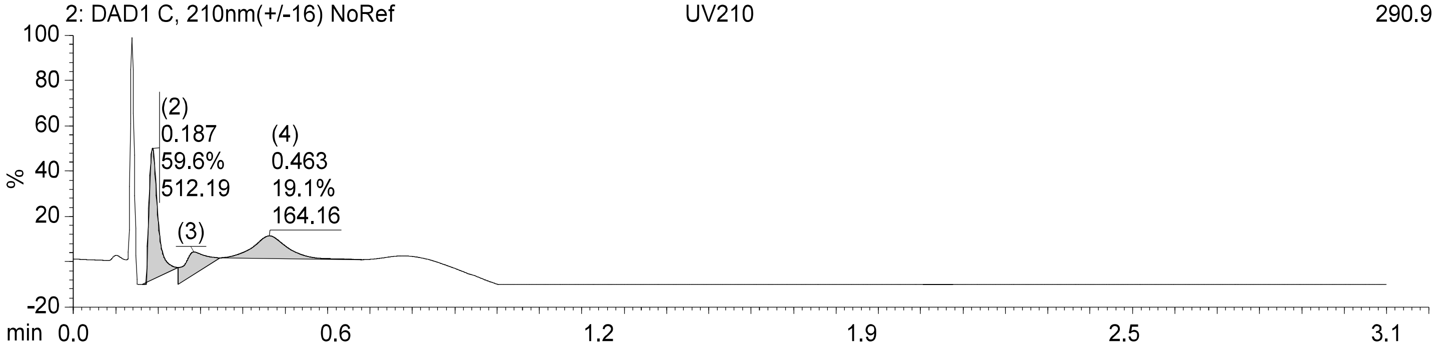

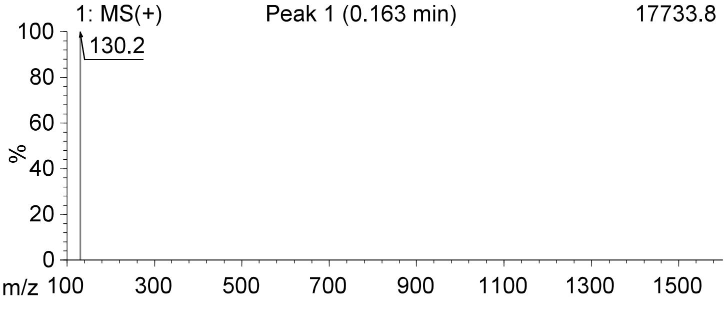

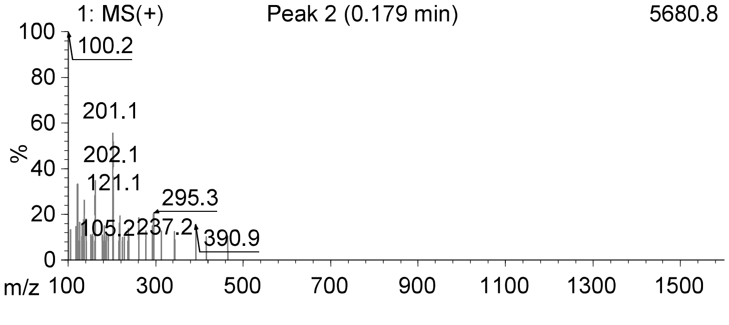

Printed: 30-Jan-18 11:49 AM

Analytical Studio Reviewer Report

Sample Name: JM-200398-328-001 **Acquired:** 30-Jan-18 11:46 AM **Instrument:** 5-90 MECN POS EZX_3M.M
Location: 1,1:D,3 **Filename:** JM-200398-328-001_027556.D

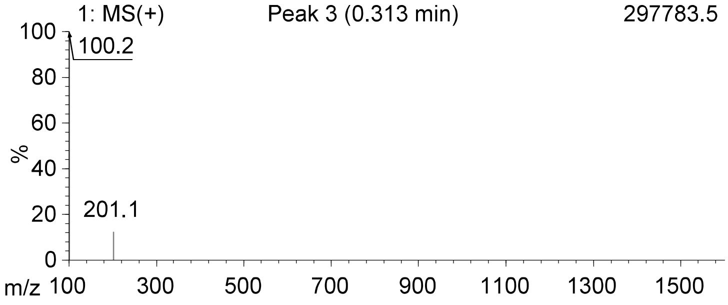

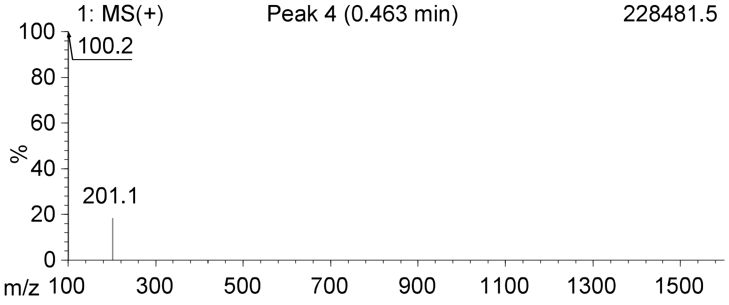

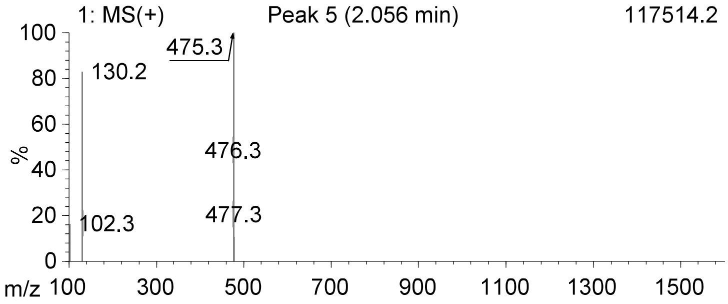

 Printed: 30-Jan-18 11:49 AM

Compound 72

PROTON.DAY DMSO {C:\Bruker\TopSpin3.2} DDU500 8

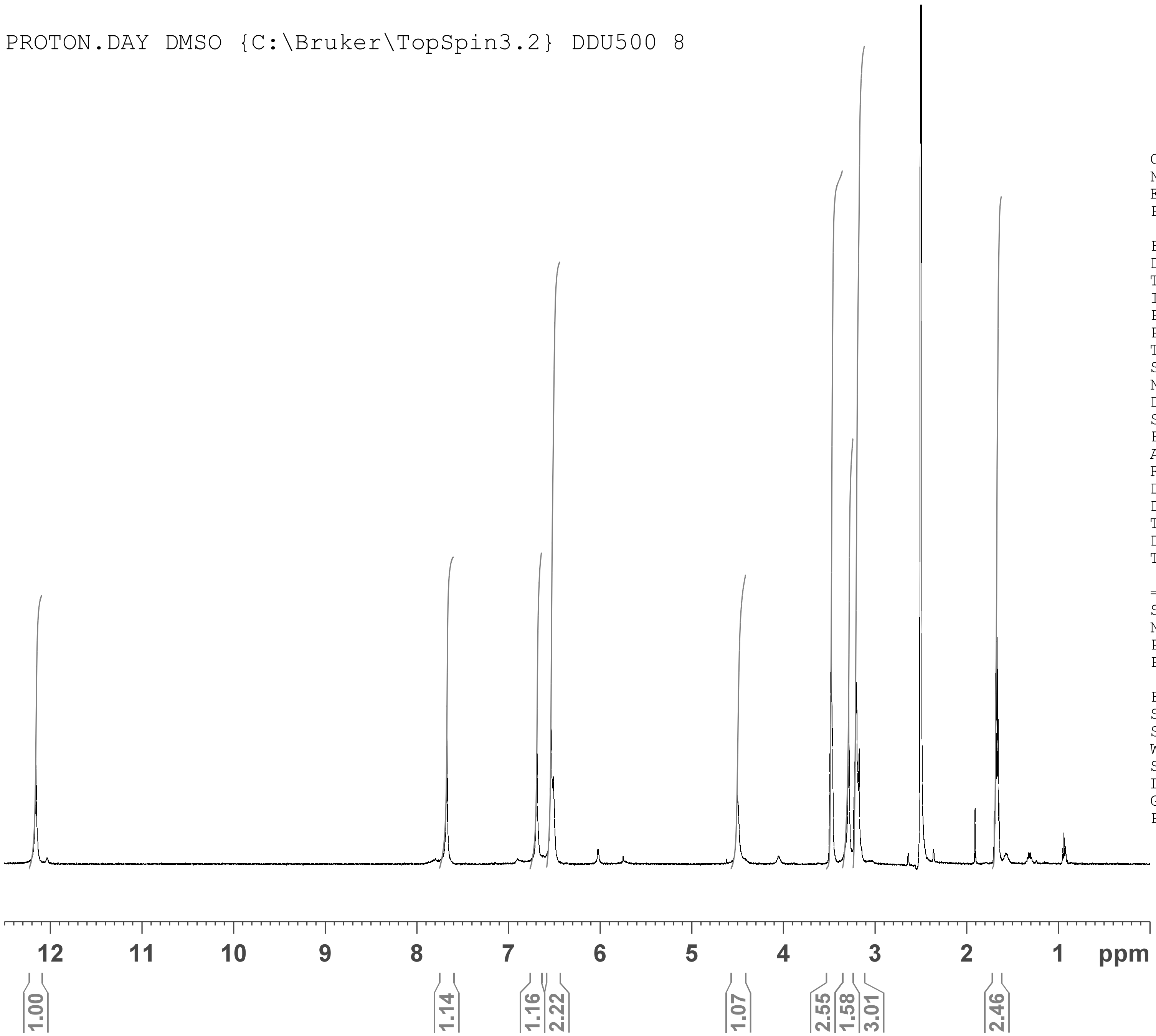


```
Current Data Parameters
NAME      IG-JM-200398-382-001
EXPNO                        1
PROCNO                       1

F2 - Acquisition Parameters
Date_             20190121
Time                 12.19
INSTRUM              spect
PROBHD    5 mm PABBO BB/
PULPROG               zg30
TD                   65536
SOLVENT               DMSO
NS                      16
DS                       2
SWH              10000.000 Hz
FIDRES            0.152588 Hz
AQ               3.2767999 sec
RG                     181
DW                  50.000 usec
DE                    6.50 usec
TE                   303.1 K
D1              1.00000000 sec
TD0                      1

======== CHANNEL f1 ========
SFO1           500.1330885 MHz
NUC1                    1H
P1                   10.00 usec
PLW1           20.85000038 W

F2 - Processing parameters
SI                   65536
SF             500.1300053 MHz
WDW                     EM
SSB                      0
LB                    0.30 Hz
GB                       0
PC                    1.00
```

Compound 73

PROTON.DAY DMSO {C:\Bruker\TopSpin3.2} DDU500 9

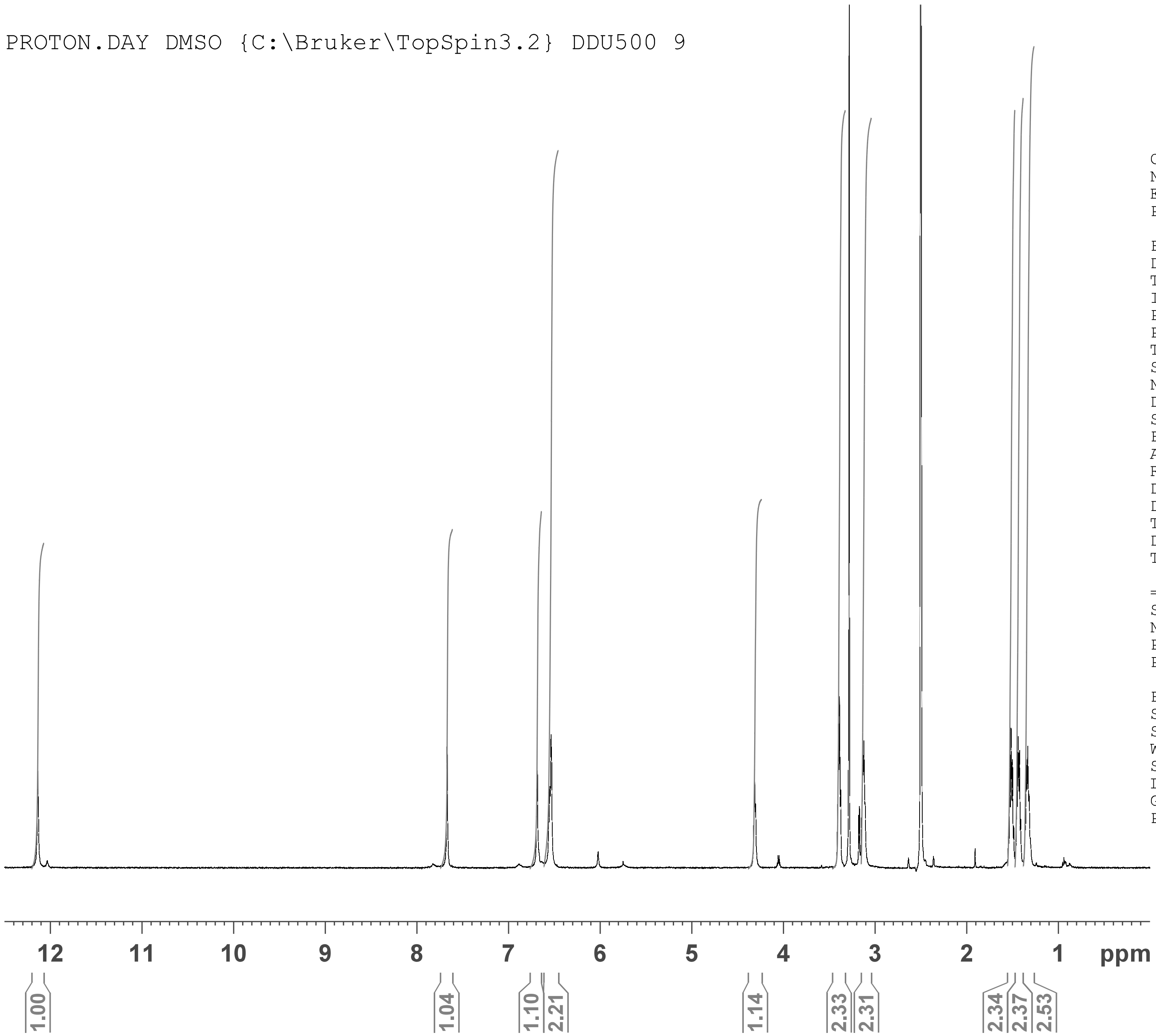


```
Current Data Parameters
NAME     IG-JM-200398-383-001
EXPNO                       1
PROCNO                      1

F2 - Acquisition Parameters
Date_          20190121
Time              12.24
INSTRUM           spect
PROBHD   5 mm PABBO BB/
PULPROG            zg30
TD                65536
SOLVENT            DMSO
NS                   16
DS                    2
SWH           10000.000 Hz
FIDRES         0.152588 Hz
AQ            3.2767999 sec
RG                  181
DW               50.000 usec
DE                 6.50 usec
TE                303.1 K
D1           1.00000000 sec
TD0                   1

======== CHANNEL f1 ========
SFO1        500.1330885 MHz
NUC1                 1H
P1                10.00 usec
PLW1        20.85000038 W

F2 - Processing parameters
SI                65536
SF          500.1300053 MHz
WDW                  EM
SSB                   0
LB                 0.30 Hz
GB                    0
PC                 1.00
```

PROTON.DAY CDCl3 {C:\Bruker\TopSpin3.2} DDU500 19

Compound 75

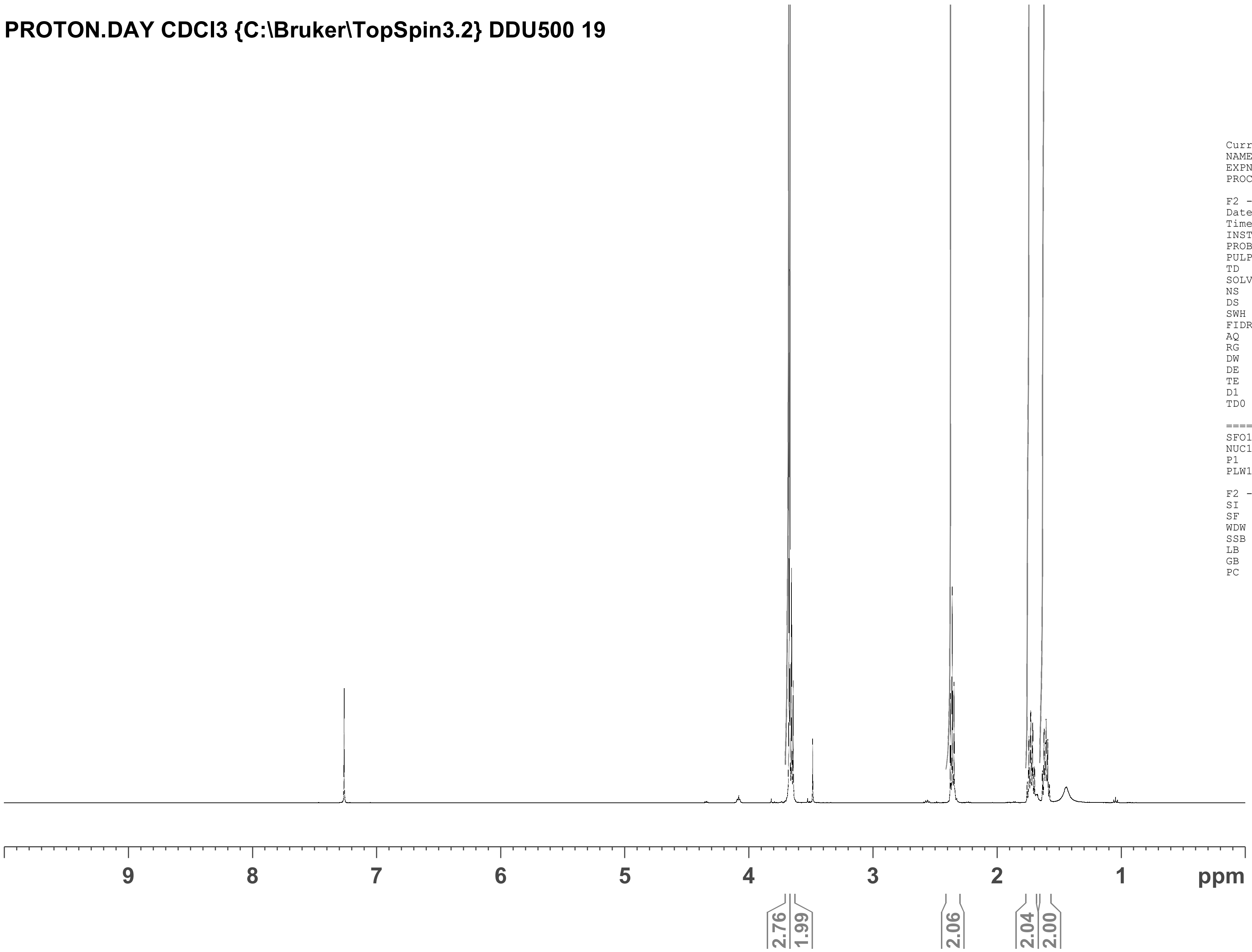


```
Current Data Parameters
NAME      IG-JM-200398-374-001
EXPNO                    1
PROCNO                   1

F2 - Acquisition Parameters
Date_             20190114
Time                 17.09
INSTRUM              spect
PROBHD    5 mm PABBO BB/
PULPROG               zg30
TD                   65536
SOLVENT              CDCl3
NS                      16
DS                       2
SWH             10000.000 Hz
FIDRES           0.152588 Hz
AQ              3.2767999 sec
RG                     181
DW                  50.000 usec
DE                    6.50 usec
TE                   303.1 K
D1              1.00000000 sec
TD0                      1

======== CHANNEL f1 ========
SFO1         500.1330885 MHz
NUC1                    1H
P1                   10.00 usec
PLW1           20.85000038 W

F2 - Processing parameters
SI                   65536
SF           500.1300129 MHz
WDW                     EM
SSB      0
LB                    0.30 Hz
GB       0
PC                    1.00
```

Analytical Studio Reviewer Report

Compound 75

Sample Name: JM-200398-374-001
Location: 1,1:A,6
Acquired: 15-Jan-19 2:44 PM
Filename: JM-200398-374-001_048030.D
Instrument: 5-90 MECN POS EZX_3M.M

		Area %			
Peak #	Time	TIC(+)	UV254	UV210	BPM
1	0.173	53.7	100.0	5.6	102.2
2	0.473	0.0	0.0	56.9	100.1
3	0.790	0.0	0.0	37.5	100.1
4	2.334	46.3	0.0	0.0	282.2

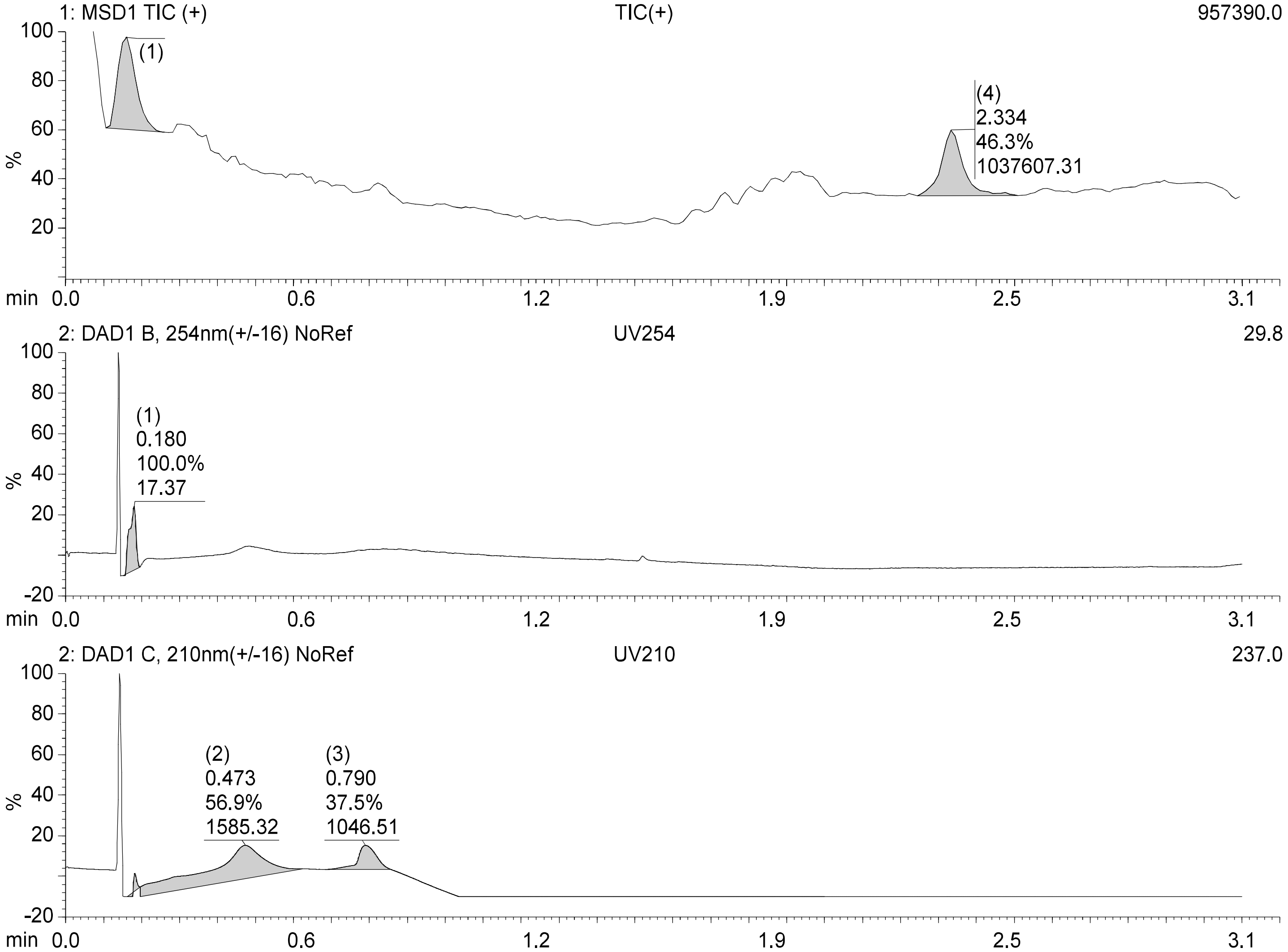

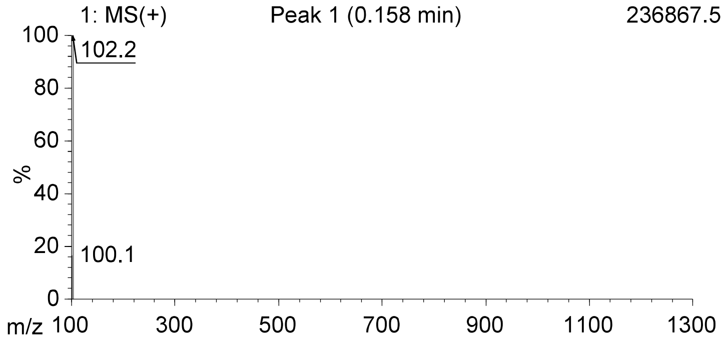

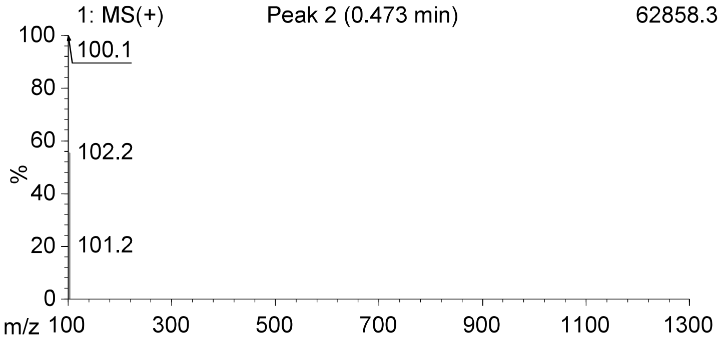

Printed: 15-Jan-19 2:47 PM

Analytical Studio Reviewer Report

Sample Name: JM-200398-374-001
Location: 1,1:A,6
Acquired: 15-Jan-19 2:44 PM
Filename: JM-200398-374-001_048030.D
Instrument: 5-90 MECN POS EZX_3M.M

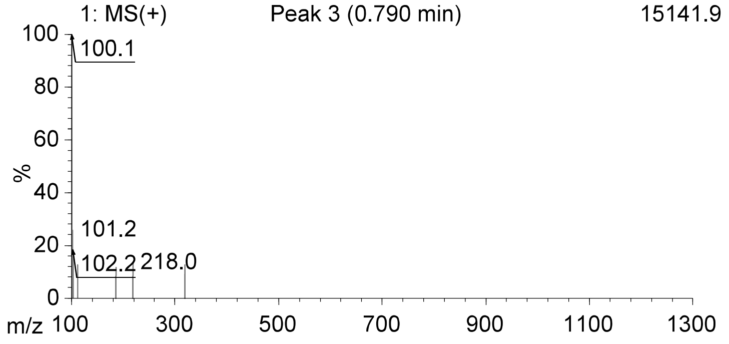

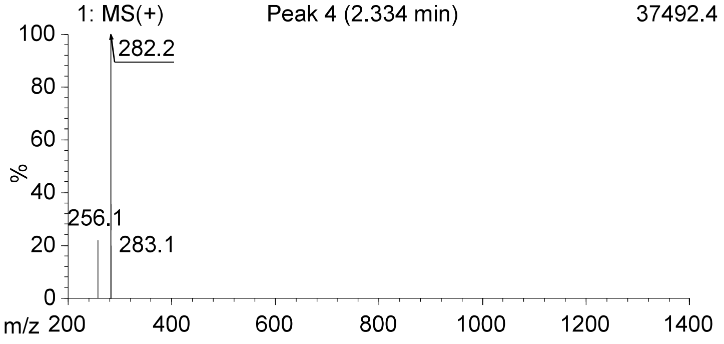

Printed: 15-Jan-19 2:47 PM

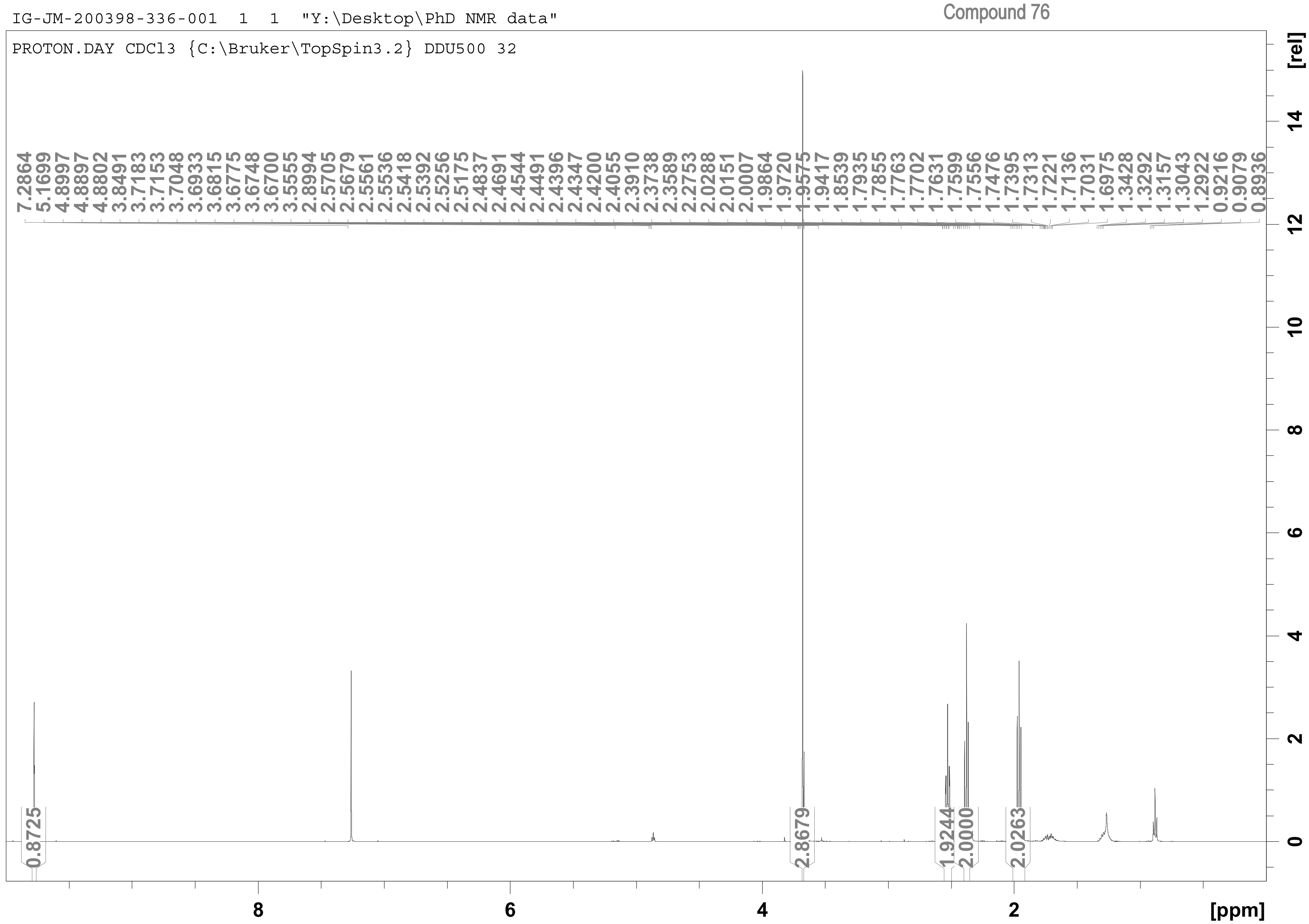
IG-JM-200398-336-001 1 1 "Y:\Desktop\PhD NMR data"
Compound 76
PROTON.DAY CDCl3 {C:\Bruker\TopSpin3.2} DDU500 32
7.2864
5.1699
4.8997
4.8897
4.8802
3.8491
3.7183
3.7153
3.7048
3.6933
3.6815
3.6775
3.6748
3.6700
3.5555
2.8994
2.5705
2.5679
2.5561
2.5536
2.5418
2.5392
2.5256
2.5175
2.4837
2.4691
2.4544
2.4491
2.4396
2.4347
2.4200
2.4055
2.3910
2.3738
2.3589
2.2753
2.0288
2.0151
2.0007
1.9864
1.9720
1.9575
1.9417
1.8539
1.7935
1.7855
1.7763
1.7702
1.7631
1.7599
1.7556
1.7476
1.7395
1.7313
1.7221
1.7136
1.7031
1.6975
1.3428
1.3292
1.3157
1.3043
1.2922
0.9216
0.9079
0.8936
0.8725
2.8679
1.9244
2.0000
2.0263
[rel]
14
12
10
8
6
4
2
0
[ppm]

Compound 77

PROTON.DAY DMSO {C:\Bruker\TopSpin3.2} DDU500 10

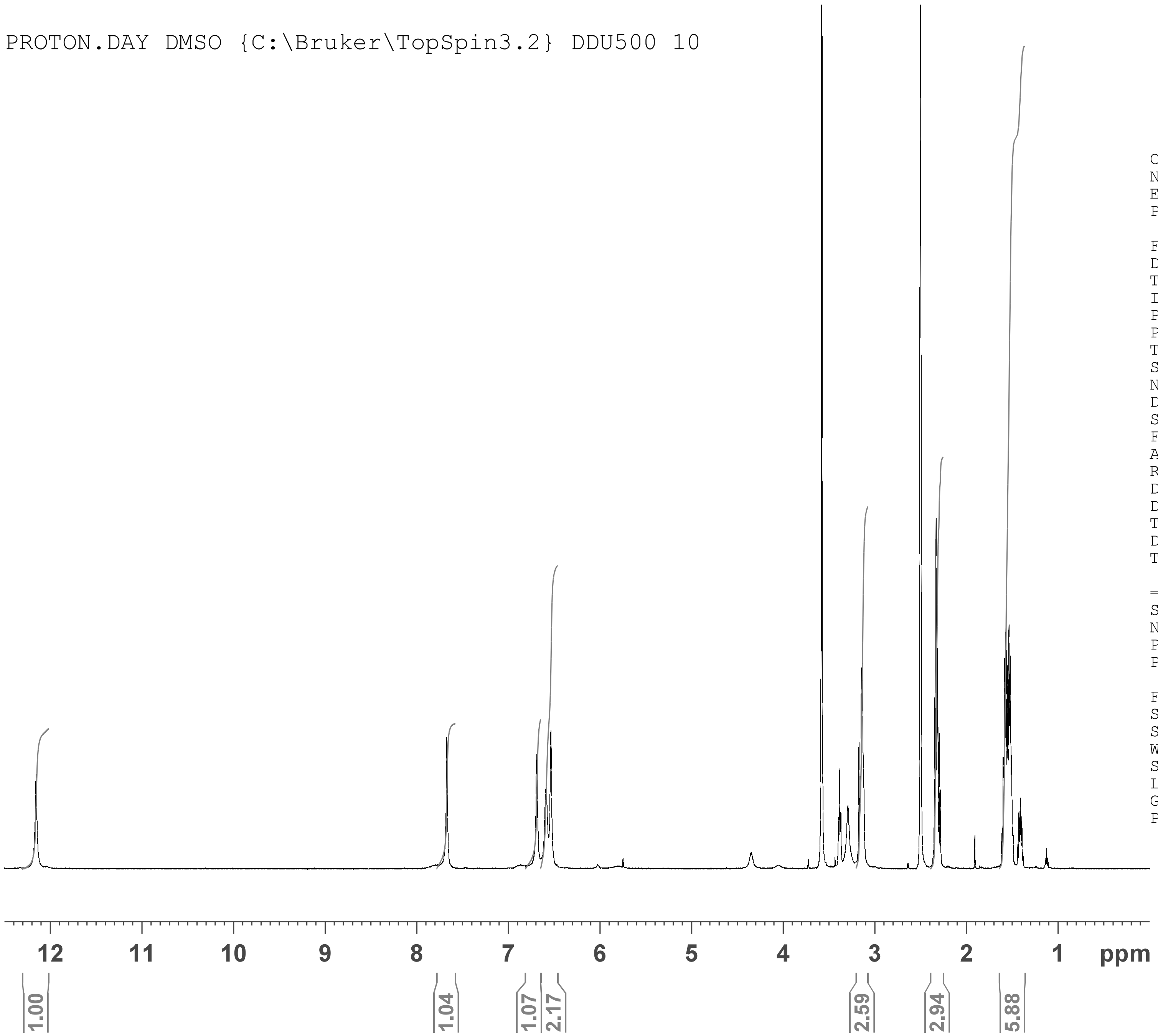


```
Current Data Parameters
NAME     IG-JM-200398-384-001
EXPNO                       1
PROCNO                      1

F2 - Acquisition Parameters
Date_            20190121
Time                12.29
INSTRUM             spect
PROBHD   5 mm PABBO BB/
PULPROG              zg30
TD                  65536
SOLVENT              DMSO
NS                     16
DS                      2
SWH             10000.000 Hz
FIDRES           0.152588 Hz
AQ              3.2767999 sec
RG                    181
DW                 50.000 usec
DE                   6.50 usec
TE                  303.1 K
D1             1.00000000 sec
TD0                     1

======== CHANNEL f1 ========
SFO1          500.1330885 MHz
NUC1                   1H
P1                  10.00 usec
PLW1          20.85000038 W

F2 - Processing parameters
SI                  65536
SF            500.1300056 MHz
WDW                    EM
SSB                     0
LB                   0.30 Hz
GB                      0
PC                   1.00
```

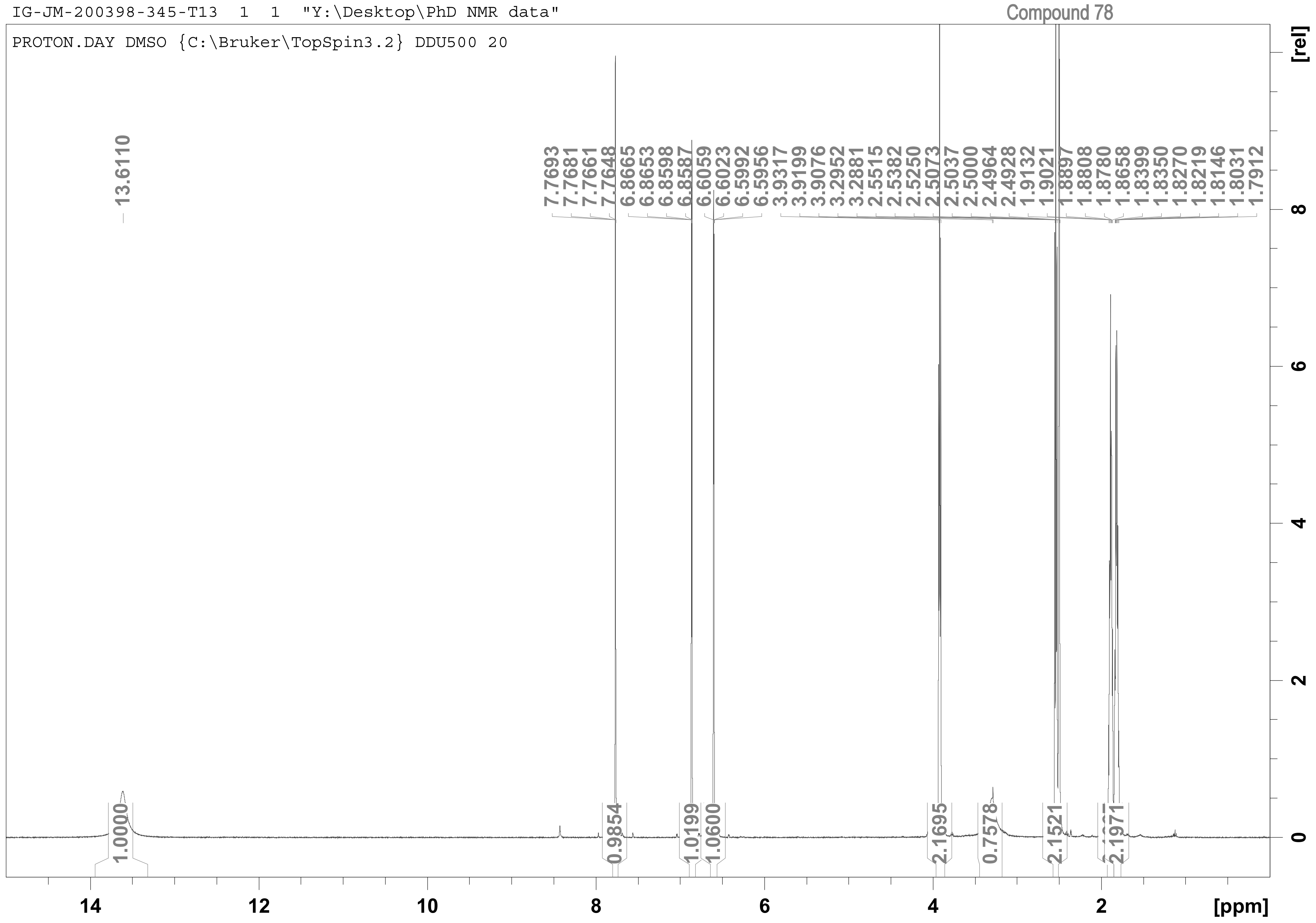
IG-JM-200398-345-T13 1 1 "Y:\Desktop\PhD NMR data"
PROTON.DAY DMSO {C:\Bruker\TopSpin3.2} DDU500 20
Compound 78
13.6110
7.7693
7.7681
7.7661
7.7648
6.8665
6.8653
6.8598
6.8587
6.6059
6.6023
6.5992
6.5956
3.9317
3.9199
3.9076
3.2952
3.2881
2.5515
2.5382
2.5250
2.5073
2.5037
2.5000
2.4964
2.4928
1.9132
1.9021
1.8897
1.8808
1.8780
1.8658
1.8399
1.8350
1.8270
1.8219
1.8146
1.8031
1.7912
1.0000
0.9854
1.0199
1.0600
2.1695
0.7578
2.1521
2.1971
[rel]
[ppm]
14
12
10
8
6
4
2
0

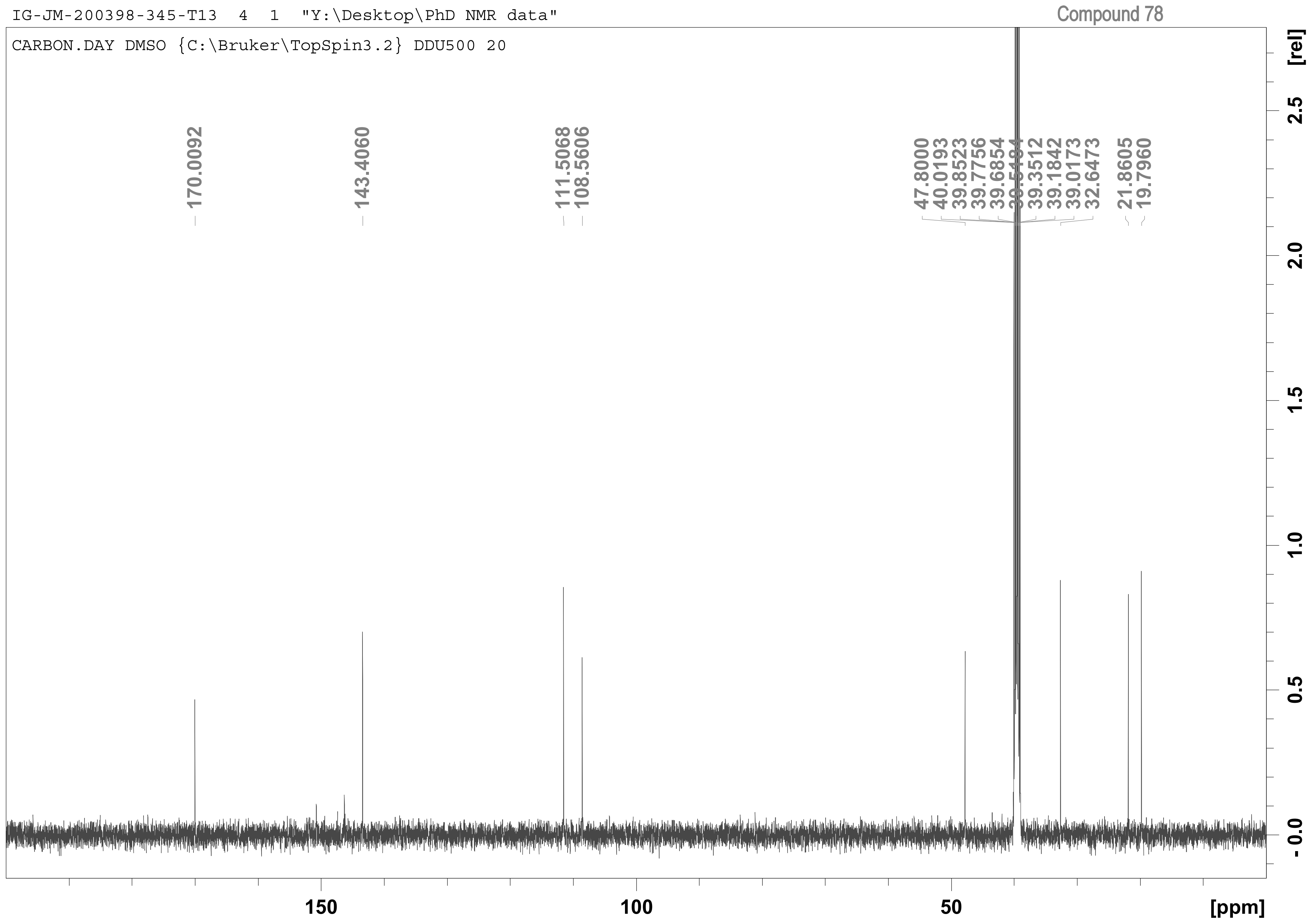
IG-JM-200398-345-T13 4 1 "Y:\Desktop\PhD NMR data"
CARBON.DAY DMSO {C:\Bruker\TopSpin3.2} DDU500 20
Compound 78
170.0092
143.4060
111.5068
108.5606
47.8000
40.0193
39.8523
39.7756
39.6854
39.3512
39.1842
39.0173
32.6473
21.8605
19.7960
[rel]
2.5
2.0
1.5
1.0
0.5
-0.0
150
100
50
[ppm]

Compound 79

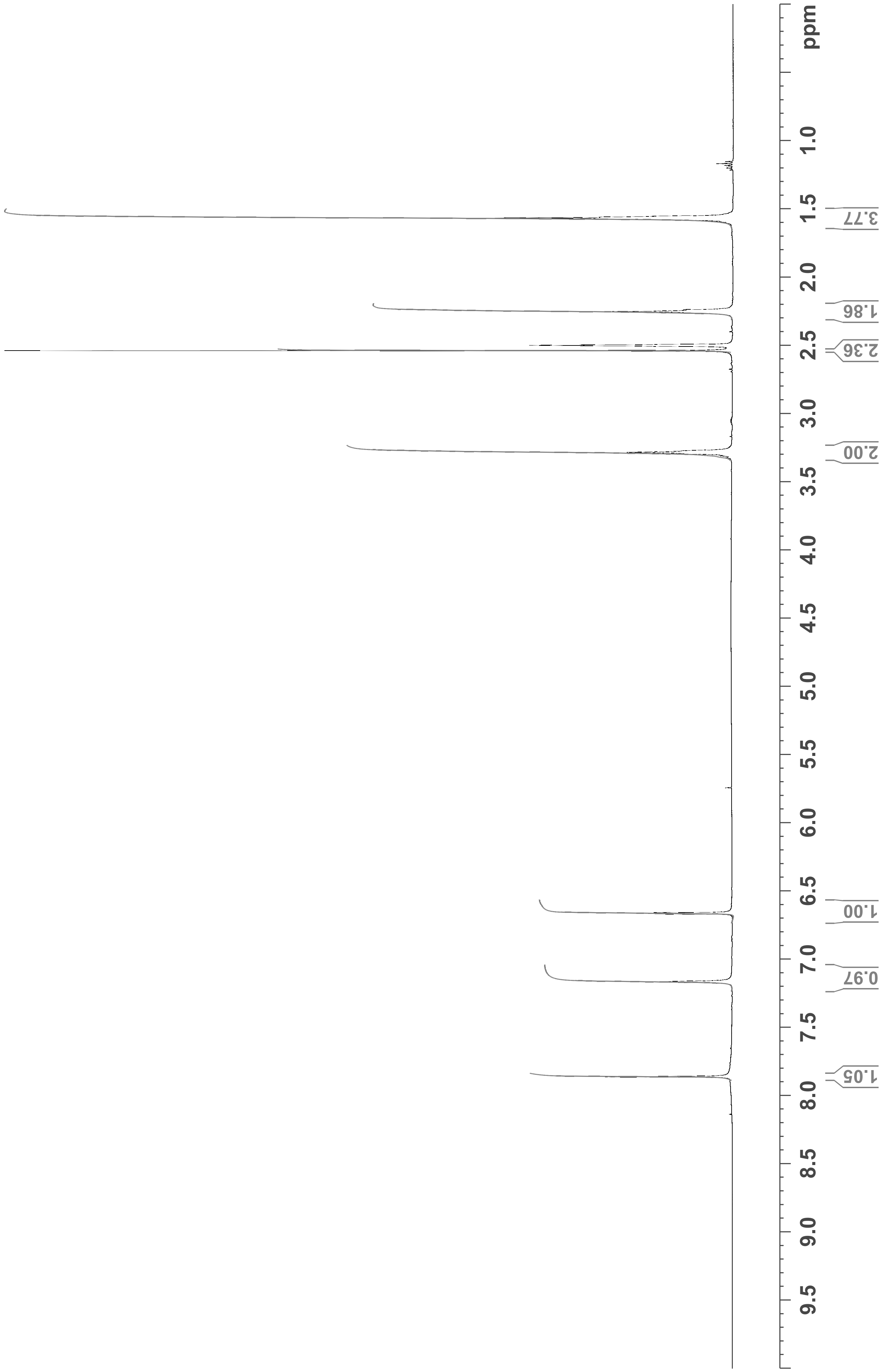
ppm
1.0
1.5
2.0
2.5
3.0
3.5
4.0
4.5
5.0
5.5
6.0
6.5
7.0
7.5
8.0
8.5
9.0
9.5
3.77
1.86
2.36
2.00
1.00
0.97
1.05

PROTON.DAY CDCl3 {C:\Bruker\TopSpin3.2} DDU500 37

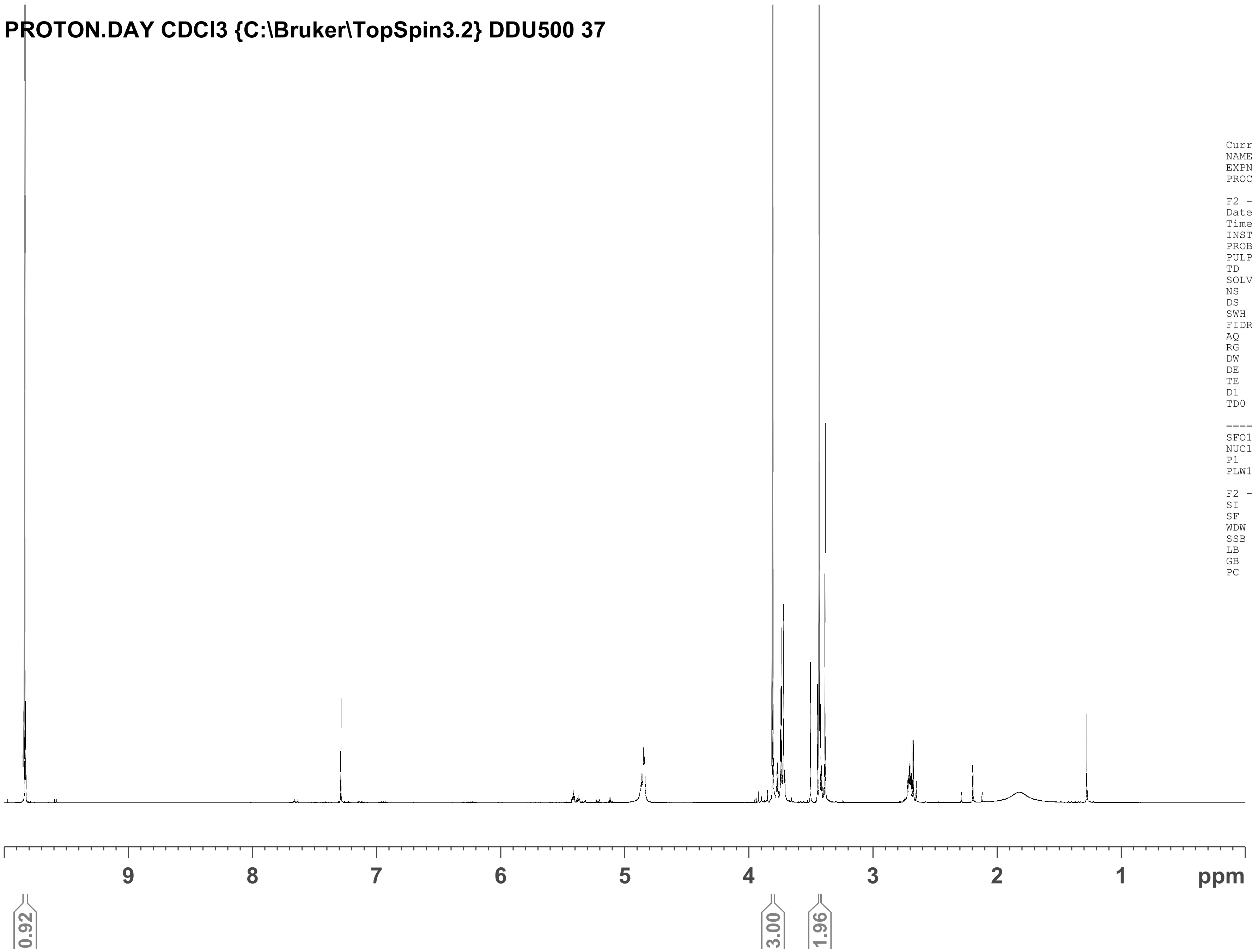

Compound 81

```
Current Data Parameters
NAME       IG-JM-200398-364-02
EXPNO                    1
PROCNO                   1

F2 - Acquisition Parameters
Date_             20180730
Time                 17.19
INSTRUM              spect
PROBHD   5 mm PABBO BB/
PULPROG               zg30
TD                   65536
SOLVENT              CDCl3
NS                      16
DS                       2
SWH           10000.000 Hz
FIDRES         0.152588 Hz
AQ            3.2767999 sec
RG                     128
DW               50.000 usec
DE                 6.50 usec
TE                303.1 K
D1           1.00000000 sec
TD0                      1

======== CHANNEL f1 ========
SFO1        500.1330885 MHz
NUC1                    1H
P1                10.00 usec
PLW1        20.85000038 W

F2 - Processing parameters
SI                   65536
SF          500.1300000 MHz
WDW                     EM
SSB      0
LB                    0.30 Hz
GB       0
PC                    1.00
```
